# Dual‐Ligand Strategy in Rh‐Catalyzed Sequential Hydrofunctionalization of Valylene

**DOI:** 10.1002/advs.202511331

**Published:** 2025-07-30

**Authors:** Yong‐Kang Mei, Su‐Yang Xu, Zhi‐Hui Wang, Ding‐Wei Ji, Qing‐An Chen

**Affiliations:** ^1^ Dalian Institute of Chemical Physics Chinese Academy of Sciences Dalian 116023 China; ^2^ University of Chinese Academy of Sciences Beijing 100049 China

**Keywords:** 1, 3‐enyne, coumarin, dual‐ligand, Rh catalysis, skeleton‐divergence

## Abstract

Controlling regio‐ and chemo‐selectivity in transition‐metal‐catalyzed reactions involving coupling reagents with multiple reactive sites remains a significant challenge. In this study, a dual‐ligand strategy is introduced to orthogonally regulate both nucleophilic and electrophilic sites in the rhodium‐catalyzed sequential hydrofunctionalization of valylene. Leveraging the synergistic effects of bidentate and monodentate phosphine ligands, cyclic prenylation of 4‐hydroxycoumarins is achieved with outstanding regio‐ and chemo‐selectivity under basic conditions. Conversely, structurally reversed prenylation is selectively obtained using a dppb (1,4‐bis(diphenylphosphino)butane)/DME (1,2‐dimethoxyethane) ligand combination under acidic conditions. This efficient and versatile protocol is also applicable to pyrazol‐5‐one substrates, yielding high‐value dihydropyrano[2,3‐c]pyrazole analogs. Mechanistic studies suggest that the cyclic prenylation proceeds via C3‐ or O‐propargylation, followed by Rh‐ or acid‐promoted intermolecular annulation. It is hoped that this strategy will provide valuable insights for addressing selectivity challenges in transition‐metal catalysis and inspire further developments in this field.

## Graphical abstract

A remarkable dual‐ligand strategy is employed in Rh‐catalyzed sequential coupling reaction of dinucleophiles and 1,3‐enynes with high regio‐ and chemo‐selectivity.

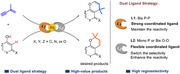

## 1 Introduction

**Scheme 1:**
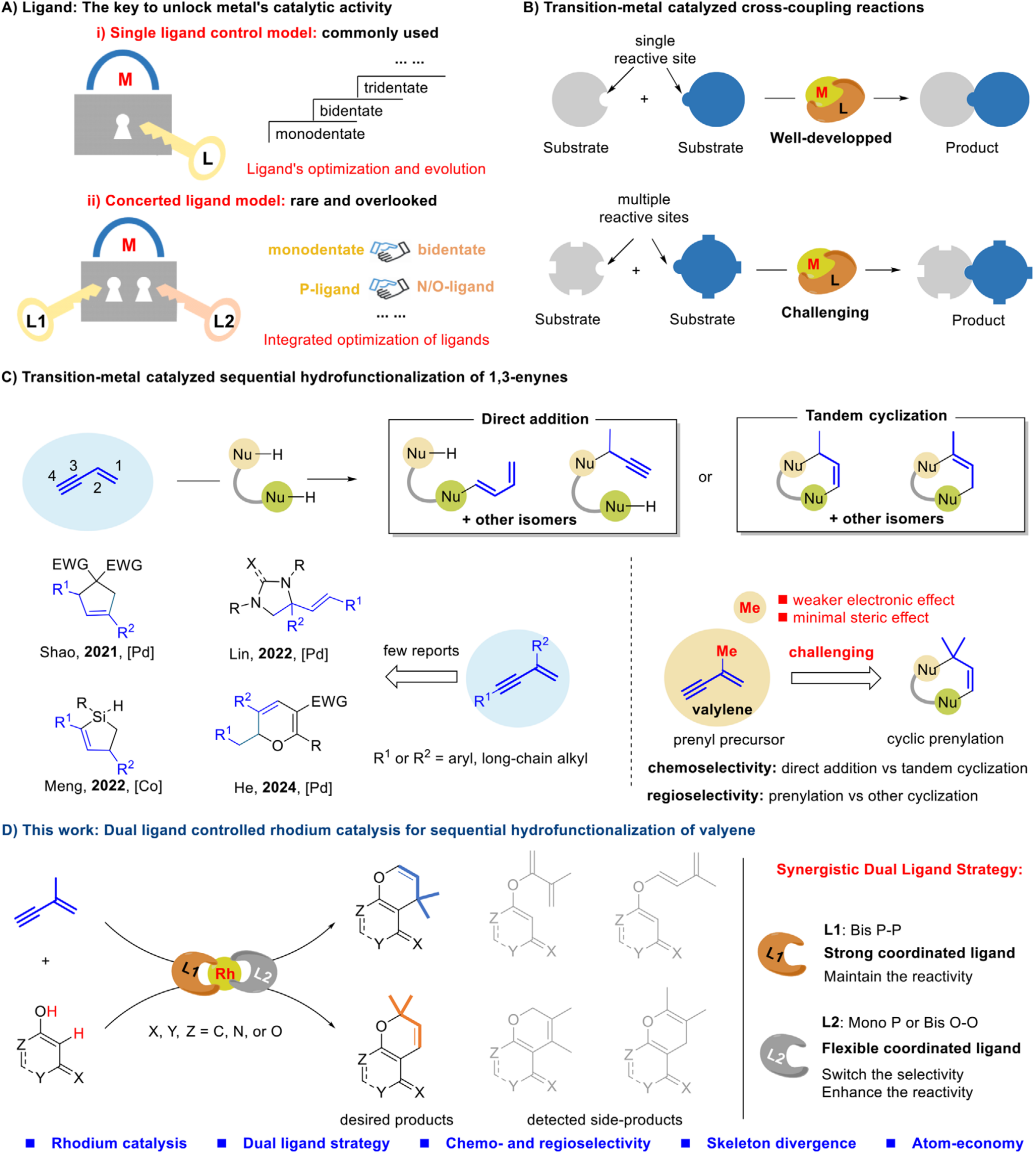
Transition‐metal catalyzed sequential hydrofunctionalization/cyclization of 1,3‐enynes.

As one of the most significant milestones in modern organic chemistry, the emergence and development of transition‐metal catalysis have profoundly advanced the technological evolution of organic synthesis.^[^ ^1^ ^]^ It is well‐established that in these reactions, ligands usually play a pivotal role in unlocking the catalytic activity of metals.^[^ ^2^ ^]^ However, the traditional “key and lock” model has often been constrained by the mode of “one key opens one lock”—in most cases, researchers tend to optimize transition‐metal catalysis through single‐ligand optimization (**Scheme** **1**A).^[^ ^3^ ^]^ To achieve superior catalytic performance, extensive efforts have been devoted to the design and modification of ligands, ranging from mono‐ to tridentate ligands and beyond. Yet, just as not all locks only require a single key, the screening of dual ligands, which has historically been underutilized, may offer new opportunities to unlock unprecedented selectivities.^[^ ^4^ ^]^

On the other hand, transition metal‐catalyzed cross‐coupling reactions have received continuous attention and emerged as a robust and versatile tool to forge C─C or C─X bonds.^[^ ^5^ ^]^ Although these reactions are well‐developed for substrates with single reactive sites, controlling regio‐ or chemo‐selectivity in reactions involving coupling reagents with multiple reactive sites remains a formidable challenge (Scheme 1B).^[^ ^6^ ^]^ For the latter, more sophisticated electronic and geometric modulation of the metal catalyst is required. Consequently, the synergistic use of two entirely distinct ligands may provide an efficient and rapid approach for the evaluation of metal catalysis by leveraging their inherently different electronic and steric properties.^[^ ^7^ ^]^

1,3‐Enynes are a class of readily accessible and versatile compounds characterized by the presence of two conjugated C═C and C≡C bonds. Their high degree of unsaturation and multiple reactive sites make them attractive precursors for maximizing molecular complexity.^[^ ^8^ ^]^ Although significant efforts have been devoted to their direct addition reactions in recent years, the sequential hydrofunctionalization/cyclization of 1,3‐enynes with dual reaction sites remains underdeveloped. This is primarily due to the challenges associated with controlling regio‐ and chemo‐selectivity, which arise from the competition between nucleophilic and electrophilic sites (Scheme 1C). Recently, leveraging aryl or long‐chain alkyl substituents, the Shao, Meng, Lin, and He groups have successfully achieved good selectivity in the sequential hydrofunctionalization/cyclization of 1,3‐enynes under Co or Pd catalysis.^[^ ^9^ ^]^ Valylene (2‐methyl‐1‐butene‐3‐yne), the smallest substituted 1,3‐enyne, is a fascinating organic building block for introducing biologically relevant prenyl groups. However, due to the weak electronic effects and minimal steric influence of the methyl group, controlling the selectivity of valylene in sequential hydrofunctionalization/cyclization poses a significant challenge (Scheme 1C). This necessitates the development of unconventional methods for selective control. Building on our ongoing research into selective prenylation reactions,^[^ ^10^ ^]^ we herein report a cooperative ligand model for the hydrofunctionalization/cyclization of valylene. By integrating a strongly coordinating bis P‐P ligand with a flexibly coordinating mono‐P or bis O─O ligand, we achieved regio‐switchable cyclic prenylation under rhodium catalysis with good selectivity and excellent atom economy (Scheme 1D).

## 2 Results and Discussion

**Scheme 2:**
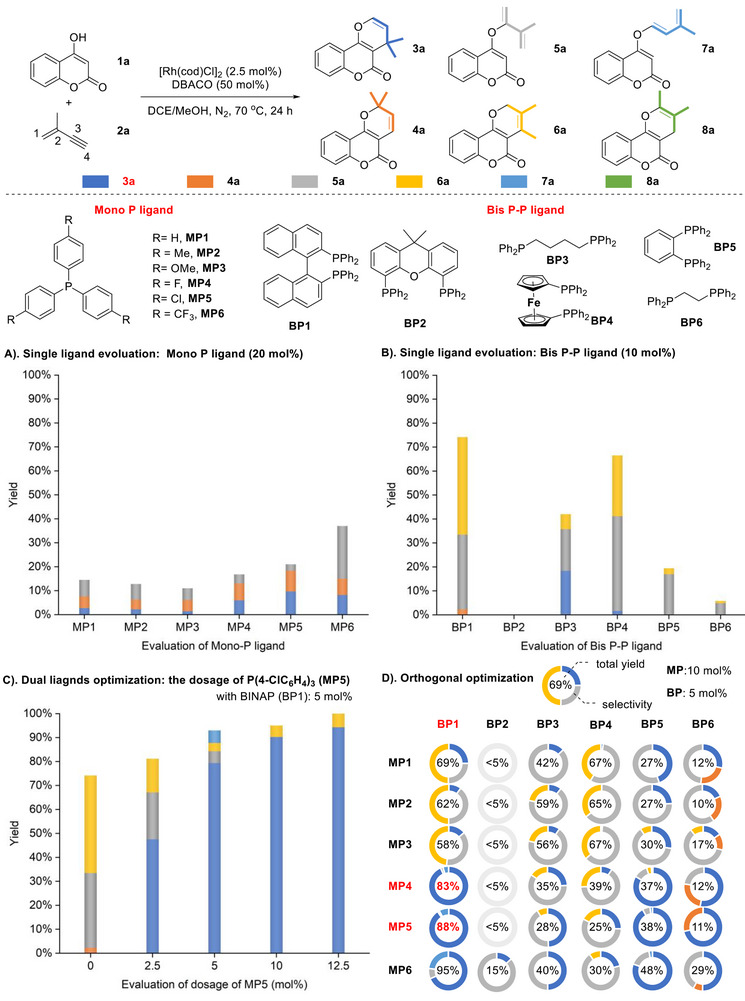
Dual‐ligand optimization for sequential 2,4‐hydroalkenoxylation of valylene with 4‐hydroxycoumarin.

Prenylated pyranocoumarins are an important class of privileged motifs found in numerous natural products, biologically active molecules, and drugs.^[^ ^11^ ^]^ Therefore, 4‐hydroxycoumarin (**1a**) and valylene (**2a**) were initially selected as model substrates under rhodium catalysis (**Scheme** **2**). Using [Rh(cod)Cl]_2_ as the catalyst precursor and DABCO (1,4‐diazabicyclo[2.2.2]octane) as the base, monodentate phosphine ligands were first evaluated. However, these reactions exhibited low reactivity, yielding a mixture of regio‐ and chemo‐isomers (**3a**, **4a**, and **5a**) (Scheme 2A). When bidentate phosphine ligands were tested, a new cyclic isomer (**6a**) and an alkenylation isomer (**7a**) were observed. Although BINAP (**BP1**) and dppf (**BP4**) delivered good total yields, the regio‐ and chemo‐selectivity remained unsatisfactory (Scheme 2B). Given that rhodium has an additional coordination site compared with its palladium or nickel analogs, we hypothesized that combining a bidentate ligand with a monodentate ligand might influence the reaction outcomes. Consequently, we investigated the reaction performance by varying the loading of the monodentate ligand (4‐ClC_6_H_4_)_3_P (**MP5**) under [Rh(cod)Cl]_2_/BINAP catalysis (Scheme 2C). Surprisingly, the reaction selectivity shifted dramatically upon the addition of (4‐ClC_6_H_4_)_3_P, and **3a** was obtained in good yield and selectivity when the loading of (4‐ClC_6_H_4_)_3_P was increased to 10 mol%. To further explore the synergistic effect between bidentate and monodentate phosphine ligands, an orthogonal optimization was conducted (Scheme 2D). The reactions exhibited significant differences depending on the ligand combinations. Among all tested ligands, a higher reactivity was observed when BINAP was used, while electron‐deficient monodentate phosphine ligands favored the formation of **3a**. In terms of yield and selectivity, the combination of BINAP (**BP1**) with (4‐FC_6_H_4_)_3_P (**MP4**) or (4‐ClC_6_H_4_)_3_P (**MP5**) displayed the best performance.

**Scheme 3:**
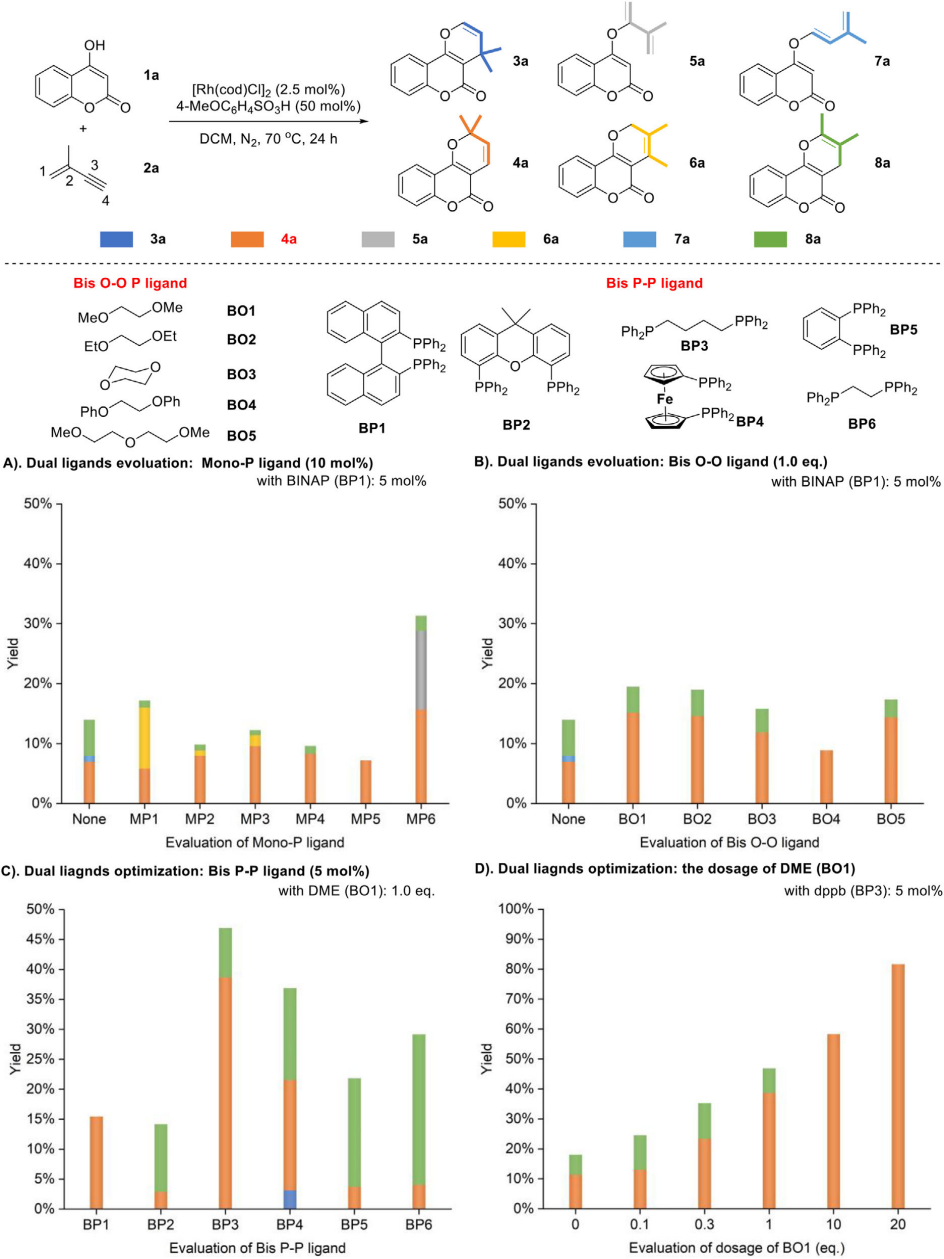
Dual‐ligand optimization for sequential 4,2‐hydroalkenoxylation of valylene with 4‐hydroxycoumarin.

When the reaction was carried out using 4‐MeOC₆H_4_SO_3_H rather than DABCO, the reaction furnished a mixture of **4a**, **7a,** and **8a** in low yields with BINAP as ligand (**Scheme** **3**A). We then wondered whether the cooperative ligand strategy could also be applied under the acidic conditions. Therefore, a series of mono‐P ligands were individually evaluated with BINAP as cooperative partner. Unfortunately, all these reactions exhibited low conversion or unsatisfactory selectivity (Scheme 3A). Unexpectedly, when bis O‐O ligands were used instead of mono P ligands as the second ligand partner, an improvement in both reactivity and selectivity was observed (Scheme 3B). Encouraged by these results, bis P‐P ligands were further tested in combination with DME (1,2‐dimethoxyethane). Among them dppb (**BP3**) demonstrated better performance in terms of reactivity (Scheme 3C). Given the weak coordination ability of bis O‐O ligands, the dosages of DME were evaluated (Scheme 3D). As the amount of DME increased, the selectivity and conversion rate to **4a** improved consistently. Ultimately, **4a** was obtained in 81% yield with cheap and easily available DME as co‐solvent (20 equivalents).

**Scheme 4:**
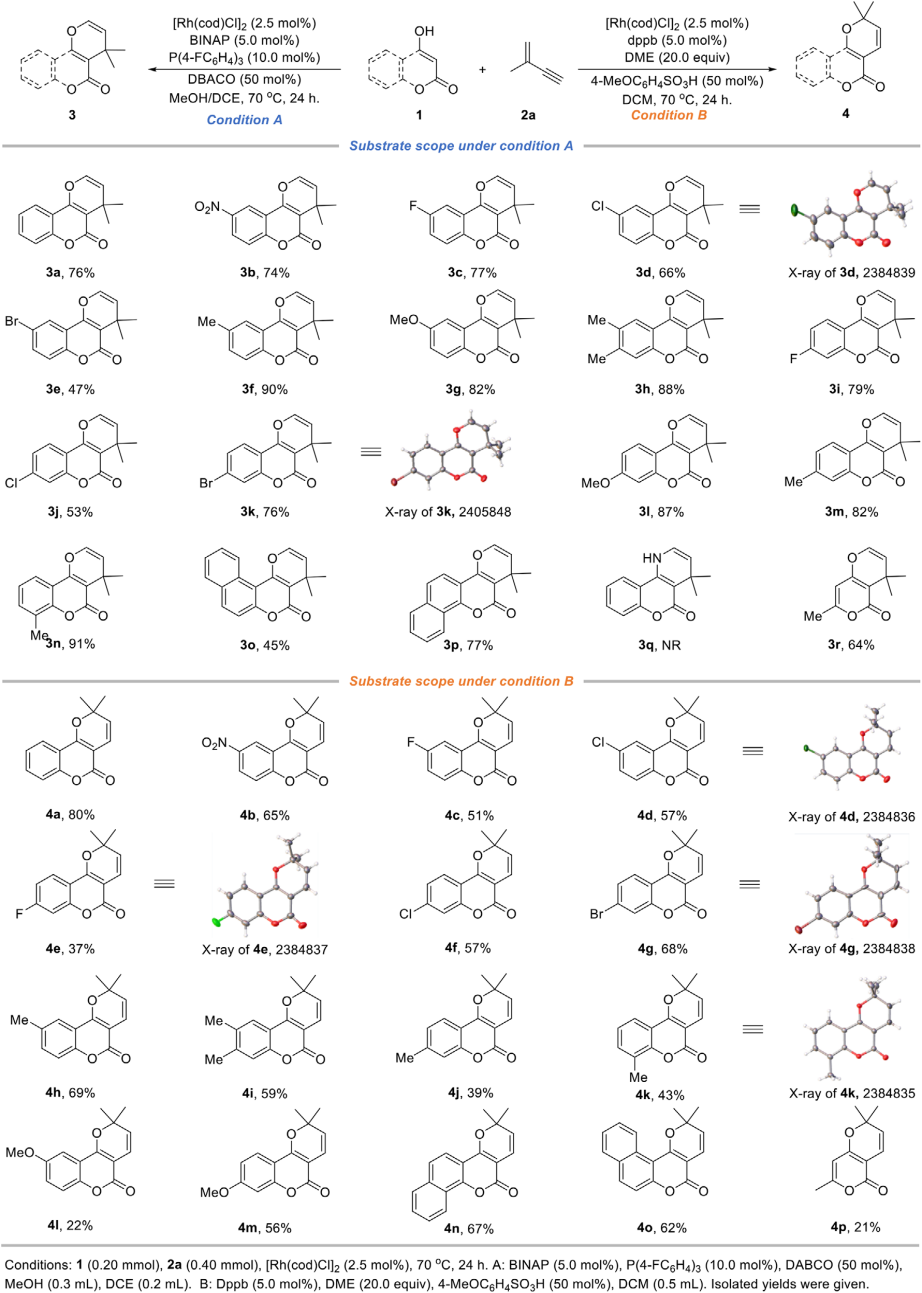
The substrate scope for regiodivergent hydroalkenoxylation using dual‐ligand strategy.

With the optimal conditions established, we next investigated the generality of 4‐hydroxycoumarins to demonstrate the feasibility and practicality of this protocol (**Scheme** **4**). Using DABCO as the base, a diverse range of 4‐hydroxycoumarins underwent sequential hydrofunctionalization, delivering high yields and excellent selectivity (Condition A). Under the optimal conditions, the desired product **3a** was isolated in 76% yield from the reaction of 4‐hydroxycoumarin **1a** and valylene (**2a**). The robust dual‐ligand system exhibited remarkable compatibility with a variety of functional groups, including electron‐withdrawing groups (‐NO_2_, ‐F, ‐Cl, and ‐Br), affording products **3b‐e** in 47–77% yields. Satisfactorily, the introduction of substituents at the 7‐position of the phenyl ring (**3i‐k**) also provided yields of 53–79%. 4‐Hydroxycoumarins bearing electron‐donating groups (‐Me, ‐OMe) were transformed into the desired products (**3f‐h** and **3l‐n**) in 82–91% yields, demonstrating high reactivity. Additionally, benzocoumarins (**3o‐p**) were well‐tolerated under the standard conditions. Although 4‐aminocoumarin was not compatible with the current conditions (**3q**), the annulation of hydroxypyranone **1r** with valylene proceeded smoothly, yielding **3r** in 64% yield. The structures of **3d**, **3k**, and **3n** were confirmed by single‐crystal X‐ray crystallography.

Subsequently, the substrate scope was explored by integrating dppb with DME as dual ligands (Scheme 4, Condition B). Under the optimized conditions, 4‐hydroxycoumarin **1a** was converted to **4a** in 80% yield. A variety of electron‐withdrawing groups (‐NO_2_, ‐F, ‐Cl, and ‐Br) were compatible with this protocol, giving the corresponding products **4b–g** in 37%–68% yields. The structures of **4d**, **4e**, and **4g** were further confirmed by single‐crystal X‐ray crystallography. Additionally, electron‐donating substituents, such as ‐Me and ‐OMe groups, were all amenable to the current reaction conditions, generating **4h‐k** and **4l‐m** in decent yields. Similarly, the structure of **4k** was also confirmed by single‐crystal X‐ray crystallography. Remarkably, benzocoumarins exhibited good reactivity, affording **4n‐o** in 62%–67% yields. However, the annulation of hydroxypyranone with valylene was comparatively sluggish, delivering **4p** in 21% yield. The low yields of **4l** and **4p** were mainly attributed to the poor solubility of their corresponding substrates (**1l** and **1p**).

**Scheme 5:**
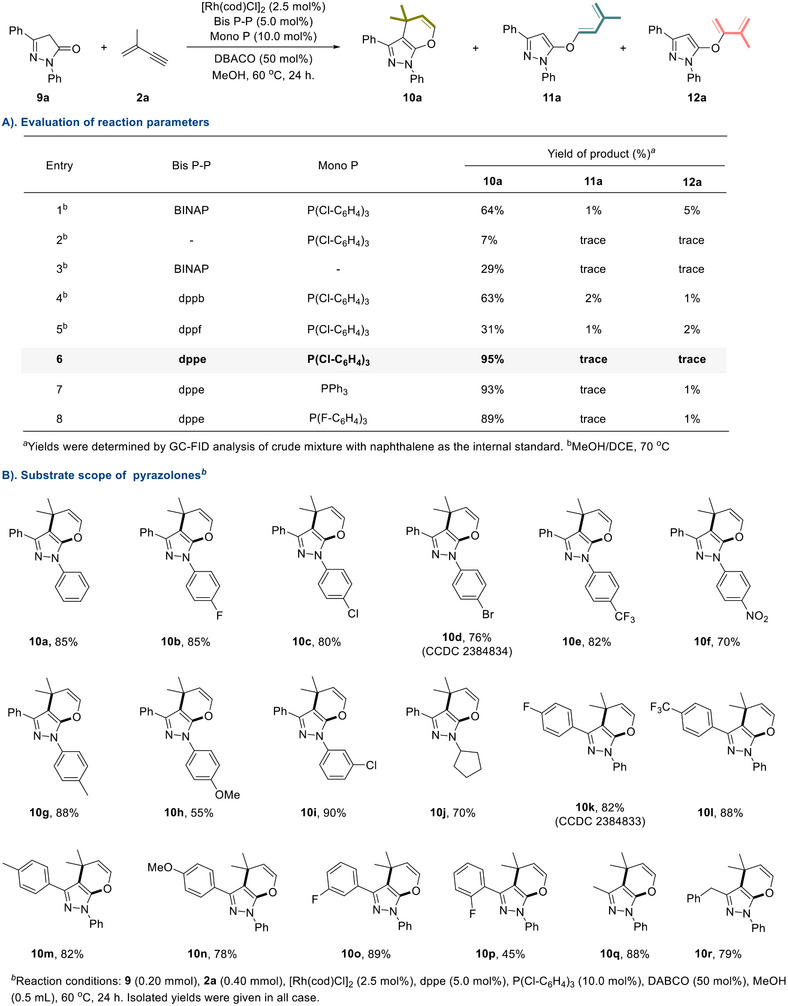
Dual‐ligand strategy in Rh‐catalyzed sequential 2,4‐hydroalkenoxylation of valylene with pyrazol‐5‐ones.

Dihydropyrano[2,3‐c]pyrazoles are a class of fused pyran and pyrazole rings with significant application in medicinal chemistry due to their broad spectrum of biological activities, including antimicrobial, anticancer, anti‐inflammatory, and antiviral properties.^[^ ^12^ ^]^ Therefore, we investigated whether the current dual‐ligand regulated rhodium catalysis system could also be applied to the annulation of pyrazol‐5‐ones with valylene to afford dihydropyrano[2,3‐c]pyrazole analogs. After carefully evaluating a series of reaction parameters (For details see Tables S9‐S11 in the Supporting Information), we were delighted to find that the reaction using pyrazol‐5‐one **9a** as the model substrate successfully delivered the annulated product **10a** in 64% yield with BINAP and P(4‐ClC₆H_4_)_3_ as cooperative ligands, accompanied by a small amount of diene side products **11a** and **12a** (**Scheme** **5**A, entry 1). Control experiments revealed that the yield of **10a** decreased dramatically when using P(4‐ClC₆H_4_)_3_ or BINAP separately, indicating that dual ligands were indispensable for maintaining the reactivity (Scheme 5A, entries 1 and 2). After further evaluation of different ligand combinations, the yield of **10a** was increased to 95% with excellent chemoselectivity when dppe and P(4‐ClC₆H_4_)_3_ were selected as ligand combo (Scheme 5A, entries 3–8).

With the optimal conditions established, we were able to investigate the scope of pyrazol‐5‐ones in sequential coupling with valylene. As shown in Scheme 5B, employing dppe/(4‐ClC_6_H_4_)_3_P as dual‐ligand and DABCO as the base in the presence of [Rh(cod)Cl]_2_, dihydropyrano[2,3‐c]pyrazole **10a** could be successfully isolated in 85% yield. A variety of substrates with substituents on the phenyl ring at the nitrogen atom, whether electron‐deficient groups (‐F, ‐Cl, ‐Br, ‐CF_3_, and ‐NO_2_) or electron‐rich groups (‐Me and ‐OMe), all provided acceptable yields (**10b‐i**). Notably, substrate **10j** bearing a cyclopentyl group attached to the nitrogen atom was also smoothly converted in 77% yield. As for substrates **9k‐o**, the electronic properties of substituents at the *meta‐* or *para‐*position of the phenyl ring (R^2^) had a slight influence on the reaction outcomes, delivering the desired products in 79‐89% yields. Due to steric hindrance, a decrease in yield was observed when *ortho*‐substituted pyrazol‐5‐one was employed (**10p**). Satisfyingly, alkyl and benzyl groups (‐Me, ‐Bn) substituted pyrazol‐5‐ones (**9q‐r**) were also tolerated in this protocol. The structures of **10d** (CCDC: 2 384 834) and **10k** (CCDC: 2 384 833) were further confirmed by single‐crystal X‐ray crystallography.

## 3 Mechanistic Studies

**Scheme 6:**
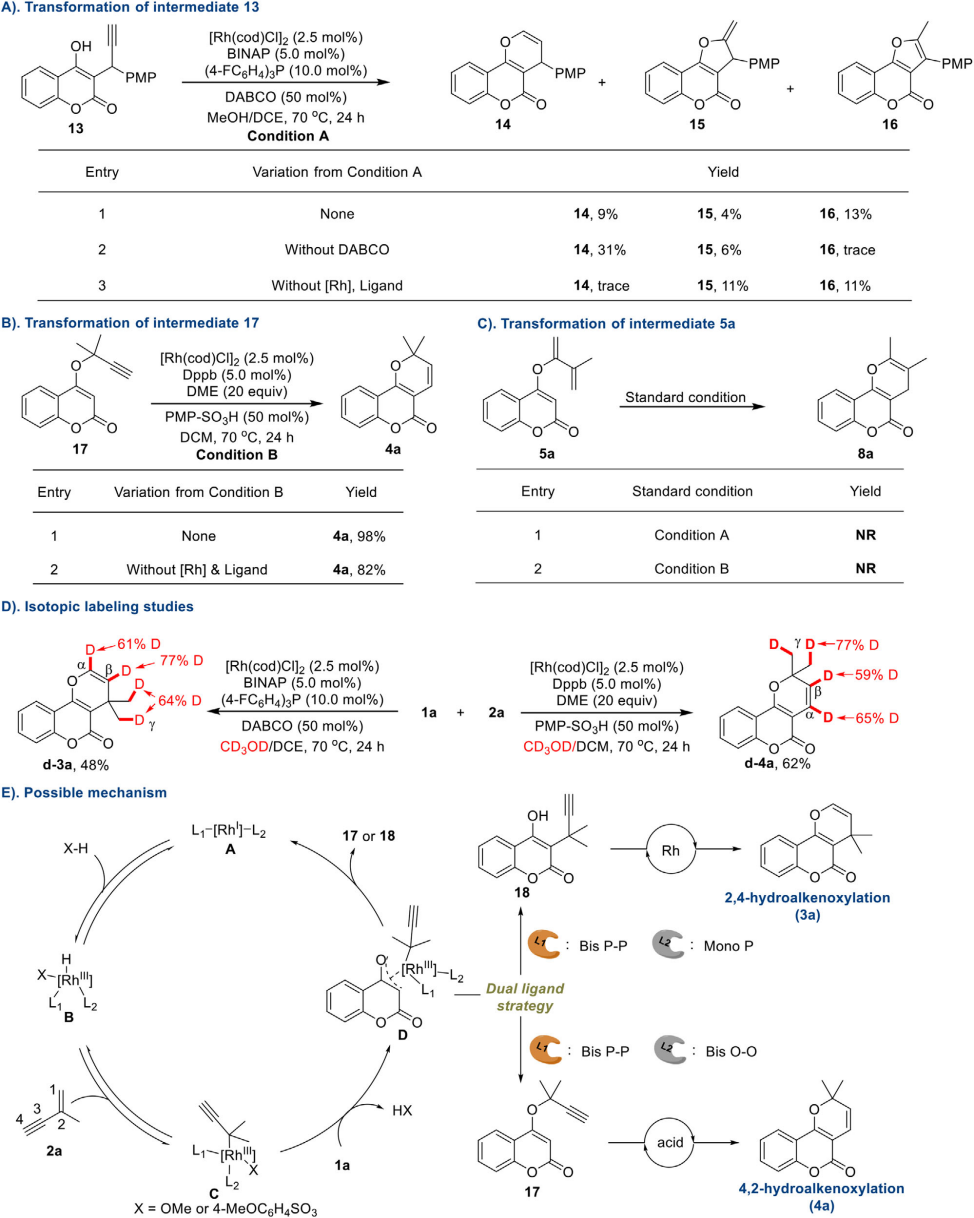
Mechanistic studies and possible mechanism.

To gain mechanistic insights into this sequential hydrofunctionalization reaction, we carried out some control experiments. Considering that 4‐hydroxycoumarin contains two reactive nucleophilic sites, the C3‐propargylated 4‐hydroxycoumarin **13** was first prepared to probe potential intermediates (**Scheme** **6**A). To our delight, the intramolecular cyclization indeed occurred to afford **14** in 9% yield under standard condition A, accompanied by furocoumarin‐type side products **15** and **16** (Scheme 6A, entry 1). Compared with the actual intermediate, the low yield and selectivity of this simulated intermediate **13** may be attributed to the absence of the Thorpe–Ingold effect. Control experiments showed that the rhodium catalyst was necessary for this transformation (Scheme 6A, entries 2 and 3). Next, O‐propargyl coumarin **17** was also synthesized and subjected to acidic conditions (Scheme 6B). As expected, cyclic prenylation occurred smoothly under standard condition B to deliver product **4a** in 98% yield (Scheme 6B, entry 1). However, in contrast to the basic condition, control experiments indicated that this ring‐closure process could be promoted by acid alone (Scheme 6B, entry 2). In addition to the propargylated intermediate, diene side‐product **5a** was also examined (Scheme 6C). The results showed that no annulated product could be detected under either condition A or condition B. Next, an isotopic labeling study was performed (Scheme 6D). When the reaction of 4‐hydroxycoumarin **1a** with valylene **2a** was conducted under standard condition A or B, respectively, the deuterium was found to be scrambled across the prenyl unit of products **3a** and **4a** in the presence of CD_3_OD. These results indicate that a reversible Rh‐H addition to valylene may exist.

Based on the above mechanistic investigations, a plausible mechanism is proposed (Scheme 6E). First, the oxidative addition of acid (or alcohol) and the Rh(I) complex affords the Rh(III)‐hydride **B**, followed by the migratory insertion with valylene **2a** to deliver the propargyl‐Rh complex **C**. Then, nucleophilic substitution between **C** and 4‐hydroxycoumarin **1a** gives complex **D**. With bidentate (BINAP) and monodentate phosphine ligand (P(4‐ClC_6_H_4_)_3_) as cooperative ligands, C–C bond may be first constructed through a reductive elimination of complex **D**, leading to C3‐propargylated intermediate **18**. Followed by Rh‐promoted intramolecular cyclization, 2,4‐hydroalkenoxylated product **3a** is delivered eventually. On the other hand, when reaction is performed with dppb and DME as ligand combination, O‐propargylated intermediate **17** may be produced preferably. Finally, the reaction proceeds through an acid‐catalyzed annulation to generate 4,2‐hydroalkenoxylated product **4a**.

## 4 Conclusion

In conclusion, we have developed a dual‐ligand strategy to control chemo‐ and regioselectivity in Rh‐catalyzed sequential hydrofunctionalization of valylene. Cooperative use of bidentate (BINAP) and monodentate phosphine ligand (P(4‐ClC_6_H_4_)_3_) under basic conditions enabled 2,4‐hydroalkenoxylation of valylene with 4‐hydroxycoumarins, while dppb/DME under acidic conditions promoted 4,2‐hydroalkenoxylation. This protocol also demonstrated broad substrate compatibility, accommodating diverse 4‐hydroxycoumarins and pyrazol‐5‐ones to deliver high‐value dihydropyrano[2,3‐c]pyrazole derivatives with excellent functional group tolerance. Mechanistic studies revealed that the reaction initially proceeds through a regioselective hydrofunctionalization of alkene bond to form C3‐ or O‐propargylated intermediates, followed by an intramolecular cyclization reaction, which leads to the regiodivergent annulation process. The current work highlights cooperative ligand effects for orthogonal selectivity, offering a generalizable platform for addressing selectivity challenges in reactions involving substrates with multiple reactive sites.

## Conflict of Interest

The authors declare no competing interests.

## Supporting information



Supporting Information

Supplemental Data

## Data Availability

The data that support the findings of this study are available in the supplementary material of this article.;
