# Supplementary material for: Dual‐Ligand Strategy in Rh‐Catalyzed Sequential Hydrofunctionalization of Valylene
Source: Adv Sci (Weinh). 2025 Jul 30;12(40):e11331. doi: 10.1002/advs.202511331 (PMC12561339; doi:10.1002/advs.202511331)

Supporting Information for:

Dual-Ligand Strategy in Rh-Catalyzed Sequential Hydrofunctionalization of Valylene

Yong-Kang Mei[1,2], Su-Yang Xu[1,2], Zhi-Hui Wang[1,2], Ding-Wei Ji*[,1], Qing-An Chen*[,1,2]

[1]Dalian Institute of Chemical Physics, Chinese Academy of Sciences, Dalian 116023, China.

[2]University of Chinese Academy of Sciences, Beijing 100049, China.

*Corresponding author. E-mail: dingweiji@dicp.ac.cn, qachen@dicp.ac.cn

Table of Contents

1. General experimental details....S2

2. Procedure for the synthesis of substrates....S2

  2.1. Procedure for the synthesis of 4-Hydroxycoumarins (1)....S2

  2.2. Procedure for the synthesis of pyrazol-5-ones (9)....S3

3. Screening of reaction conditions....S3

  3.1 Optimization of the reaction condition for 3a....S3

  3.2 Optimization of the reaction condition for 4a....S6

  3.3 Optimization of the reaction condition for the coupling of 9a and 2a....S8

4. General procedure for Hydrofunctionalization of 1,3-Enynes....S10

  4.1. General procedure A: Rh-catalyzed annulation of 1,3-Enynes with 4-Hydroxycoumarins under basic condition....S10

  4.2. General procedure B: Rh-catalyzed annulation of 1,3-Enynes with 4-Hydroxycoumarins under acidic condition....S14

  4.3. General procedure C: Rh-catalyzed annulation of 1,3-Enynes with pyrazol-5-ones....S21

  5. Mechanistic studies....S27

6. Evaluation of alternative dinucleophilic substrates under standard conditions....S32

7. Crystallographic data....S33

8. References....S41

9. Copies of NMR spectra....S42

1. General experimental details.

Commercially available reagents were used without further purification. Solvents were treated prior to use according to the standard methods. Unless otherwise stated, all reactions were conducted under inert atmosphere using standard Schlenk techniques or in a nitrogen-filled glove-box. ^{1}H NMR and ^{13}C NMR spectra were recorded at room temperature in $CDCl_3$ on 400 MHz or 700 MHz instrument with tetramethylsilane (TMS) as internal standard. Data are reported as follows: chemical shift in ppm (δ), multiplicity (s = singlet, d = doublet, t = triplet, q = quartet, brs = broad singlet, m = multiplet), coupling constant (Hz), and integration. Flash column chromatography was performed on silica gel (200-300 mesh). All reactions were monitored by TLC, GC-FID, GC-MS or NMR analysis. HRMS data was obtained with Micromass HPLC-Q-TOF mass spectrometer (ESI) or Agilent 6540 Accurate-MS spectrometer (Q-TOF).

2. Procedure for the synthesis of substrates.

2.1. Procedure for the synthesis of 4-Hydroxycoumarins (1)

4-Hydroxycoumarins **1a**-**1b**, **1e**, **1g**, **1m**, and **1q** are commercially available. Other substrates were synthesized according to the following procedure.

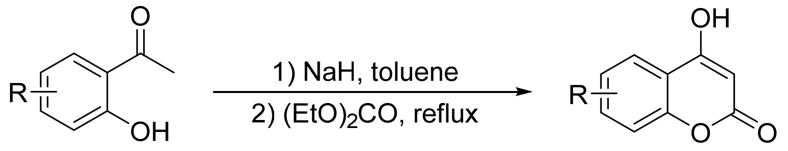

According to the previous reported procedure,[1] under nitrogen atmosphere, a suspension of NaH (50 mmol, 5.0 eq.) in toluene (20 mL) was cooled in an ice bath. To the suspension was added 1-(2-hydroxyphenyl)ethan-1-one derivative (10 mmol, 1.0 eq.) in one portion and the resulting mixture was stirred for 10 min. Next, the mixture was allowed to warm to room temperature and stirred for 30 min. Then, diethyl carbonate (15 mmol, 1.5 eq.) was added dropwise to the mixture at room temperature. The reaction was heated to reflux and stirred overnight. After the reaction completed, the solution was cooled to room temperature and the precipitate was collected, washed sequentially with 1 M HCl solution and water to give the crude product. The crude product was triturated with small amount of EtOH and recrystallized from EtOH to give corresponding pure product.

2.2. Procedure for the synthesis of pyrazol-5-ones (9)

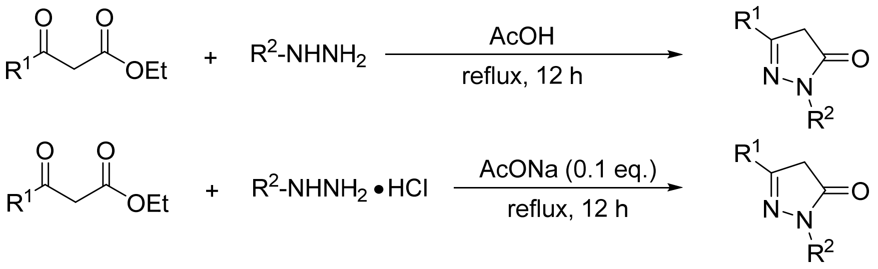

According to the previously reported procedure,[2] to a solution of β-ketoester in acetic acid (1 M) was added substituted phenylhydrazine (1.2 eq.) (For phenylhydrazine hydrochloride salts, 0.1 equiv. of NaOAc was added as a base). The reaction mixture was refluxed for 12 h. After completed, the solution was cooled to room temperature, and the solvent was removed *in vacuo*. The resulting crude product was triturated with a minimal amount of EtOH and recrystallized from EtOH to give pure pyrazol-5-ones.

3. Screening of reaction conditions.

3.1 Optimization of the reaction condition for 3a.

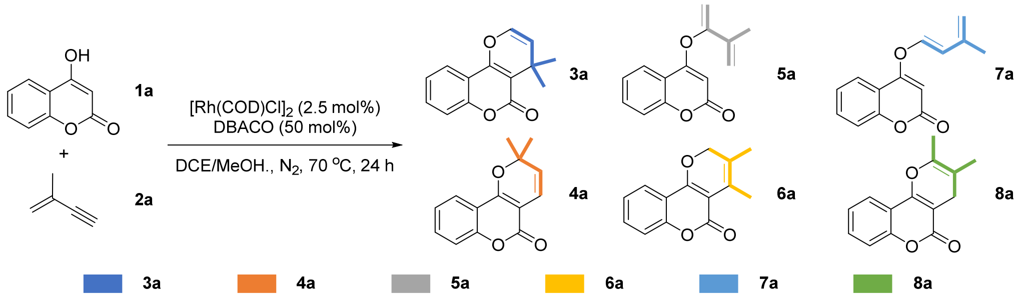

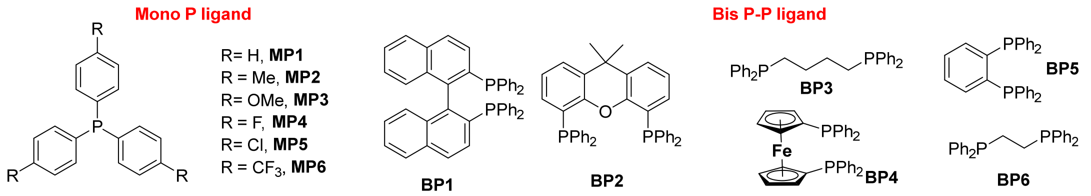

Table S1: The evaluation of Mono-P ligand

Entry	Mono-P ligand	[a]Yield (%)					
		3a	4a	5a	6a	7a	8a
1	**MP1**	3%	5%	7%	trace	trace	trace
2	**MP2**	2%	4%	7%	trace	trace	trace
3	**MP3**	1%	5%	5%	trace	trace	trace
4	**MP4**	6%	7%	4%	trace	trace	trace
5	**MP5**	10%	9%	3%	trace	trace	trace
6	**MP6**	8%	7%	22%	trace	trace	trace

Reaction condition: **1a** (0.20 mmol), **2a** (0.40 mmol), $[Rh(cod)Cl]_2$ (2.5 mol%), Mono-P ligand (20 mol%), DABCO (50 mol%), DCE (0.2 mL), MeOH (0.3 mL), 70 ºC, 24 h. [a]Yields were determined by GC-Fid analysis of crude mixture with 1,3,5-trimethoxybenzene as the internal standard.

Table S2: The evaluation of Bis P-P ligand

Entry	Bis P-P ligand	[a]Yield (%)					
		3a	4a	5a	6a	7a	8a
1	**BP1**	trace	2%	31%	41%	trace	trace
2	**BP2**	trace	trace	trace	trace	trace	trace
3	**BP3**	18%	trace	17%	6%	trace	trace
4	**BP4**	2%	trace	40%	25%	trace	trace
5	**BP5**	trace	trace	17%	2%	trace	trace
6	**BP6**	trace	trace	5%	1%	trace	trace

Reaction condition: **1a** (0.20 mmol), **2a** (0.40 mmol), $[Rh(cod)Cl]_2$ (2.5 mol%), Bis P-P ligand (10 mol%), DABCO (50 mol%), DCE (0.2 mL), MeOH (0.3 mL), 70 ºC, 24 h. [a]Yields were determined by GC-Fid analysis of crude mixture with 1,3,5-trimethoxybenzene as the internal standard.

Table S3: The evaluation of $P(4\text{-}ClC_6H_4)_3$ (MP6) amount with BINAP (5 mol%)

Entry	MP6 (x mol%)	[a]Yield (%)					
		3a	4a	5a	6a	7a	8a
1	None	trace	2%	31%	41%	trace	trace
2	2.5	48%	trace	20%	14%	trace	trace
3	5	79%	trace	5%	3%	5%	trace
4	7.5	89%	trace	trace	trace	7%	trace
5	10	90%	trace	trace	5%	trace	trace
6	12.5	94%	trace	trace	9%	trace	trace

Reaction condition: **1a** (0.20 mmol), **2a** (0.40 mmol), $[Rh(cod)Cl]_2$ (2.5 mol%), $P(4\text{-}ClC_6H_4)_3$ (x mol%), BINAP (5 mol%), DABCO (50 mol%), DCE (0.2 mL), MeOH (0.3 mL), 70 ºC, 24 h. [a]Yields were determined by GC-Fid analysis of crude mixture with 1,3,5-trimethoxybenzene as the internal standard.

Table S4: Orthogonal optimization of mono- and bidendate phosphine ligands

Entry	Mono-P ligand	Bis P-P ligand	[a]Yield (%)					
			3a	4a	5a	6a	7a	8a
1	**MP1**	**BP1**	17%	trace	18%	35%	trace	trace
2		**BP2**	1%	trace	trace	trace	trace	trace
3		**BP3**	5%	trace	37%	trace	trace	trace
4		**BP4**	1%	trace	38%	28%	trace	trace
5		**BP5**	12%	trace	15%	trace	trace	trace
6		**BP6**	4%	3%	8%	trace	trace	trace
7	**MP2**	**BP1**	6%	trace	25%	31%	trace	trace
8		**BP2**	trace	trace	2%	trace	trace	trace
9		**BP3**	6%	trace	37%	12%	trace	trace

10		**BP4**	trace	trace	44%	25%	trace	trace
11		**BP5**	6%	trace	21%	trace	trace	trace
12		**BP6**	2%	2%	8%	trace	trace	trace
13		**BP1**	8%	trace	22%	28%	trace	trace
14		**BP2**	trace	trace	trace	trace	trace	trace
15	**MP3**	**BP3**	5%	trace	39%	12%	trace	trace
16		**BP4**	1%	trace	42%	24%	trace	trace
17		**BP5**	8%	trace	19%	3%	trace	trace
18		**BP6**	3%	2%	12%	2%	trace	trace
19		**BP1**	77%	trace	2%	trace	4%	trace
20		**BP2**	2%	trace	trace	trace	trace	trace
21	**MP4**	**BP3**	8%	trace	21%	6%	trace	trace
22		**BP4**	3%	trace	26%	10%	trace	trace
23		**BP5**	31%	trace	4%	1%	trace	trace
24		**BP6**	8%	3%	4%	trace	trace	trace
25		**BP1**	80%	trace	1%	trace	7%	trace
26		**BP2**	4%	trace	trace	trace	trace	trace
27	**MP5**	**BP3**	13%	trace	12%	2%	trace	trace
28		**BP4**	6%	trace	14%	4%	trace	trace
29		**BP5**	35%	trace	2%	trace	1%	trace
30		**BP6**	11%	4%	trace	trace	trace	trace
31		**BP1**	61%	trace	9%	trace	25%	trace
32		**BP2**	2%	trace	13%	trace	trace	trace
33	**MP6**	**BP3**	19%	trace	20%	trace	trace	trace
34		**BP4**	6%	trace	21%	3%	trace	trace
35		**BP5**	38%	trace	8%	trace	1%	trace
36		**BP6**	16%	2%	14%	trace	trace	trace

Reaction condition: **1a** (0.20 mmol), **2a** (0.40 mmol), $[Rh(cod)Cl]_2$ (2.5 mol%), Mono-P ligand (10 mol%), Bis P-P ligand (5 mol%), DABCO (50 mol%), DCE (0.2 mL), MeOH (0.3 mL), 70 ºC, 24 h. [a]Yields were determined by GC-Fid analysis of crude mixture with 1,3,5-trimethoxybenzene as the internal standard.

Control reaction (without base):

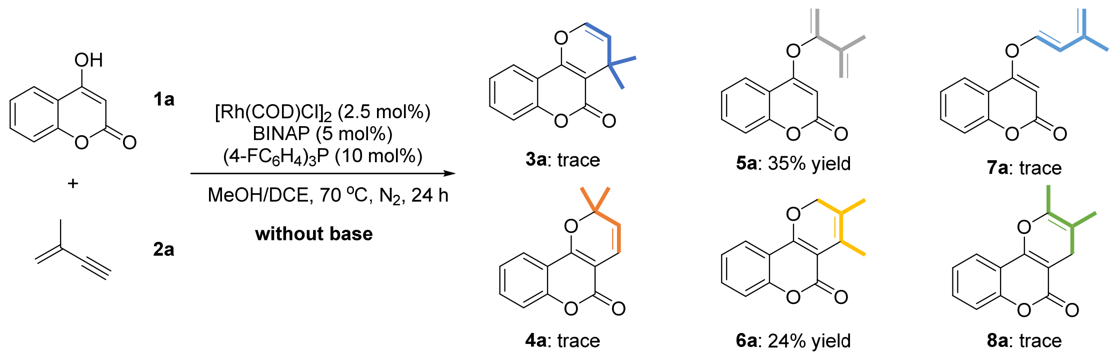

3.2 Optimization of the reaction condition for 4a.

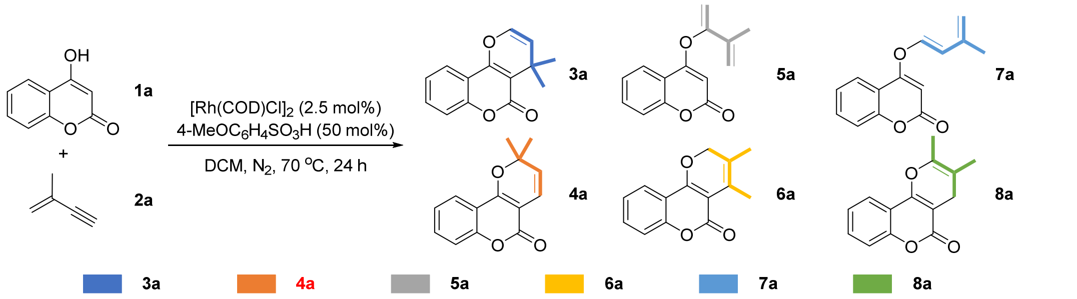

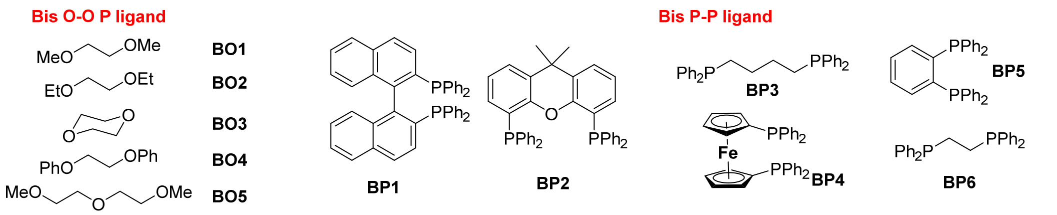

Table S5: The evaluation of Mono-P ligands (10 mol%) with BINAP (5 mol%)

Entry	**Mono-P ligand**	[a]Yield (%)					
		**3a**	**4a**	**5a**	**6a**	**7a**	**8a**
1	None	trace	7%	trace	trace	1%	6%
2	**MP1**	trace	6%	trace	10%	trace	1%
3	**MP2**	trace	8%	trace	1%	trace	1%
4	**MP3**	trace	10%	trace	2%	trace	1%
5	**MP4**	trace	8%	trace	trace	trace	1%
6	**MP5**	trace	7%	trace	trace	trace	trace
7	**MP6**	trace	16%	13%	trace	trace	3%

Reaction condition: **1a** (0.20 mmol), **2a** (0.40 mmol), $[Rh(cod)Cl]_2$ (2.5 mol%), Mono-P ligand (10 mol%), BINAP (5 mol%), $4\text{-}MeOC_6H_4SO_3H$ (50 mol%), DCM (0.5 mL), 70 °C, 24 h. [a]Yields were determined by GC-Fid analysis of crude mixture with 1,3,5-trimethoxybenzene as the internal standard.

Table S6: The evaluation of Bis O-O ligands with BINAP (5 mol%).

Entry	**Bis O-O ligand**	[a]Yield (%)					
		**3a**	**4a**	**5a**	**6a**	**7a**	**8a**
1	None	trace	7%	trace	trace	1%	6%
2	**BO1**	trace	15%	trace	trace	trace	4%
3	**BO2**	trace	15%	trace	trace	trace	4%
4	**BO3**	trace	12%	trace	trace	trace	4%
5	**BO4**	trace	9%	trace	trace	trace	trace
6	**BO5**	trace	14%	trace	trace	trace	3%

Reaction condition: **1a** (0.20 mmol), **2a** (0.40 mmol), $[Rh(cod)Cl]_2$ (2.5 mol%), Bis O-O ligand (1.0 equiv.), BINAP (5 mol%), $4\text{-}MeOC_6H_4SO_3H$ (50 mol%), DCM (0.5 mL), 70 °C, 24 h. [a]Yields were determined by GC-Fid analysis of crude mixture with 1,3,5-trimethoxybenzene as the internal standard.

Table S7: Evaluation of Bis P-P ligands with DME (1.0 equiv.)

Entry	Bis P-P ligand	[a]Yield (%)					
		**3a**	**4a**	**5a**	**6a**	**7a**	**8a**
1	**BP1**	trace	15%	trace	trace	trace	trace
2	**BP2**	trace	3%	trace	trace	trace	11%
3	**BP3**	trace	39%	trace	trace	trace	8%
4	**BP4**	3%	18%	trace	trace	trace	15%
5	**BP5**	trace	4%	trace	trace	trace	18%
6	**BP6**	trace	4%	trace	trace	trace	25%

Reaction condition: **1a** (0.20 mmol), **2a** (0.40 mmol), $[Rh(cod)Cl]_2$ (2.5 mol%), DME (0.2 mmol), bis P-P (5 mol%), $4\text{-}MeOC_6H_4SO_3H$ (50 mol%), DCM (0.5 mL), 70 ºC, 24 h. [a]Yields were determined by GC-Fid analysis of crude mixture with 1,3,5-trimethoxybenzene as the internal standard.

Table S8: The evaluation of DME amount with Dppb (5 mol%)

Entry	DME (x equiv.)	[a]Yield (%)					
		**3a**	**4a**	**5a**	**6a**	**7a**	**8a**
1	0	trace	12%	trace	trace	trace	4%
2	0.1	trace	13%	trace	trace	trace	12%
3	0.3	trace	23%	trace	trace	trace	12%
4	1.0	trace	39%	trace	trace	trace	8%
5	10	trace	58%	trace	trace	trace	trace
6	20	trace	82%	trace	trace	trace	trace

Reaction condition: **1a** (0.20 mmol), **2a** (0.40 mmol), $[Rh(cod)Cl]_2$ (2.5 mol%), DME (x equiv.), Dppb (5 mol%), $4\text{-}MeOC_6H_4SO_3H$ (50 mol%), DCM (0.5 mL), 70 ºC, 24 h. [a]Yields were determined by GC-Fid analysis of crude mixture with 1,3,5-trimethoxybenzene as the internal standard.

Control reaction (without acid):

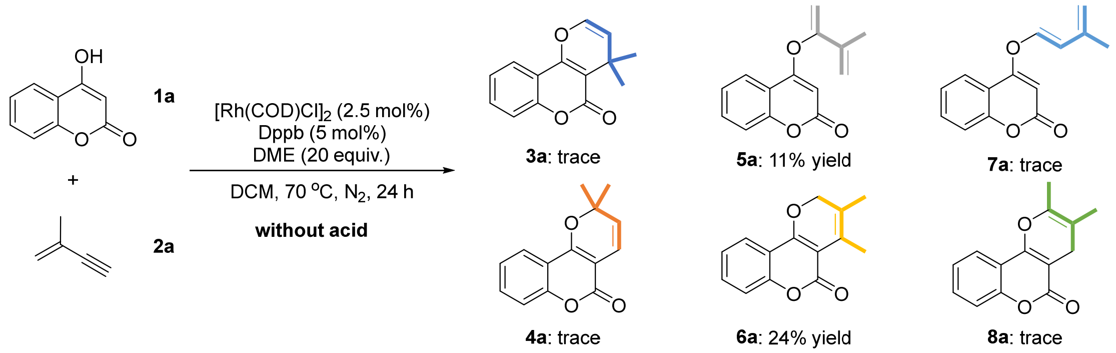

The evaluation of different mono P ligands combining with DME:

A) Evaluation of mono P in the presence of DME under basic condition

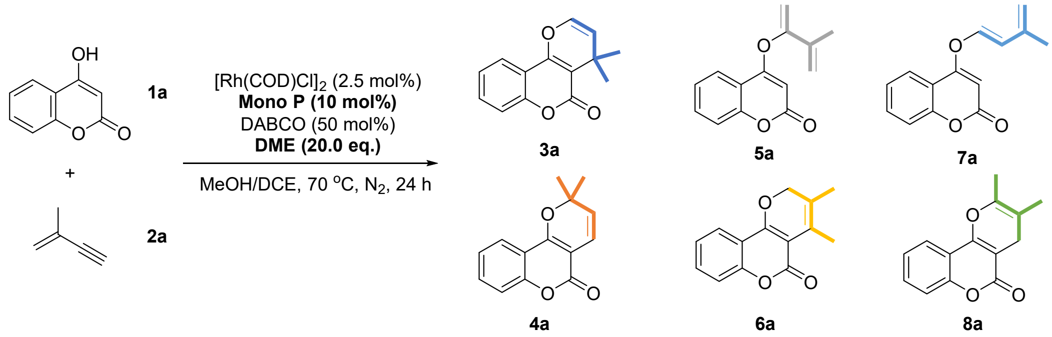

Entry	Mono P	3a	4a	5a	6a	7a	8a
1	PPh_3	trace	2%	trace	9%	trace	trace
2	$(4\text{-}MeOC_6H_4)_3P$	trace	3%	trace	trace	trace	trace
3	$(4\text{-}FC_6H_4)_3P$	trace	3%	trace	trace	trace	trace

B) Evaluation of mono P in the presence of DME under acidic condition

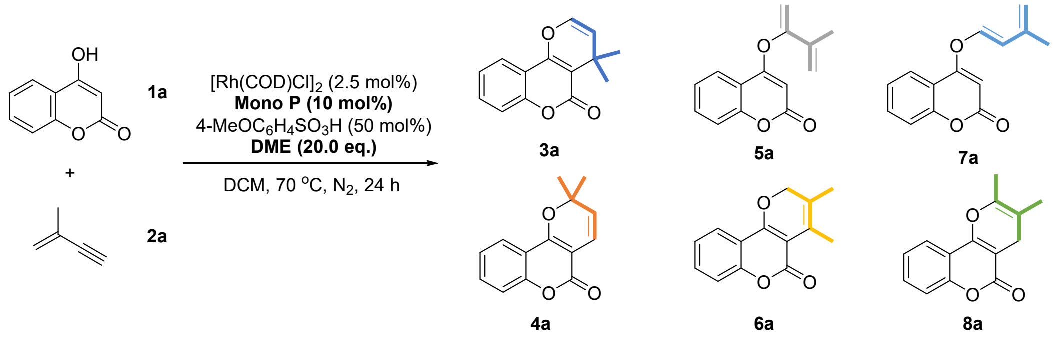

Entry	Mono P	3a	4a	5a	6a	7a	8a
1	PPh_3	trace	trace	5%	9%	trace	trace
2	$(4\text{-}MeOC_6H_4)_3P$	trace	3%	4%	trace	trace	trace
3	$(4\text{-}FC_6H_4)_3P$	trace	2%	2%	trace	trace	trace

3.3 Optimization of the reaction condition for the coupling of 9a and 2a.

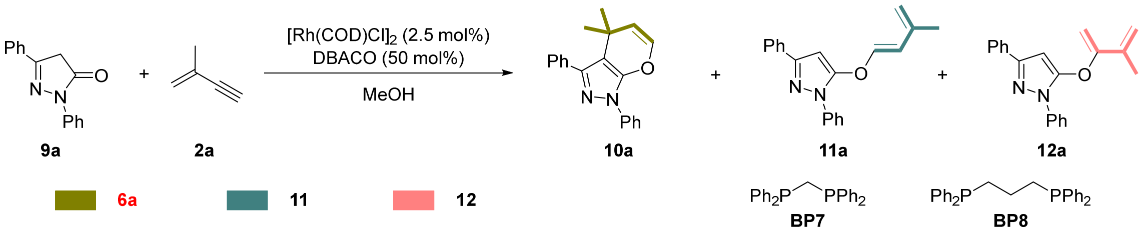

Table S9: Evaluation of Mono-P ligand with BINAP or Dppb (5 mol%)

Entry	Bis P-P	Mono-P ligand	[a]Yield (%)		
			**10a**	**11a**	**12a**
1[b]	BINAP	None	29%	trace	trace
2[b]		**MP5**	64%	1%	5%
3	Dppb	None	trace	trace	trace

4	**MP1**	43%	trace	trace
5	**MP2**	43%	trace	trace
6	**MP3**	34%	trace	trace
7	**MP4**	27%	trace	trace
8	**MP5**	63%	2%	1%
9	**MP6**	43%	3%	1%

Reaction condition: **5a** (0.20 mmol), **2a** (0.40 mmol), $[Rh(cod)Cl]_2$ (2.5 mol%), Mono-P ligand (10 mol%), Dppb (5 mol%), DABCO (50 mol%), DCE (0.2 mL), MeOH (0.3 mL), 70 ºC, 24 h. [a]Yields were determined by GC-Fid analysis of crude mixture with 1,3,5-trimethoxybenzene as the internal standard. [b]MeOH, 60 ^{o}C.

Table S10: Evaluation of Bis P-P ligand with $(4\text{-}ClC_6H_4)_3P$ (10 mol%)

Entry	**Bis P-P ligand**	[a]Yield (%)		
		**10a**	**11a**	**12a**
1	No BP	7%	trace	trace
2	**BP1**	64%	1%	5%
3	**BP2**	1%	2%	trace
4	**BP3**	63%	2%	1%
5	**BP4**	31%	1%	2%
6[b]	**BP6**	95%	trace	trace
7[b]	**BP7**	10%	trace	trace
8[b]	**BP8**	89%	2%	trace

Reaction condition: **5a** (0.20 mmol), **2a** (0.40 mmol), $[Rh(cod)Cl]_2$ (2.5 mol%), $(4\text{-}ClC_6H_4)_3P$ (10 mol%), Bis P-P ligand (5 mol%), DABCO (50 mol%), DCE (0.2 mL), MeOH (0.3 mL), 70 ºC, 24 h. [a]Yields were determined by GC-Fid analysis of crude mixture with 1,3,5-trimethoxybenzene as the internal standard. [b]MeOH, 60 ^{o}C.

Table S11: Evaluation of other reaction conditions

Entry	**Bis P-P ligand**	**Mono-P ligand**	**Solvent**	**Base**	[a]Yield (%)		
					**10a**	**11a**	**12a**
1[b]	Dppb	$P(4\text{-}ClC_6H_4)_3$	MeOH/DCE	DBACO	63%	1%	trace
2[b]	Dppb	$P(4\text{-}ClC_6H_4)_3$	MeOH/DCE	DBU	50%	2%	2%
3[b]	Dppb	$P(4\text{-}ClC_6H_4)_3$	MeOH/DCE	K_2CO_3	42%	2%	2%
4	Dppb	$P(4\text{-}ClC_6H_4)_3$	MeOH/DCE	DABCO	67%	2%	1%
5	Dppb	$P(4\text{-}ClC_6H_4)_3$	MeOH	DABCO	59%	1%	trace
6	Dppb	$P(4\text{-}ClC_6H_4)_3$	DCE	DABCO	5%	1%	trace
7	Dppm	$P(4\text{-}ClC_6H_4)_3$	MeOH	DABCO	10%	trace	trace
8	***Dppe***	***$P(4\text{-}ClC_6H_4)_3$***	***MeOH***	***DABCO***	***95%***	trace	trace
9	Dppp	$P(4\text{-}ClC_6H_4)_3$	MeOH	DABCO	89%	2%	trace
10	Dppe	-	MeOH	DABCO	14%	trace	trace

Reaction condition: **5a** (0.20 mmol), **2a** (0.40 mmol), $[Rh(cod)Cl]_2$ (2.5 mol%), Bis P-P ligand (5.0 mol%), Mono-P ligand (10 mol%), base (50 mol%), solvent (0.5 mL), 60 °C, 24 h. [a]Yields were determined by GC-Fid analysis of crude mixture with 1,3,5-trimethoxybenzene as the internal standard. [b]70 °C.

4. General procedure for Hydrofunctionalization of 1,3-Enynes.

4.1. General procedure A: Rh-catalyzed annulation of 1,3-Enynes with 4-Hydroxycoumarins under basic condition.

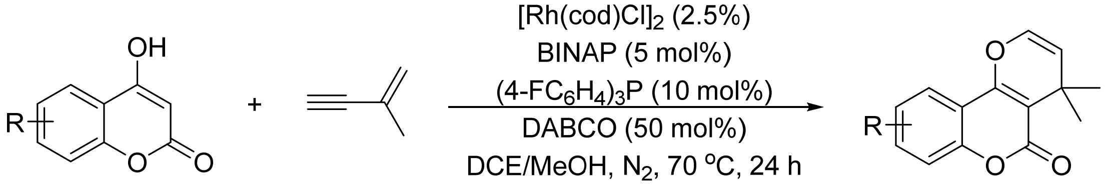

1 **2a** **3**

In a glovebox, **1** (0.20 mmol), $[Rh(cod)Cl]_2$ (0.005 mmol, 2.5 mol%), BINAP (0.01 mmol, 5 mol%), $(4\text{-}FC_6H_4)_3P$ (0.02 mmol, 10 mol%), DABCO (0.10 mmol, 50 mol%), DCE (0.2 mL) and MeOH (0.3 mL) were sequentially added to a vial (4.0 mL) with a stir bar at room temperature. 1,3-enynes **2a** was then added to the mixture via syringe. The reaction vial was sealed with a cap, removed from the glove box. Then, the reaction mixture was stirred at 70 °C for 24 h. After the starting material was consumed completely (monitored by TLC), the crude reaction mixture was purified by flash column chromatography on silica gel using petroleum and ethyl acetate (PE:EA = 20:1, v/v) to afford the desired products **3**.

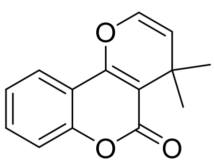

4,4-Dimethyl-4H,5H-pyrano[3,2-c]chromen-5-one (3a). Light yellow solid (m.p. 76 °C), 34.8 mg, 76% yield. **^{1}H NMR** (400 MHz, Chloroform-*d*) δ 7.72 (dd, *J* = 7.9, 1.4 Hz, 1H), 7.49 (ddd, *J* = 8.6, 7.3, 1.6 Hz, 1H), 7.29 – 7.21 (m, 2H), 6.47 (d, *J* = 6.2 Hz, 1H), 4.94 (d, *J* = 6.2 Hz, 1H), 1.51 (s, 6H). **^{13}C NMR** (101 MHz, $CDCl_3$) δ 161.07, 155.40, 152.41, 135.32, 131.71, 123.87, 123.00, 116.28, 116.05, 114.32, 107.87, 30.02, 29.62. **HRMS** Calculated for $C_{14}H_{12}O_3$ $[M+H]^+$ 229.0865, found 229.0857.

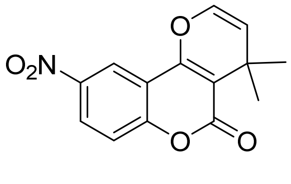

4,4-Dimethyl-9-nitro-4H,5H-pyrano[3,2-c]chromen-5-one (3b). Light yellow solid (m.p. 177 °C), 40.4 mg, 74% yield. **^{1}H NMR** (400 MHz, Chloroform-*d*) δ 7.87 (d, *J* = 2.3 Hz, 1H), 7.59 (dd, *J* = 8.8, 2.4 Hz, 1H), 7.17 (d, *J* = 8.8 Hz, 1H), 6.48 (d, *J* = 6.2 Hz, 1H), 4.96 (d, *J* = 6.2 Hz, 1H), 1.52 (s, 6H). **^{13}C NMR** (101 MHz, $CDCl_3$) δ 160.40, 154.33, 151.25, 135.26, 134.55, 125.69, 118.06, 116.66, 116.07, 115.91, 108.73, 30.12,

29.58. **HRMS** Calculated for $C_{14}H_{12}NO_5$ $[M+H]^+$ 274.0715, found 274.0705.

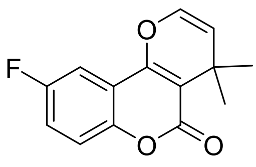

9-Fluoro-4,4-dimethyl-4H,5H-pyrano[3,2-c]chromen-5-one (3c). Light yellow solid (m.p. 111 °C), 37.7 mg, 77% yield. **^{1}H NMR** (400 MHz, Chloroform-*d*) δ 7.41 (dd, *J* = 8.5, 2.9 Hz, 1H), 7.30 – 7.20 (m, 2H), 6.49 (d, *J* = 6.2 Hz, 1H), 4.97 (d, *J* = 6.2 Hz, 1H), 1.54 (s, 6H). **^{13}C NMR** (101 MHz, $CDCl_3$) δ 160.70, 158.61 (d, *J* = 243.5 Hz), 154.68 (d, *J* = 2.8 Hz), 148.53 (d, *J* = 1.9 Hz), 135.26, 119.26 (d, *J* = 24.6 Hz), 117.91 (d, *J* = 8.3 Hz), 116.06, 115.23 (d, *J* = 9.2 Hz), 108.92, 108.66, 30.09, 29.56. **^{19}F NMR** (376 MHz, $CDCl_3$) δ -117.36. **HRMS** Calculated for $C_{14}H_{12}FO_3$ $[M+H]^+$ 247.0770, found 247.0768.

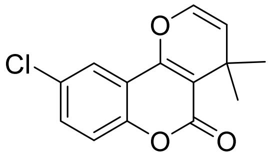

9-Chloro-4,4-dimethyl-4H,5H-pyrano[3,2-c]chromen-5-one (3d). Light yellow solid (m.p. 148 °C), 34.9 mg, 66% yield. **^{1}H NMR** (400 MHz, Chloroform-*d*) δ 7.64 (d, *J* = 2.5 Hz, 1H), 7.38 (dd, *J* = 8.8, 2.5 Hz, 1H), 7.16 (d, *J* = 8.8 Hz, 1H), 6.41 (d, *J* = 6.2 Hz, 1H), 4.89 (d, *J* = 6.2 Hz, 1H), 1.46 (s, 6H). **^{13}C NMR** (101 MHz, $CDCl_3$) δ 160.45, 154.42, 150.78, 135.26, 131.73, 129.40, 122.63, 117.76, 116.07, 115.47, 108.73, 30.11, 29.57. **HRMS** Calculated for $C_{14}H_{12}ClO_3$ $[M+H]^+$ 263.0475, found 263.0481

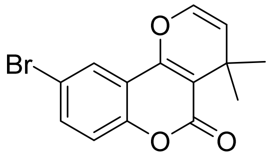

9-Bromo-4,4-dimethyl-4H,5H-pyrano[3,2-c]chromen-5-one (3e). Light yellow solid (m.p. 74 °C), 28.6 mg, 47% yield. **^{1}H NMR** (400 MHz, Chloroform-*d*) δ 7.73 (dd, *J* = 7.9, 1.5 Hz, 1H), 7.50 (ddd, *J* = 8.6, 7.3, 1.6 Hz, 1H), 7.29 – 7.23 (m, 1H), 6.48 (d, *J* = 6.2 Hz, 1H), 4.95 (d, *J* = 6.2 Hz, 1H), 1.53 (s, 6H). **^{13}C NMR** (101 MHz, $CDCl_3$) δ 161.07, 155.40, 152.40, 135.32, 131.71, 123.87, 123.00, 116.27, 116.04, 114.31, 107.86, 30.02, 29.62. **HRMS** Calculated for $C_{14}H_{12}BrO_3$ $[M+H]^+$ 306.9970, found 306.9963.

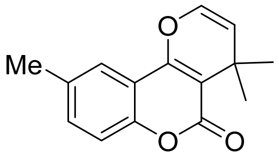

4,4,9-Trimethyl-4H,5H-pyrano[3,2-c]chromen-5-one (3f). Light yellow solid (m.p. 115 °C), 43.8 mg, 90% yield. **^{1}H NMR** (400 MHz, Chloroform-*d*) δ 7.54 – 7.51 (d, *J* = 1.7 Hz, 1H), 7.31 (dd, *J* = 8.4, 1.7 Hz, 1H), 7.18 (d, *J* = 8.4 Hz, 1H), 6.48 (d, *J* = 6.2 Hz, 1H), 4.95 (d, *J* = 6.2 Hz, 1H), 2.40 (s, 3H), 1.53 (s, 6H). **^{13}C NMR** (101 MHz, $CDCl_3$) δ 161.28, 155.39, 150.56, 135.28, 133.55, 132.73, 122.66, 116.04, 116.01, 113.94, 107.72, 30.01, 29.63, 20.98. **HRMS** Calculated for $C_{15}H_{15}O_3$ $[M+H]^+$ 243.1021, found 243.1017.

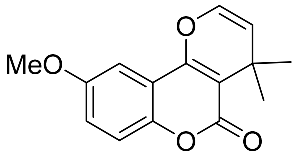

9-Methoxy-4,4-dimethyl-4H,5H-pyrano[3,2-c]chromen-5-one (3g). Light yellow solid (m.p. 104 °C), 42.4 mg, 82% yield. **^{1}H NMR** (400 MHz, Chloroform-*d*) δ 7.20 (d, *J* = 9.0 Hz, 1H), 7.14

(d, J = 2.9 Hz, 1H), 7.07 (dd, J = 9.0, 3.0 Hz, 1H), 6.48 (d, J = 6.2 Hz, 1H), 4.94 (d, J = 6.2 Hz, 1H), 3.83 (s, 3H), 1.52 (s, 6H). **^{13}C NMR** (101 MHz, $CDCl_3$) δ 161.21, 155.81, 155.18, 146.91, 135.22, 120.01, 117.44, 116.09, 114.58, 108.03, 104.65, 55.84, 30.06, 29.62. **HRMS** Calculated for $C_{15}H_{15}O_4$ $[M+H]^+$ 259.0970, found 259.0957.

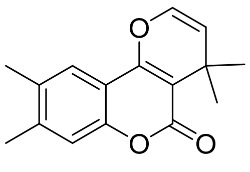

4,4,8,9-Tetramethyl-4H,5H-pyrano[3,2-c]chromen-5-one (3h). Light yellow solid (m.p. 95 °C), 45.2 mg, 88% yield. **^{1}H NMR** (400 MHz, Chloroform-*d*) δ 7.45 (s, 1H), 7.04 (s, 1H), 6.47 (d, J = 6.2 Hz, 1H), 4.94 (d, J = 6.2 Hz, 1H), 2.33 (s, 3H), 2.29 (s, 3H), 1.52 (s, 6H). **^{13}C NMR** (101 MHz, $CDCl_3$) δ 161.49, 155.56, 150.85, 141.68, 135.26, 132.64, 122.87, 116.71, 116.05, 111.78, 106.81, 29.93, 29.62, 20.27, 19.35. **HRMS** Calculated for $C_{16}H_{17}O_3$ $[M+H]^+$ 257.1178, found 257.1160.

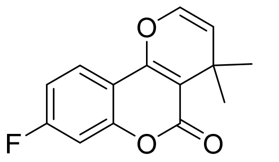

8-Fluoro-4,4-dimethyl-4H,5H-pyrano[3,2-c]chromen-5-one (3i). Light yellow solid (m.p. 73 °C), 38.8 mg, 79% yield. **^{1}H NMR** (400 MHz, Chloroform-*d*) δ 7.73 (dd, J = 9.5, 6.1 Hz, 1H), 7.03 – 6.98 (m, 2H), 6.48 (d, J = 6.2 Hz, 1H), 4.96 (d, J = 6.2 Hz, 1H), 1.52 (s, 6H). **^{13}C NMR** (101 MHz, $CDCl_3$) δ 163.44 (d, J = 253.3 Hz), 159.77, 154.09, 152.52 (d, J = 13.2 Hz), 134.22, 123.84 (d, J = 10.2 Hz), 115.13, 111.02 (d, J = 22.8 Hz), 109.94 (d, J = 2.7 Hz), 105.85 (d, J = 2.5 Hz), 102.69 (d, J = 25.6 Hz), 28.89, 28.51. **^{19}F NMR** (376 MHz, $CDCl_3$) δ -105.64. **HRMS** Calculated for $C_{14}H_{12}FO_3$ $[M+H]^+$ 247.0770, found 247.0763.

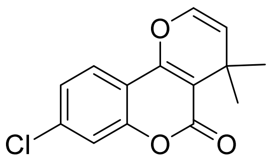

8-Chloro-4,4-dimethyl-4H,5H-pyrano[3,2-c]chromen-5-one (3j). Light yellow solid (m.p. 92 °C), 27.7 mg, 53% yield. **^{1}H NMR** (400 MHz, Chloroform-*d*) δ 7.66 (d, J = 8.5 Hz, 1H), 7.30 (d, J = 1.9 Hz, 1H), 7.24 (dd, J = 8.5, 2.0 Hz, 1H), 6.48 (d, J = 6.1 Hz, 1H), 4.96 (d, J = 6.1 Hz, 1H), 1.52 (s, 6H). **^{13}C NMR** (101 MHz, $CDCl_3$) δ 160.46, 154.97, 152.66, 137.60, 135.26, 124.51, 124.09, 116.53, 116.10, 112.96, 107.89, 30.03, 29.55. **HRMS** Calculated for $C_{14}H_{12}ClO_3$ $[M+H]^+$ 263.0475, found 263.0480.

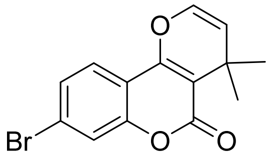

8-Bromo-4,4-dimethyl-4H,5H-pyrano[3,2-c]chromen-5-one (3k). Light yellow solid (m.p. 110 °C), 39.8 mg, 76% yield. **^{1}H NMR** (400 MHz, Chloroform-*d*) δ 7.59 (d, *J* = 8.5 Hz, 1H), 7.46 (d, *J* = 1.7 Hz, 1H), 7.39 (dd, *J* = 8.5, 1.8 Hz, 1H), 6.47 (d, *J* = 6.2 Hz, 1H), 4.96 (d, *J* = 6.2 Hz, 1H), 1.52 (s, 6H). **^{13}C NMR** (101 MHz, $CDCl_3$) δ 159.29, 153.97, 151.58, 134.23, 126.28, 124.54, 123.15, 118.44, 115.06, 112.31, 107.09, 29.03, 28.51. **HRMS** Calculated for $C_{14}H_{12}BrO_3$ $[M+H]^+$ 306.9970, found 306.9977.

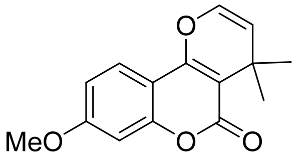

8-Methoxy-4,4-dimethyl-4H,5H-pyrano[3,2-c]chromen-5-one (3l). Light yellow solid (m.p. 67 °C), 44.8 mg, 87% yield. **^{1}H NMR** (400 MHz, Chloroform-*d*) δ 7.62 (d, *J* = 8.8 Hz, 1H), 6.83 (dd, *J* = 8.8, 2.3 Hz, 1H), 6.76 (d, *J* = 2.3 Hz, 1H), 6.47 (d, *J* = 6.2 Hz, 1H), 4.95 (d, *J* = 6.2 Hz, 1H), 3.86 (s, 3H), 1.52 (s, 6H). **^{13}C NMR** (101 MHz, $CDCl_3$) δ 162.67, 161.50, 155.79, 154.16, 135.26, 124.03, 116.10, 112.23, 107.52, 105.13, 99.98, 55.72, 29.78, 29.56. **HRMS** Calculated for $C_{15}H_{15}O_4$ $[M+H]^+$ 259.0970, found 259.0970.

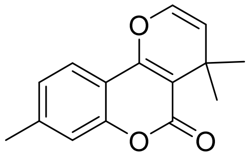

4,4,8-Trimethyl-4H,5H-pyrano[3,2-c]chromen-5-one (3m). Light yellow solid (m.p. 74 °C), 39.7 mg, 82% yield. **^{1}H NMR** (400 MHz, Chloroform-*d*) δ 7.60 (d, *J* = 7.9 Hz, 1H), 7.07 (m, 2H), 6.47 (d, *J* = 6.2 Hz, 1H), 4.94 (d, *J* = 6.2 Hz, 1H), 2.43 (s, 3H), 1.52 (s, 6H). **^{13}C NMR** (101 MHz, $CDCl_3$) δ 161.32, 155.60, 152.54, 142.84, 135.29, 125.09, 122.68, 116.37, 116.07, 111.80, 106.89, 29.92, 29.61, 21.71. **HRMS** Calculated for $C_{15}H_{15}O_3$ $[M+H]^+$ 243.1021, found 243.1011.

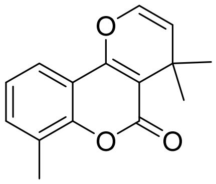

4,4,7-Trimethyl-4H,5H-pyrano[3,2-c]chromen-5-one (3n). Light yellow solid (m.p. 150 °C), 43.9 mg, 91% yield. **^{1}H NMR** (400 MHz, Chloroform-*d*) δ 7.57 (d, *J* = 7.9 Hz, 1H), 7.35 (d, *J* = 7.3 Hz, 1H), 7.15 (t, *J* = 7.7 Hz, 1H), 6.48 (d, *J* = 6.2 Hz, 1H), 4.95 (d, *J* = 6.2 Hz, 1H), 2.44 (s, 3H), 1.54 (s, 6H). **^{13}C NMR** (101 MHz, $CDCl_3$) δ 161.10, 155.65, 150.78, 135.36, 132.86, 125.66, 123.38, 120.56, 115.97, 114.01, 107.53, 30.00, 29.61, 15.50. **HRMS** Calculated for $C_{15}H_{15}O_3$ $[M+H]^+$ 243.1021, found 243.1004.

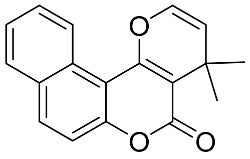

4,4-Dimethyl-4H,5H-benzo[f]pyrano[3,2-c]chromen-5-one (3o). Light yellow solid (m.p. 166 °C), 24.9 mg, 45% yield. **^{1}H NMR** (400 MHz, Chloroform-*d*) δ 9.04 (d, *J* = 8.8 Hz, 1H), 7.94 (d, *J* = 8.9 Hz, 1H), 7.86 (d, *J* = 7.8 Hz, 1H), 7.62 (t, *J* = 7.2 Hz, 1H), 7.53 (t, *J* = 7.5 Hz, 1H), 7.41 (d, *J* = 8.9 Hz, 1H), 6.65 (d, *J* = 6.1 Hz, 1H), 5.05 (d, *J* = 6.1 Hz, 1H), 1.61 (s, 6H). **^{13}C NMR** (101 MHz, $CDCl_3$) δ 160.75, 159.13, 153.38, 134.70, 133.73, 130.96,

129.15, 128.92, 128.18, 126.29, 125.59, 116.94, 116.11, 107.81, 107.69, 29.96, 29.82. **HRMS** Calculated for $C_{18}H_{15}O_3$ $[M+H]^+$ 279.1021, found 279.1014.

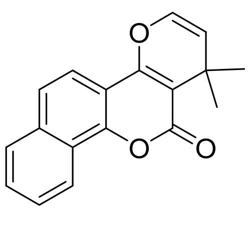

1,1-Dimethyl-1H,12H-benzo[h]pyrano[3,2-c]chromen-12-one (3p). Light yellow solid (m.p. 164 °C), 42.8 mg, 77% yield. **^{1}H NMR** (400 MHz, Chloroform-*d*) δ 8.54 – 8.48 (m, 1H), 7.87 – 7.82 (m, 1H), 7.74 – 7.59 (m, 4H), 6.53 (d, *J* = 6.1 Hz, 1H), 4.99 (d, *J* = 6.2 Hz, 1H), 1.58 (s, 6H). **^{13}C NMR** (101 MHz, $CDCl_3$) δ 161.11, 156.34, 149.75, 135.41, 134.74, 128.55, 127.75, 126.99, 123.84, 122.67, 122.41, 118.59, 116.09, 109.53, 107.34, 30.01, 29.58. **HRMS** Calculated for $C_{18}H_{15}O_3$ $[M+H]^+$ 279.1021, found 279.1029.

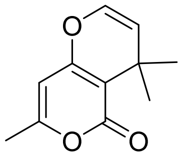

4,4,7-Trimethyl-4H,5H-pyrano[4,3-b]pyran-5-one (3r). Light yellow solid (m.p. 56 °C), 24.5 mg, 64% yield. **^{1}H NMR** (400 MHz, Chloroform-*d*) δ 6.27 (d, *J* = 6.2 Hz, 1H), 5.70 (s, 1H), 4.84 (d, *J* = 6.2 Hz, 1H), 2.17 (s, 3H), 1.43 (s, 6H). **^{13}C NMR** (101 MHz, $CDCl_3$) δ 162.99, 160.67, 160.03, 135.25, 115.99, 104.86, 99.05, 29.30, 19.69. **HRMS** Calculated for $C_{11}H_{13}O_3$ $[M+H]^+$ 193.0865, found 193.0866.

4.2. General procedure B: Rh-catalyzed annulation of 1,3-Enynes with 4-Hydroxycoumarins under acidic condition.

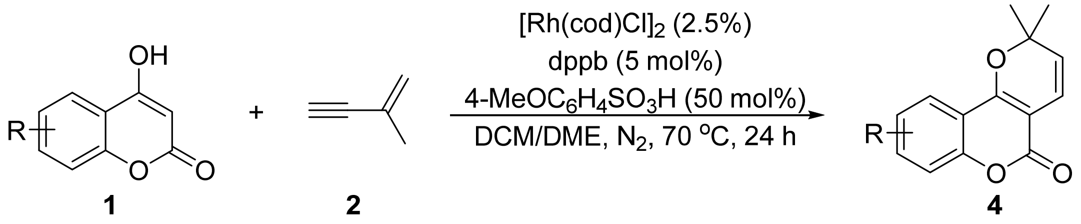

In a glove box, **1** (0.20 mmol), $[Rh(cod)Cl]_2$ (0.005 mmol, 2.5 mol%), dppb (0.01 mmol, 5 mol%), $4\text{-}MeOC_6H_4SO_3H$ (0.10 mmol, 50 mol%), DCM (0.5 mL) and DME (0.4 mL) were sequentially added to a vial (4.0 mL) with a stir bar at room temperature. 1,3-enynes **2a** was then added to the mixture via syringe. The reaction vial was sealed with a cap, removed from the glove box. Then, the reaction mixture was stirred at 70 °C for 24 h. After the starting material was consumed completely (monitored by TLC), the crude reaction mixture was purified by flash column chromatography on silica gel using petroleum and ethyl acetate (PE:EA = 20:1, v/v) to afford the desired products **4**.

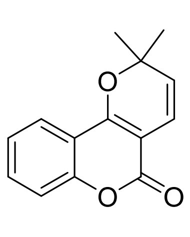

2,2-Dimethyl-2H,5H-pyrano[3,2-c]chromen-5-one (4a).[3] Known compound, light yellow solid, 36.4 mg, 80% yield. **^{1}H NMR** (400 MHz, Chloroform-*d*) δ 7.80 (dd, *J* = 7.9, 1.5 Hz, 1H), 7.52 (ddd, *J* = 8.7, 7.3, 1.6 Hz, 1H), 7.34 – 7.23 (m, 2H), 6.54 (d, *J* = 10.0 Hz, 1H), 5.54 (d, *J* = 10.0 Hz, 1H), 1.55 (s, 6H). **^{13}C NMR** (101 MHz, $CDCl_3$) δ 160.96, 158.83, 153.15, 132.08, 126.19, 123.97, 122.74, 116.74, 115.56, 100.26, 80.57, 28.58.

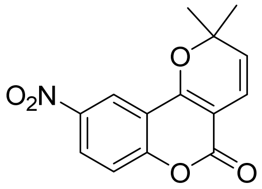

2,2-Dimethyl-9-nitro-2H,5H-pyrano[3,2-c]chromen-5-one (4b).[4] Known compound, light yellow solid, 35.6 mg, 65% yield. **^{1}H NMR** (400 MHz, Chloroform-*d*) δ 7.79 (dd, *J* = 7.9, 1.5 Hz, 1H), 7.51 (ddd, *J* = 8.6, 7.3, 1.6 Hz, 1H), 7.31 – 7.24 (m, 2H), 6.53 (d, *J* = 10.0 Hz, 1H), 5.52 (d, *J* = 10.0 Hz, 1H), 1.54 (s, 6H). **^{13}C NMR** (101 MHz, $CDCl_3$) δ 160.96, 158.83, 153.14, 132.09, 126.19, 123.97, 122.74, 116.73, 115.55, 100.25, 80.57, 28.58.

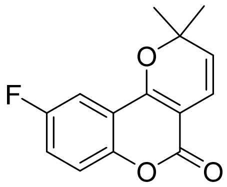

9-Fluoro-2,2-dimethyl-2H,5H-pyrano[3,2-c]chromen-5-one (4c).[5] Known compound, light yellow solid, 25.2 mg, 51% yield. **^{1}H NMR** (400 MHz, Chloroform-*d*) δ 7.44 (dd, *J* = 8.3, 2.9 Hz, 1H), 7.31 – 7.23 (dd, *J* = 9.0, 4.5 Hz, 1H), 7.22 (ddd, *J* = 9.1, 7.7, 2.9 Hz, 1H), 6.52 (d, *J* = 10.0 Hz, 1H), 5.56 (d, *J* = 10.0 Hz, 1H), 1.55 (s, 6H). **^{13}C NMR** (101 MHz, $CDCl_3$) δ 160.63, 158.72 (d, *J* = 242.3 Hz), 157.92 (d, *J* = 2.8 Hz), 149.22 (d, *J* = 1.9 Hz), 126.84, 119.53 (d, *J* = 24.6 Hz), 118.35 (d, *J* = 8.2 Hz), 116.49, 116.48 (d, *J* = 6.3 Hz), 108.35 (d, *J* = 25.3 Hz), 100.85, 80.95, 28.60. **^{19}F NMR** (376 MHz, $CDCl_3$) δ -117.30.

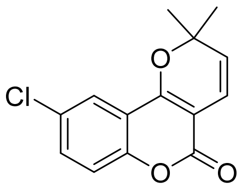

9-Chloro-2,2-dimethyl-2H,5H-pyrano[3,2-c]chromen-5-one (4d).[5] Known compound, light yellow solid, 31.6 mg, 57% yield. **^{1}H NMR** (400 MHz, Chloroform-*d*) δ 7.75 (d, *J* = 2.5 Hz, 1H), 7.46 (dd, *J* = 8.8, 2.5 Hz, 1H), 7.24 (d, *J* = 8.8 Hz, 1H), 6.52 (d, *J* = 10.0 Hz, 1H), 5.57 (d, *J* = 10.0 Hz, 1H), 1.56 (s, 6H). **^{13}C NMR** (101 MHz, $CDCl_3$) δ 160.34, 157.59, 151.45, 131.96, 129.54, 126.87, 122.20, 118.19, 116.69, 116.44, 100.89, 81.02, 28.60.

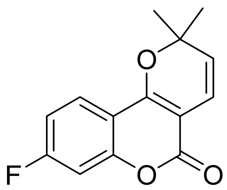

8-Fluoro-2,2-dimethyl-2H,5H-pyrano[3,2-c]chromen-5-one (4e).[5] Known compound, light yellow solid, 18.2 mg, 37% yield. **^{1}H NMR** (400 MHz, Chloroform-*d*) δ 7.79 (dd, *J* = 9.5, 6.1 Hz, 1H), 7.04 – 6.98 (m, 2H), 6.51 (d, *J* = 10.0 Hz, 1H), 5.52 (d, *J* = 10.0 Hz, 1H), 1.54 (s, 6H). ^{13}C NMR (101 MHz, $CDCl_3$) δ 164.80 (d, *J* = 253.7 Hz), 160.67, 158.51, 154.30 (d, *J* = 13.2 Hz), 126.03, 124.57 (d, *J* = 10.3 Hz), 116.54, 112.21 (d, *J* = 2.6

Hz), 112.17 (d, J = 22.9 Hz), 104.24 (d, J = 25.6 Hz), 99.27 (d, J = 2.2 Hz), 80.84, 28.59. **^{19}F NMR** (376 MHz, $CDCl_3$) δ -104.86.

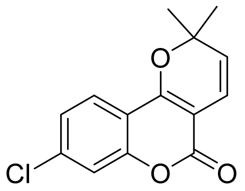

8-Chloro-2,2-dimethyl-2H,5H-pyrano[3,2-c]chromen-5-one (4f). Light yellow solid (m.p. 139 °C), 29.9 mg, 57% yield. **^{1}H NMR** (400 MHz, Chloroform-*d*) δ 7.72 (d, J = 8.5 Hz, 1H), 7.30 (d, J = 1.9 Hz, 1H), 7.24 (dd, J = 8.5, 1.9 Hz, 1H), 6.50 (d, J = 10.0 Hz, 1H), 5.54 (d, J = 10.0 Hz, 1H), 1.54 (s, 6H). **^{13}C NMR** (101 MHz, $CDCl_3$) δ 160.37, 158.26, 153.32, 137.96, 126.48, 124.62, 123.75, 116.98, 116.51, 114.17, 100.19, 80.90, 28.60. **HRMS** Calculated for $C_{14}H_{12}ClO_3$ $[M+H]^+$ 263.0475, found 263.0477.

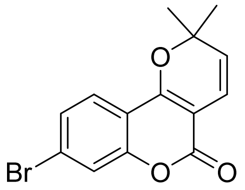

8-Bromo-2,2-dimethyl-2H,5H-pyrano[3,2-c]chromen-5-one (4g). Light yellow solid (m.p. 146 °C), 41.5 mg, 68% yield. **^{1}H NMR** (400 MHz, Chloroform-*d*) δ 7.63 (d, J = 8.4 Hz, 1H), 7.44 (d, J = 1.5 Hz, 1H), 7.38 (dd, J = 8.4, 1.6 Hz, 1H), 6.48 (d, J = 10.0 Hz, 1H), 5.53 (d, J = 10.0 Hz, 1H), 1.53 (s, 6H). **^{13}C NMR** (100 MHz, $CDCl_3$) δ 159.18, 157.23, 152.20, 126.38, 125.52, 124.94, 122.79, 118.85, 115.48, 113.52, 99.34, 79.87, 27.55. **HRMS** Calculated for $C_{14}H_{12}BrO_3$ $[M+H]^+$ 306.9970, found 306.9992.

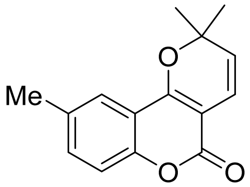

2,2,9-Trimethyl-2H,5H-pyrano[3,2-c]chromen-5-one (4h).[5] Known compound, light yellow solid, 33.4 mg, 69% yield. **^{1}H NMR** (400 MHz, Chloroform-*d*) δ 7.59 – 7.54 (d, J = 1.9 Hz, 1H), 7.32 (dd, J = 8.5, 1.9 Hz, 1H), 7.19 (d, J = 8.4 Hz, 1H), 6.54 (d, J = 10.0 Hz, 1H), 5.52 (d, J = 10.0 Hz, 1H), 2.42 (s, 3H), 1.56 (s, 6H). **^{13}C NMR** (101 MHz, $CDCl_3$) δ 161.16, 158.84, 151.33, 133.71, 133.15, 126.02, 122.34, 116.86, 116.49, 115.19, 100.19, 80.45, 28.56, 20.92.

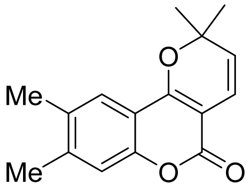

2,2,8,9-Tetramethyl-2H,5H-pyrano[3,2-c]chromen-5-one (4i).[6] Known compound, light yellow solid, 28.5 mg, 59% yield. **^{1}H NMR** (400 MHz, Chloroform-*d*) δ 7.51 (s, 1H), 7.07 (s, 1H), 6.53 (d, J = 10.0 Hz, 1H), 5.49 (d, J = 10.0 Hz, 1H), 2.34 (s, 3H), 2.32 (s, 3H), 1.54 (s, 6H). **^{13}C NMR** (101 MHz, $CDCl_3$) δ 161.34, 159.13, 151.66, 142.28, 132.81, 125.52, 122.61, 117.27, 116.99, 113.05, 99.46, 80.31, 28.52, 20.35, 19.28.

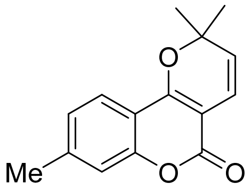

2,2,8-Trimethyl-2H,5H-pyrano[3,2-c]chromen-5-one (4j).[5] Known compound, light yellow solid, 19.1 mg, 39% yield. **^{1}H NMR** (400 MHz, Chloroform-*d*) δ 7.66 (d, *J* = 7.9 Hz, 1H), 7.07 (m, 2H), 6.52 (d, *J* = 10.0 Hz, 1H), 5.49 (d, *J* = 10.0 Hz, 1H), 2.43 (s, 3H), 1.53 (s, 6H). **^{13}C NMR** (101 MHz, $CDCl_3$) δ 161.15, 159.12, 153.30, 143.37, 125.68, 125.19, 122.46, 116.86, 113.05, 99.43, 80.40, 28.54, 21.80.

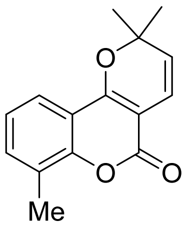

2,2,7-Trimethyl-2H,5H-pyrano[3,2-c]chromen-5-one (4k).[7] Known compound, light yellow solid, 20.6 mg, 43% yield. **^{1}H NMR** (400 MHz, Chloroform-*d*) δ 7.65 (d, *J* = 7.9 Hz, 1H), 7.37 (d, *J* = 7.3 Hz, 1H), 7.17 (t, *J* = 7.6 Hz, 1H), 6.56 (d, *J* = 10.4 Hz, 1H), 5.53 (d, *J* = 10.0 Hz, 1H), 2.45 (s, 3H), 1.55 (s, 6H). **^{13}C NMR** (101 MHz, $CDCl_3$) δ 160.97, 159.14, 151.52, 133.34, 126.12, 126.03, 123.47, 120.36, 116.86, 115.29, 100.01, 80.39, 28.52, 15.69.

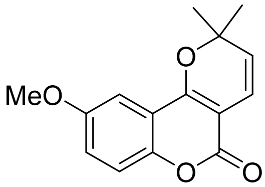

9-Methoxy-2,2-dimethyl-2H,5H-pyrano[3,2-c]chromen-5-one (4l).[5] Known compound, light yellow solid, 11.2 mg, 22% yield. **^{1}H NMR** (400 MHz, Chloroform-*d*) δ 7.23 (d, *J* = 9.0 Hz, 1H), 7.18 (d, *J* = 3.0 Hz, 1H), 7.10 (dd, *J* = 9.0, 3.0 Hz, 1H), 6.54 (d, *J* = 10.0 Hz, 1H), 5.53 (d, *J* = 10.0 Hz, 1H), 3.87 (s, 3H), 1.55 (s, 6H). **^{13}C NMR** (101 MHz, $CDCl_3$) δ 161.12, 158.58, 155.91, 147.68, 126.23, 120.17, 117.90, 116.87, 115.88, 104.43, 100.47, 80.61, 55.91, 28.58.

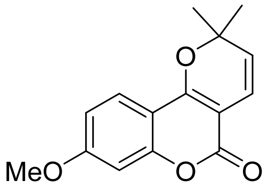

8-Methoxy-2,2-dimethyl-2H,5H-pyrano[3,2-c]chromen-5-one (4m).[5] Known compound, light yellow solid, 28.9 mg, 56% yield. **^{1}H NMR** (400 MHz, Chloroform-*d*) δ 7.69 (d, *J* = 8.8 Hz, 1H), 6.85 (d, *J* = 2.4 Hz, 1H), 6.77 (d, *J* = 2.4 Hz, 1H), 6.51 (d, *J* = 10.0 Hz, 1H), 5.46 (d, *J* = 10.0 Hz, 1H), 3.86 (s, 3H), 1.52 (s, 6H). **^{13}C NMR** (101 MHz, $CDCl_3$) δ 163.15, 161.32, 159.45, 155.03, 125.04, 123.88, 116.88, 112.41, 108.77, 100.50, 97.88, 80.43, 55.78, 28.56.

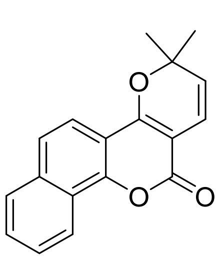

3,3-Dimethyl-3H,12H-benzo[h]pyrano[3,2-c]chromen-12-one (4n). Light yellow solid (m.p. 160 °C), 37.2 mg, 67% yield. **^{1}H NMR** (400 MHz, Chloroform-*d*) δ 8.55 – 8.48 (m, 1H), 7.86 – 7.81 (m, 1H), 7.76 (d, *J* = 8.7 Hz, 1H), 7.66 – 7.58 (m, 3H), 6.59 (d, *J* = 10.0 Hz, 1H), 5.54 (d, *J* = 10.0 Hz, 1H), 1.58 (s, 6H). **^{13}C NMR** (101 MHz, $CDCl_3$) δ 160.92, 159.87, 150.57, 135.05, 128.66, 127.82, 127.04, 125.88, 123.95, 122.95, 122.60, 118.31, 116.86, 110.76, 99.94, 80.66, 28.64. **HRMS** Calculated

for $C_{18}H_{15}O_3$ [M+H]$^+$ 279.1021, found 279.1014.

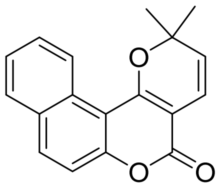

2,2-Dimethyl-2H,5H-benzo[f]pyrano[3,2-c]chromen-5-one (4o). Light yellow solid (m.p. 138 °C), 34.4 mg, 62% yield. **^{1}H NMR** (400 MHz, Chloroform-*d*) δ 9.22 (d, *J* = 8.8 Hz, 1H), 7.93 (d, *J* = 9.0 Hz, 1H), 7.86 (d, *J* = 7.9 Hz, 1H), 7.63 (t, *J* = 7.8 Hz, 1H), 7.54 (t, *J* = 7.5 Hz, 1H), 7.41 (d, *J* = 9.0 Hz, 1H), 6.63 (d, *J* = 9.9 Hz, 1H), 5.58 (d, *J* = 9.9 Hz, 1H), 1.68 (s, 6H). **^{13}C NMR** (101 MHz, $CDCl_3$) δ 162.37, 160.63, 154.16, 133.88, 130.85, 129.04, 129.01, 128.23, 126.15, 125.76, 125.57, 117.42, 117.30, 109.04, 101.03, 81.02, 28.56. **HRMS** Calculated for $C_{18}H_{15}O_3$ [M+H]$^+$ 279.1021, found 279.1024.

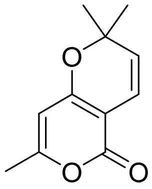

2,2,7-Trimethyl-2H,5H-pyrano[4,3-b]pyran-5-one (4p).[8] Known compound, light yellow solid, 8.1 mg, 21% yield. **^{1}H NMR** (400 MHz, Chloroform-*d*) δ 6.38 (d, *J* = 10.0 Hz, 1H), 5.77 (s, 1H), 5.36 (d, *J* = 10.0 Hz, 1H), 2.21 (d, *J* = 1.0 Hz, 3H), 1.44 (s, 6H). **^{13}C NMR** (101 MHz, $CDCl_3$) δ 164.16, 162.38, 124.84, 116.33, 100.33, 97.90, 80.11, 28.53, 20.21.

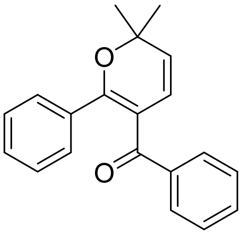

(2,2-Dimethyl-6-phenyl-2H-pyran-5-yl)(phenyl)methanone (4q). Light yellow oil, 16.1 mg (0.10 mmol scale), 51% yield (with 10% inseparable isomers). **^{1}H NMR** (400 MHz, Chloroform-*d*) δ 7.90 – 7.84 (m, 2H), 7.78 – 7.73 (m, 2H), 7.56 (d, *J* = 12.2 Hz, 1H), 7.53 – 7.48 (m, 2H), 7.41 (m, 4H), 6.16 (d, *J* = 12.2 Hz, 1H), 1.89 (s, 3H), 1.86 (s, 3H). **^{13}C NMR** (101 MHz, $CDCl_3$) δ 196.24, 194.68, 152.23, 141.83, 138.13, 137.44, 136.75, 133.45, 132.21, 129.22, 129.08, 128.68, 128.40, 121.49, 27.22, 19.17.

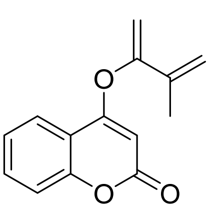

4-((3-Methylbuta-1,3-dien-2-yl)oxy)-2H-chromen-2-one (5a). Light yellow solid, **^{1}H NMR** (400 MHz, Chloroform-*d*) δ 7.96 (dd, *J* = 7.9, 1.5 Hz, 1H), 7.60 (ddd, *J* = 8.7, 7.4, 1.6 Hz, 1H), 7.39 – 7.30 (m, 2H), 5.73 (s, 1H), 5.28 (d, *J* = 2.1 Hz, 1H), 5.19 (s, 1H), 5.08 (s, 1H), 5.06 (d, *J* = 1.9 Hz, 2H), 2.00 (s, 3H). **^{13}C NMR** (101 MHz, $CDCl_3$) δ 165.19, 162.82, 155.65, 153.67, 134.82, 132.65, 124.09, 122.83, 116.86, 115.28, 115.28, 104.17, 92.99, 19.39. **HRMS** Calculated for $C_{14}H_{12}O_3$ [M+H]$^+$ 229.0865, found 229.0865.

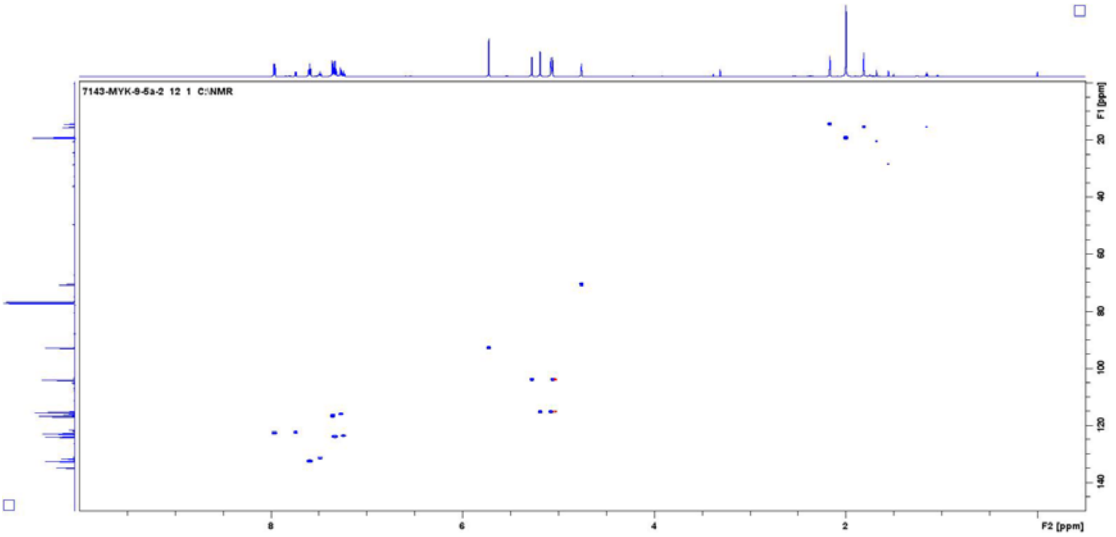

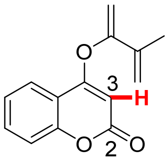

5a

HSQC (700 MHz, Chloroform-*d*)

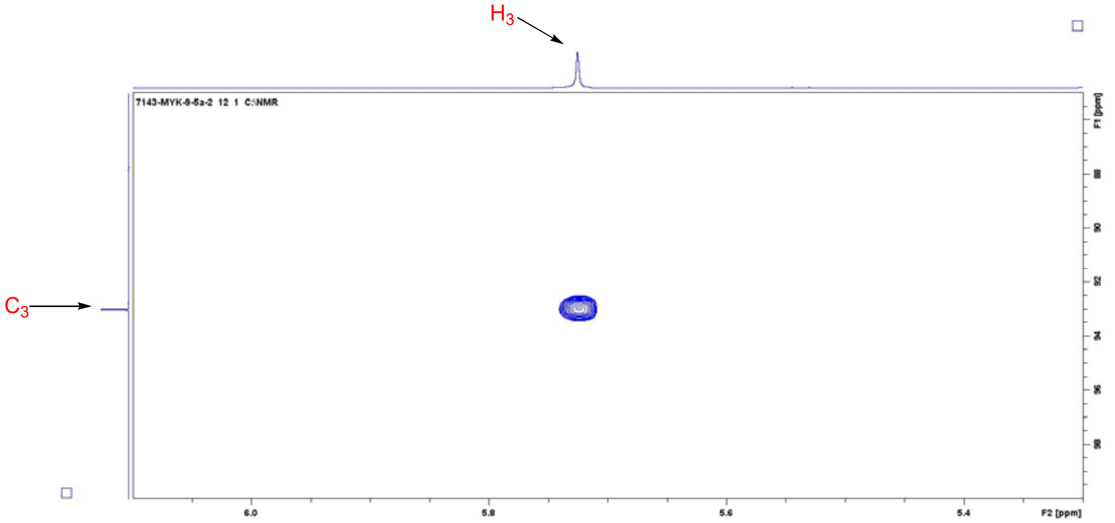

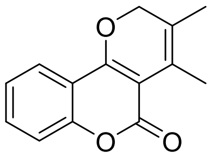

3,4-Dimethyl-2H,5H-pyrano[3,2-c]chromen-5-one (6a). Light yellow solid, **^{1}H NMR** (400 MHz, Chloroform-*d*) δ 7.73 (dd, *J* = 7.9, 1.5 Hz, 1H), 7.48 (ddd, *J* = 8.6, 7.4, 1.5 Hz, 1H), 7.28 – 7.20 (m, 2H), 4.75 (s, 2H), 2.16 (s, 3H), 1.80 (s, 3H). **^{13}C NMR** (101 MHz, $CDCl_3$) δ 160.24, 159.93, 152.90, 131.67, 123.74, 122.64, 122.53, 121.66, 116.21, 115.16, 105.14, 70.69, 15.65, 14.45. **HRMS** Calculated for $C_{14}H_{12}O_3$ $[M+H]^+$ 229.0865, found 229.0861.

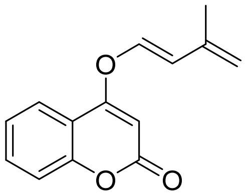

(*E*)-4-((3-Methylbuta-1,3-dien-1-yl)oxy)-2H-chromen-2-one (7a). An inseparable mixture of **3a** and **17**. NMR date for product **7a** was provided. **^{1}H NMR** (700 MHz, Chloroform-*d*) δ 7.90 (dd, *J* = 7.9, 1.3 Hz, 1H), 7.56 – 7.53 (m, 1H), 7.35 – 7.31 (m, 2H), 6.83 (d, *J* = 15.8

Hz, 1H), 6.00 (d, *J* = 15.8 Hz, 1H), 5.22 (s, 1H), 5.16 (s, 1H), 5.00 (s, 1H), 1.94 (s, 3H). **^{13}C NMR** (175 MHz, $CDCl_3$) δ 160.98, 155.04, 152.51, 143.25, 141.05, 131.75, 131.73, 123.87, 122.91, 120.93, 118.93, 116.33, 115.90, 18.33.

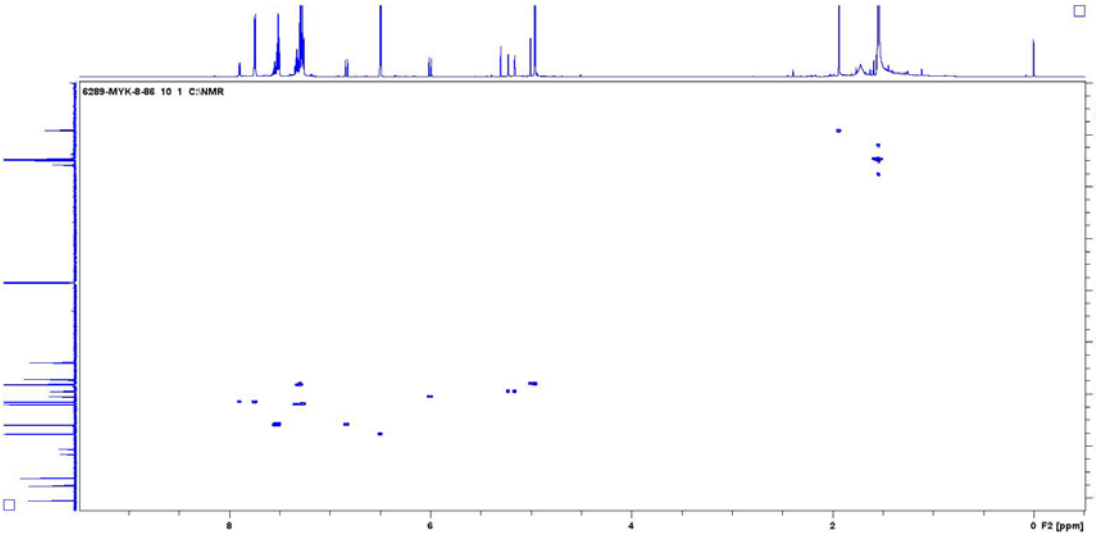

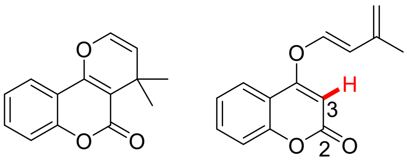

3a **7a**

HSQC (700 MHz, Chloroform-*d*)

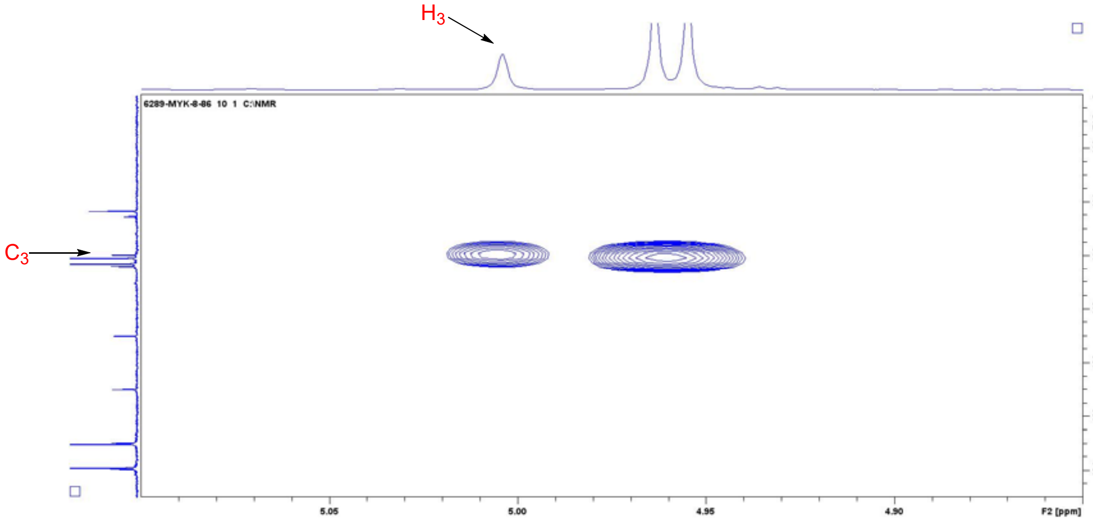

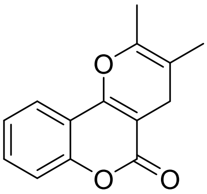

2,3-Dimethyl-4H,5H-pyrano[3,2-c]chromen-5-one (8a). Light yellow solid, **^{1}H NMR** (400 MHz, Chloroform-*d*) δ 7.75 (dd, *J* = 7.9, 1.4 Hz, 1H), 7.54 – 7.47 (m, 1H), 7.34 – 7.24 (m, 2H), 3.00 (s, 2H), 1.99 (s, 3H), 1.71 (s, 3H). **^{13}C NMR** (101 MHz, $CDCl_3$) δ 162.66, 156.41, 152.43, 140.55, 131.51, 123.90, 122.36, 116.62, 114.43, 105.52, 99.46, 25.24, 17.52, 15.17. **HRMS** Calculated for $C_{14}H_{12}O_3$ $[M+H]^+$ 229.0865, found 229.0865.

4.3. General procedure C: Rh-catalyzed annulation of 1,3-Enynes with pyrazol-5-ones.

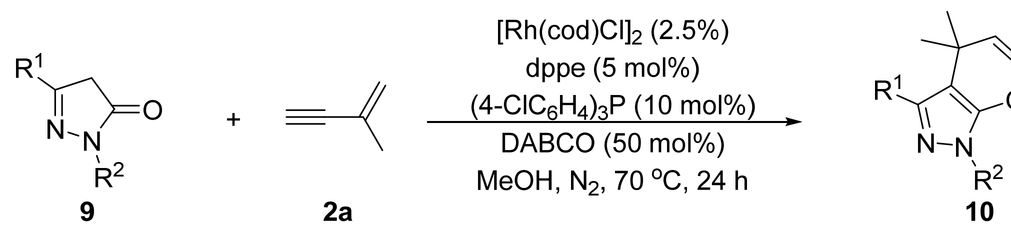

In a glove box, **9** (0.20 mmol), $[Rh(cod)Cl]_2$ (0.005 mmol, 2.5 mol%), dppe (0.01 mmol, 5 mol%), $(4\text{-}ClC_6H_4)_3P$ (0.02 mmol, 10 mol%), DABCO (0.10 mmol, 50 mol%), MeOH (0.5 mL) were sequentially added to a vial (4.0 mL) with a stir bar at room temperature. 1,3-enynes **2a** was then added to the mixture via syringe. The reaction vial was sealed with a cap, removed from the glove box. Then, the reaction mixture was stirred at 70 ºC for 24 h. After the starting material was consumed completely (monitored by TLC), the crude reaction mixture was purified by flash column chromatography on silica gel using petroleum and ethyl acetate (PE:EA = 20:1, v/v) to afford the desired products **10**.

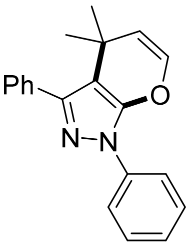

4,4-Dimethyl-1,3-diphenyl-1,4-dihydropyrano[2,3-c]pyrazole (10a). Light yellow solid (m.p. 101 °C), 51.6 mg, 85% yield. **^{1}H NMR** (400 MHz, Chloroform-*d*) δ 7.82 (dd, *J* = 8.7, 1.1 Hz, 2H), 7.59 (dd, *J* = 7.9, 1.6 Hz, 2H), 7.46 – 7.36 (m, 5H), 7.25 (t, *J* = 7.4 Hz, 1H), 6.46 (d, *J* = 6.1 Hz, 1H), 4.81 (d, *J* = 6.1 Hz, 1H), 1.30 (s, 6H). **^{13}C NMR** (101 MHz, $CDCl_3$) δ 149.89, 145.40, 138.31, 136.37, 135.11, 129.55, 128.99, 128.18, 128.11, 126.20, 121.10, 115.20, 104.79, 32.23, 31.35. **HRMS** Calculated for $C_{20}H_{19}N_2O$ $[M+H]^+$ 303.1497, found 303.1500.

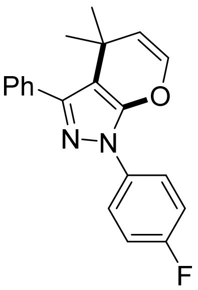

1-(4-Fluorophenyl)-4,4-dimethyl-3-phenyl-1,4-dihydropyrano[2,3-c] pyrazole (10b). Light yellow solid (m.p. 109 °C), 54.3 mg, 85% yield. **^{1}H NMR** (400 MHz, Chloroform-*d*) δ 7.79 (dd, *J* = 9.1, 4.8 Hz, 2H), 7.60 (dd, *J* = 7.8, 1.7 Hz, 2H), 7.46 – 7.38 (m, 3H), 7.16 – 7.08 (m, 2H), 6.48 (d, *J* = 6.1 Hz, 1H), 4.83 (d, *J* = 6.1 Hz, 1H), 1.31 (s, 6H). **^{13}C NMR** (101 MHz, $CDCl_3$) δ 160.85 (d, *J* = 245.6 Hz), 149.93, 145.26, 136.31, 134.47 (d, *J* = 2.9 Hz), 128.82 (d, *J* = 136.7 Hz), 128.24, 122.82 (d, *J* = 8.3 Hz), 115.77 (d, *J* = 22.8 Hz), 115.27, 104.75, 32.18, 31.37. **^{19}F NMR** (376 MHz, $CDCl_3$) δ -116.08. **HRMS** Calculated for $C_{20}H_{18}FN_2O$ $[M+H]^+$ 321.1403, found 321.1403.

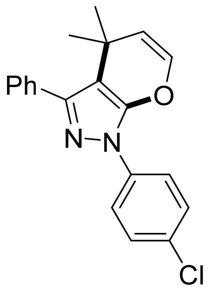

1-(4-Chlorophenyl)-4,4-dimethyl-3-phenyl-1,4-dihydropyrano[2,3-c]pyrazole (10c). Light yellow solid (m.p. 128 °C), 53.7 mg, 80% yield. **^{1}H NMR** (400 MHz, Chloroform-*d*) δ 7.80 (d, *J* = 9.0 Hz, 2H), 7.59 (dd, *J* = 7.7, 1.7 Hz, 2H), 7.47 – 7.37 (m, 5H), 6.48 (d, *J* = 6.1 Hz, 1H), 4.84 (d, *J* = 6.1 Hz, 1H), 1.31 (s, 6H). **^{13}C NMR** (101 MHz, $CDCl_3$) δ 150.22, 145.43, 136.92, 136.29, 134.85, 131.49, 129.48, 129.07, 128.31, 128.16, 121.99, 115.29, 105.04, 32.16, 31.33. **HRMS** Calculated for $C_{20}H_{18}ClN_2O$ [M+H]$^+$ 337.1108, found 337.1102.

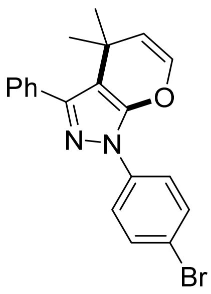

1-(4-Bromophenyl)-4,4-dimethyl-3-phenyl-1,4-dihydropyrano[2,3-c]pyrazole (10d). Light yellow solid (m.p. 137 °C), 57.7 mg, 76% yield. **^{1}H NMR** (400 MHz, Chloroform-*d*) δ 7.73 (d, *J* = 8.9 Hz, 2H), 7.57 (dd, *J* = 7.7, 1.8 Hz, 2H), 7.53 (d, *J* = 9.0 Hz, 2H), 7.41 (d, *J* = 7.4 Hz, 3H), 6.47 (d, *J* = 6.1 Hz, 1H), 4.82 (d, *J* = 6.1 Hz, 1H), 1.29 (s, 6H). **^{13}C NMR** (101 MHz, $CDCl_3$) δ 150.27, 145.45, 137.42, 136.29, 134.83, 132.02, 129.47, 128.33, 128.16, 122.26, 119.33, 115.29, 105.10, 32.16, 31.33. **HRMS** Calculated for $C_{20}H_{18}BrN_2O$ [M+H]$^+$ 381.0603, found 381.0608.

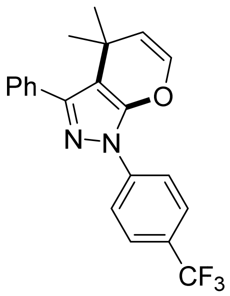

4,4-Dimethyl-3-phenyl-1-(4-(trifluoromethyl)phenyl)-1,4-dihydropyrano[2,3-c]pyrazole (10e). Light yellow solid (m.p. 94 °C), 61.0 mg, 82% yield. **^{1}H NMR** (400 MHz, Chloroform-*d*) δ 8.02 (d, *J* = 8.6 Hz, 2H), 7.69 (d, *J* = 8.7 Hz, 2H), 7.60 (dd, *J* = 7.6, 1.9 Hz, 2H), 7.44 (d, *J* = 7.2 Hz, 3H), 6.51 (d, *J* = 6.1 Hz, 1H), 4.86 (d, *J* = 6.1 Hz, 1H), 1.32 (s, 6H). **^{13}C NMR** (101 MHz, $CDCl_3$) δ 150.89, 145.83, 141.12, 136.27, 134.66, 129.46, 128.46, 128.21, 127.66 (q, *J* = 32.8 Hz), 126.22 (q, *J* = 3.7 Hz), 124.09 (q, *J* = 271.7 Hz), 120.31, 115.37, 105.48, 32.10, 31.31. **^{19}F NMR** (376 MHz, $CDCl_3$) δ -62.20. **HRMS** Calculated for $C_{21}H_{18}F_3N_2O$ [M+H]$^+$ 371.1371, found 371.1387.

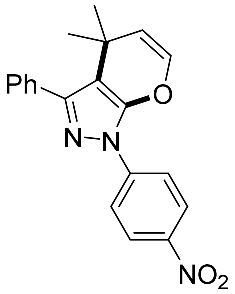

4,4-Dimethyl-1-(4-nitrophenyl)-3-phenyl-1,4-dihydropyrano[2,3-c]pyrazole (10f). Light yellow solid (m.p. 125 °C), 48.6 mg, 70% yield. **^{1}H NMR** (400 MHz, Chloroform-*d*) δ 8.29 (d, *J* = 9.3 Hz, 2H), 8.08 (d, *J* = 9.3 Hz, 2H), 7.62 – 7.55 (m, 2H), 7.44 (m, 3H), 6.53 (d, *J* = 6.0 Hz, 1H), 4.88 (d, *J* = 6.0 Hz, 1H), 1.30 (s, 6H). **^{13}C NMR** (101 MHz, $CDCl_3$) δ 151.84, 146.22, 144.83, 143.36, 136.23, 134.28, 129.37, 128.68, 128.27, 124.86, 119.85, 115.48, 106.08, 32.03, 31.30. **HRMS** Calculated for $C_{20}H_{18}N_3O_3$ [M+H]$^+$ 348.1348, found 348.1355.

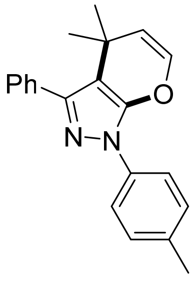

4,4-Dimethyl-3-phenyl-1-(p-tolyl)-1,4-dihydropyrano[2,3-c]pyrazole (10g). Light yellow solid (m.p. 129 °C), 55.4 mg, 88% yield. **^{1}H NMR** (400 MHz, Chloroform-*d*) δ 7.67 (d, *J* = 8.5 Hz, 2H), 7.59 (dd, *J* = 7.9, 1.6 Hz, 2H), 7.45 – 7.34 (m, 3H), 7.22 (d, *J* = 8.2 Hz, 2H), 6.45 (d, *J* = 6.1 Hz, 1H), 4.79 (d, *J* = 6.1 Hz, 1H), 2.36 (s, 3H), 1.30 (s, 6H). **^{13}C NMR** (101 MHz, $CDCl_3$) δ 149.56, 145.26, 136.39, 136.00, 135.88, 135.21, 129.57, 128.09, 121.17, 115.16, 104.57, 32.25, 31.36, 21.04. **HRMS** Calculated for $C_{21}H_{21}N_2O$ $[M+H]^+$ 317.1654, found 317.1650.

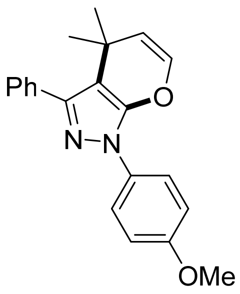

1-(4-Methoxyphenyl)-4,4-dimethyl-3-phenyl-1,4-dihydropyrano[2,3-c]pyrazole (10h). Light yellow solid (m.p. 150 °C), 36.8 mg, 55% yield. **^{1}H NMR** (400 MHz, Chloroform-*d*) δ 7.70 (d, *J* = 9.0 Hz, 2H), 7.61 (d, *J* = 7.9 Hz, 2H), 7.47 – 7.37 (m, 3H), 6.96 (d, *J* = 9.0 Hz, 2H), 6.46 (d, *J* = 6.1 Hz, 1H), 4.81 (d, *J* = 6.1 Hz, 1H), 3.83 (s, 3H), 1.31 (s, 3H). **^{13}C NMR** (101 MHz, $CDCl_3$) δ 158.02, 149.35, 145.10, 136.38, 135.21, 131.58, 129.54, 128.07, 122.92, 115.18, 114.14, 104.36, 55.51, 32.24, 31.39. **HRMS** Calculated for $C_{21}H_{21}N_2O_2$ $[M+H]^+$ 333.1603, found 333.1599.

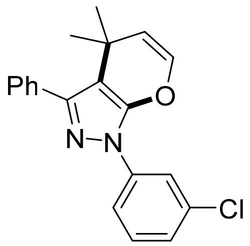

1-(3-Chlorophenyl)-4,4-dimethyl-3-phenyl-1,4-dihydropyrano[2,3-c]pyrazole (10i). Light yellow solid (m.p. 90 °C), 60.8 mg, 90% yield. **^{1}H NMR** (400 MHz, Chloroform-*d*) δ 7.90 (t, *J* = 2.0 Hz, 1H), 7.75 (ddd, *J* = 8.2, 2.0, 0.9 Hz, 1H), 7.58 (dd, *J* = 7.8, 1.8 Hz, 2H), 7.47 – 7.38 (m, 3H), 7.33 (t, *J* = 8.1 Hz, 1H), 7.21 (ddd, *J* = 8.0, 2.0, 1.0 Hz, 1H), 6.48 (d, *J* = 6.1 Hz, 1H), 4.82 (d, *J* = 6.1 Hz, 1H), 1.29 (s, 6H). **^{13}C NMR** (101 MHz, $CDCl_3$) δ 150.43, 145.58, 139.36, 136.31, 134.78, 134.74, 130.01, 129.49, 128.37, 128.18, 126.02, 120.84, 118.65, 115.28, 105.18, 32.15, 31.31. **HRMS** Calculated for $C_{20}H_{18}ClN_2O$ $[M+H]^+$ 337.1108, found 337.1105.

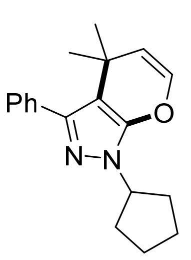

1-Cyclopentyl-4,4-dimethyl-3-phenyl-1,4-dihydropyrano[2,3-c]pyrazole (10j). Light yellow solid (m.p. 84 °C), 41.0 mg, 70% yield. **^{1}H NMR** (400 MHz, Chloroform-*d*) δ 7.54 (dd, *J* = 8.1, 1.5 Hz, 2H), 7.36 (m, 3H), 6.40 (d, *J* = 6.1 Hz, 1H), 4.74 (d, *J* = 6.1 Hz, 1H), 4.64 (p, *J* = 7.9 Hz, 1H), 2.20 – 2.02 (m, 4H), 1.97 – 1.85 (m, 2H), 1.71 – 1.58 (m, 2H), 1.27 (s, 6H). **^{13}C NMR** (101 MHz, $CDCl_3$) δ 147.32, 145.08, 136.22, 135.91, 129.46, 127.98, 127.60, 115.19, 102.85, 58.14, 32.29, 31.75, 31.32, 24.41. **HRMS** Calculated for $C_{19}H_{23}N_2O$ $[M+H]^+$ 295.1810, found 295.1815.

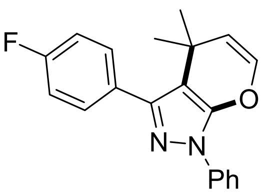

3-(4-Fluorophenyl)-4,4-dimethyl-1-phenyl-1,4-dihydropyrano[2,3-c]pyrazole (10k). Light yellow solid (m.p. 117 °C), 52.6 mg, 82% yield. **^{1}H NMR** (400 MHz, Chloroform-*d*) δ 7.80 (dd, *J* = 8.6, 1.0 Hz, 2H), 7.56 (dd, *J* = 8.7, 5.5 Hz, 2H), 7.42 (t, *J* = 8.0 Hz, 2H), 7.26 (t, *J* = 7.4 Hz, 1H), 7.11 (t, *J* = 8.7 Hz, 2H), 6.47 (d, *J* = 6.1 Hz, 1H), 4.81 (d, *J* = 6.1 Hz, 1H), 1.29 (s, 6H). **^{13}C NMR** (101 MHz, $CDCl_3$) δ 162.83 (d, *J* = 247.1 Hz), 148.84, 145.43, 138.20, 136.41, 131.29 (d, *J* = 8.2 Hz), 131.15 (d, *J* = 3.2 Hz), 129.02, 126.31, 121.11, 115.10 (d, *J* = 21.3 Hz), 115.09, 104.83, 32.21, 31.29. **^{19}F NMR** (376 MHz, $CDCl_3$) δ -113.83. **HRMS** Calculated for $C_{20}H_{18}FN_2O$ $[M+H]^+$ 321.1403, found 321.1399.

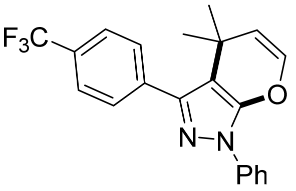

4,4-Dimethyl-1-phenyl-3-(4-(trifluoromethyl)phenyl)-1,4-dihydropyrano[2,3-c]pyrazole (10l). Light yellow solid (m.p. 131 °C), 65.5 mg, 88% yield. **^{1}H NMR** (400 MHz, Chloroform-*d*) δ 7.80 (d, *J* = 7.8 Hz, 2H), 7.75 (d, *J* = 8.1 Hz, 2H), 7.69 (d, *J* = 8.2 Hz, 2H), 7.44 (t, *J* = 7.9 Hz, 2H), 7.27 (t, *J* = 7.4 Hz, 1H), 6.48 (d, *J* = 6.1 Hz, 1H), 4.83 (d, *J* = 6.1 Hz, 1H), 1.31 (s, 6H). **^{13}C NMR** (101 MHz, $CDCl_3$) δ 148.26, 145.64, 138.85, 138.10, 136.39, 130.25 (q, *J* = 32.1 Hz), 129.82, 129.06, 124.23 (q, *J* = 3.7 Hz), 126.52, 125.11 (q, *J* = 3.7 Hz), 121.17, 115.05, 104.95, 32.28, 31.33. **^{19}F NMR** (376 MHz, $CDCl_3$) δ -62.51. **HRMS** Calculated for $C_{21}H_{18}F_3N_2O$ $[M+H]^+$ 371.1371, found 371.1387.

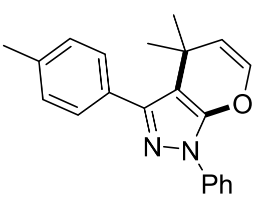

4,4-Dimethyl-1-phenyl-3-(p-tolyl)-1,4-dihydropyrano[2,3-c]pyrazole (10m). Light yellow solid (m.p. 134 °C), 51.8 mg, 82% yield. **^{1}H NMR** (400 MHz, Chloroform-*d*) δ 7.81 (dd, *J* = 8.6, 1.1 Hz, 2H), 7.48 (d, *J* = 8.1 Hz, 2H), 7.45 – 7.38 (m, 2H), 7.26 – 7.19 (m, 3H), 6.46 (d, *J* = 6.1 Hz, 1H), 4.80 (d, *J* = 6.1 Hz, 1H), 2.39 (s, 3H), 1.30 (s, 6H). **^{13}C NMR** (101 MHz, $CDCl_3$) δ 149.91, 145.36, 138.37, 137.88, 136.35, 132.17, 129.39, 128.96, 128.81, 126.10, 121.05, 115.22, 104.72, 32.22, 31.35, 21.36. **HRMS** Calculated for $C_{21}H_{21}N_2O$ $[M+H]^+$ 317.1654, found 317.1673.

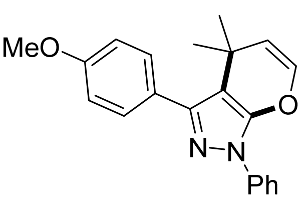

3-(4-Methoxyphenyl)-4,4-dimethyl-1-phenyl-1,4-dihydropyrano[2,3-c]pyrazole (10n). Light yellow solid (m.p. 120 °C), 51.5 mg, 78% yield. **^{1}H NMR** (400 MHz, Chloroform-*d*) δ 7.81 (dd, *J* = 8.7, 1.1 Hz, 2H), 7.52 (d, *J* = 8.8 Hz, 2H), 7.47 – 7.37 (dd, *J* = 8.2, 7.5 Hz, 2H), 7.24 (t, *J* = 7.4 Hz, 1H), 6.95 (d, *J* = 8.8 Hz, 2H), 6.46 (d, *J* = 6.1 Hz, 1H), 4.80 (d, *J* = 6.1 Hz, 1H), 3.83 (s, 3H), 1.30 (s, 6H).

^{13}C NMR (101 MHz, $CDCl_3$) δ 159.60, 149.62, 145.35, 138.35, 136.36, 130.70, 128.96, 127.49, 126.09, 121.04, 115.20, 113.55, 104.71, 55.30, 32.19, 31.32. **HRMS** Calculated for $C_{21}H_{21}N_2O_2$ $[M+H]^+$ 333.1603, found 333.1609.

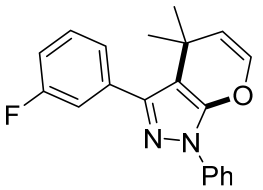

3-(3-Fluorophenyl)-4,4-dimethyl-1-phenyl-1,4-dihydropyrano[2,3-c]pyrazole (10o). Light yellow solid (m.p. 118 °C), 56.8 mg, 89% yield. **^{1}H NMR** (400 MHz, Chloroform-*d*) δ 7.80 (d, *J* = 7.8 Hz, 2H), 7.47 – 7.31 (m, 5H), 7.26 (t, *J* = 7.4 Hz, 1H), 7.12 – 7.05 (m, 1H), 6.47 (d, *J* = 6.1 Hz, 1H), 4.82 (d, *J* = 6.1 Hz, 1H), 1.32 (s, 6H). **^{13}C NMR** (101 MHz, $CDCl_3$) δ 162.50 (d, *J* = 245.9 Hz), 148.46, 145.51, 138.16, 137.24 (d, *J* = 8.1 Hz), 136.36, 129.66 (d, *J* = 8.4 Hz), 129.03, 126.41, 125.28 (d, *J* = 2.9 Hz), 121.16, 116.57 (d, *J* = 22.0 Hz), 115.12, 115.11 (d, *J* = 20.9 Hz), 104.77, 32.22, 31.33. **^{19}F NMR** (376 MHz, $CDCl_3$) δ -113.32. **HRMS** Calculated for $C_{20}H_{18}FN_2O$ $[M+H]^+$ 321.1403, found 321.1409.

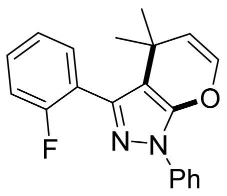

3-(2-Fluorophenyl)-4,4-dimethyl-1-phenyl-1,4-dihydropyrano[2,3-c]pyrazole (10p). Light yellow solid (m.p. 104 °C), 29.1 mg, 45% yield. **^{1}H NMR** (400 MHz, Chloroform-*d*) δ 7.80 (dd, *J* = 8.7, 1.1 Hz, 2H), 7.46 – 7.36 (m, 4H), 7.26 (t, *J* = 7.4 Hz, 1H), 7.19 (td, *J* = 7.5, 1.1 Hz, 1H), 7.14 (t, *J* = 8.5 Hz, 1H), 6.47 (d, *J* = 6.1 Hz, 1H), 4.81 (d, *J* = 6.1 Hz, 1H), 1.21 (s, 6H). **^{13}C NMR** (101 MHz, $CDCl_3$) δ 160.60 (d, *J* = 247.5 Hz), 145.30, 143.72, 138.20, 136.61, 132.53 (d, *J* = 2.8 Hz), 130.50 (d, *J* = 8.0 Hz), 128.97, 126.31, 123.75 (d, *J* = 3.7 Hz), 122.78 (d, *J* = 16.3 Hz), 121.11, 115.70 (d, *J* = 21.9 Hz), 114.96, 105.95, 31.60, 31.07. **^{19}F NMR** (376 MHz, $CDCl_3$) δ -112.42. **HRMS** Calculated for $C_{20}H_{18}FN_2O$ $[M+H]^+$ 321.1403, found 321.1410.

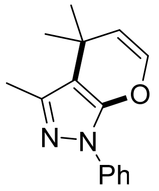

3,4,4-Trimethyl-1-phenyl-1,4-dihydropyrano[2,3-c]pyrazole (10q). Light yellow solid (m.p. 78 °C), 42.1 mg, 88% yield. **^{1}H NMR** (400 MHz, Chloroform-*d*) δ 7.71 (dd, *J* = 8.7, 1.1 Hz, 2H), 7.44 – 7.36 (dd, *J* = 7.5, 8.2 Hz, 2H), 7.21 (t, *J* = 7.4 Hz, 1H), 6.43 (d, *J* = 6.1 Hz, 1H), 4.82 (d, *J* = 6.1 Hz, 1H), 2.34 (s, 3H), 1.37 (s, 6H). **^{13}C NMR** (101 MHz, $CDCl_3$) δ 145.65, 145.34, 138.38, 128.97, 125.76, 120.65, 114.64, 103.72, 31.15, 30.65, 14.37. **HRMS** Calculated for $C_{15}H_{17}N_2O$ $[M+H]^+$ 241.1341, found 241.1335.

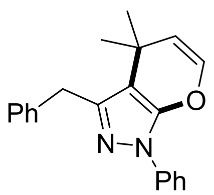

3-Benzyl-4,4-dimethyl-1-phenyl-1,4-dihydropyrano[2,3-c]pyrazole (10r). Light yellow oil, 49.9 mg, 79% yield. **^{1}H NMR** (400 MHz, Chloroform-*d*) δ 7.76 (dd, *J* = 8.7, 1.1 Hz, 2H), 7.42 (ddd, *J* = 8.5, 5.7, 1.8 Hz, 2H), 7.33 – 7.15 (m, 6H), 6.40 (d, *J* = 6.1 Hz, 1H), 4.75 (d, *J* =

6.1 Hz, 1H), 4.07 (s, 2H), 1.17 (s, 6H). **^{13}C NMR** (101 MHz, $CDCl_3$) δ 147.96, 145.58, 139.28, 138.44, 136.39, 129.01, 128.73, 128.31, 126.20, 125.94, 120.92, 114.93, 103.74, 34.96, 31.56, 30.88. **HRMS** Calculated for $C_{21}H_{21}N_2O$ [M+H]$^+$ 317.1654, found 317.1654.

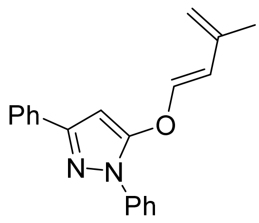

(*E*)-4-(3-Methylbuta-1,3-dien-1-yl)-1,3-diphenyl-1H-pyrazol-5-ol (11a). Light yellow solid, An inseparable mixture of **10a** and **11a.** Characteristic ^{1}H NMR signal for product **11a** was provided. **^{1}H NMR** (400 MHz, Chloroform-*d*) δ 6.73 (d, *J* = 15.7 Hz, 1H), 6.05 (d, *J* = 15.8 Hz, 1H), 5.12 (s, 2H), 4.91 (s, 1H), 1.92 (s, 3H).

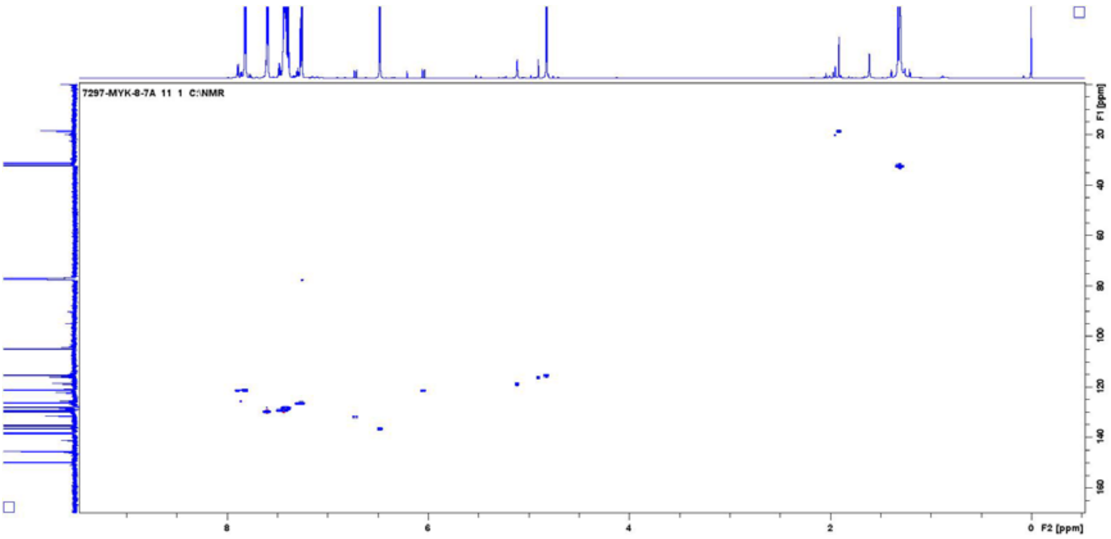

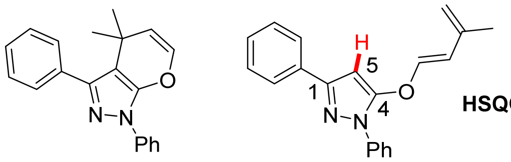

HSQC (700 MHz, Chloroform-*d*)

10a : 11a = 10.5 : 1

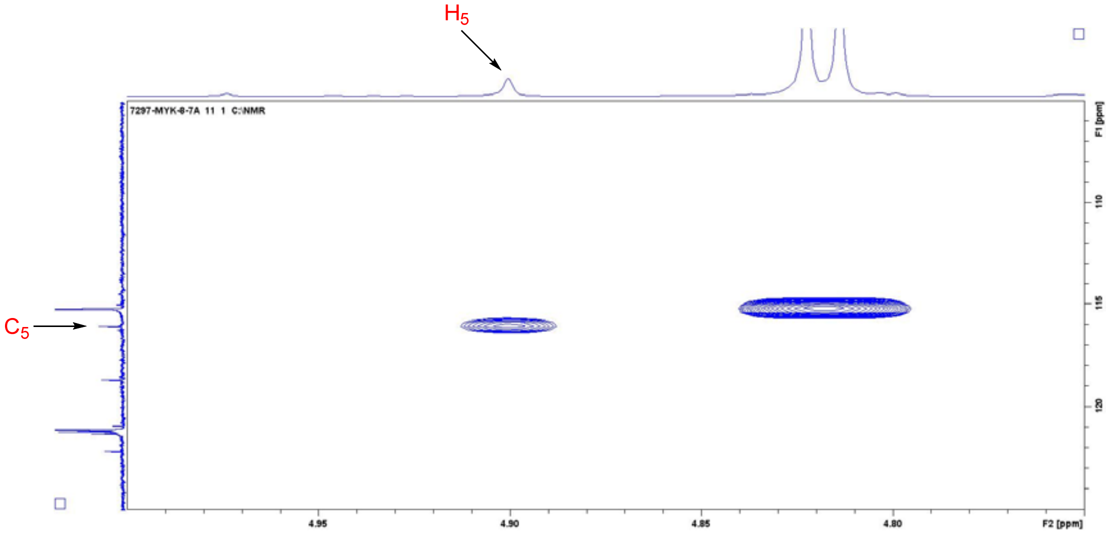

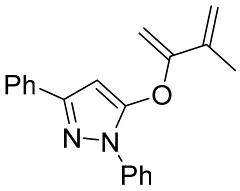

5-((3-Methylbuta-1,3-dien-2-yl)oxy)-1,3-diphenyl-1H-pyrazole (12a). Light yellow solid, **^{1}H NMR** (400 MHz, Chloroform-*d*) δ 7.91 – 7.86 (m, 2H), 7.82 – 7.78 (m, 2H), 7.49 – 7.40 (m, 4H), 7.37 – 7.29 (m, 2H), 6.23 (s, 1H), 5.55 (s, 1H), 5.13 (s, 1H), 4.82 (d, *J* = 2.9 Hz, 1H), 4.77 (d, *J* = 2.0 Hz, 1H), 1.97 (s, 3H). **^{13}C NMR** (101 MHz, $CDCl_3$) δ 158.83, 151.20, 150.66, 138.54, 135.93, 133.28, 129.01, 128.60, 128.15, 126.73, 125.50, 122.17, 115.00, 94.83, 90.26, 19.77. **HRMS** Calculated for $C_{20}H_{19}N_2O$ $[M+H]^+$ 303.1497, found 303.1500.

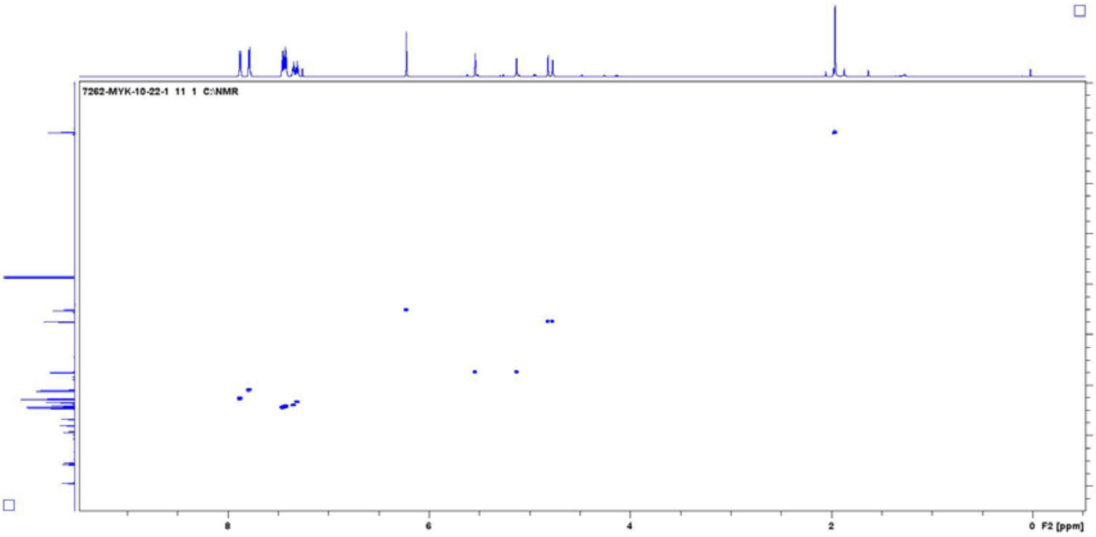

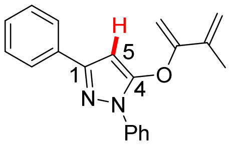

12a

HSQC (700 MHz, Chloroform-*d*)

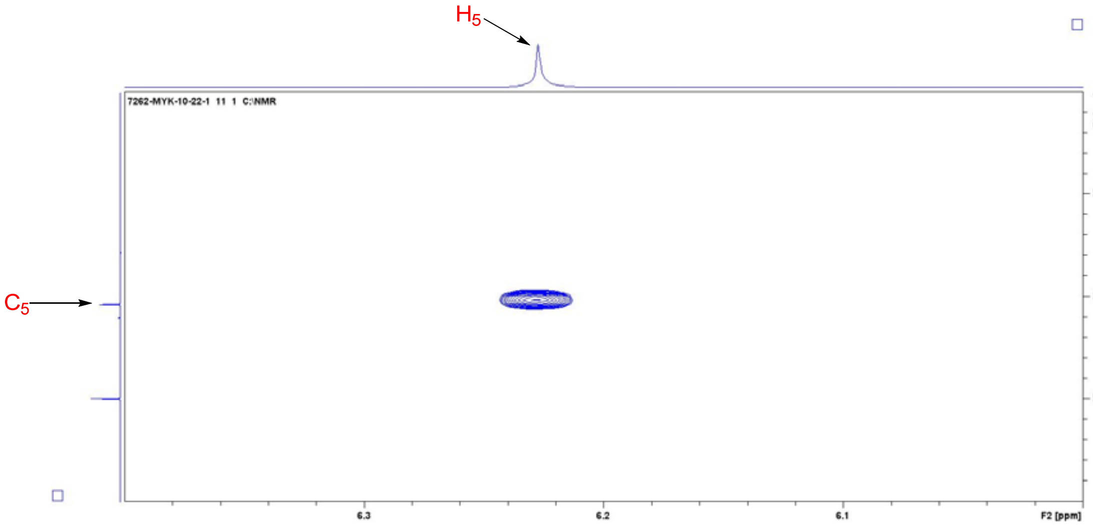

5. Mechanistic studies

5.1. The synthesis and transformation of intermediate 13

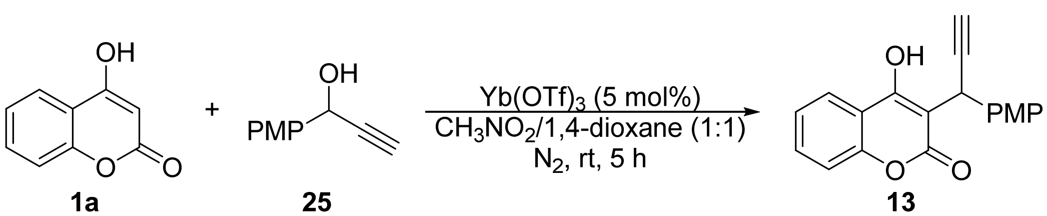

To an oven-dried 15 mL vial was sequentially added **1a** (5.0 mmol, 1.0 equiv.), **25** (10 mmol, 2.0 equiv.), $Yb(OTf)_3$ (0.25 mmol, 5 mol%), CH_3NO_2 (10 mL), and 1,4-dioxane (10 mL) with a stir bar in the glovebox. Then the reaction vial was sealed with a cap, removed from the glove box. The reaction mixture was stirred at room temperature for 5 h. After the reaction completed, the reaction mixture was quenched with H_2O and extracted with ethyl acetate (3 × 30 mL). The combined organic layers were dried over anhydrous Na_2SO_4, filtered, and concentrated under reduced pressure. the crude product was purified by flash column chromatography on silica gel using petroleum ether and ethyl acetate to afford the corresponding product **11** (865 mg, 63% yield) as a pale yellow solid.

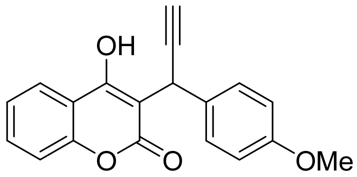

4-Hydroxy-3-(1-(4-methoxyphenyl)prop-2-yn-1-yl)-2H-chromen-2-one (13).[9] Known compound, pale yellow solid. **^{1}H NMR** (400 MHz, Chloroform-*d*) δ 8.11 (s, 1H), 7.85 (d, *J* = 7.8 Hz, 1H), 7.57 – 7.49 (t, *J* = 7.8 Hz, 1H), 7.46 (d, *J* = 8.7 Hz, 2H), 7.33 – 7.23 (m, 2H), 6.87 (d, *J* = 8.7 Hz, 2H), 5.51 (d, *J* = 2.6 Hz, 1H), 3.74 (s, 3H), 2.77 (d, *J* = 2.7 Hz, 1H). **^{13}C NMR** (100 MHz, $CDCl_3$) δ 162.50, 160.87, 159.22, 152.62, 132.39, 129.68, 128.16, 124.10, 123.41, 116.50, 115.93, 114.41, 104.66, 82.60, 75.68, 55.32, 31.83.

Table S12. Transformation of intermediate 13

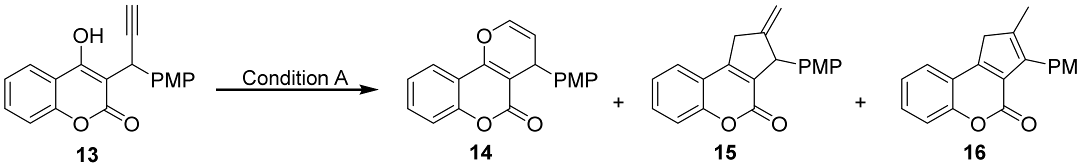

Entry	Variation from Condition A	Yield		
1	None	**14**, 9%	**15**, 4%	**16**, 13%
2	Without DABCO	**14**, 31%	**15**, 6%	**16**, NR
3	Without [Rh], Ligand	**14**, None	**15**, 11%	**16**, 11%
4	Condition B	**14**, trace	**15**, NR	**16**, 25%

To an oven-dried 4 mL vial was sequentially added **13** (0.20 mmol, 1.0 equiv.), $[Rh(cod)Cl]_2$ (0.005 mmol, 5 mol%), Ligand (BINAP, 0.01 mmol, 5 mol%; $(4\text{-}FC_6H_4)_3P$,

0.02 mmol, 10 mol%; or dppb, 0.01 mmol, 5 mol%), DABCO or 4-$MeOC_6H_4SO_3H$ (0.10 mmol, 50 mol%), and solvents (DCE, 0.2 mL, MeOH, 0.3mL; or DCM, 0.5 mL, DME, 0.4 mL) with a stir bar in the glovebox. Then the reaction vial was sealed with a cap, removed from the glovebox. Subsequently, the reaction mixture was stirred at 70 ^oC for 24 h. Upon completion (monitored by TLC), the crude product was purified by flash column chromatography on silica gel using petroleum ether and ethyl acetate to afford the corresponding products.

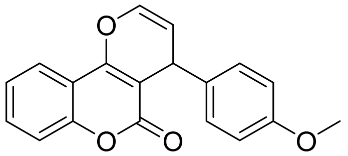

4-(4-Methoxyphenyl)-4H,5H-pyrano[3,2-c]chromen-5-one (14). An inseparable mixture of **14** and **15**. NMR date for product **14** was provided. Yellow solid. **^{1}H NMR** (400 MHz, Chloroform-*d*) δ 7.81 (dd, *J* = 7.9, 1.3 Hz, 1H), 7.52 (ddd, *J* = 8.9, 7.8, 1.6 Hz, 1H), 7.32 – 7.26 (m, 4H), 6.84 (d, *J* = 8.7 Hz, 2H), 6.77 (dd, *J* = 6.0, 1.2 Hz, 1H), 5.33 (dd, *J* = 6.0, 4.6 Hz, 1H), 4.48 (dd, *J* = 4.5, 1.2 Hz, 1H), 3.76 (s, 3H). **^{13}C NMR** (101 MHz, $CDCl_3$) δ 161.56, 158.69, 155.42, 152.53, 137.99, 135.86, 131.85, 129.48, 124.06, 122.71, 116.67, 114.38, 113.91, 109.02, 103.99, 34.42, 12.62.

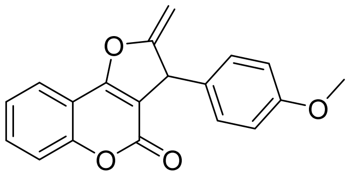

3-(4-Methoxyphenyl)-2-methylene-2,3-dihydro-4H-furo[3,2-c]chromen-4-one (15).[10] Known compound, yellow solid. **^{1}H NMR** (400 MHz, Chloroform-*d*) δ 7.78 (d, *J* = 7.8 Hz, 1H), 7.60 (t, *J* = 7.2 Hz, 1H), 7.42 – 7.31 (m, 2H), 7.25 (d, *J* = 8.9 Hz, 2H), 6.88 (d, *J* = 8.6 Hz, 2H), 5.12 (t, *J* = 2.2 Hz, 1H), 5.09 (t, *J* = 3.0 Hz, 1H), 4.50 (t, *J* = 2.4 Hz, 1H), 3.79 (s, 3H). **^{13}C NMR** (100 MHz, $CDCl_3$) δ 165.16, 164.19, 159.07, 158.56, 155.24, 132.83, 131.70, 128.85, 124.21, 122.83, 117.17, 114.33, 111.58, 106.61, 91.38, 55.28, 47.83. **HRMS** Calculated for $C_{19}H_{15}O_4$ $[M+H]^+$ 307.0970, found 307.0985.

5.2. The synthesis and transformation of intermediate 17

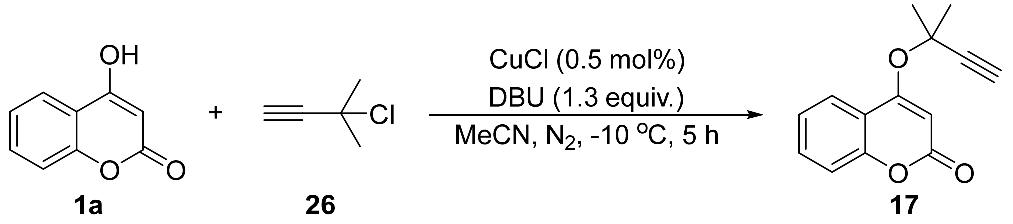

To a flame-dried Schlenk flask with a magnetic stir bar was sequentially added **1a** (6.0 mmol, 1.2 equiv.), CuCl (0.025 mmol, 0.5 mol%) and MeCN (5 mL) at room temperature under N_2 atmosphere. The reaction mixture was then cooled to -10 ^oC, treated with DBU

(1.3 equiv.) and stirred at -10 °C for 30 min. Subsequently, **26** (5.0 mmol, 1.0 equiv.) was added slowly to the above mixture, and the resulting mixture was maintained at -10 °C for 5 h. Upon reaction completion (monitored by TLC), the solution was allowed to warm to room temperature. After concentration under reduced pressure, the crude product was purified by flash column chromatography on silica gel using petroleum ether and ethyl acetate to afford the corresponding product **17** (490 mg, 43%) as light yellow solid.

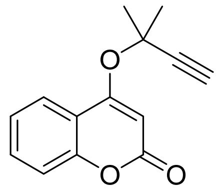

4-((2-Methylbut-3-yn-2-yl)oxy)-2H-chromen-2-one (17).[11] Known compound. light yellow solid. **^{1}H NMR** (400 MHz, Chloroform-*d*) δ 7.76 (dd, *J* = 8.0, 1.5 Hz, 1H), 7.55 – 7.49 (dt, *J* = 7.8, 1.6 Hz, 1H), 7.30 (d, *J* = 8.3 Hz, 1H), 7.28 – 7.22 (t, *J* = 7.6 Hz, 1H), 6.34 (s, 1H), 2.78 (s, 1H), 1.85 (s, 6H). **^{13}C NMR** (101 MHz, $CDCl_3$) δ 162.72, 161.92, 153.45, 132.12, 123.77, 123.18, 116.77, 116.50, 94.79, 82.42, 77.07, 74.17, 29.07.

Table S13. Transformation of intermediate 17

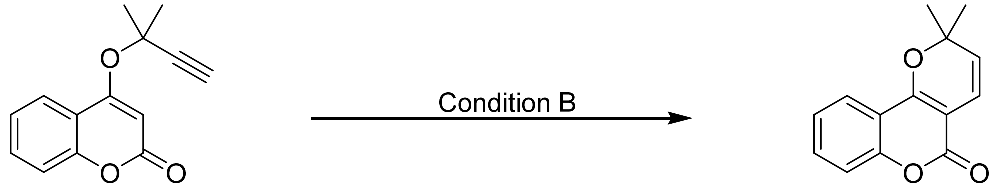

17 → **4a**

Entry	Variation from Condition B	Yield
1	None	**4a**, 98%
2	Without [Rh], Ligand	**4a**, 82%
3	Condition A	**3a**, 27%<br>**4a**, 21%
4	**1i,** Condition A	**3a**, 43%<br>**3i**, 37%

To an oven-dried 4 mL vial was sequentially added **17** (0.20 mmol, 1.0 equiv.), or the mixture of **17** (0.10 mmol) and **3i** (0.10 mmol), $[Rh(cod)Cl]_2$ (0.005 mmol, 5 mol%), Ligand (BINAP, 0.01 mmol, 5 mol%; $(4\text{-}FC_6H_4)_3P$, 0.02 mmol, 10 mol%; or dppb, 0.01 mmol, 5 mol%), DABCO or $4\text{-}MeOC_6H_4SO_3H$ (0.10 mmol, 50 mol%), and solvents (DCE, 0.2 mL, MeOH, 0.3mL; or DCM, 0.5 mL, DME, 0.4 mL) with a stir bar in the glovebox. The reaction vial was sealed with a cap, removed from the glovebox. Then, the reaction mixture was stirred at 70 °C for 24 h. After the reaction completed (monitored by TLC), the crude reaction mixture was purified by flash column chromatography on silica gel using

petroleum and ethyl acetate to afford the desired products.

5.3 Transformation of intermediate 5a.

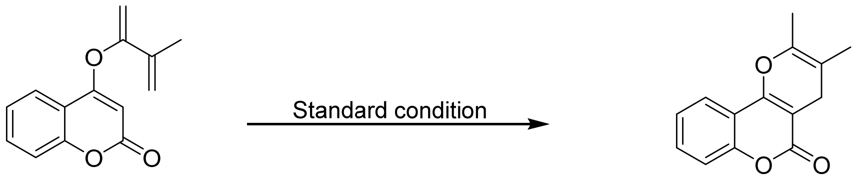

5a **8a**

Entry	Standard condition	**8a**
1	Condition A	ND
2	Condition B	ND

Condition A: To an oven-dried 4 mL vial was sequentially added **5a** (0.10 mmol, 1.0 equiv.), $[Rh(cod)Cl]_2$ (0.0025 mmol, 2.5 mol%), BINAP (0.005 mmol, 5 mol%), $(4\text{-}FC_6H_4)_3P$ (0.01 mmol, 10 mol%), DABCO (0.05 mmol, 50 mol%), MeOH (0.15 mL), and DCE (0.10 mL) with a stir bar in the glovebox. The reaction vial was sealed with a cap, removed from the glovebox. Then, the reaction mixture was stirred at 70 ºC for 24 h. After the reaction completed (monitored by TLC), the crude reaction mixture was purified by flash column chromatography on silica gel using petroleum and ethyl acetate to afford the desired products.

Condition B: To an oven-dried 4 mL vial was sequentially added the mixture of **5a** (0.10 mmol, 1.0 equiv.), $[Rh(cod)Cl]_2$ (0.0025 mmol, 2.5 mol%), dppb (0.005 mmol, 5 mol%), $4\text{-}MeOC_6H_4SO_3H$ (0.05 mmol, 50 mol%), DME (0.20 mL), and DCM (0.25 mL) with a stir bar in the glove box. The reaction vial was sealed with a cap, removed from the glovebox. Then, the reaction mixture was stirred at 70 ºC for 24 h. After the reaction completed (monitored by TLC), the crude reaction mixture was purified by flash column chromatography on silica gel using petroleum and ethyl acetate to afford the desired products.

5.4. Isotopic labeling experiments

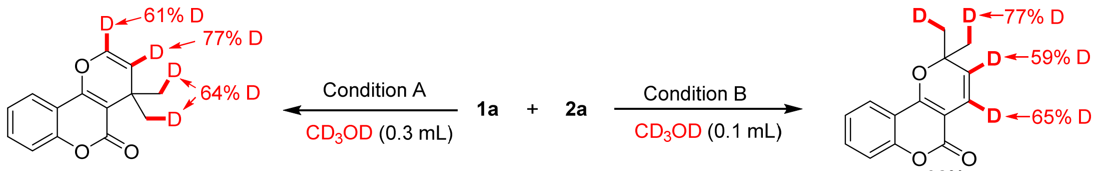

d-3a 48% **d-4a 62%**

Condition A: To an oven-dried 4 mL vial was sequentially added **1a** (0.20 mmol, 1.0 equiv.), $[Rh(cod)Cl]_2$ (0.005 mmol, 2.5 mol%), BINAP (0.01 mmol, 5 mol%), $(4\text{-}FC_6H_4)_3P$ (0.02 mmol, 10 mol%), DABCO (0.10 mmol, 50 mol%), CD_3OD (0.3 mL), and DCE (0.2 mL) with a stir bar in the glove box. Then 2-methylbut-1-en-3-yne **2a** (4.0 mmol, 2.0 equiv.) was added to the above mixture using a syringe at room temperature. The reaction vial was sealed with a cap, removed from the glovebox. Then, the reaction mixture was stirred at 70 °C for 24 h. After the reaction completed (monitored by TLC), the crude reaction mixture was purified by flash column chromatography on silica gel using petroleum and ethyl acetate to afford the desired product *d*-**3a** (22.0 mg, 48% yield) as light yellow oil.

Condition B: To an oven-dried 4 mL vial was sequentially added **1a** (0.20 mmol, 1.0 equiv.), $[Rh(cod)Cl]_2$ (0.005 mmol, 2.5 mol%), dppb (0.01 mmol, 5 mol%), $4\text{-}MeOC_6H_4SO_3H$ (0.1 mmol, 50 mol%), DME (0.4 mL), DCM (0.5 mL) and CD_3OD (0.1 mL) with a stir bar in the glove box. Then 2-methylbut-1-en-3-yne **2a** (4.0 mmol, 2.0 equiv.) was added to the above mixture using a syringe at room temperature. The reaction vial was sealed with a cap, removed from the glovebox. Then, the reaction mixture was stirred at 70 °C for 24 h. After the reaction completed (monitored by TLC), the crude reaction mixture was purified by flash column chromatography on silica gel using petroleum and ethyl acetate to afford the desired product *d*-**4a** (28.6 mg, 63% yield) as light yellow oil.

6. Unsuccessful substrates

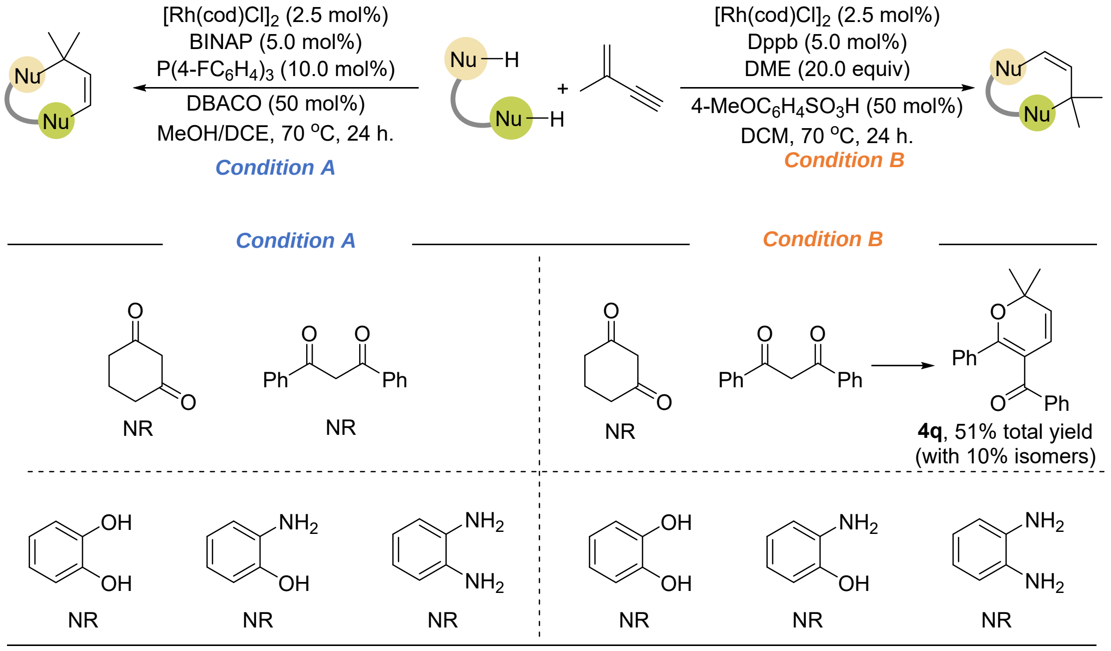

7. Crystallographic data

Crystal data and structure refinement for **3d** (CCDC 2384839)

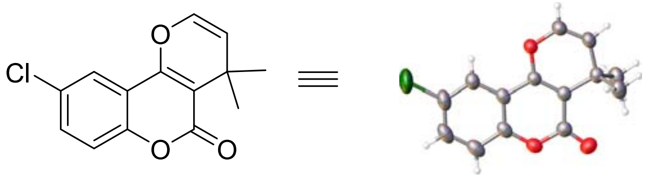

Table S14 Crystal data and structure refinement for 3d.

Identification code	**3d**
Empirical formula	$C_{14}H_{11}ClO_3$
Formula weight	262.68
Temperature/K	298.0
Crystal system	orthorhombic
Space group	Pnma
a/Å	16.9170(8)
b/Å	6.9625(4)
c/Å	20.9672(11)
α/°	90
β/°	90
γ/°	90
Volume/Å^3	2469.6(2)
Z	8
ρ_{calc}g/cm^3	1.413
μ/mm^{-1}	0.306
F(000)	1088.0
Crystal size/mm^3	0.21 × 0.16 × 0.12
Radiation	MoKα (λ = 0.71073)
2Θ range for data collection/°	3.094 to 52.906
Index ranges	-15 ≤ h ≤ 21, -8 ≤ k ≤ 7, -26 ≤ l ≤ 26
Reflections collected	20959
Independent reflections	2756 [R_{int} = 0.0731, R_{sigma} = 0.0423]
Data/restraints/parameters	2756/0/213
Goodness-of-fit on F^2	1.051
Final R indexes [I>=2σ (I)]	R_1 = 0.0541, wR_2 = 0.1493
Final R indexes [all data]	R_1 = 0.0871, wR_2 = 0.1751
Largest diff. peak/hole / e Å^{-3}	0.27/-0.44

Crystal data and structure refinement for **3k** (CCDC 2405848)

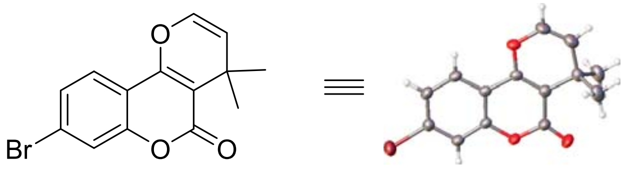

Table S15 Crystal data and structure refinement for 3k.

Identification code	**3k**
Empirical formula	$C_{14}H_{11}O_3Br$
Formula weight	307.14
Temperature/K	295.0
Crystal system	orthorhombic
Space group	Pbcm
a/Å	8.6358(13)
b/Å	21.155(3)
c/Å	6.8598(9)
α/°	90
β/°	90
γ/°	90
Volume/Å^3	1253.2(3)
Z	4
ρ_{calc}g/cm^3	1.628
μ/mm^{-1}	3.276
F(000)	616.0
Crystal size/mm^3	0.17 × 0.13 × 0.11
Radiation	MoKα (λ = 0.71073)
2Θ range for data collection/°	3.85 to 50.07
Index ranges	-10 ≤ h ≤ 10, -25 ≤ k ≤ 25, -7 ≤ l ≤ 8
Reflections collected	12730
Independent reflections	1186 [R_{int} = 0.0935, R_{sigma} = 0.1066]
Data/restraints/parameters	1186/0/107
Goodness-of-fit on F^2	1.076
Final R indexes [I>=2σ (I)]	R_1 = 0.0511, wR_2 = 0.1300
Final R indexes [all data]	R_1 = 0.0577, wR_2 = 0.1383
Largest diff. peak/hole / e Å^{-3}	0.39/-0.36

Crystal data and structure refinement for **4d** (CCDC 2384836)

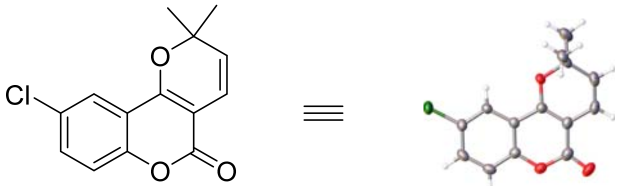

Table S16 Crystal data and structure refinement for 4d.

Identification code	**4d**
Empirical formula	$C_{14}H_{11}O_3Cl$
Formula weight	262.68
Temperature/K	150.0
Crystal system	triclinic
Space group	P-1
a/Å	7.2396(5)
b/Å	12.2973(10)
c/Å	13.7364(11)
α/°	92.145(4)
β/°	95.435(3)
γ/°	97.982(4)
Volume/Å^3	1204.04(16)
Z	4
ρ_{calc}g/cm^3	1.449
μ/mm^{-1}	0.313
F(000)	544.0
Crystal size/mm^3	0.21 × 0.13 × 0.11
Radiation	MoKα (λ = 0.71073)
2Θ range for data collection/°	2.982 to 53.142
Index ranges	-9 ≤ h ≤ 9, -15 ≤ k ≤ 15, -15 ≤ l ≤ 17
Reflections collected	16122
Independent reflections	4952 [R_{int} = 0.0606, R_{sigma} = 0.0567]
Data/restraints/parameters	4952/0/329
Goodness-of-fit on F^2	1.068
Final R indexes [I>=2σ (I)]	R_1 = 0.0408, wR_2 = 0.1039
Final R indexes [all data]	R_1 = 0.0585, wR_2 = 0.1149
Largest diff. peak/hole / e Å^{-3}	0.25/-0.27

Crystal data and structure refinement for **4e** (CCDC 2384837)

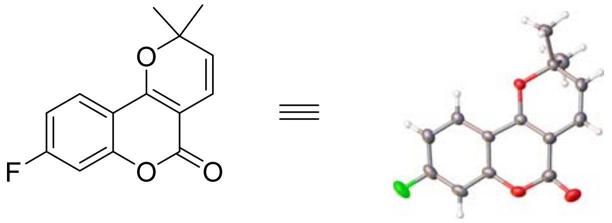

Table S17 Crystal data and structure refinement for 4e.

Identification code	**4e**
Empirical formula	$C_{14}H_{11}O_3F$
Formula weight	246.23
Temperature/K	296.3
Crystal system	monoclinic
Space group	C2/c
a/Å	8.4159(6)
b/Å	18.7306(16)
c/Å	15.1115(13)
α/°	90
β/°	99.176(4)
γ/°	90
Volume/Å^3	2351.6(3)
Z	8
ρ_{calc}g/cm^3	1.391
μ/mm^{-1}	0.108
F(000)	1024.0
Crystal size/mm^3	0.25 × 0.18 × 0.14
Radiation	MoKα (λ = 0.71073)
2Θ range for data collection/°	5.136 to 51.97
Index ranges	-10 ≤ h ≤ 5, -21 ≤ k ≤ 22, -18 ≤ l ≤ 18
Reflections collected	8521
Independent reflections	2261 [R_{int} = 0.0402, R_{sigma} = 0.0357]
Data/restraints/parameters	2261/0/165
Goodness-of-fit on F^2	1.037
Final R indexes [I>=2σ (I)]	R_1 = 0.0456, wR_2 = 0.1154
Final R indexes [all data]	R_1 = 0.0742, wR_2 = 0.1320
Largest diff. peak/hole / e Å^{-3}	0.16/-0.19

Crystal data and structure refinement for **4g** (CCDC 2384838)

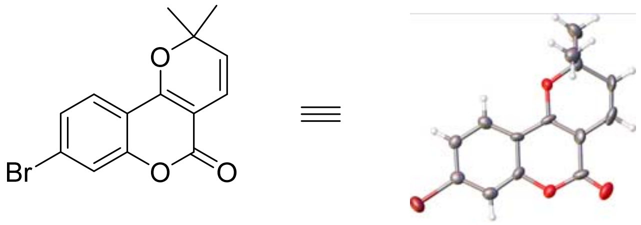

Table S18 Crystal data and structure refinement for 4g.

Identification code	**4g**
Empirical formula	$C_{14}H_{11}O_3Br$
Formula weight	307.14
Temperature/K	296.3
Crystal system	triclinic
Space group	P1
a/Å	7.1892(7)
b/Å	12.3763(11)
c/Å	14.3209(12)
α/°	85.576(4)
β/°	78.661(4)
γ/°	81.573(4)
Volume/Å^3	1234.32(19)
Z	4
ρ_{calc}g/cm^3	1.653
μ/mm^{-1}	3.327
F(000)	616.0
Crystal size/mm^3	0.16 × 0.14 × 0.12
Radiation	MoKα (λ = 0.71073)
2Θ range for data collection/°	3.33 to 50.7
Index ranges	-8 ≤ h ≤ 8, -14 ≤ k ≤ 14, -17 ≤ l ≤ 17
Reflections collected	21078
Independent reflections	8753 [R_{int} = 0.0479, R_{sigma} = 0.0591]
Data/restraints/parameters	8753/3/657
Goodness-of-fit on F^2	1.048
Final R indexes [I>=2σ (I)]	R_1 = 0.0365, wR_2 = 0.0910
Final R indexes [all data]	R_1 = 0.0504, wR_2 = 0.0988
Largest diff. peak/hole / e Å^{-3}	0.31/-0.61
Flack parameter	0.42(2)

Crystal data and structure refinement for **4k** (CCDC 2384835)

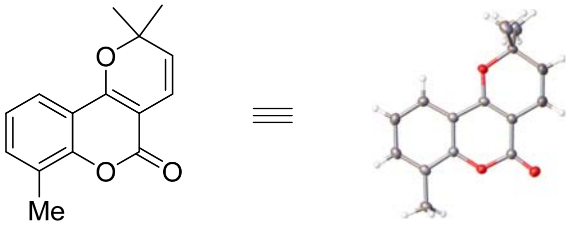

Table S19 Crystal data and structure refinement for 4k.

Identification code	**4k**
Empirical formula	$C_{15}H_{14}O_3$
Formula weight	242.26
Temperature/K	296.3
Crystal system	orthorhombic
Space group	Pnma
a/Å	15.2572(4)
b/Å	6.8430(2)
c/Å	11.4579(3)
α/°	90
β/°	90
γ/°	90
Volume/Å^3	1196.26(6)
Z	4
ρ_{calc}g/cm^3	1.345
μ/mm^{-1}	0.093
F(000)	512.0
Crystal size/mm^3	0.21 × 0.14 × 0.12
Radiation	MoKα (λ = 0.71073)
2Θ range for data collection/°	4.446 to 52.864
Index ranges	-19 ≤ h ≤ 17, -5 ≤ k ≤ 8, -13 ≤ l ≤ 14
Reflections collected	10904
Independent reflections	1315 [R_{int} = 0.0503, R_{sigma} = 0.0341]
Data/restraints/parameters	1315/0/108
Goodness-of-fit on F^2	1.140
Final R indexes [I>=2σ (I)]	R_1 = 0.0367, wR_2 = 0.1129
Final R indexes [all data]	R_1 = 0.0505, wR_2 = 0.1326
Largest diff. peak/hole / e Å^{-3}	0.41/-0.49

Crystal data and structure refinement for **10d** (CCDC 2384834)

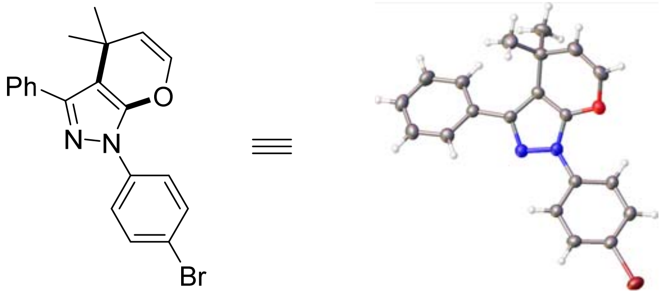

Table S20 Crystal data and structure refinement for 10d.

Identification code	**10d**
Empirical formula	$C_{20}H_{17}N_2OBr$
Formula weight	381.26
Temperature/K	298.0
Crystal system	monoclinic
Space group	$P2_1/c$
a/Å	17.3667(12)
b/Å	6.0766(4)
c/Å	16.2575(16)
α/°	90
β/°	91.591(5)
γ/°	90
Volume/Å^3	1715.0(2)
Z	4
ρ_{calc}g/cm^3	1.477
μ/mm^{-1}	2.405
F(000)	776.0
Crystal size/mm^3	0.24 × 0.15 × 0.12
Radiation	MoKα (λ = 0.71073)
2Θ range for data collection/°	4.692 to 52.842
Index ranges	-21 ≤ h ≤ 16, -7 ≤ k ≤ 7, -20 ≤ l ≤ 20
Reflections collected	15712
Independent reflections	3507 [R_{int} = 0.0667, R_{sigma} = 0.0552]
Data/restraints/parameters	3507/0/219
Goodness-of-fit on F^2	1.113
Final R indexes [I>=2σ (I)]	R_1 = 0.0662, wR_2 = 0.2008
Final R indexes [all data]	R_1 = 0.0770, wR_2 = 0.2136
Largest diff. peak/hole / e Å^{-3}	1.25/-1.03

Crystal data and structure refinement for **10k** (CCDC 2384833)

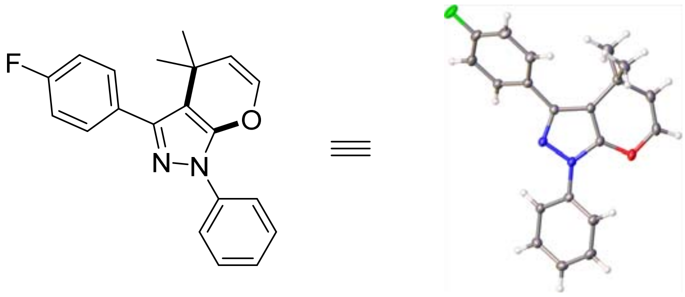

Table S21 Crystal data and structure refinement for 10k.

Identification code	**10k**
Empirical formula	$C_{20}H_{17}N_2OF$
Formula weight	320.35
Temperature/K	296.3
Crystal system	triclinic
Space group	P-1
a/Å	6.0254(4)
b/Å	11.7227(8)
c/Å	11.9718(9)
α/°	77.622(3)
β/°	80.706(3)
γ/°	75.577(3)
Volume/Å^3	794.69(10)
Z	2
ρ_{calc}g/cm^3	1.339
μ/mm^{-1}	0.092
F(000)	336.0
Crystal size/mm^3	0.19 × 0.16 × 0.12
Radiation	MoKα (λ = 0.71073)
2Θ range for data collection/°	5.502 to 52.904
Index ranges	-7 ≤ h ≤ 7, -14 ≤ k ≤ 14, -14 ≤ l ≤ 14
Reflections collected	9967
Independent reflections	3201 [R_{int} = 0.0459, R_{sigma} = 0.0461]
Data/restraints/parameters	3201/0/219
Goodness-of-fit on F^2	1.144
Final R indexes [I>=2σ (I)]	R_1 = 0.0442, wR_2 = 0.1295
Final R indexes [all data]	R_1 = 0.0562, wR_2 = 0.1509
Largest diff. peak/hole / e Å^{-3}	0.29/-0.31

8. References

[1] (a) X. Feng, Z. Qin, X. Cheng, D. Liu, Y. Peng, H. Huang, B. Song, J. Bian, Z. Li, *J. Org. Chem.* **2021**, *86*, 12537-12548; (b) S.-Y. Hao, S.-L. Feng, X.-R. Wang, Z. Wang, S.-W. Chen, L. Hui, *Bioorg. Med. Chem. Lett.* **2019**, *29*, 2129-2135; (c) Z. Huang, O. Matsubara, S. Jia, E. Tokunaga, N. Shibata, *Org. Lett.* **2017**, *19*, 934-937.

[2] (a) R. H. Wang, Y. L. Li, H. J. He, Y. C. Xiao, F. E. Chen, *Chem. Eur. J.* **2021**, *27*, 4302-4306; (b) J. L. Howard, W. Nicholson, Y. Sagatov, D. L. Browne, *Beilstein J. Org. Chem.* **2017**, *13*, 1950-1956; (c) V. Kumar, C.-K. Chang, K.-P. Tan, Y.-S. Jung, S.-H. Chen, Y.-S. E. Cheng, P.-H. Liang, *Org. Lett.* **2014**, *16*, 5060-5063.

[3] Y. Li, Y. C. Hu, H. Zheng, D. W. Ji, Y. F. Cong, Q. A. Chen, *Eur. J. Org. Chem.* **2019**, *2019*, 6510-6514.

[4] C.-N. Huang, P.-Y. Kuo, C.-H. Lin, D.-Y. Yang, *Tetrahedron* **2007**, *63*, 10025-10033.

[5] R. Chen, X. Li, J. Xiao, M. Zhu, A. Sun, K. K. Wang, *J. Heterocycl. Chem.* **2024**, *61*, 1924-1931.

[6] E. J. Jung, Y. R. Lee, H.-J. Lee, *Bull. Korean Chem. Soc.* **2009**, *30*, 2833-2836.

[7] R. R. Shah, S. M. Desai, K. N. Trivedi, *Pharmazie* **1983**, *38*, 439-441.

[8] J. Moreau, C. Hubert, J. Batany, L. Toupet, T. Roisnel, J.-P. Hurvois, J.-L. Renaud, *J. Org. Chem.* **2009**, *74*, 8963-8973.

[9] V. Cadierno, J. Díez, J. Gimeno, N. Nebra, *J. Org. Chem.* **2008**, *73*, 5852–5858.

[10] S. Rohilla, S. Shah, V. K. Singh, *Org. Lett.* **2023**, *25*, 3733-3738.

[11] M. R. Saidi, K. Bigdeli, *J. Chem. Res. (S)* **1998**, 800-801.

9. Copies of NMR spectra.

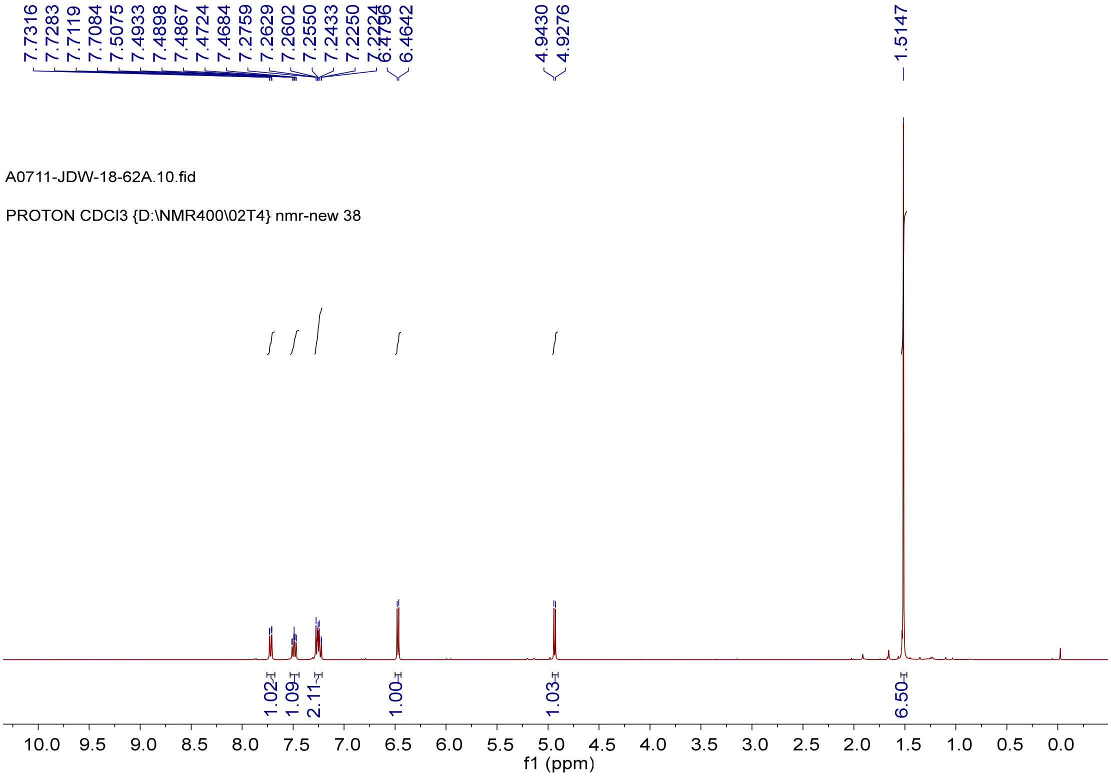

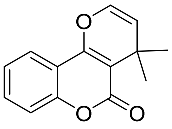

3a

^{1}H NMR (400 MHz, Chloroform-*d*)
^{13}C NMR (100 MHz, Chloroform-*d*)

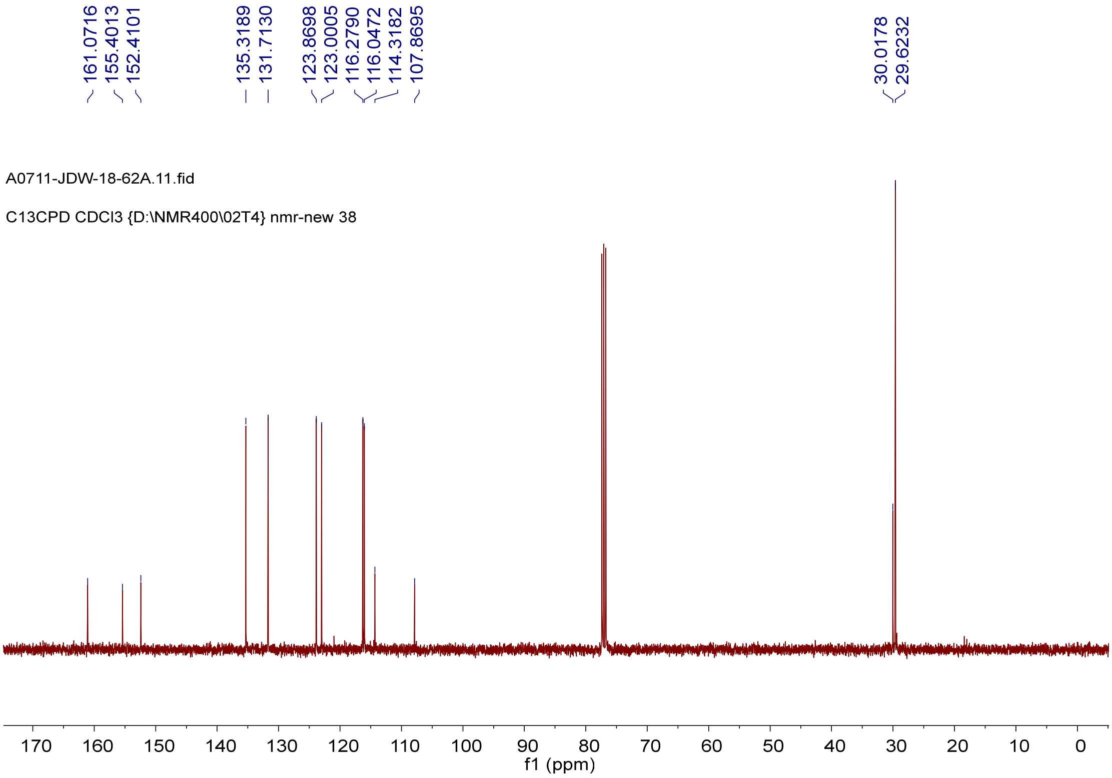

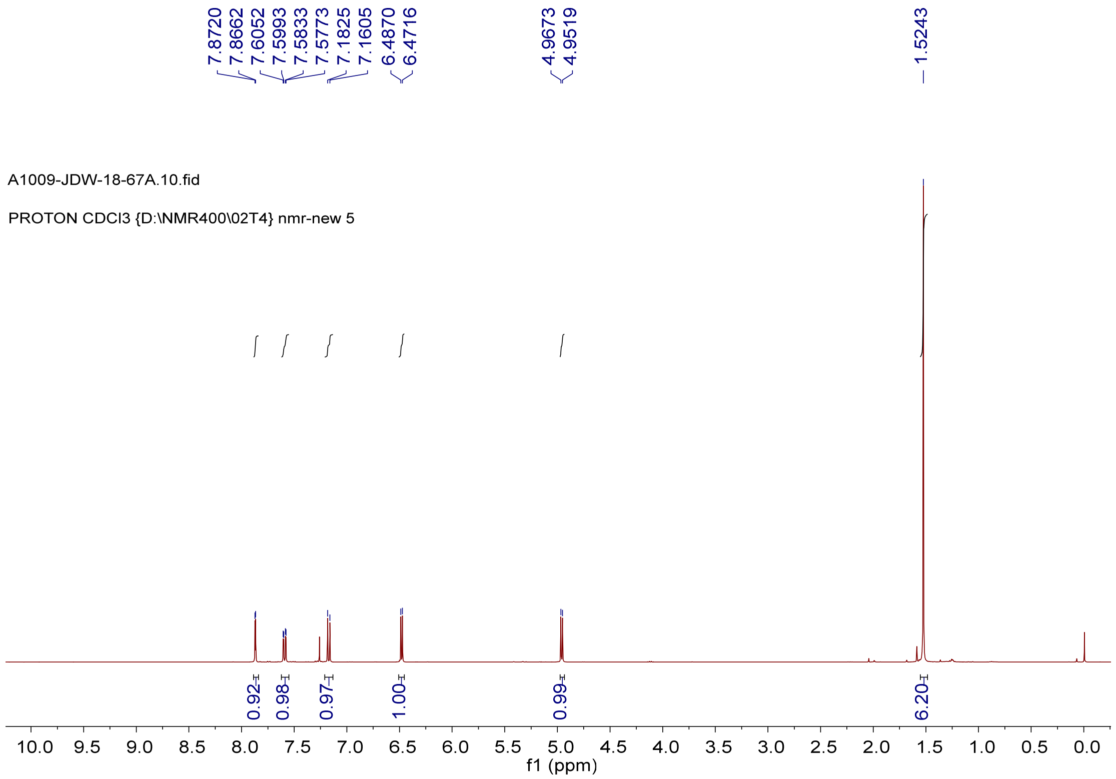

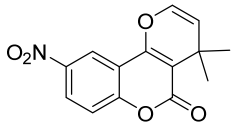

3b

^{1}H NMR (400 MHz, Chloroform-*d*)
^{13}C NMR (100 MHz, Chloroform-*d*)

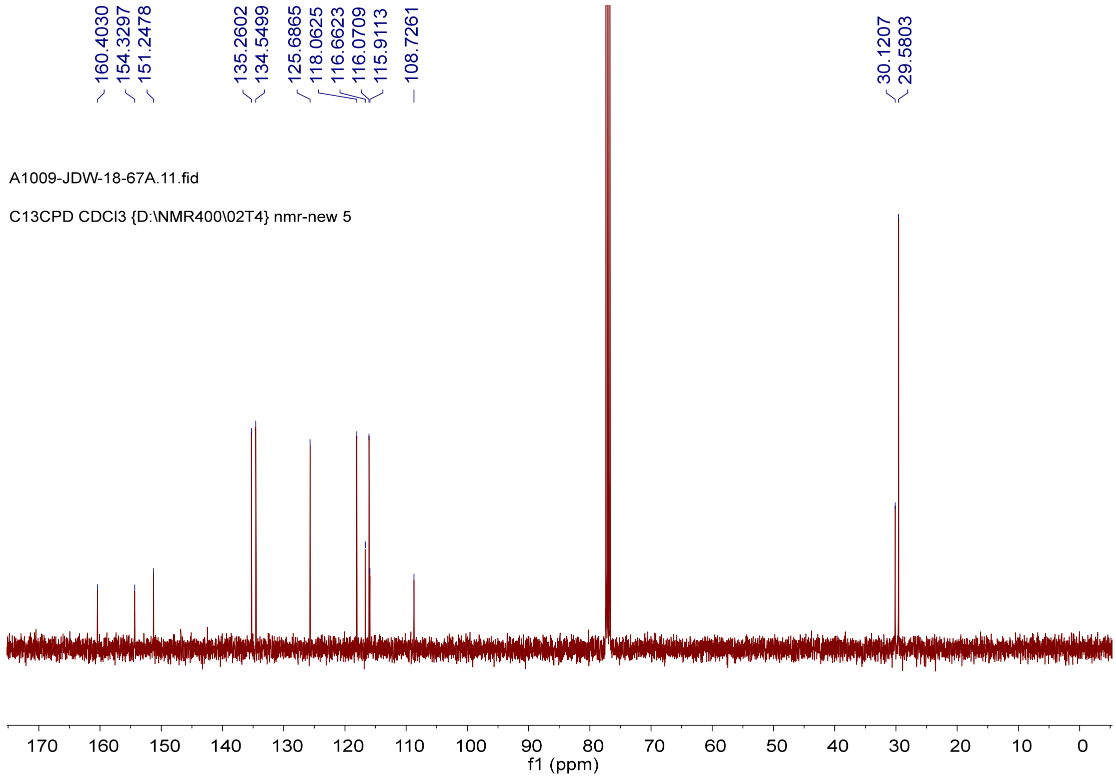

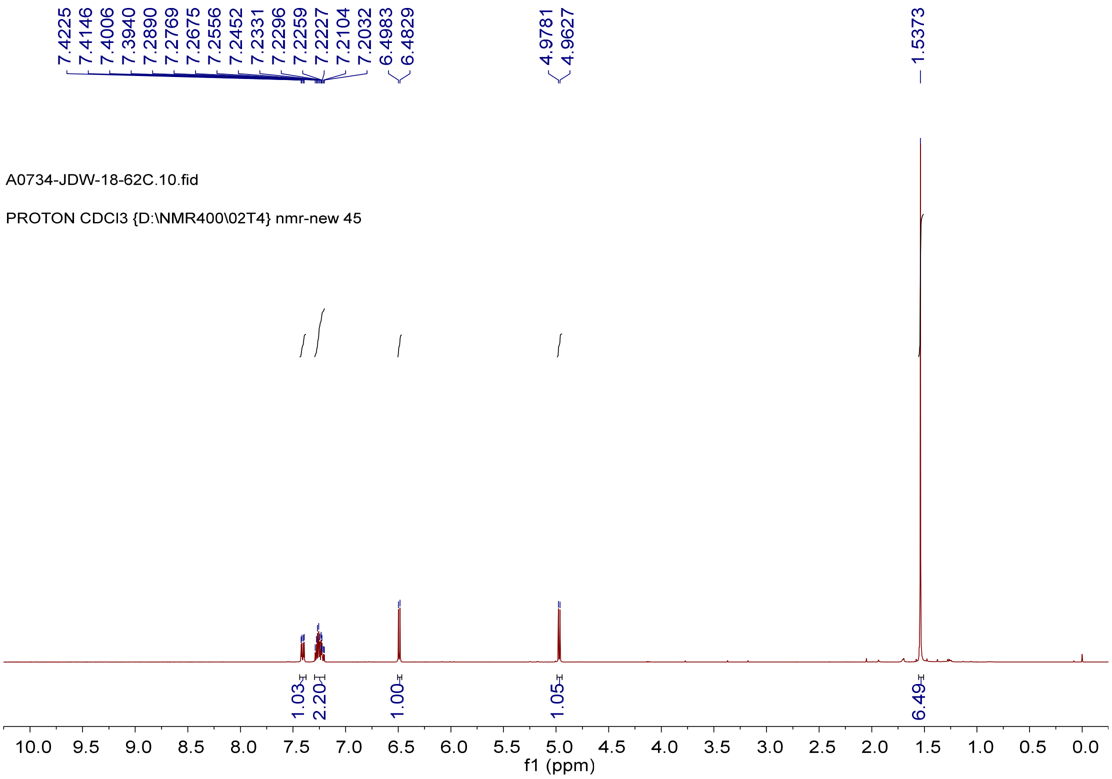

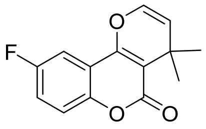

3c

^{1}H NMR (400 MHz, Chloroform-*d*)
^{13}C NMR (100 MHz, Chloroform-*d*)

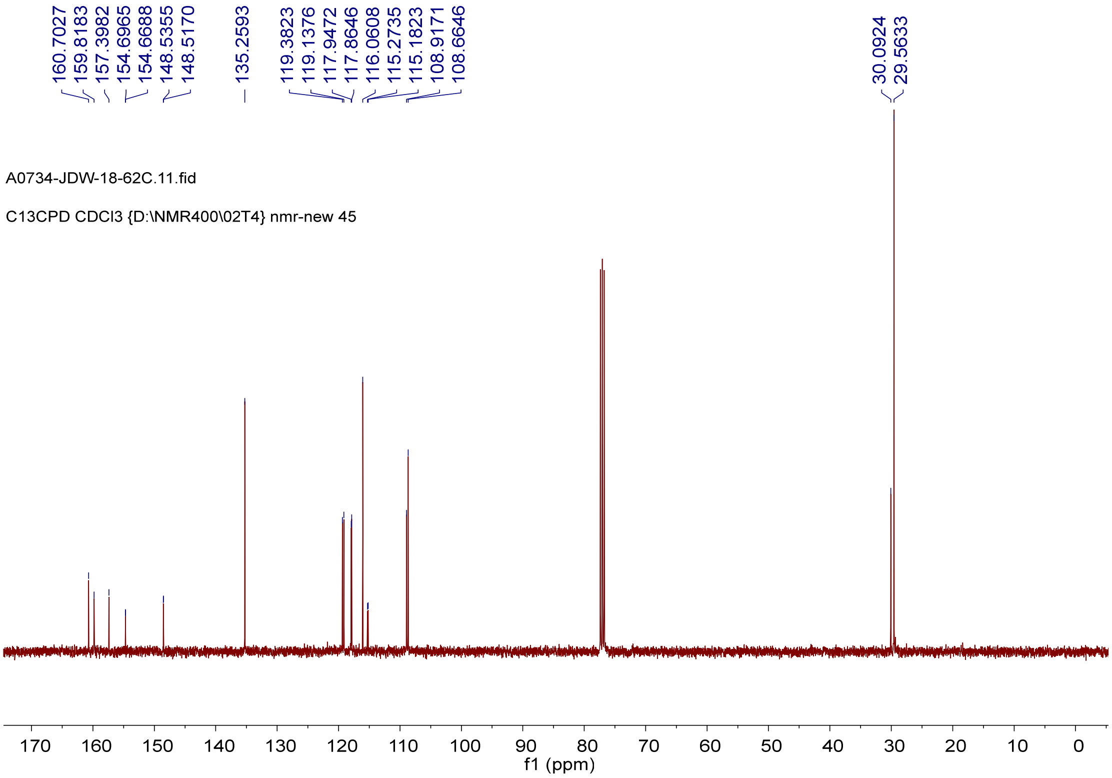

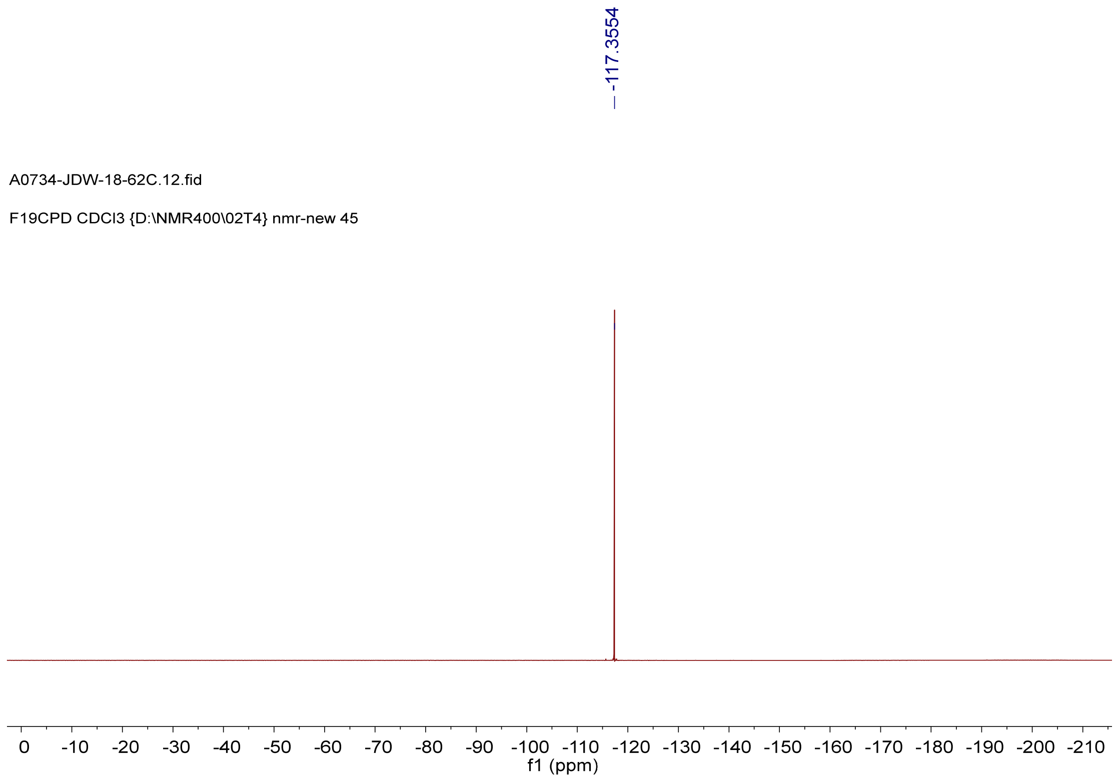

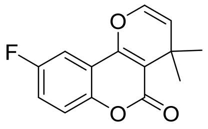

3c

^{19}**F NMR** (375 MHz, Chloroform-*d*)

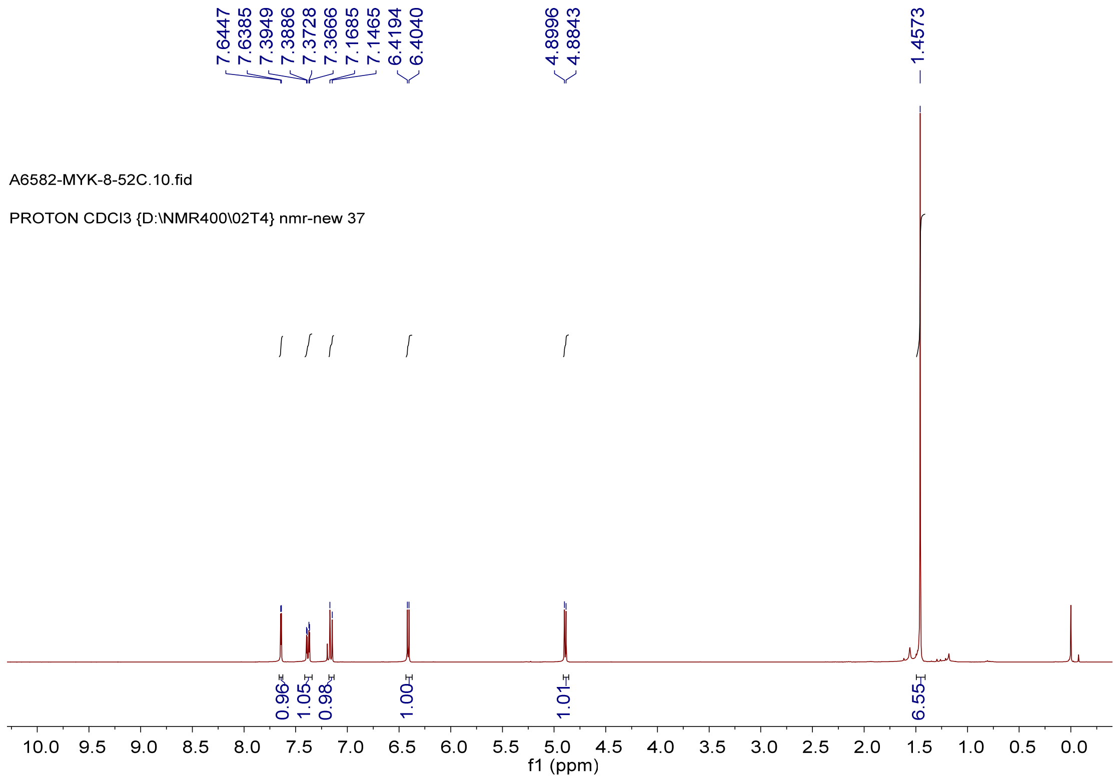

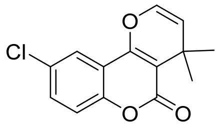

3d

^{1}H NMR (400 MHz, Chloroform-*d*)

^{13}C NMR (100 MHz, Chloroform-*d*)

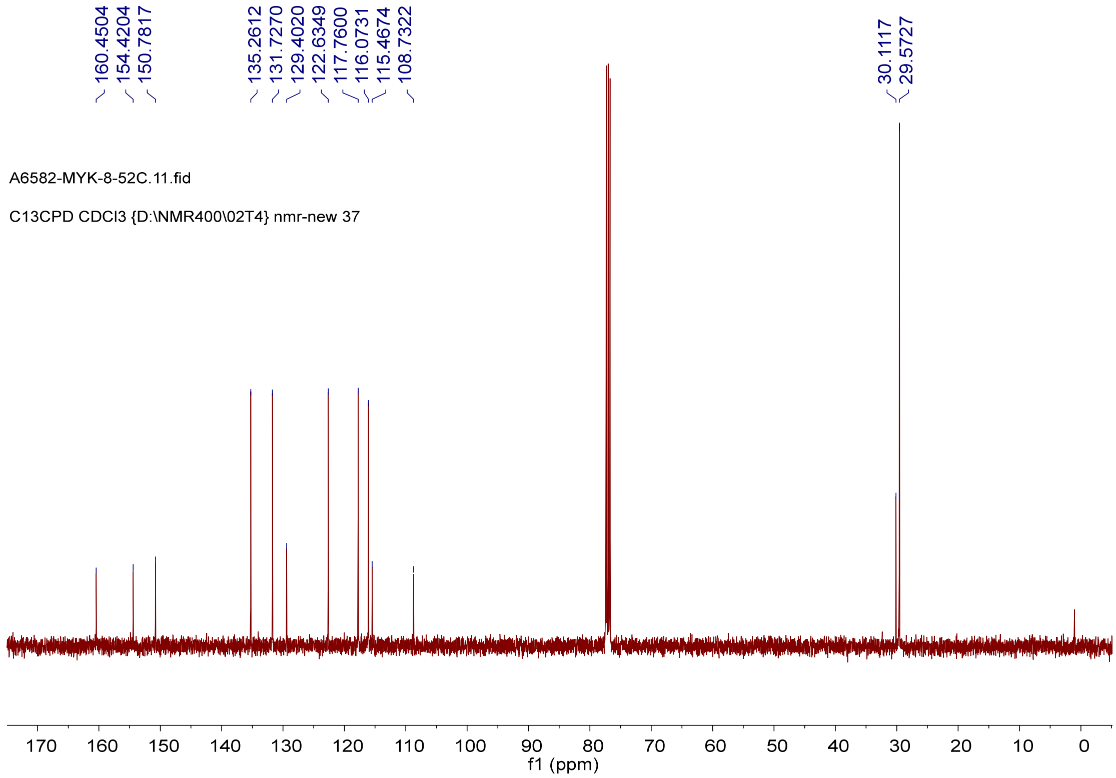

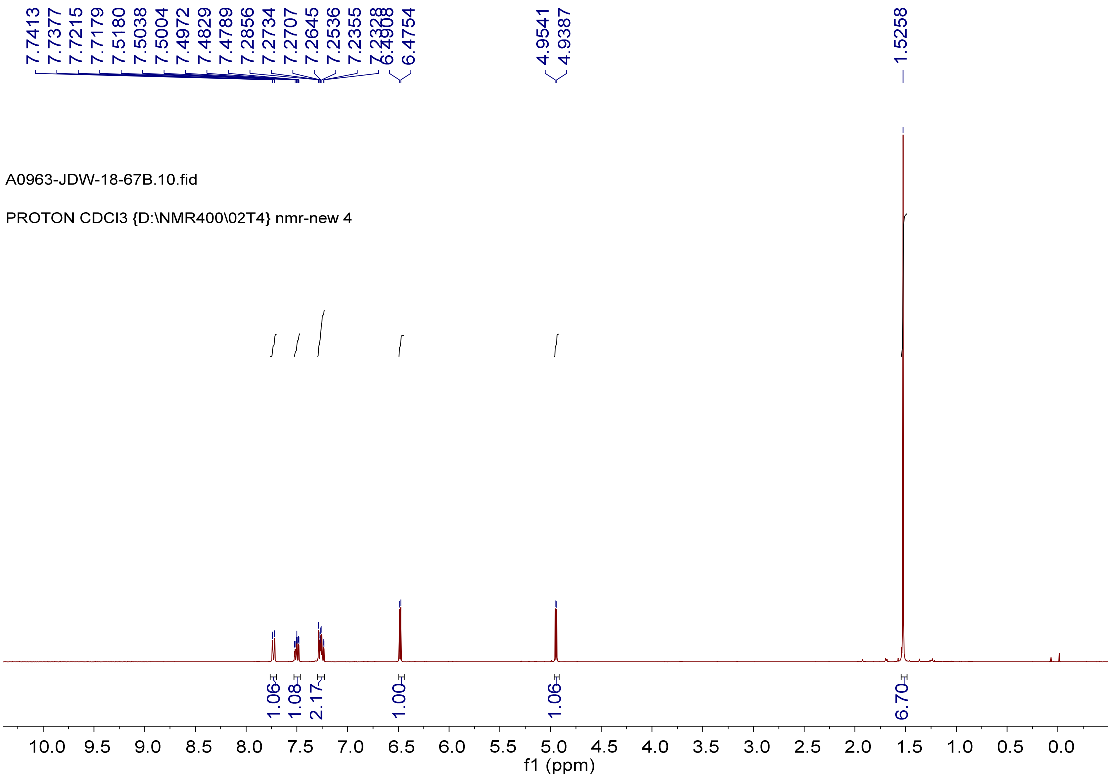

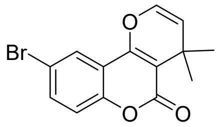

3e

^{1}H NMR (400 MHz, Chloroform-*d*)

^{13}C NMR (100 MHz, Chloroform-*d*)

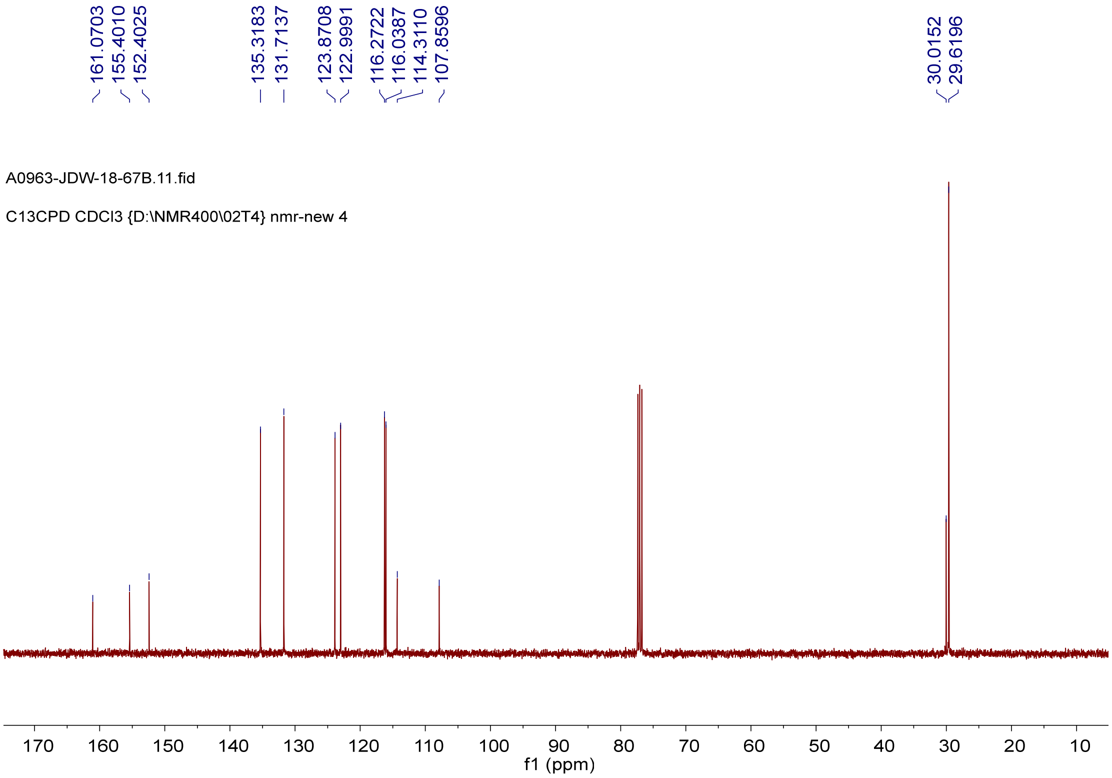

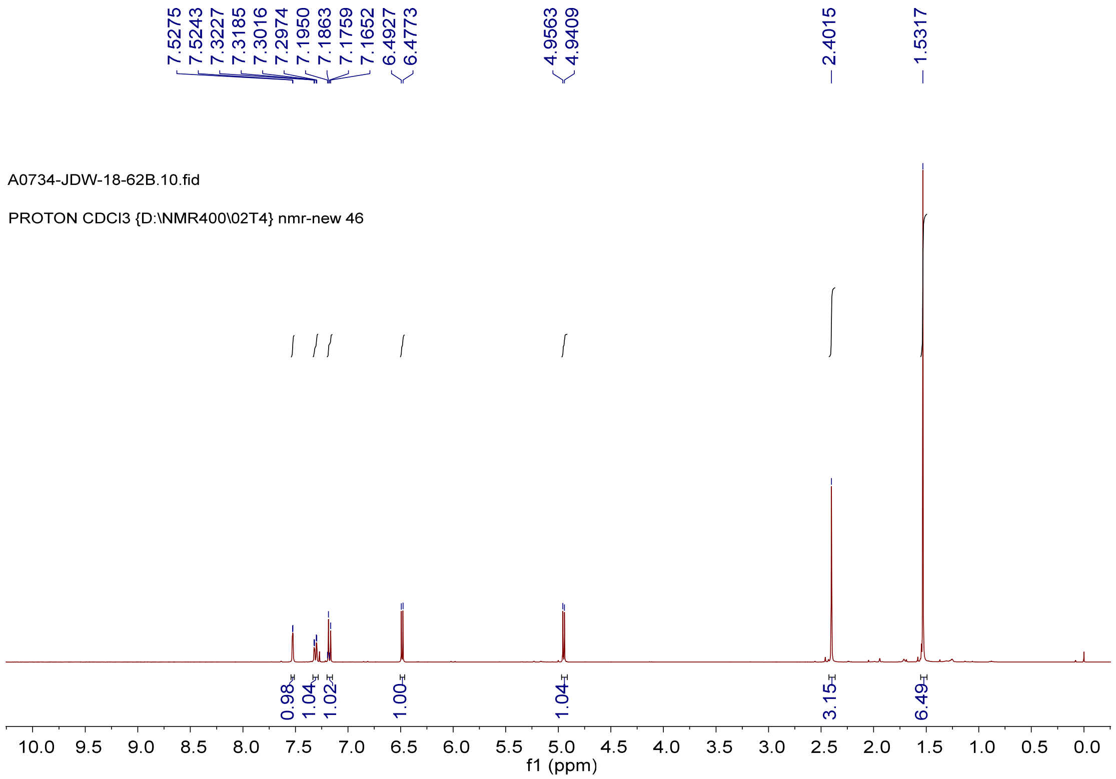

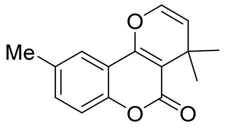

3f

^{1}H NMR (400 MHz, Chloroform-*d*)
^{13}C NMR (100 MHz, Chloroform-*d*)

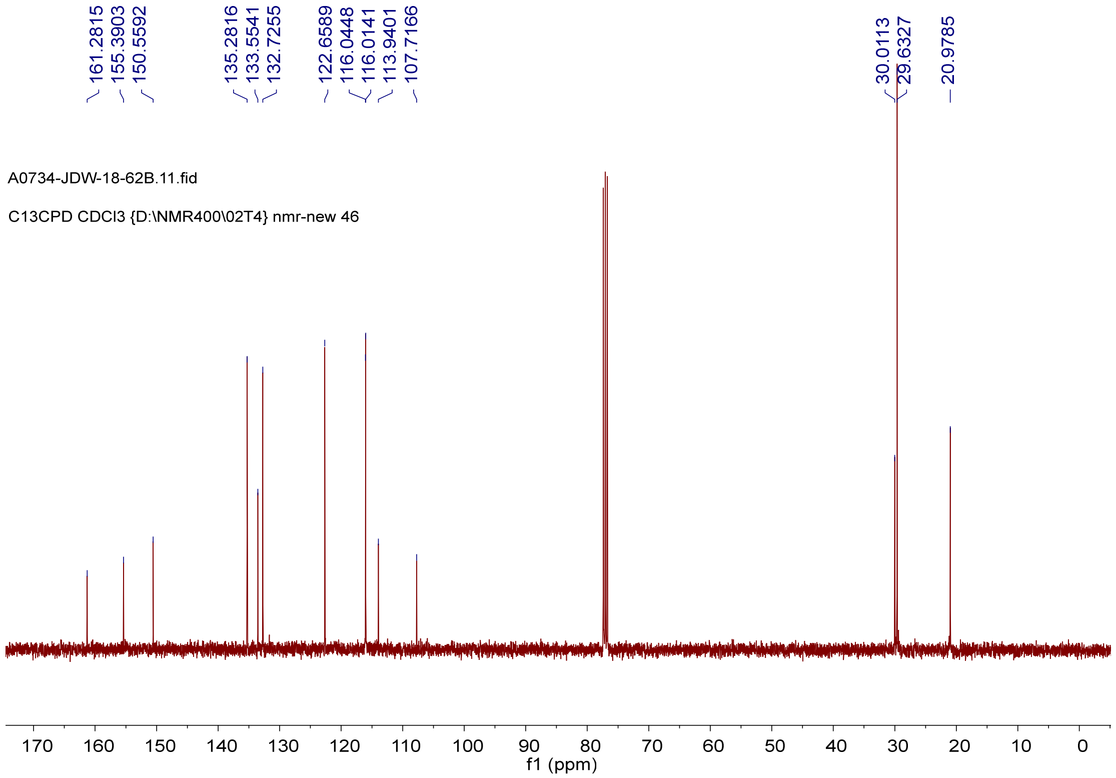

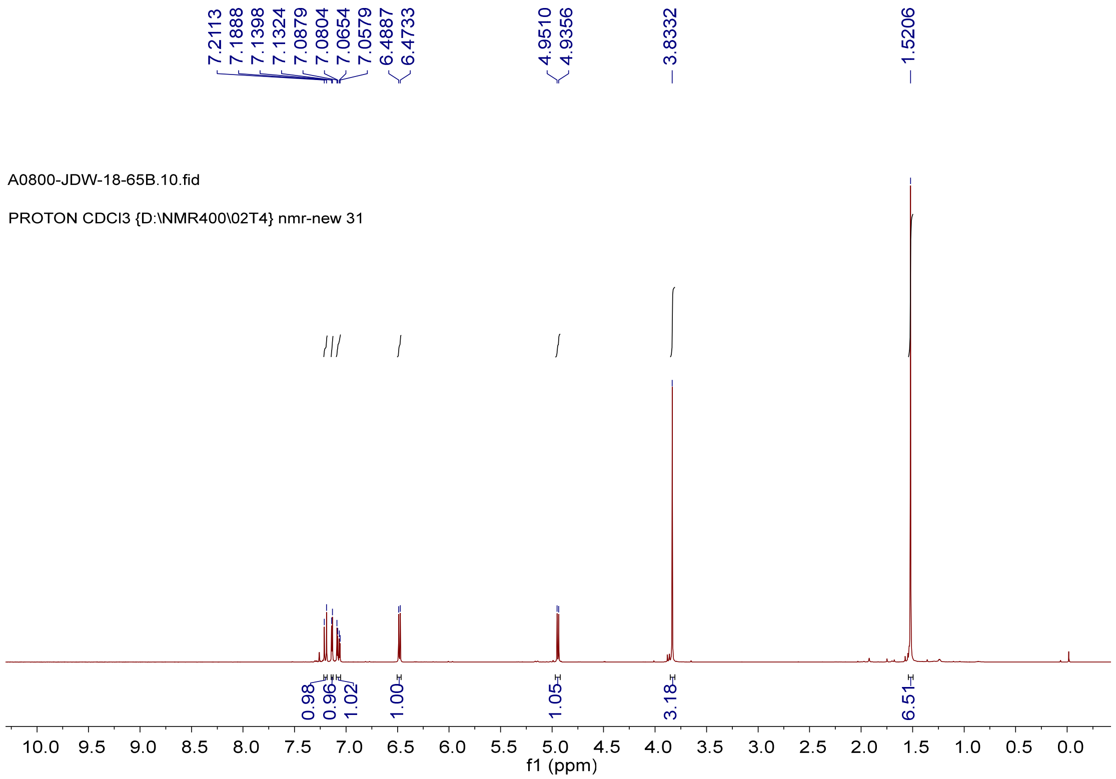

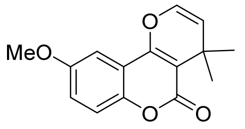

3g

^{1}H NMR (400 MHz, Chloroform-*d*)
^{13}C NMR (100 MHz, Chloroform-*d*)

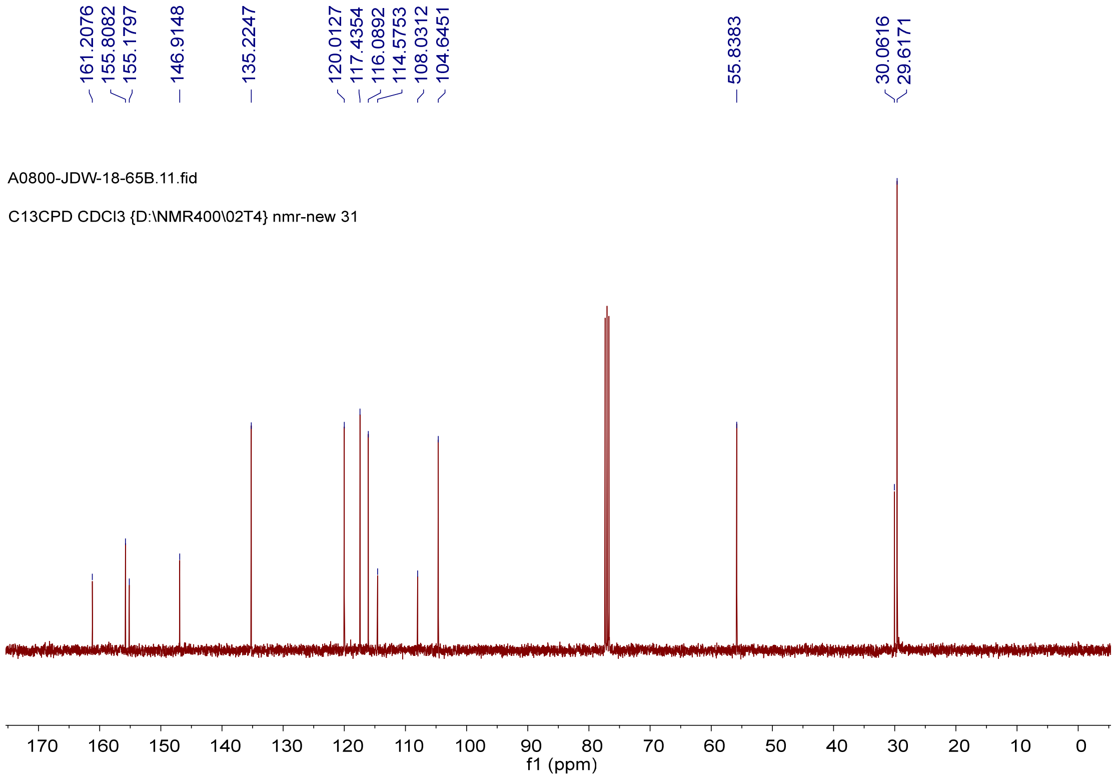

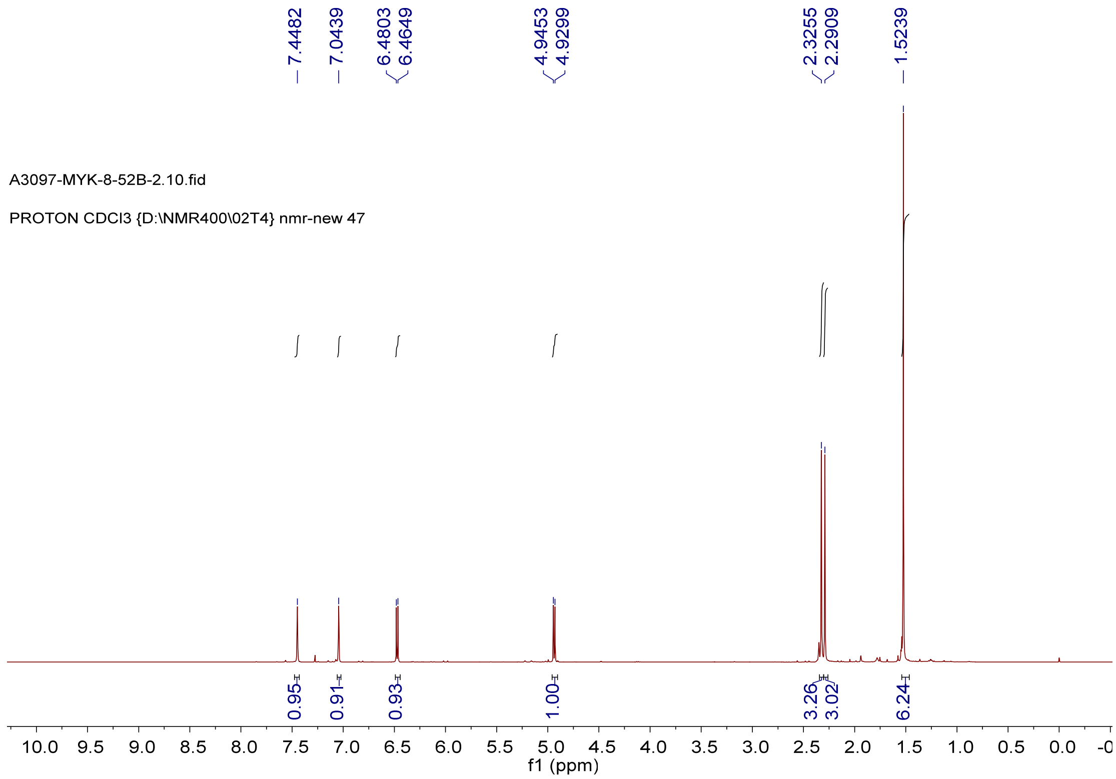

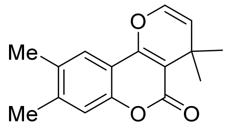

3h

^{1}H NMR (400 MHz, Chloroform-*d*)
^{13}C NMR (100 MHz, Chloroform-*d*)

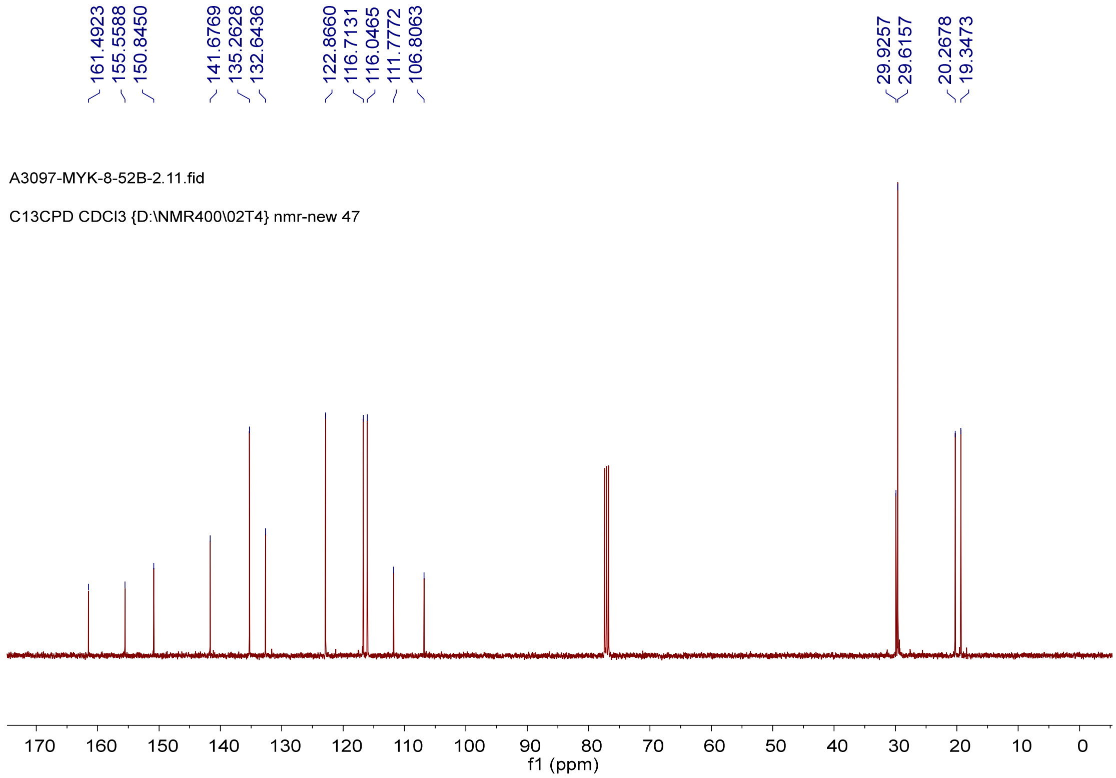

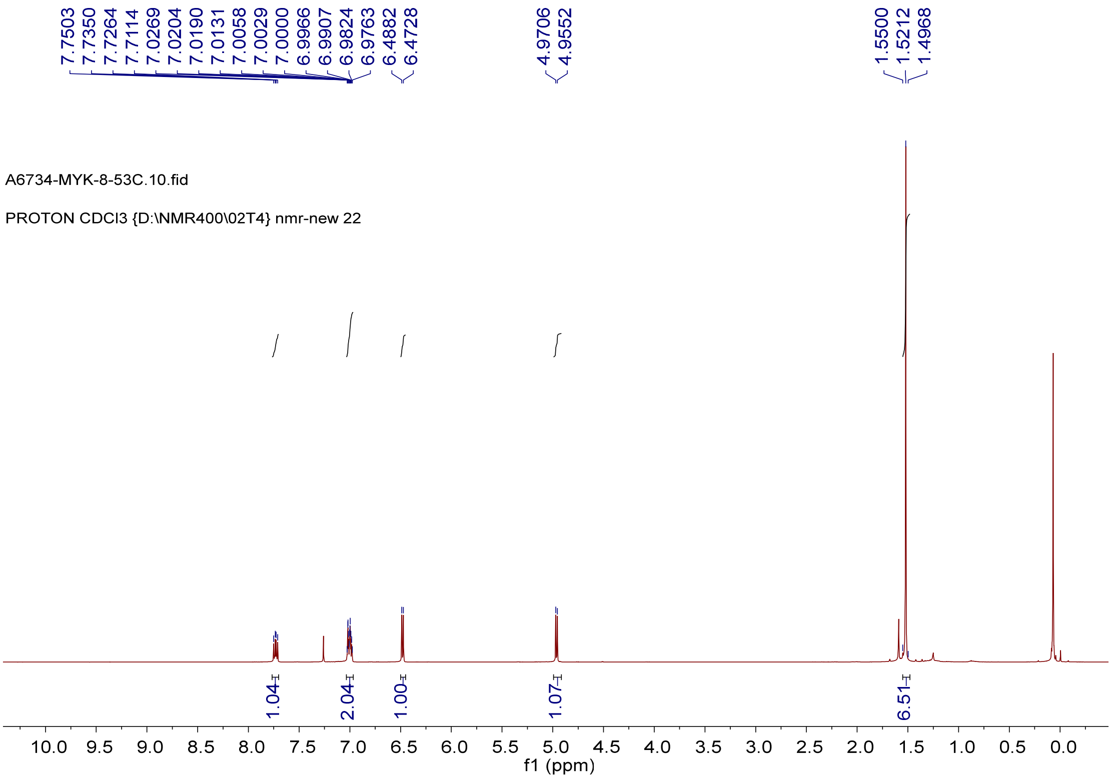

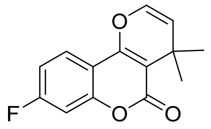

3i

^{1}H NMR (400 MHz, Chloroform-*d*)

^{13}C NMR (100 MHz, Chloroform-*d*)

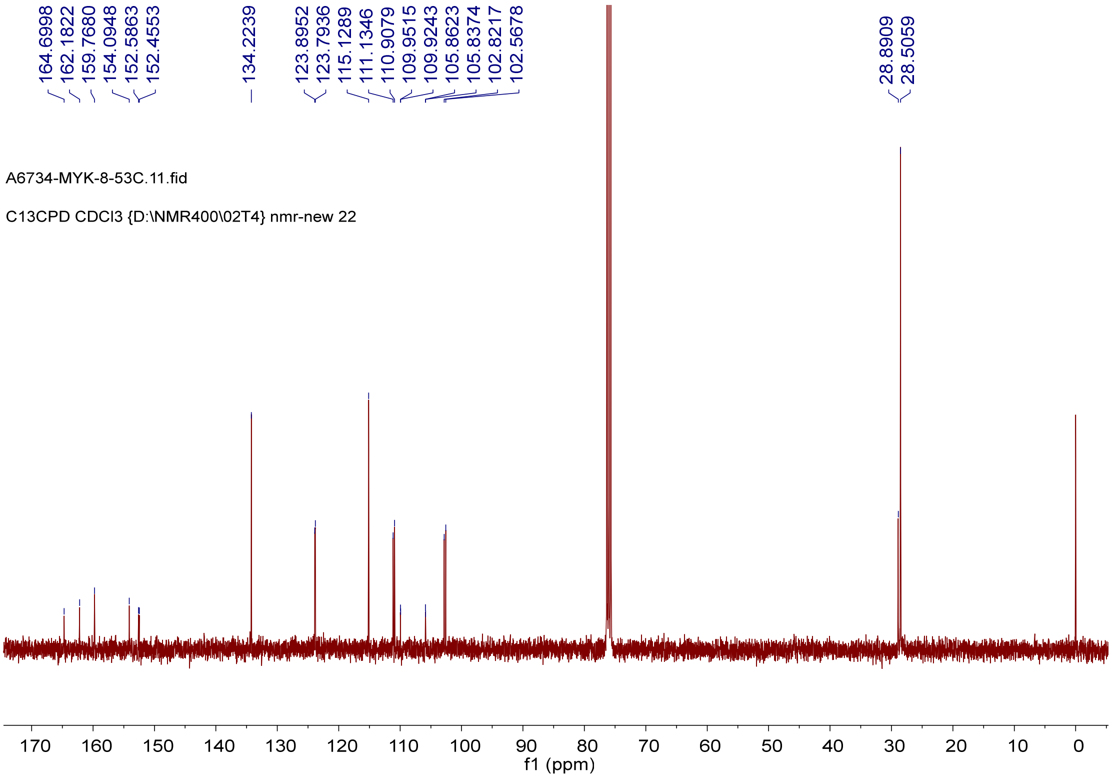

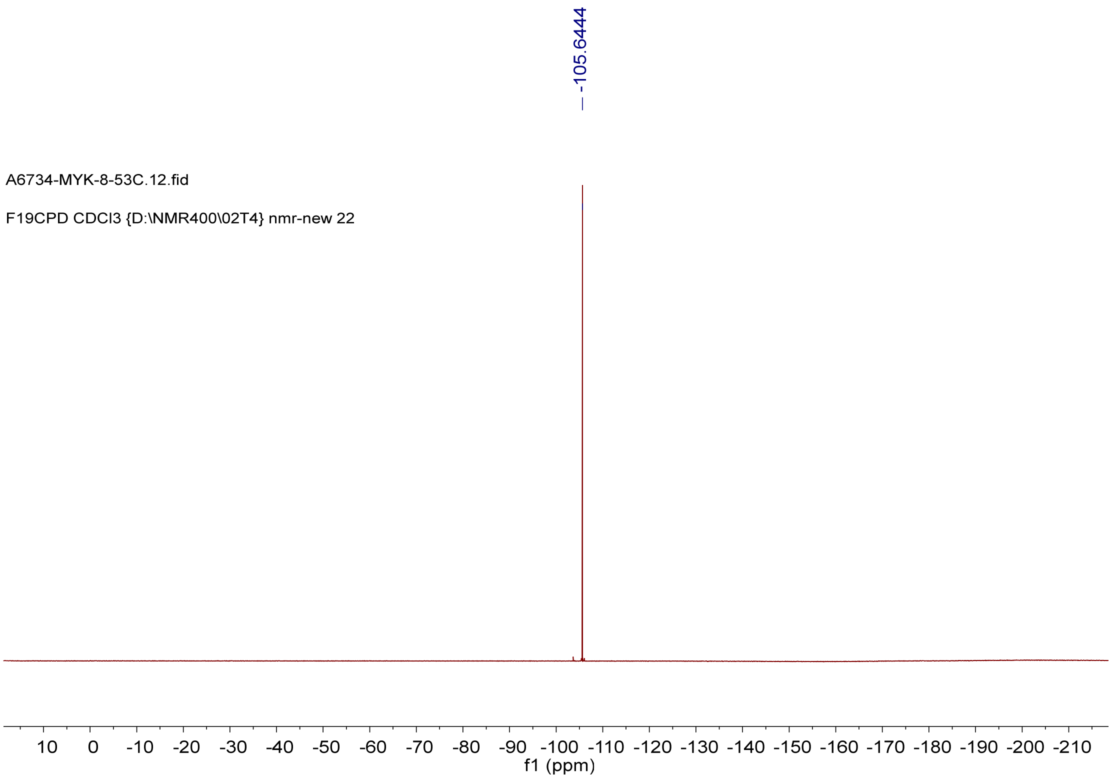

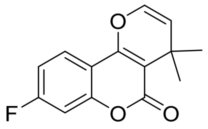

3i

^{19}**F NMR** (375 MHz, Chloroform-*d*)

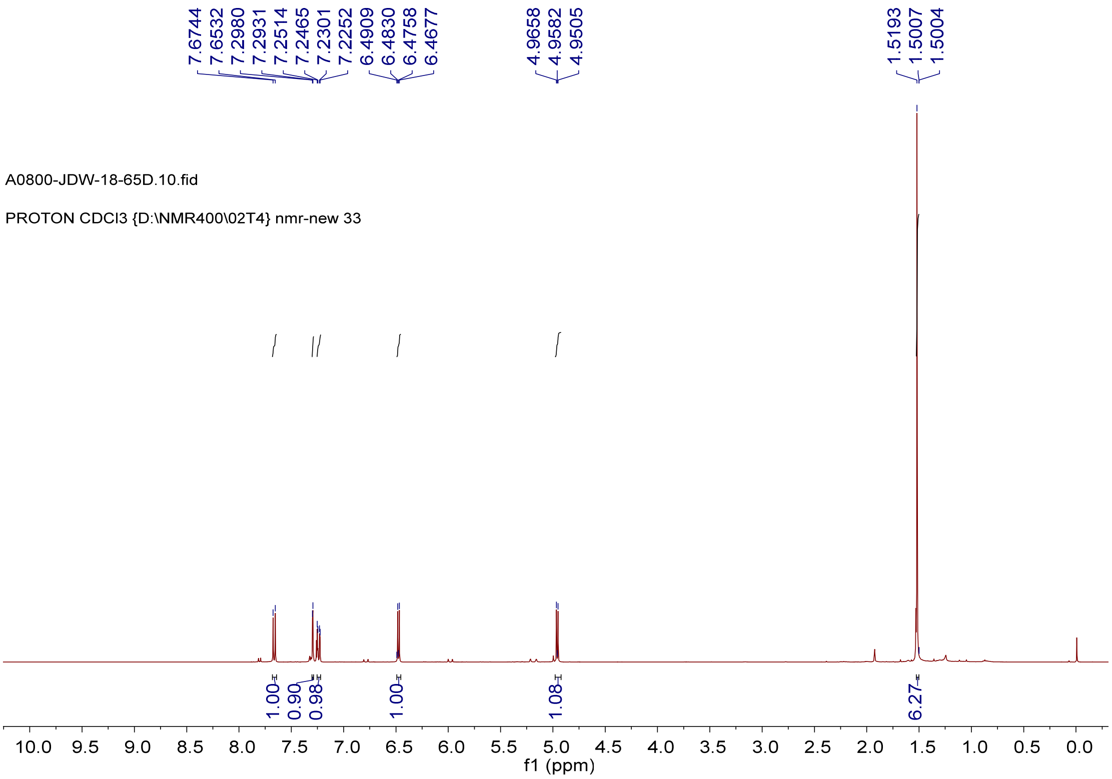

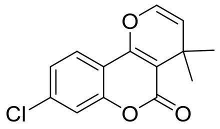

3j

^{1}H NMR (400 MHz, Chloroform-*d*)
^{13}C NMR (100 MHz, Chloroform-*d*)

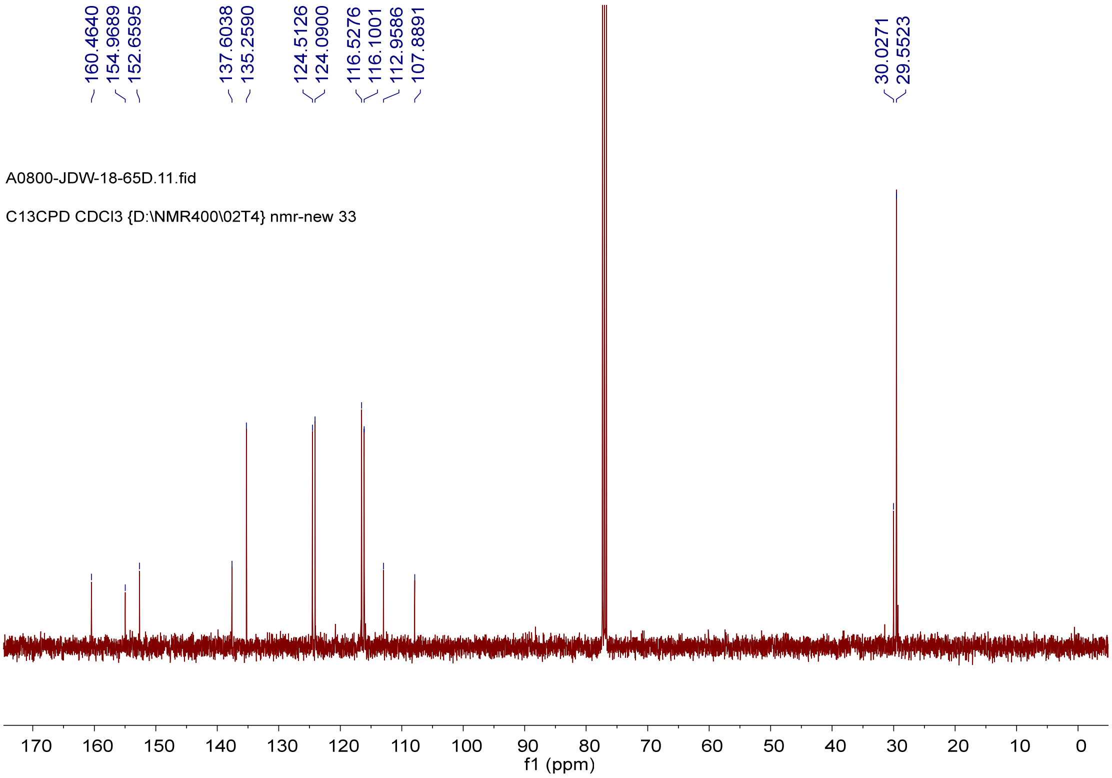

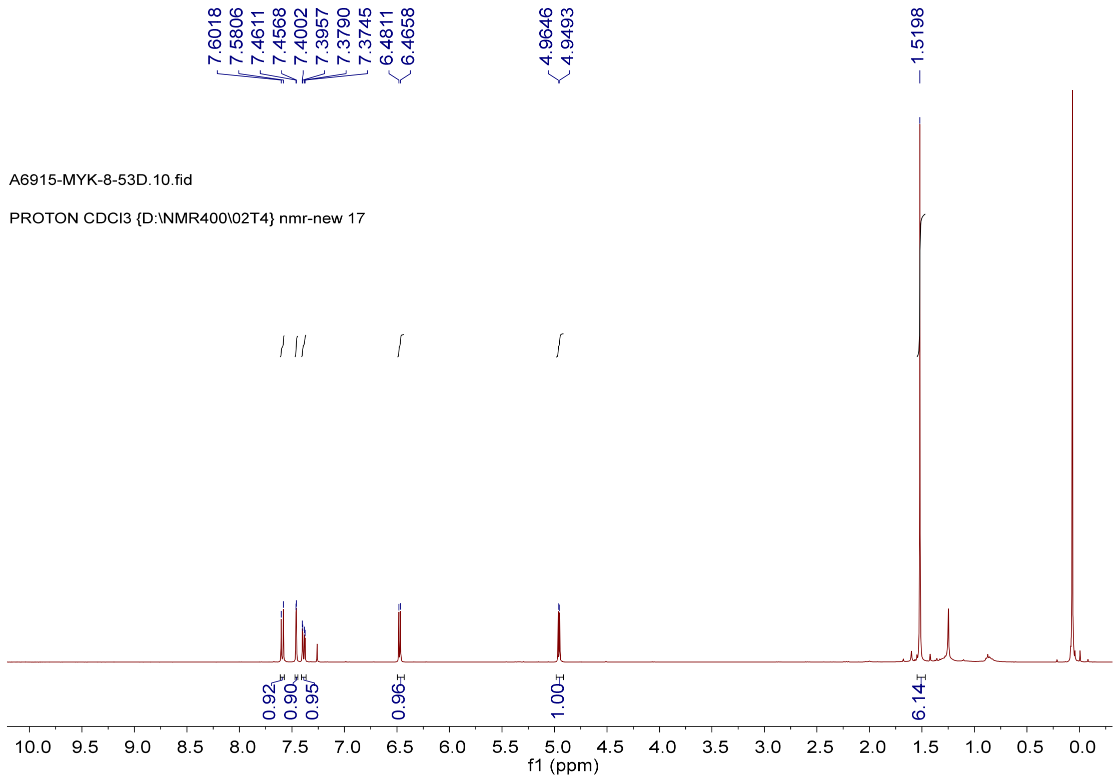

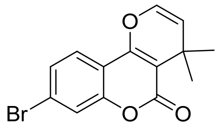

3k

^{1}H NMR (400 MHz, Chloroform-*d*)
^{13}C NMR (100 MHz, Chloroform-*d*)

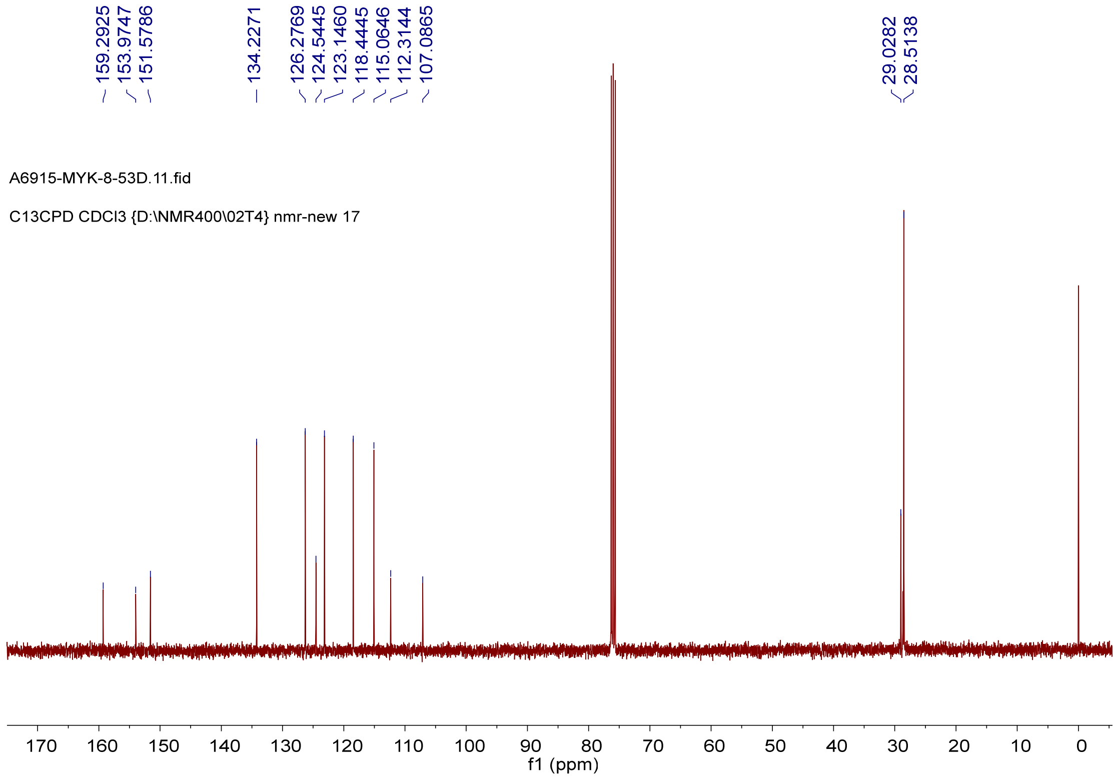

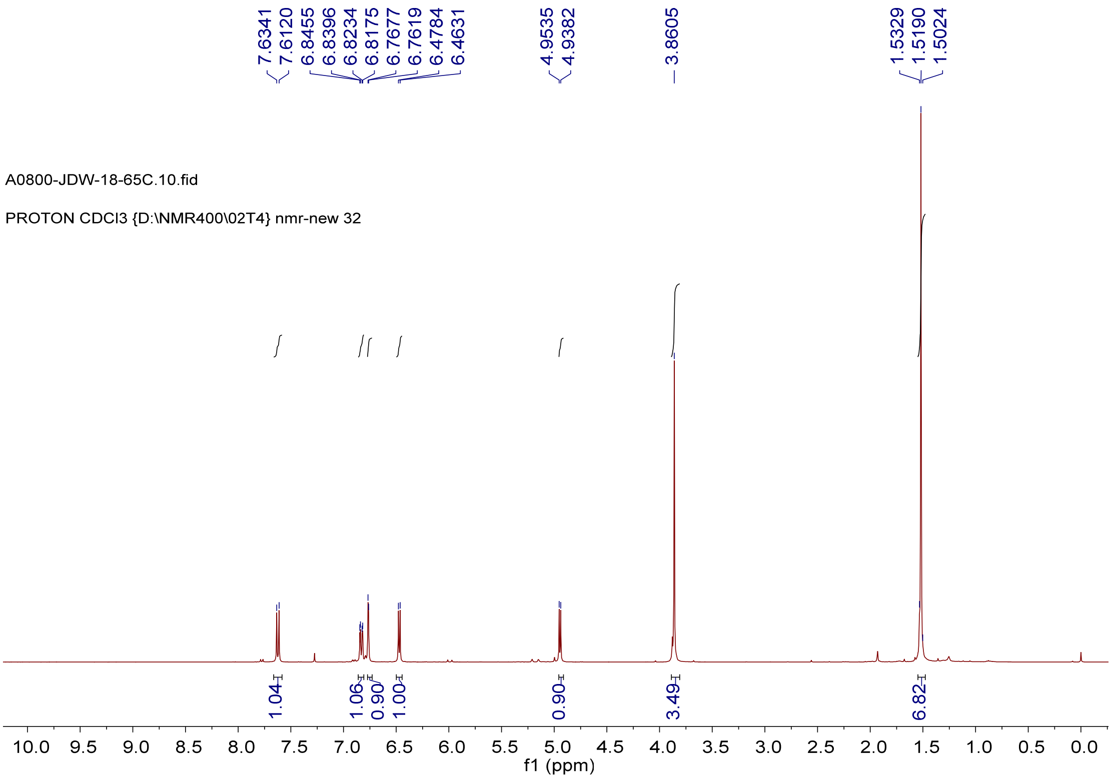

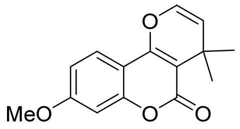

3l

^{1}H NMR (400 MHz, Chloroform-*d*)
^{13}C NMR (100 MHz, Chloroform-*d*)

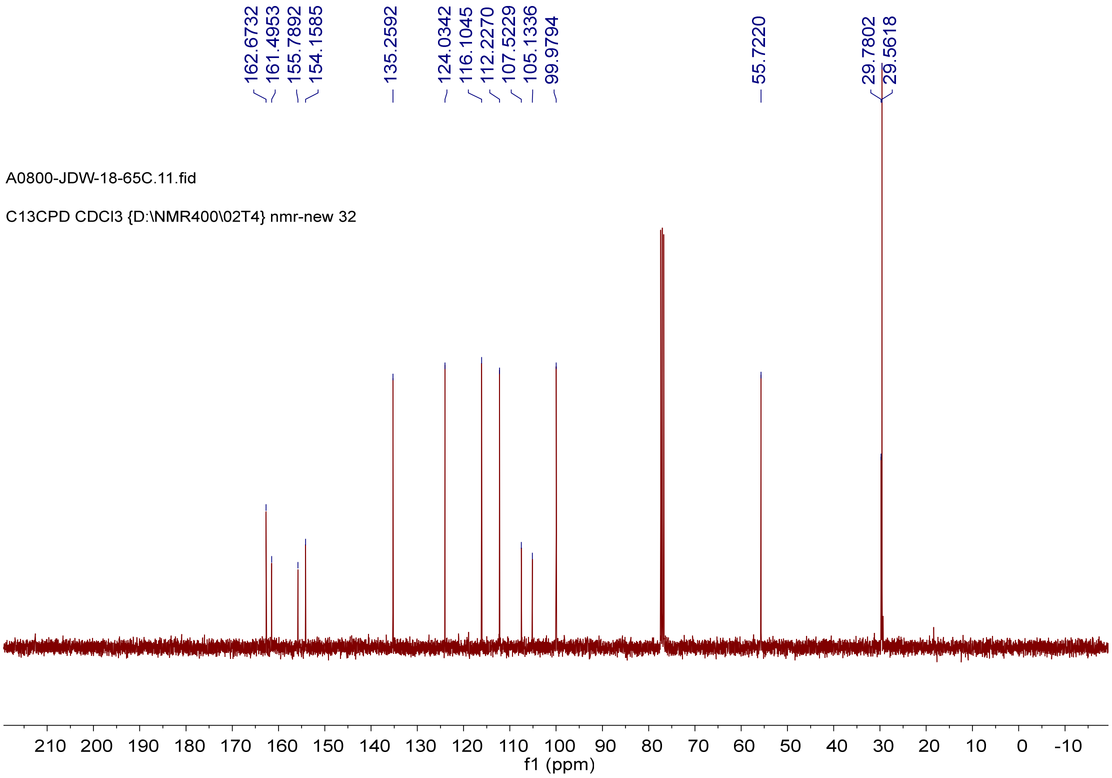

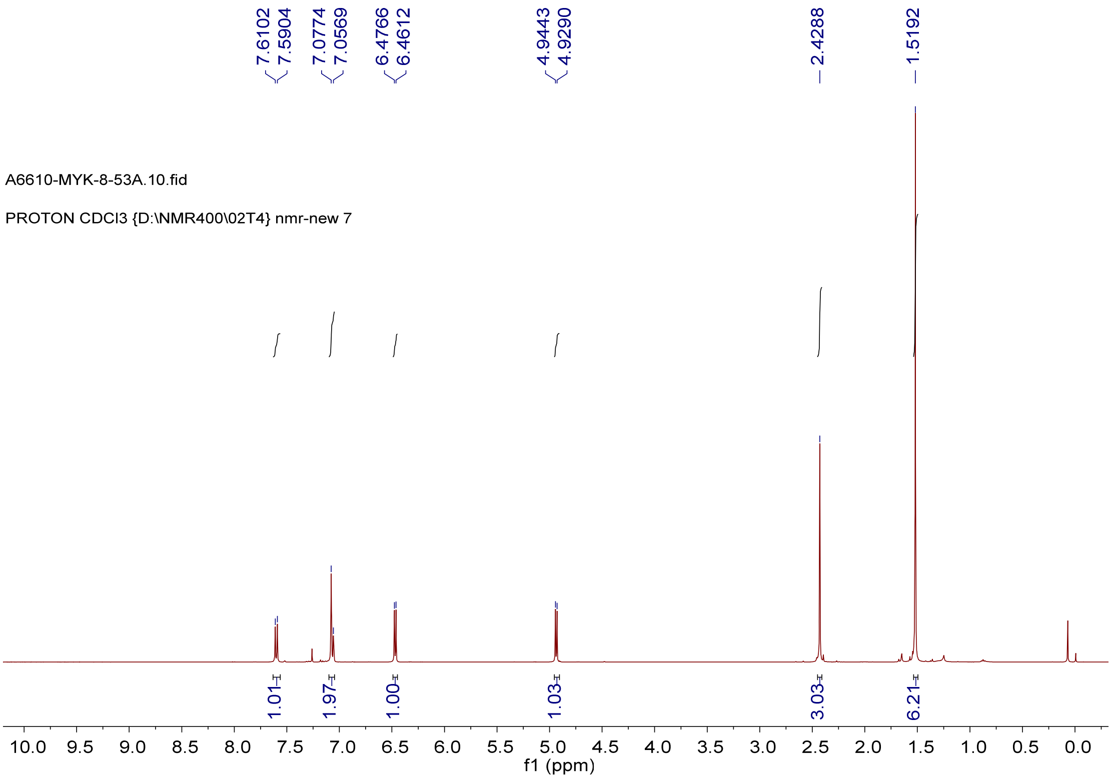

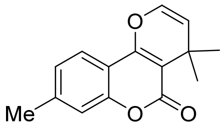

3m

^{1}H NMR (400 MHz, Chloroform-*d*)
^{13}C NMR (100 MHz, Chloroform-*d*)

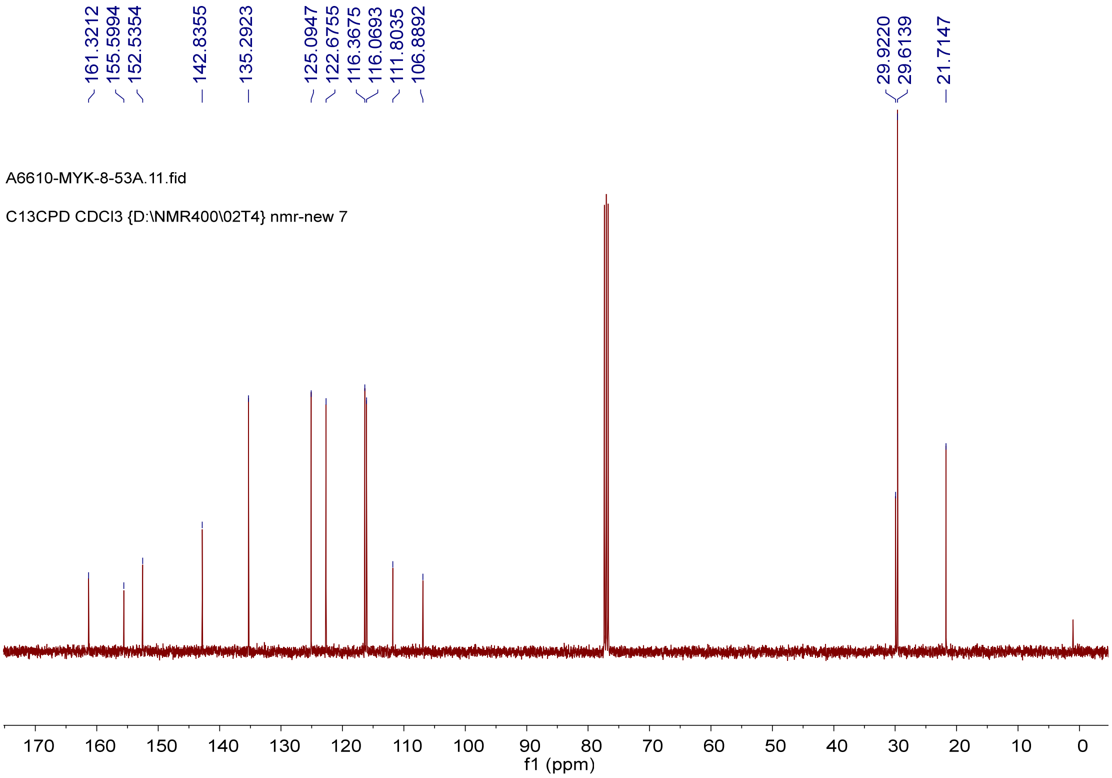

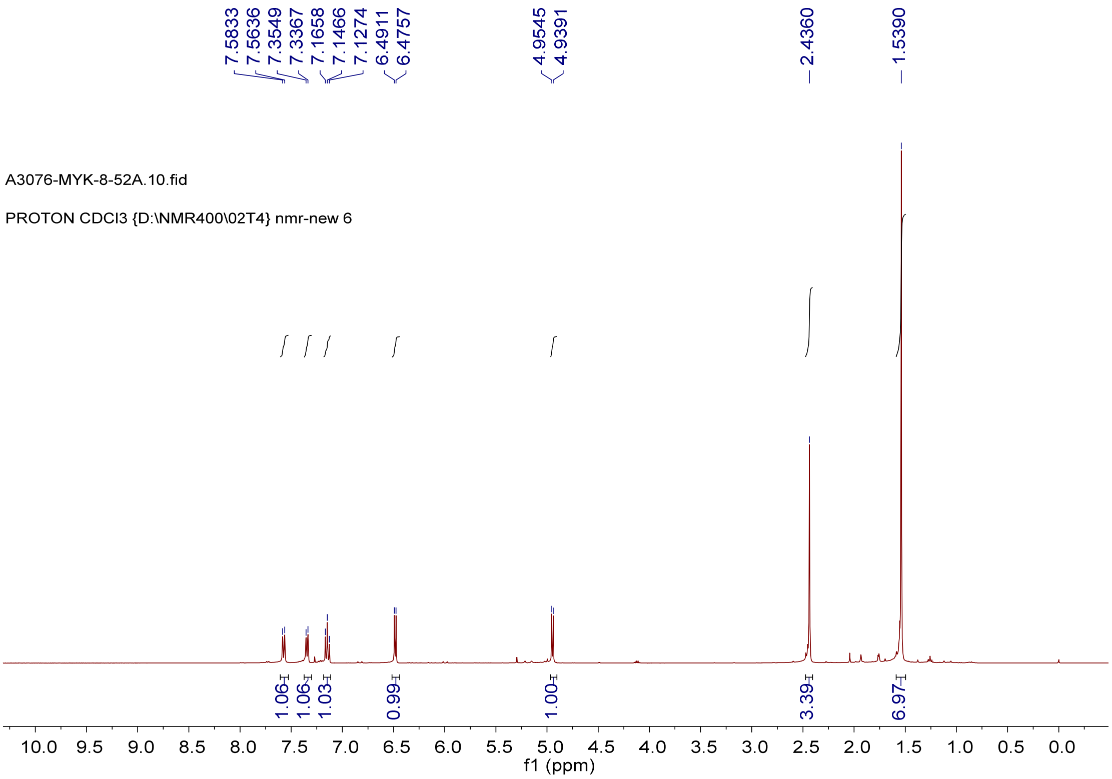

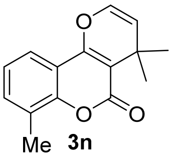

3n

^{1}**H NMR** (400 MHz, Chloroform-*d*)

^{13}**C NMR** (100 MHz, Chloroform-*d*)

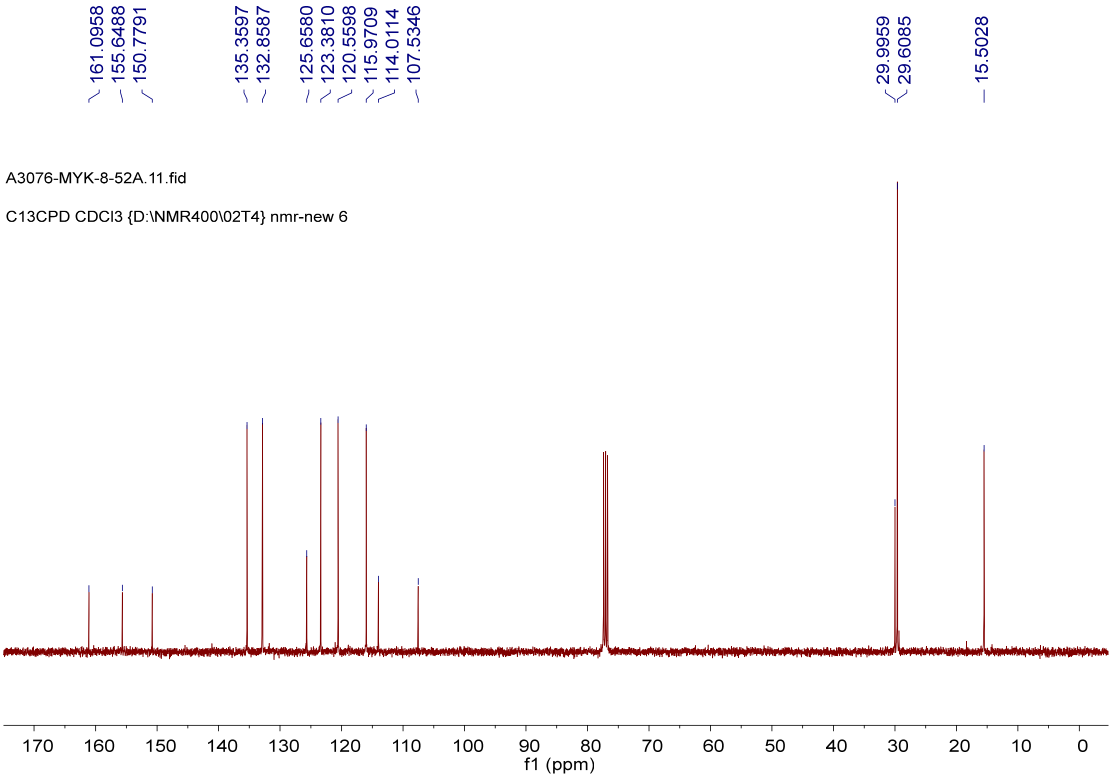

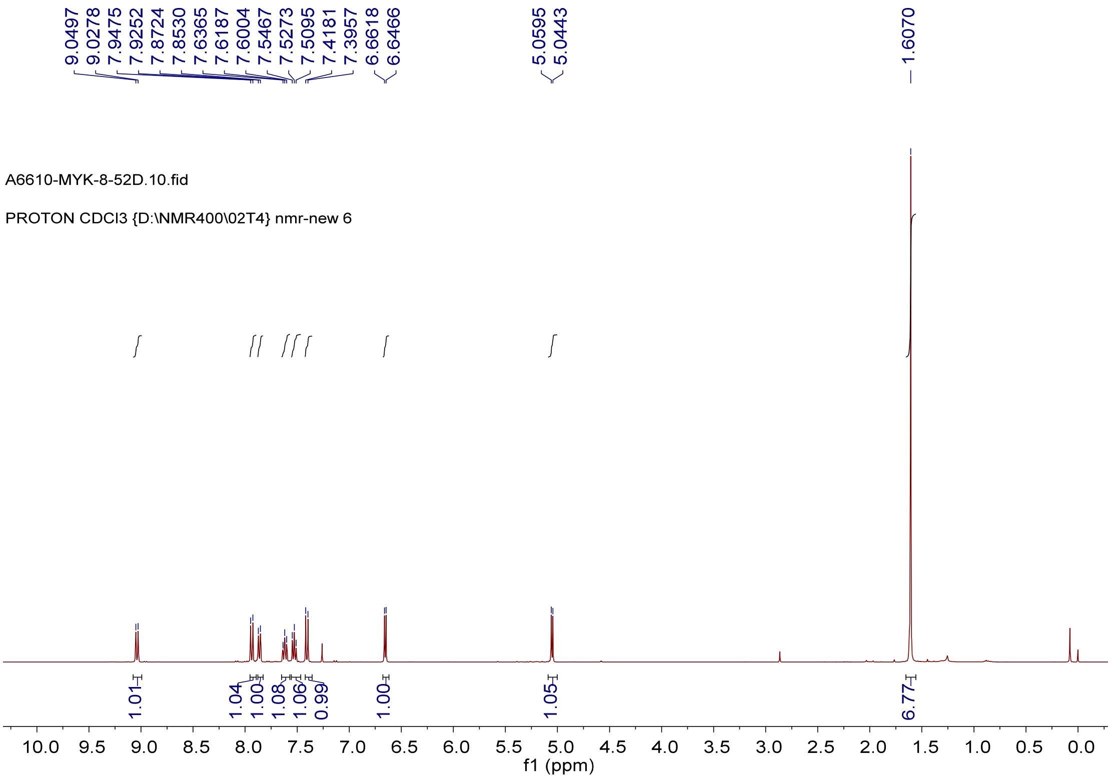

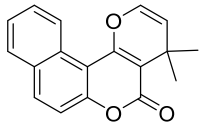

3o

^{1}H NMR (400 MHz, Chloroform-*d*)
^{13}C NMR (100 MHz, Chloroform-*d*)

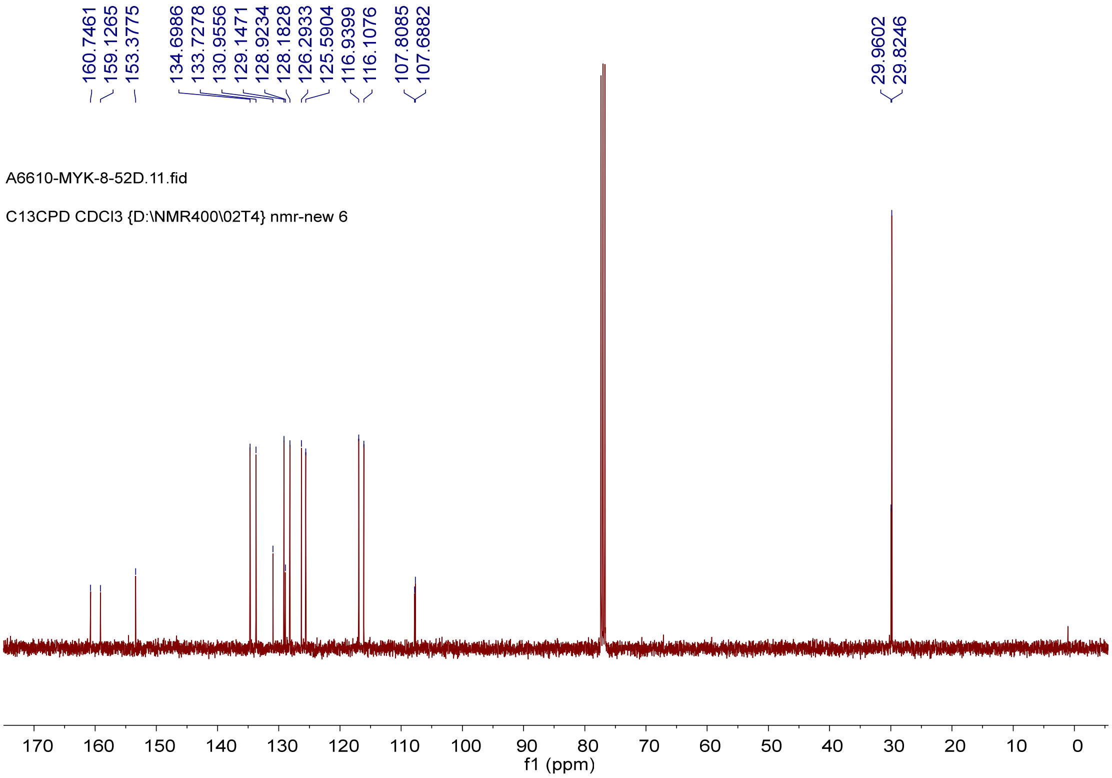

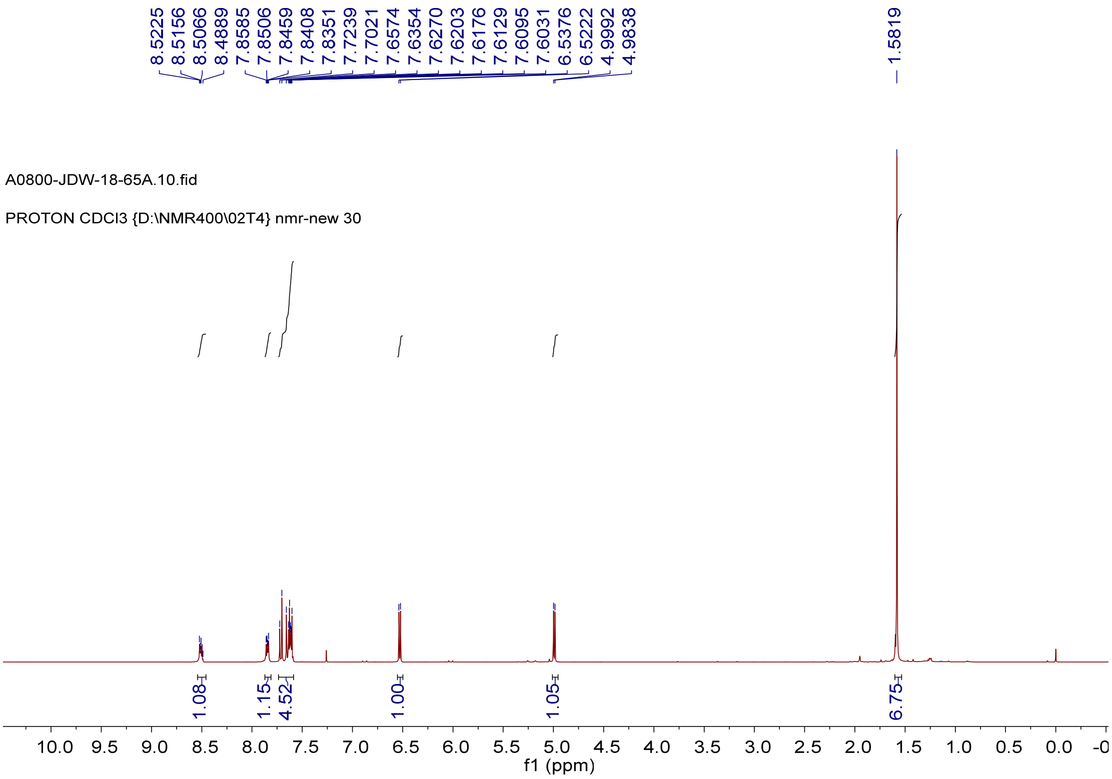

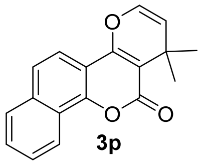

3p

^{1}H NMR (400 MHz, Chloroform-*d*)
^{13}C NMR (100 MHz, Chloroform-*d*)

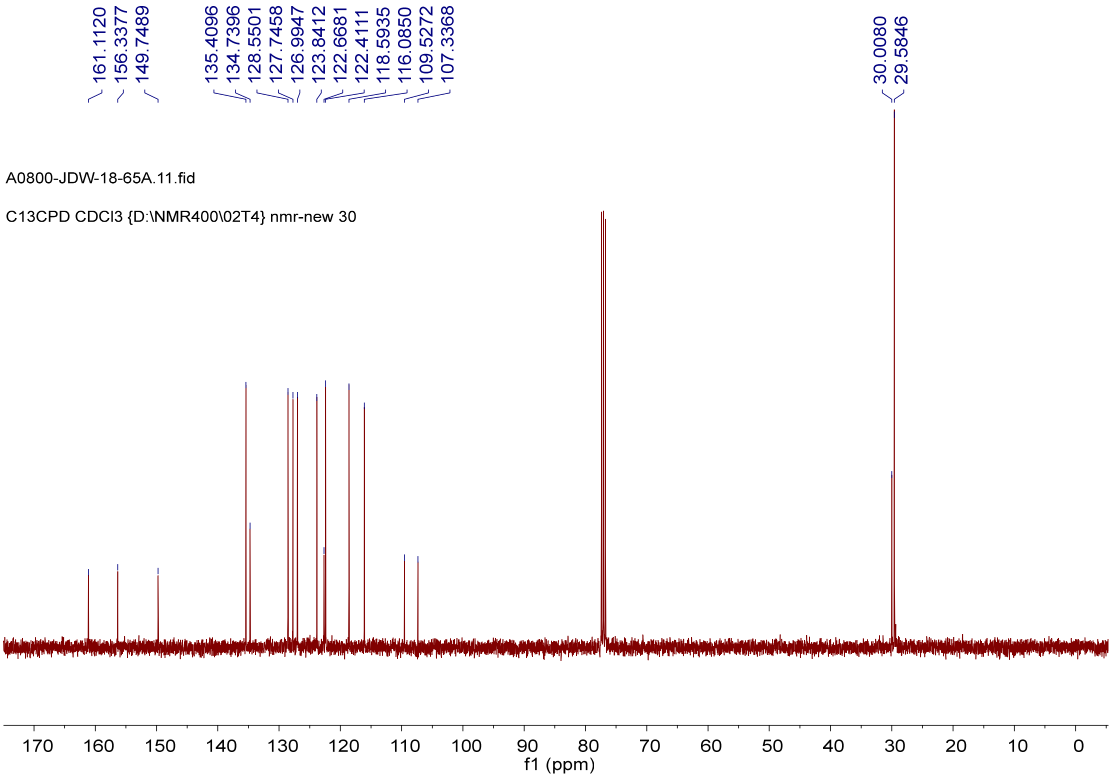

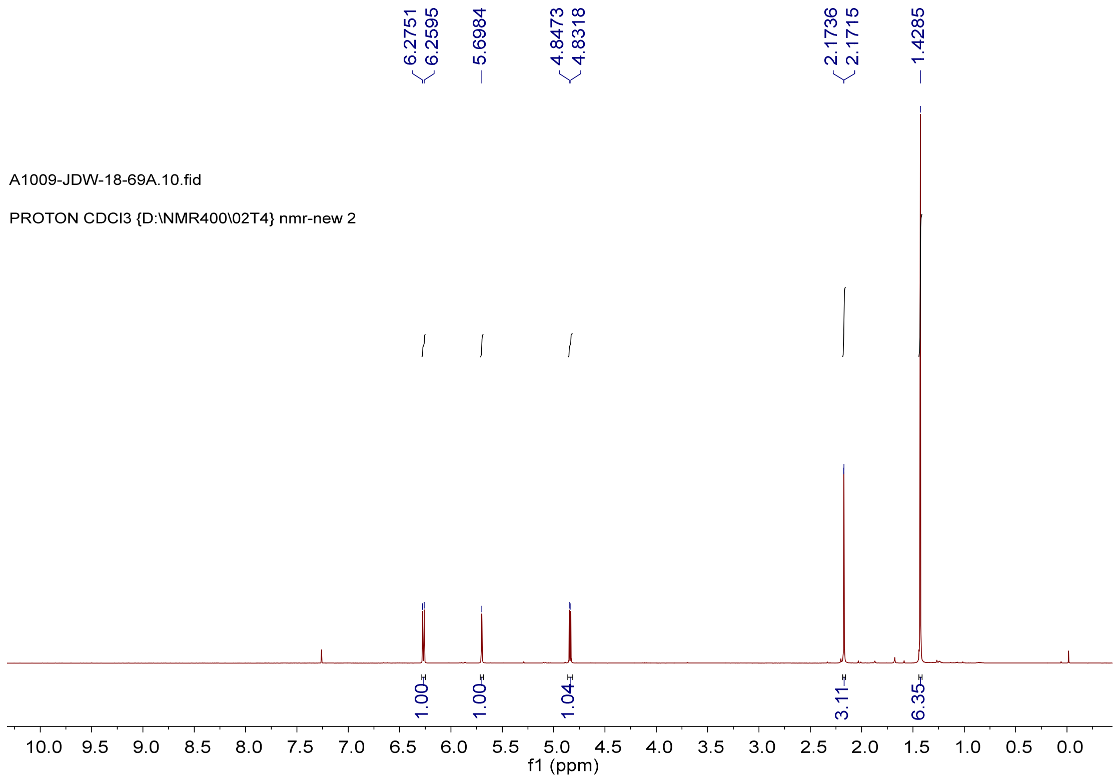

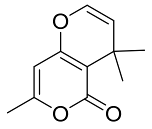

3r

^{1}H NMR (400 MHz, Chloroform-*d*)
^{13}C NMR (100 MHz, Chloroform-*d*)

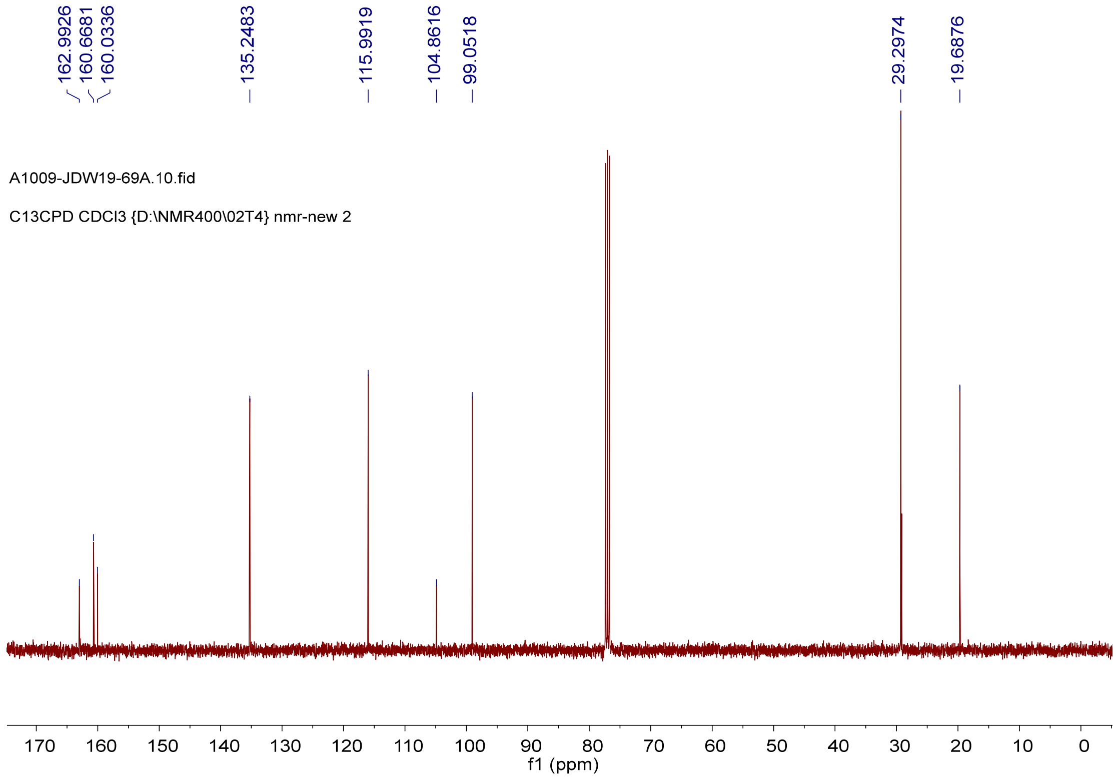

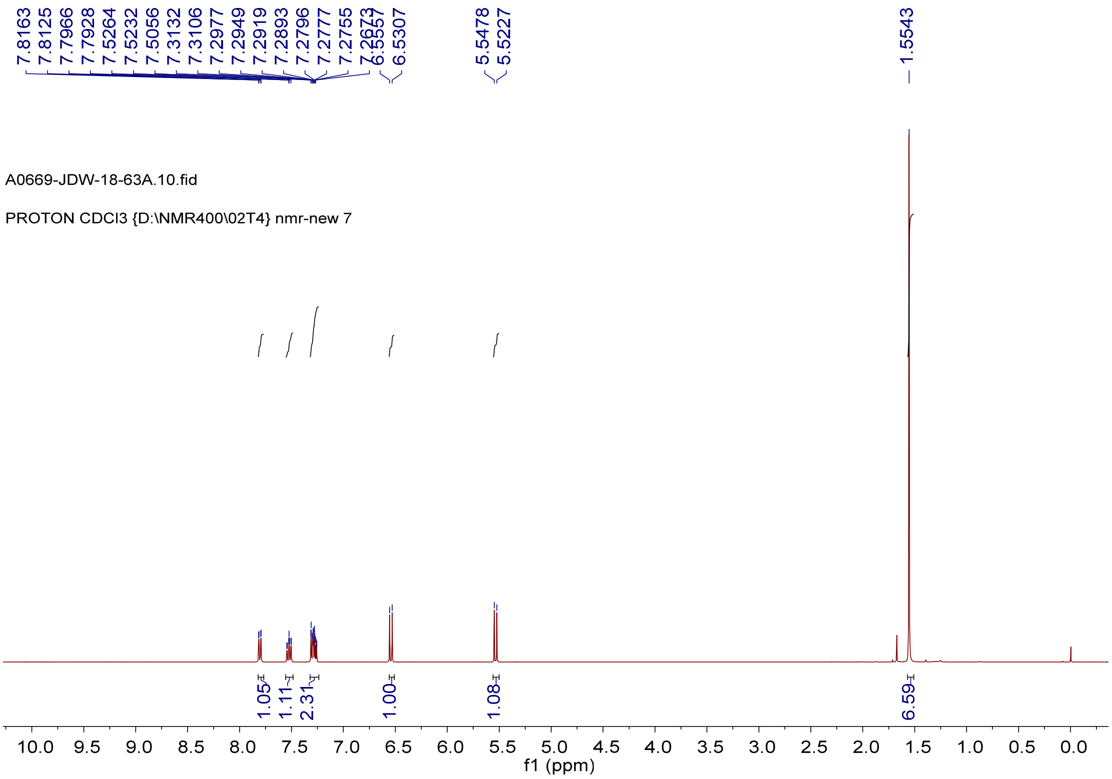

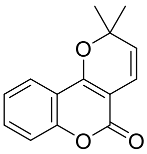

4a

^{1}H NMR (400 MHz, Chloroform-*d*)
^{13}C NMR (100 MHz, Chloroform-*d*)

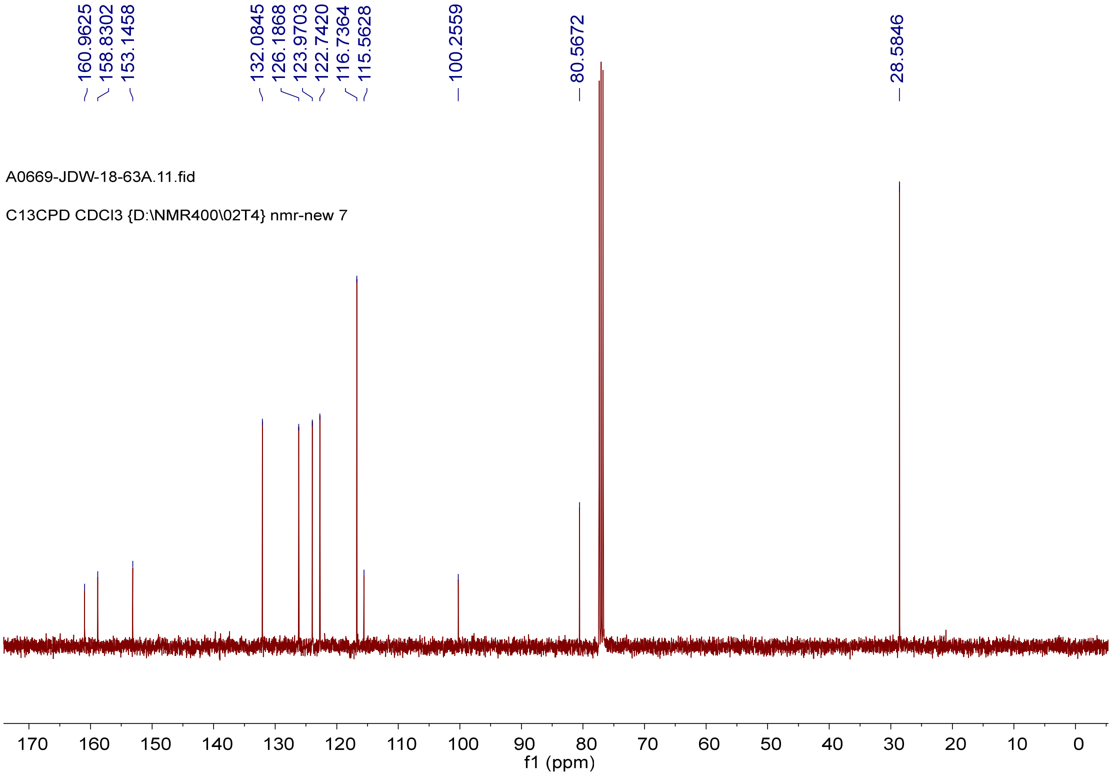

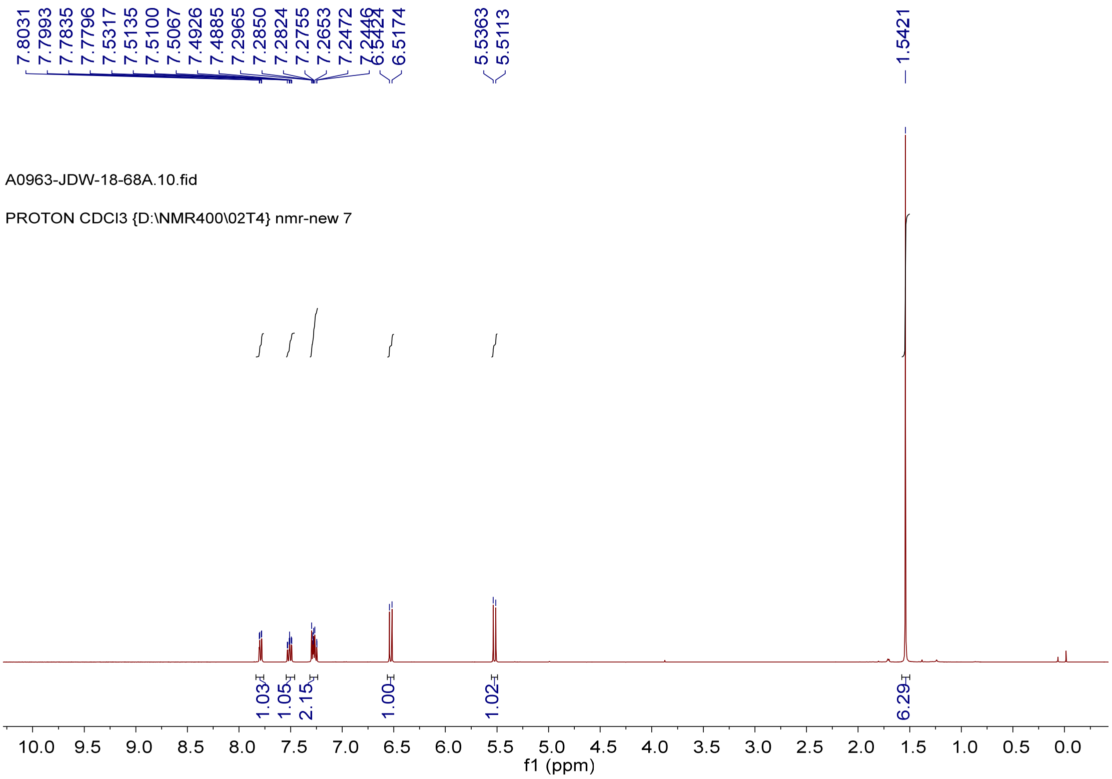

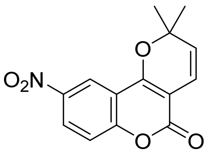

4b

^{1}H NMR (400 MHz, Chloroform-*d*)
^{13}C NMR (100 MHz, Chloroform-*d*)

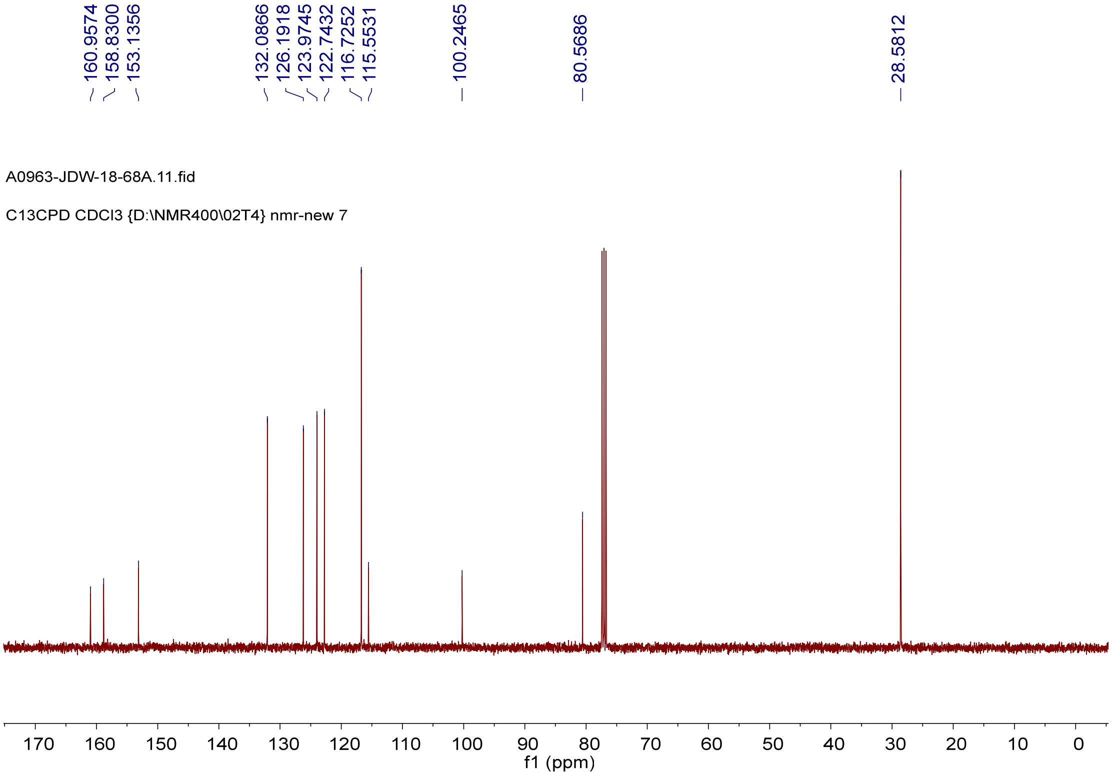

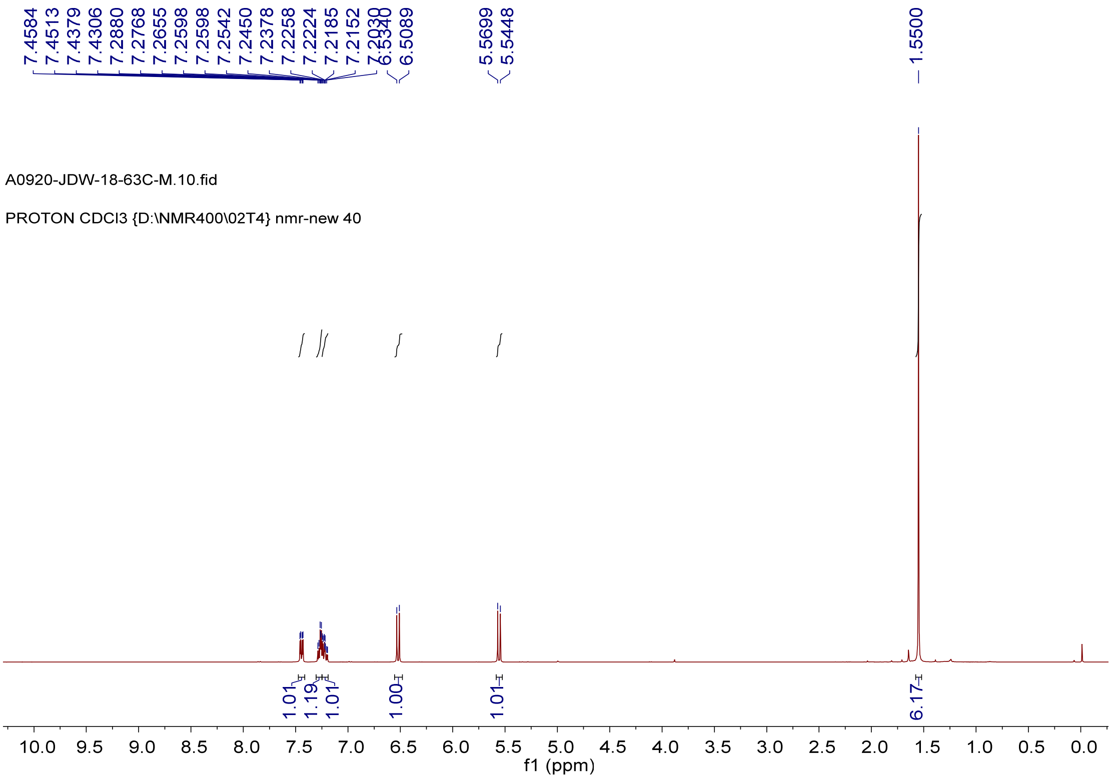

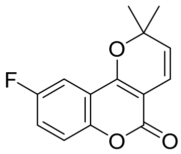

4c

^{1}H NMR (400 MHz, Chloroform-*d*)
^{13}C NMR (100 MHz, Chloroform-*d*)

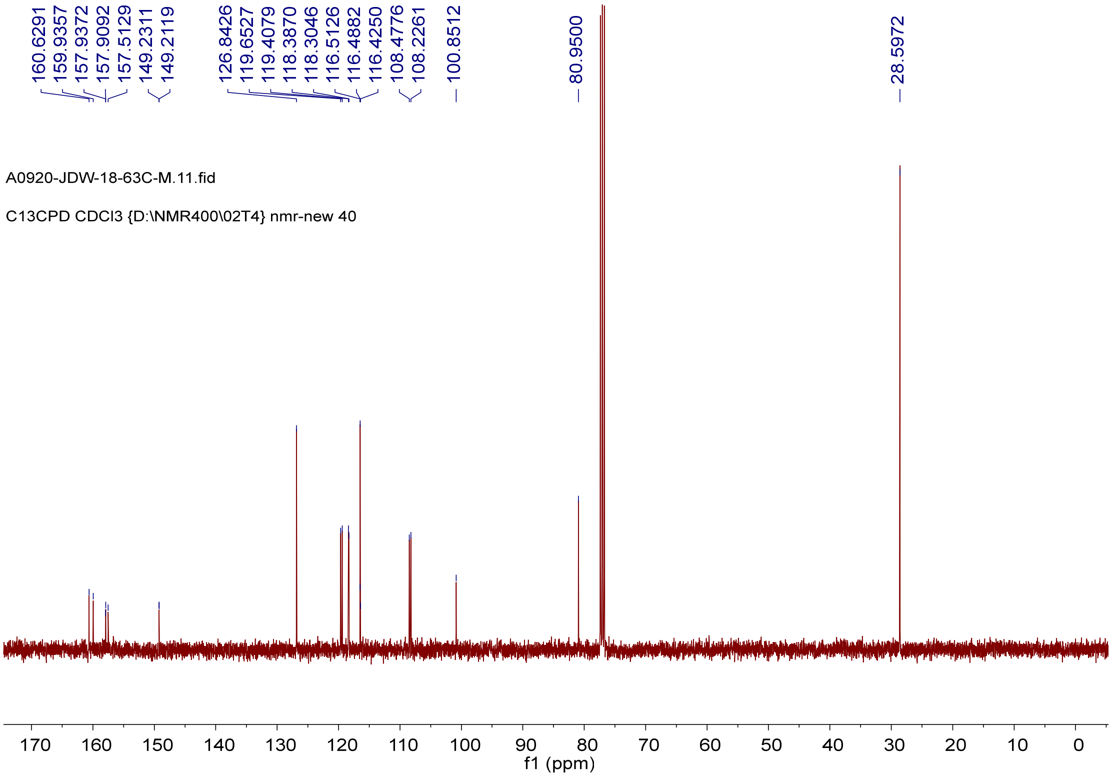

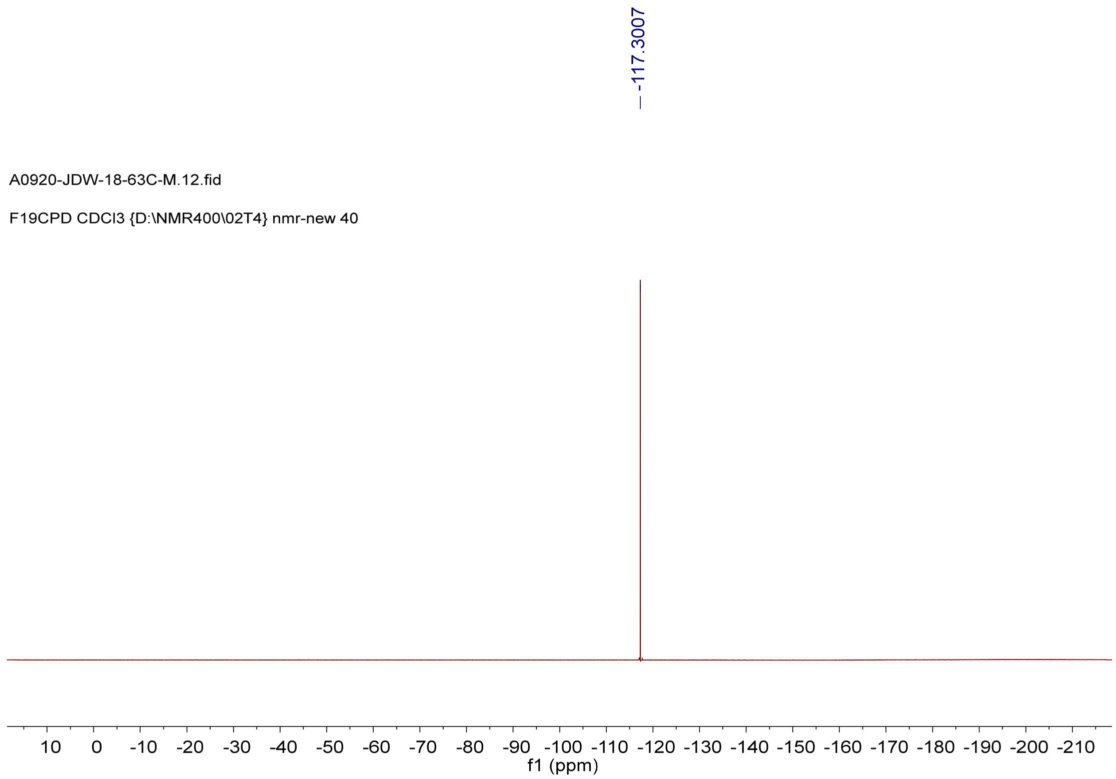

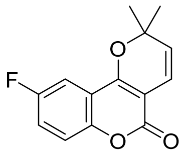

4c

^{19}F NMR (375 MHz, Chloroform-*d*)

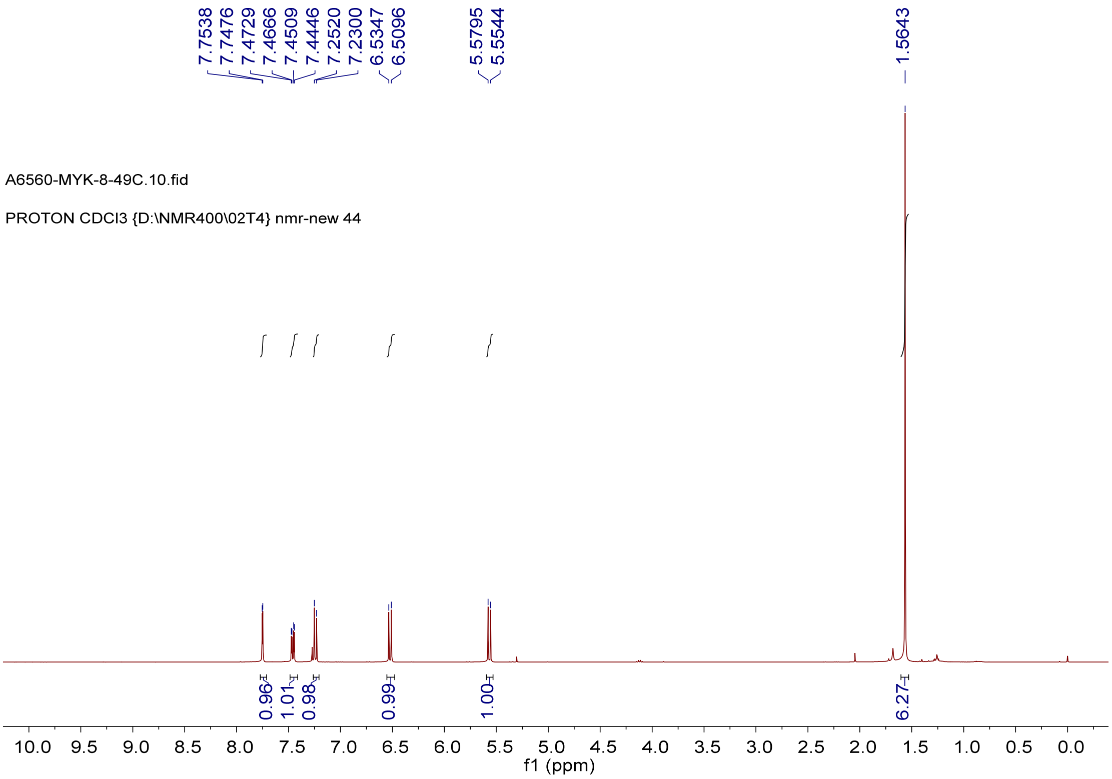

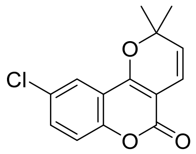

4d

^{1}H NMR (400 MHz, Chloroform-*d*)
^{13}C NMR (100 MHz, Chloroform-*d*)

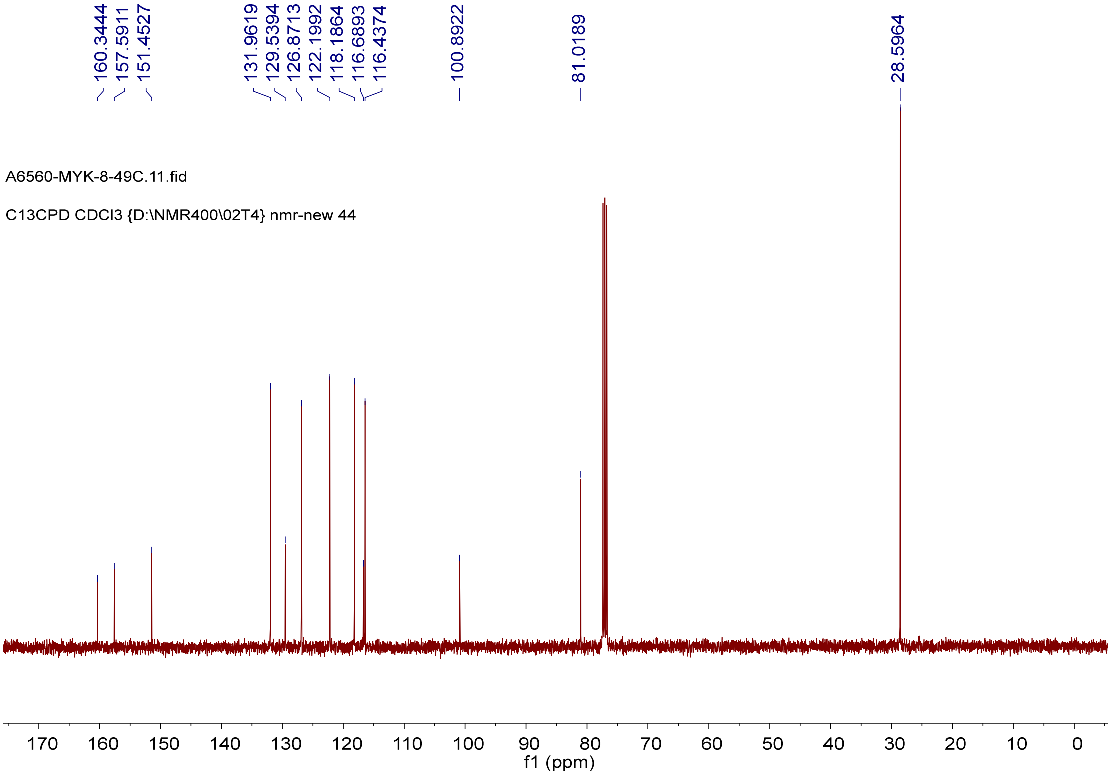

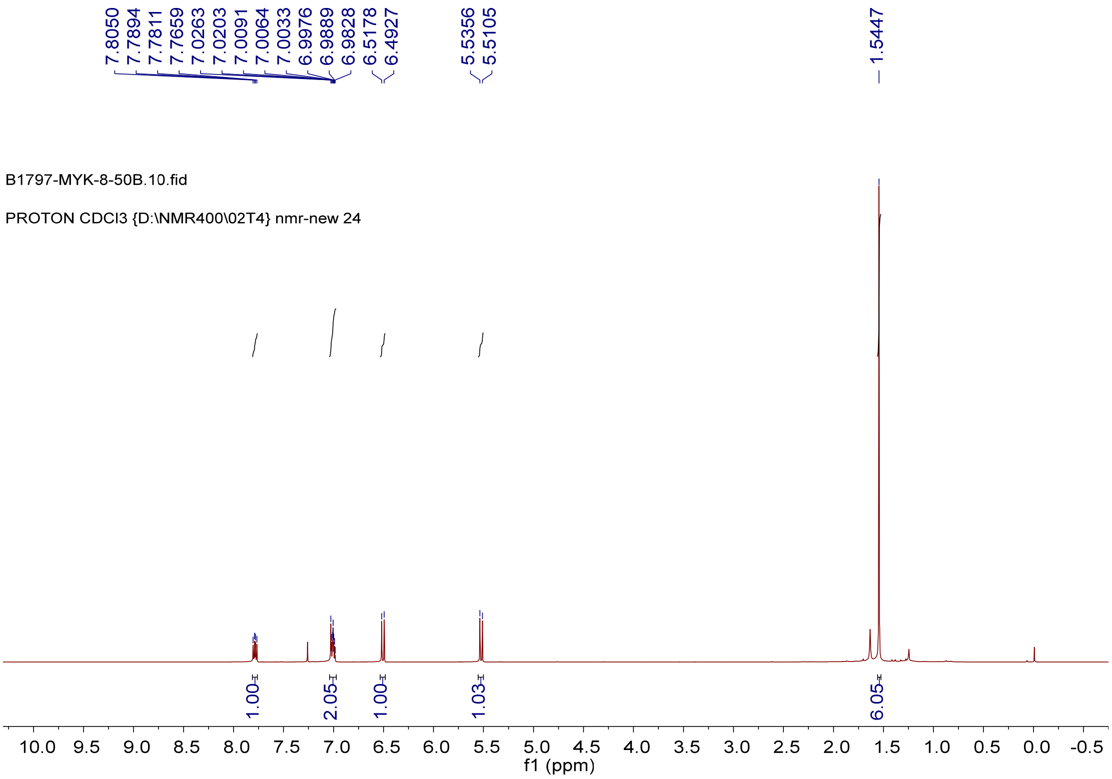

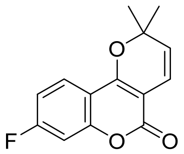

4e

^{1}H NMR (400 MHz, Chloroform-*d*)
^{13}C NMR (100 MHz, Chloroform-*d*)

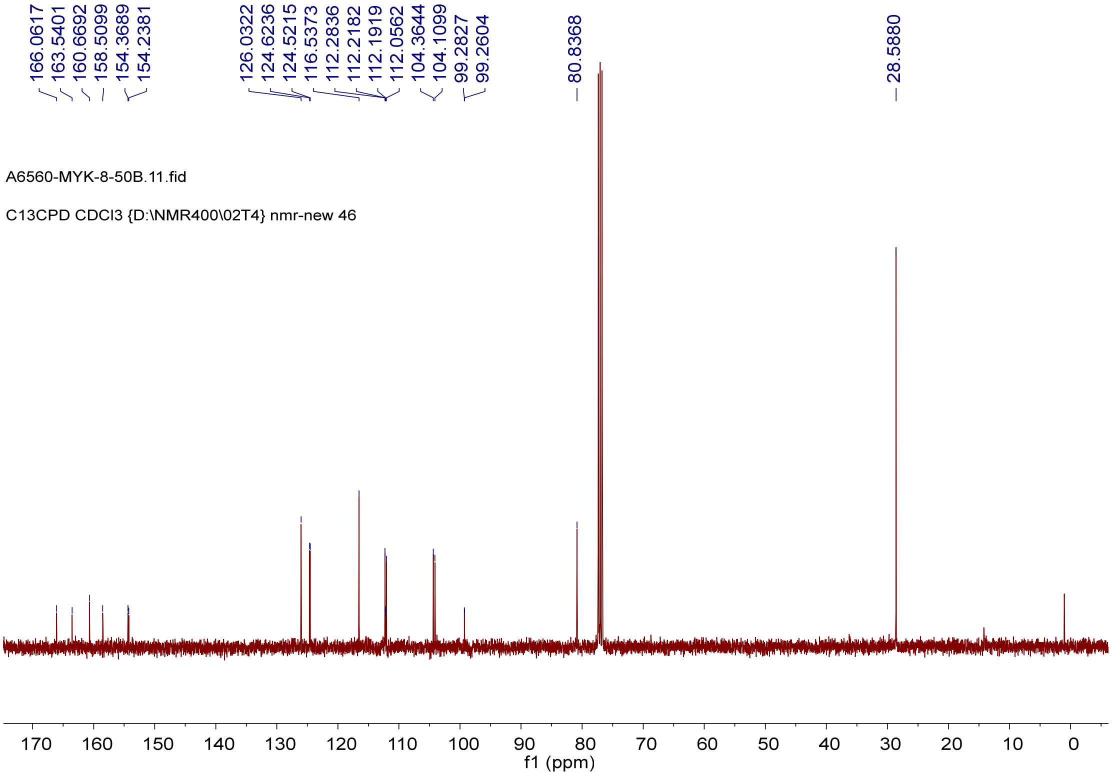

A3543-MYK-8-50B.10.fid

F19CPD CDCl3 {D:\NMR400\02T4} nmr-new 37

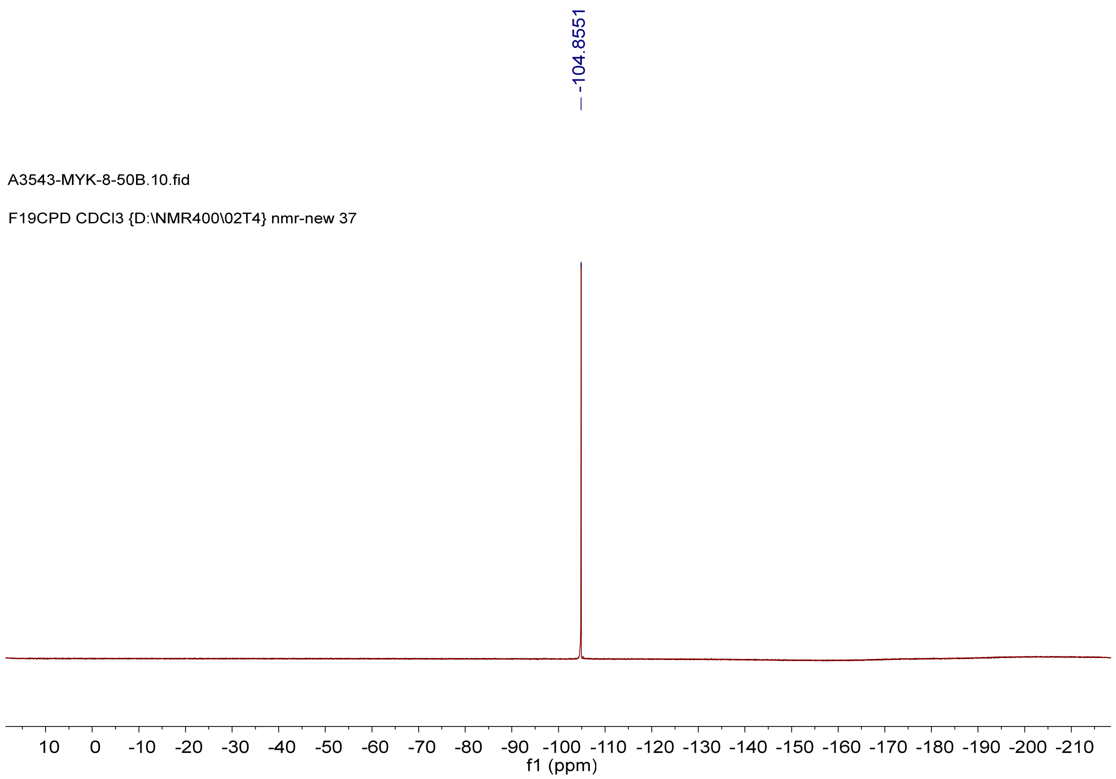

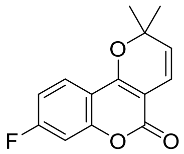

4e

^{19}F NMR (375 MHz, Chloroform-*d*)

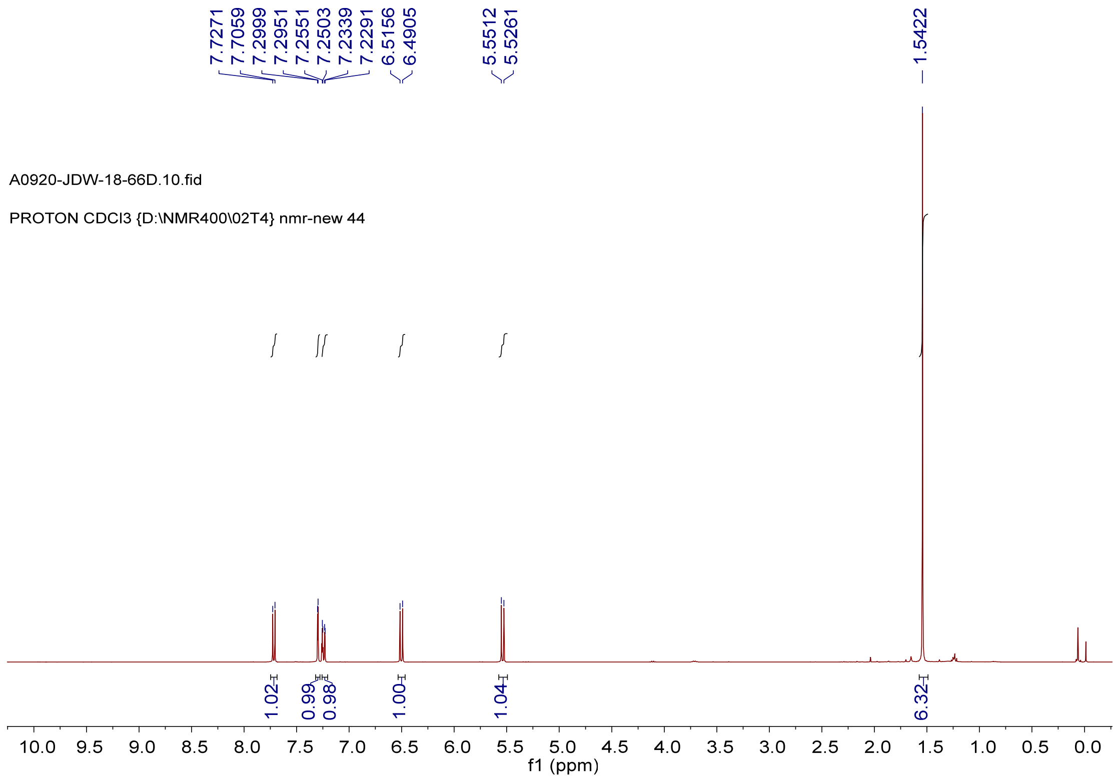

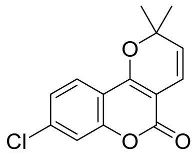

4f

^{1}H NMR (400 MHz, Chloroform-*d*)
^{13}C NMR (100 MHz, Chloroform-*d*)

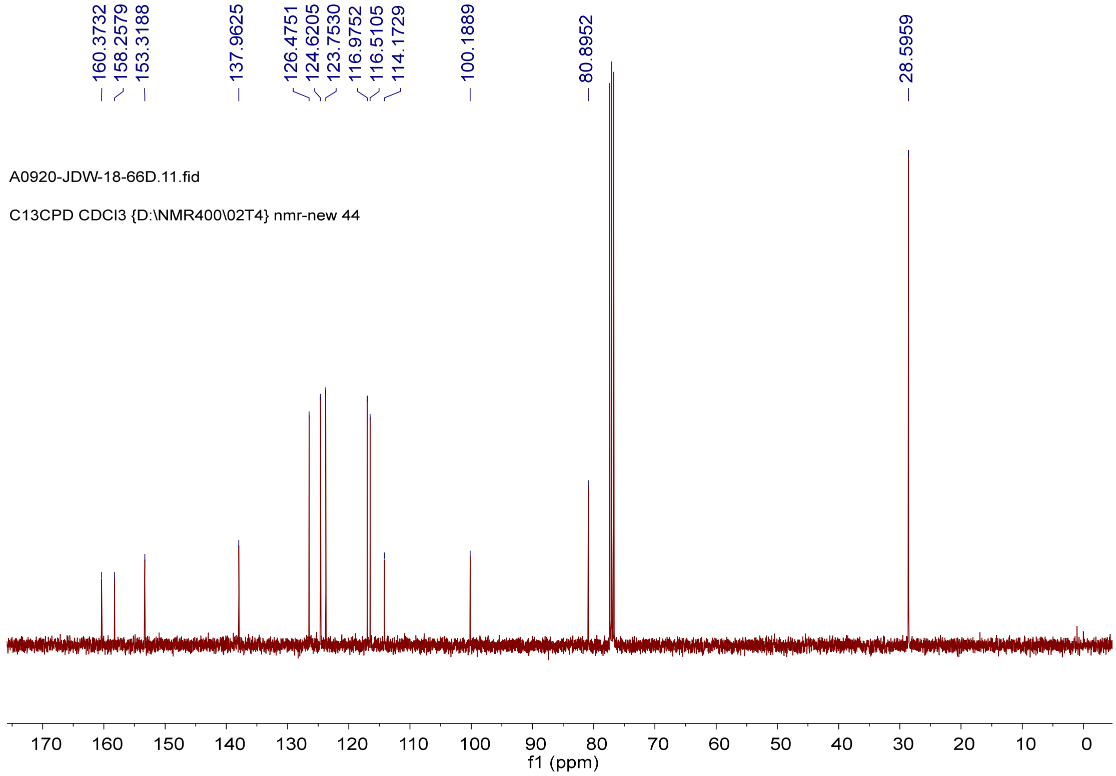

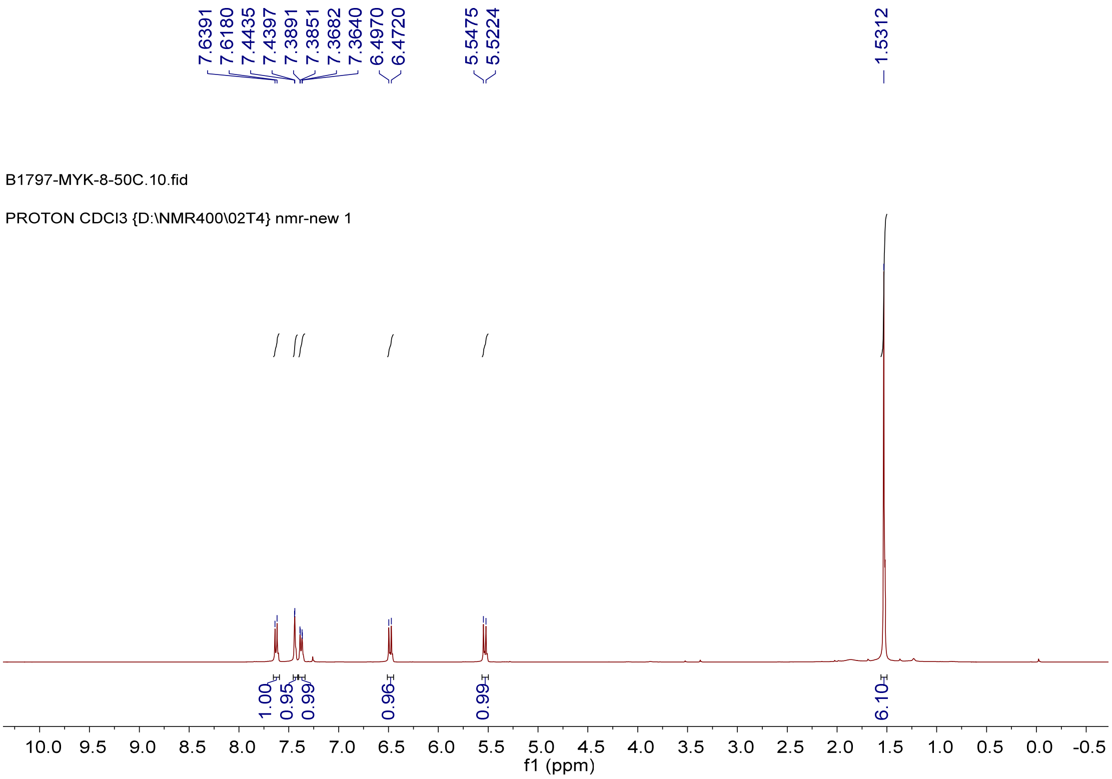

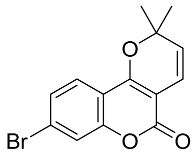

4g

^{1}H NMR (400 MHz, Chloroform-*d*)
^{13}C NMR (100 MHz, Chloroform-*d*)

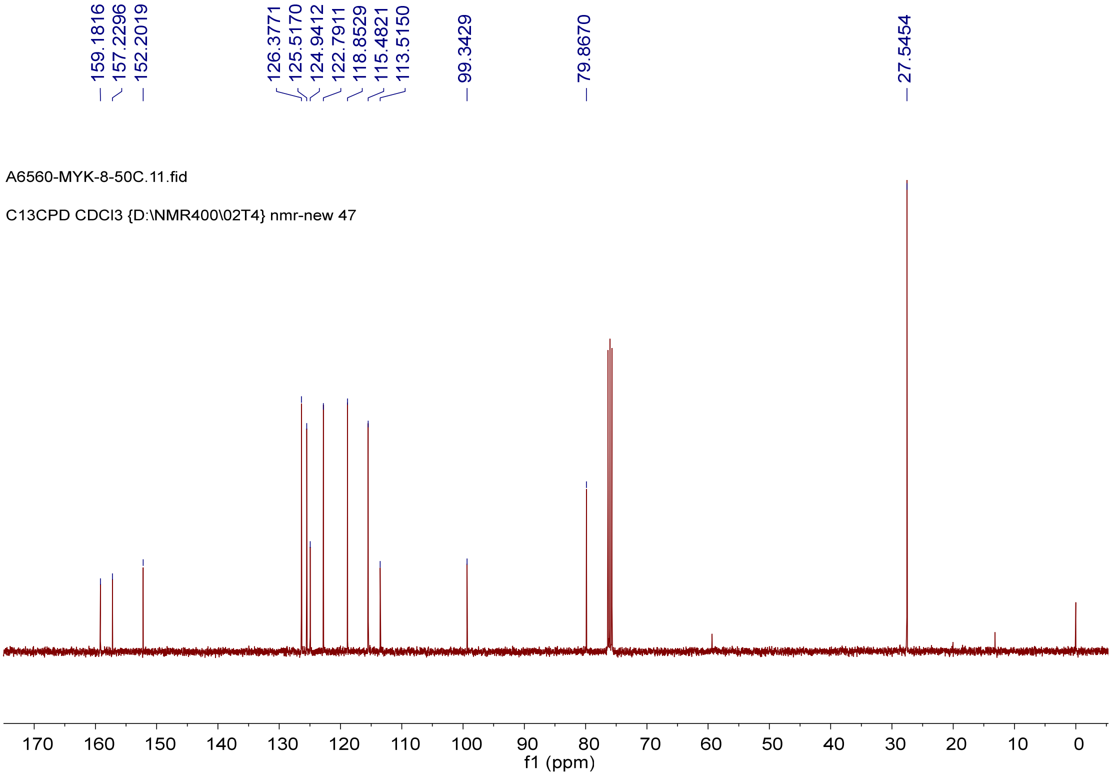

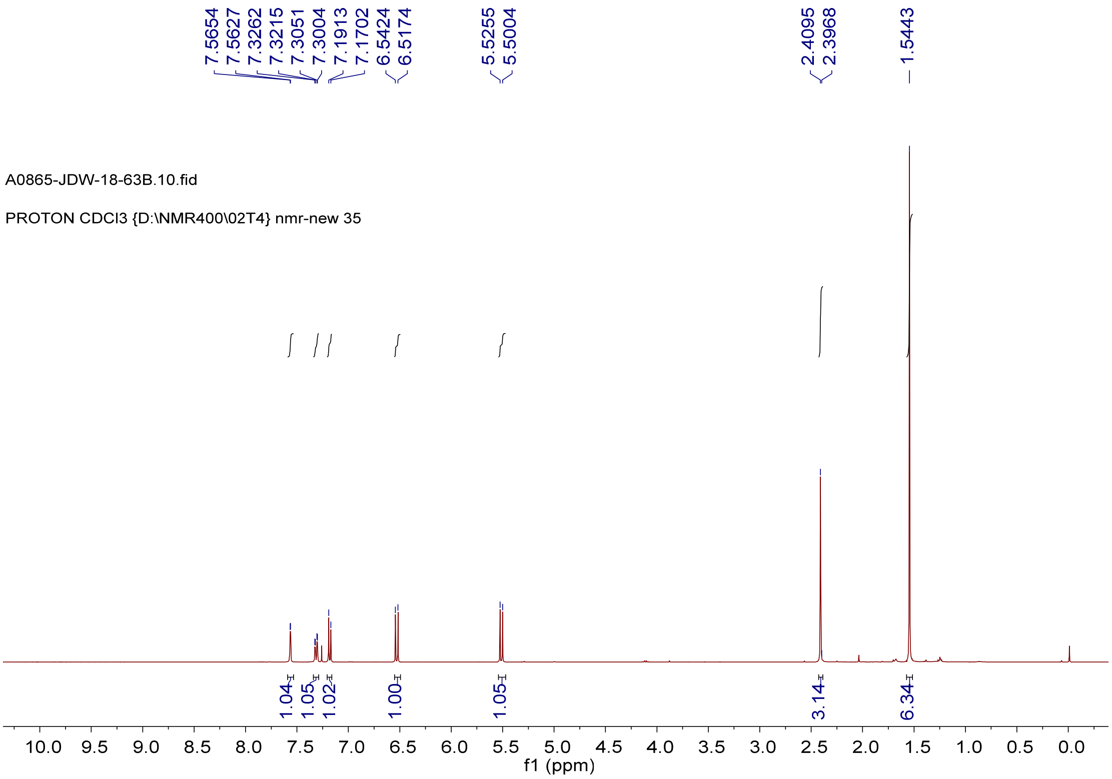

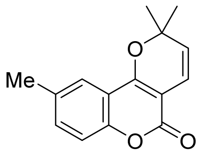

4h

^{1}H NMR (400 MHz, Chloroform-*d*)
^{13}C NMR (100 MHz, Chloroform-*d*)

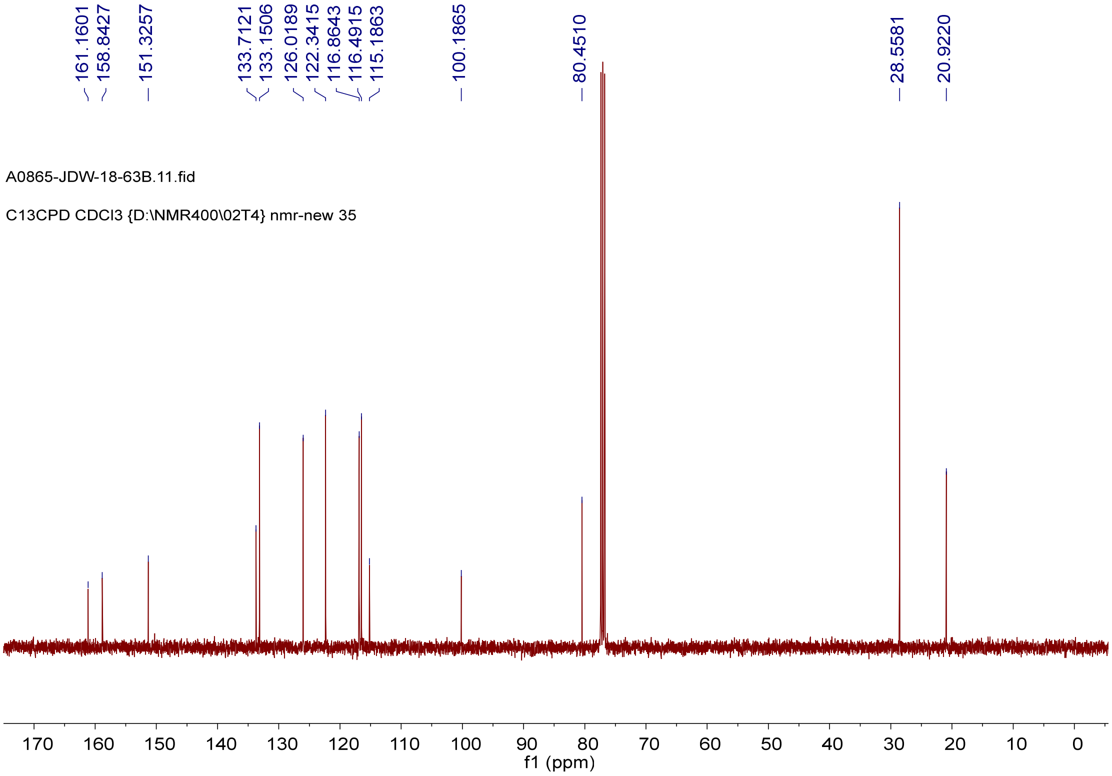

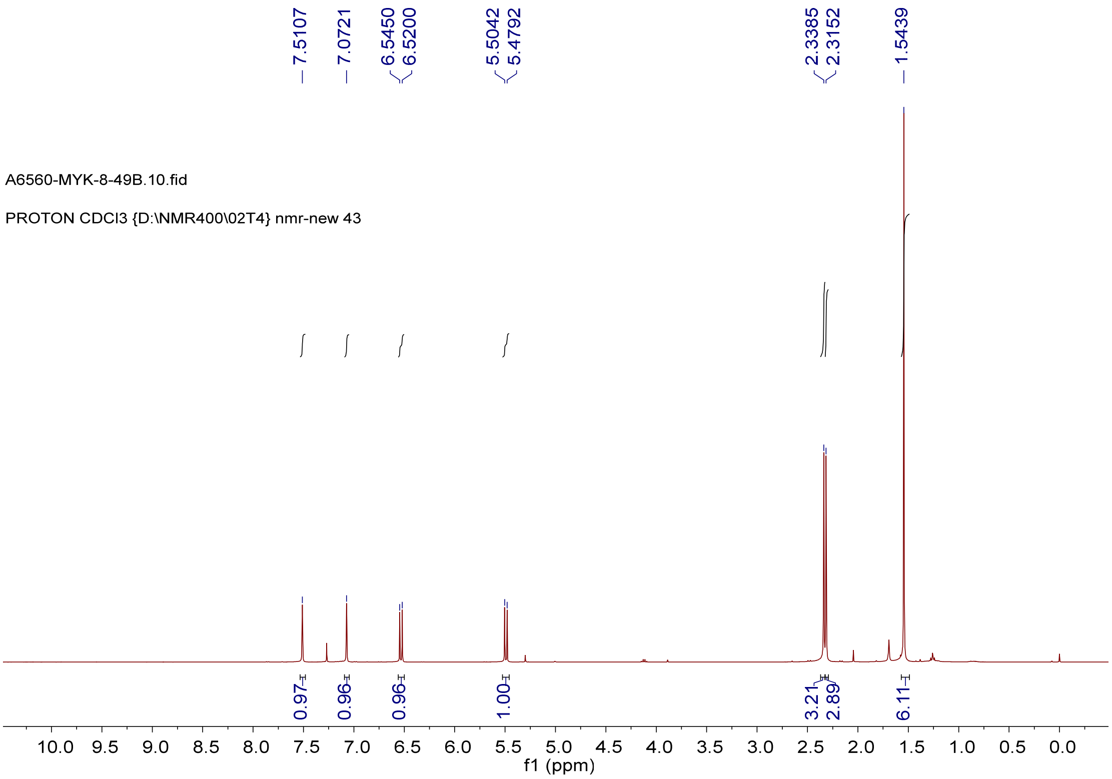

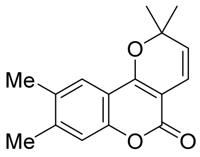

4i

^{1}H NMR (400 MHz, Chloroform-*d*)

^{13}C NMR (100 MHz, Chloroform-*d*)

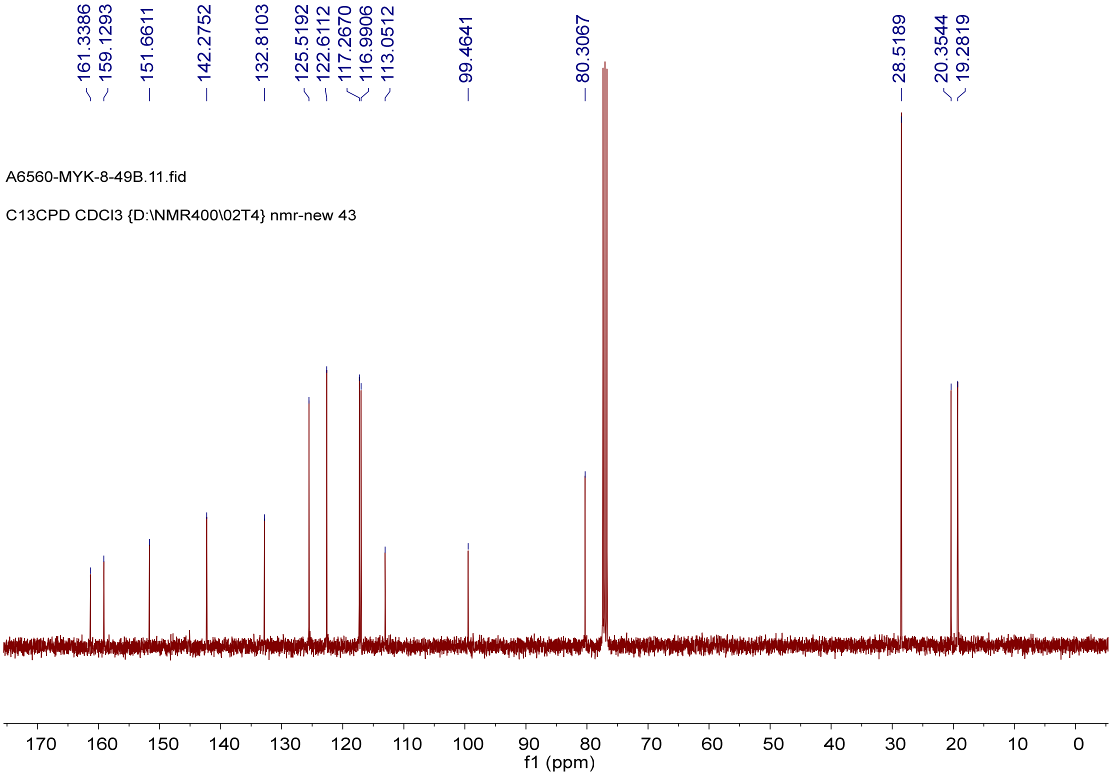

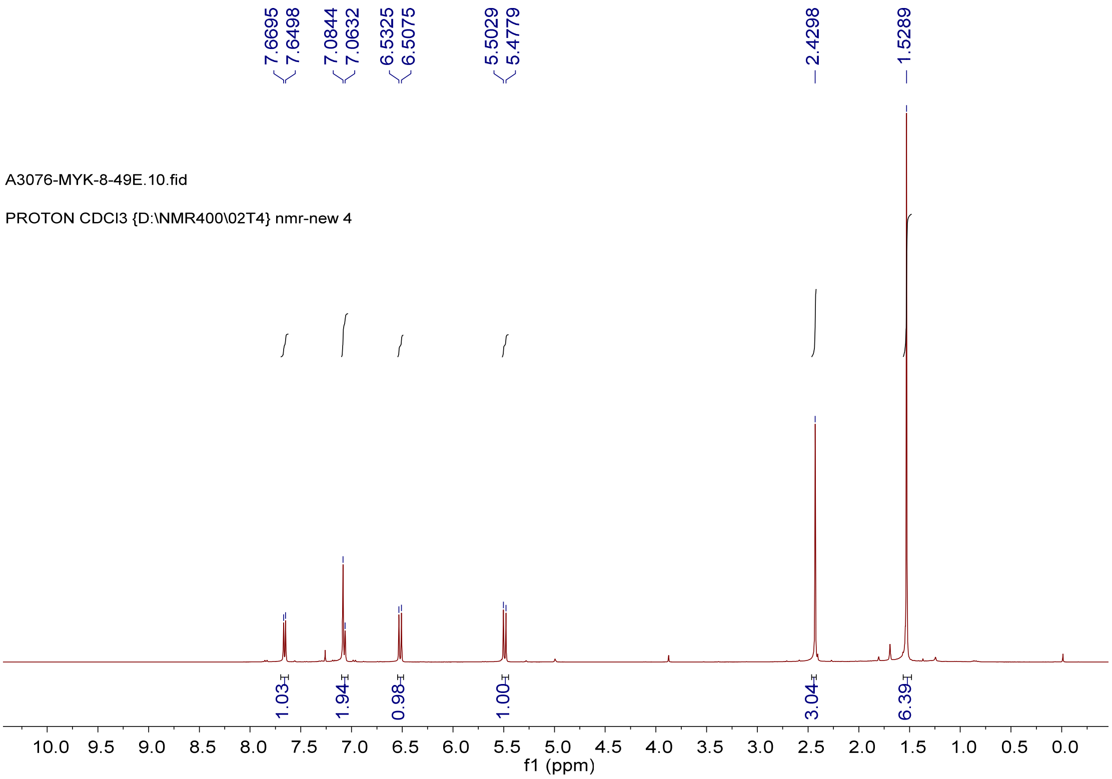

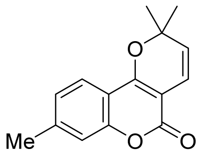

4j

^{1}H NMR (400 MHz, Chloroform-*d*)
^{13}C NMR (100 MHz, Chloroform-*d*)

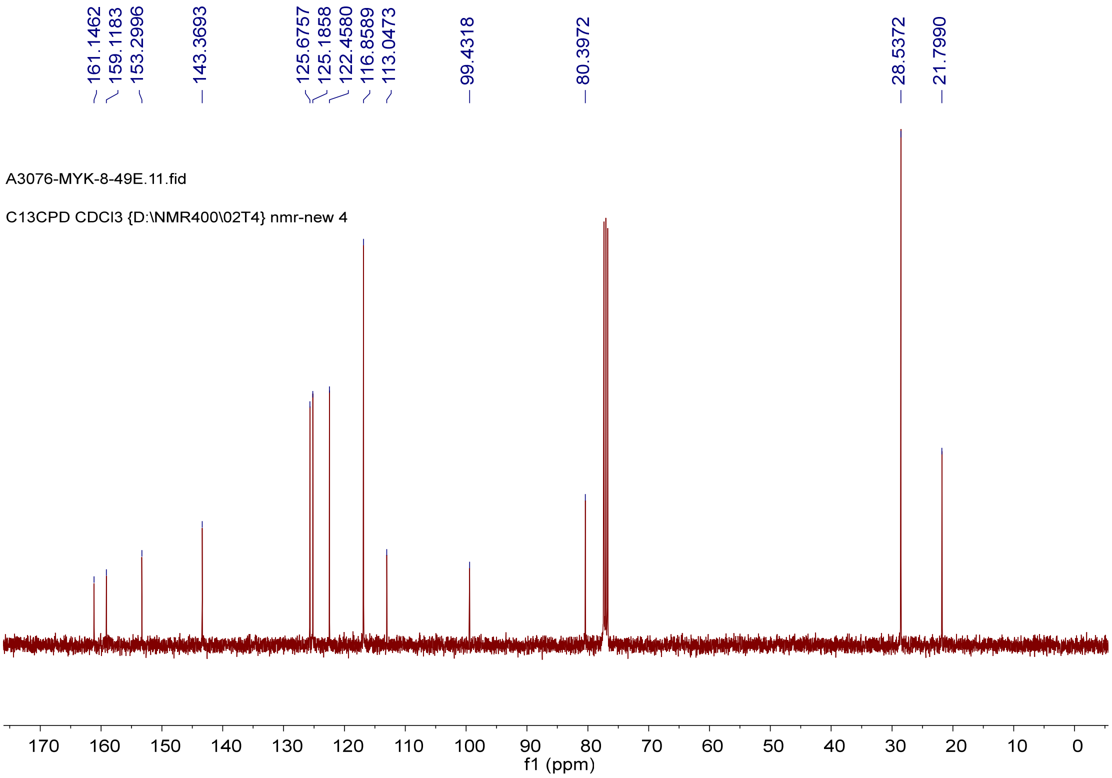

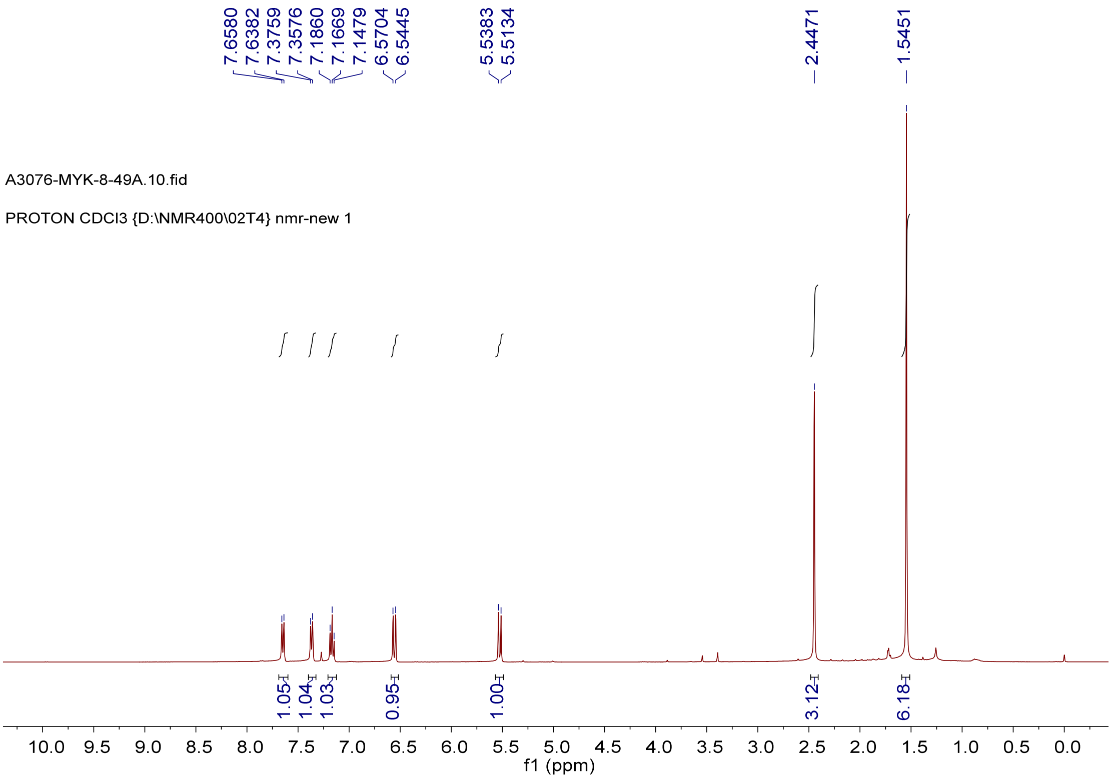

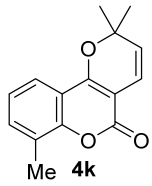

4k

^{1}H NMR (400 MHz, Chloroform-*d*)
^{13}C NMR (100 MHz, Chloroform-*d*)

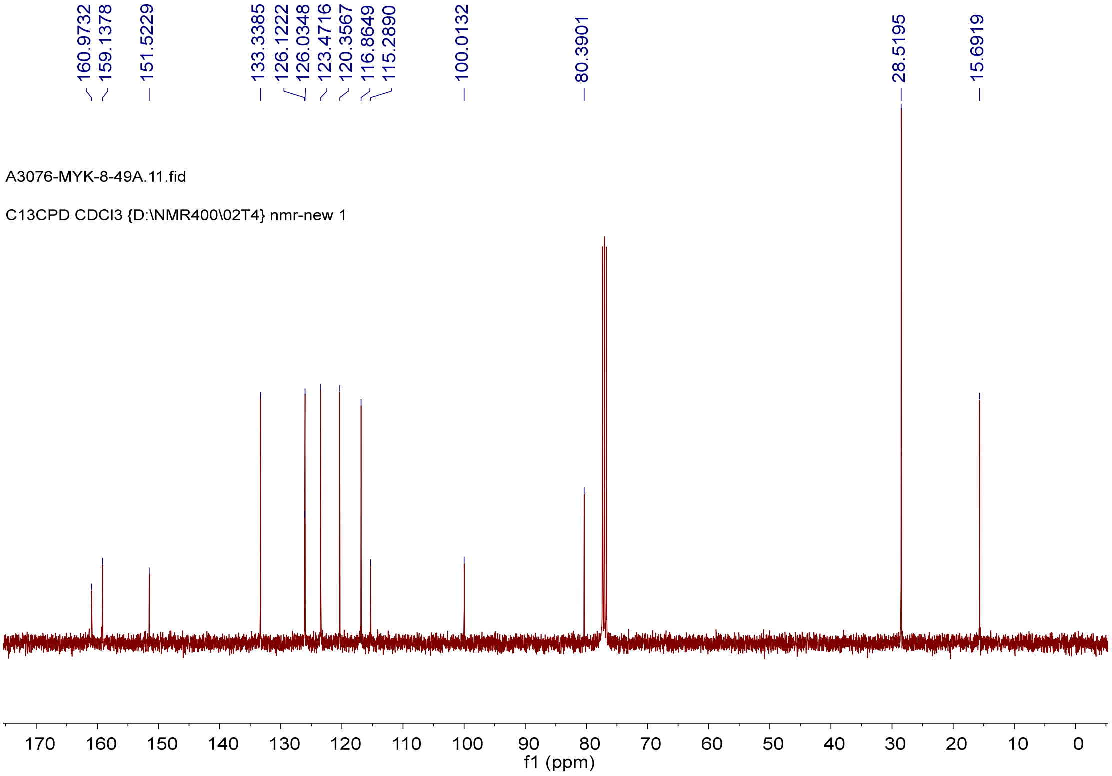

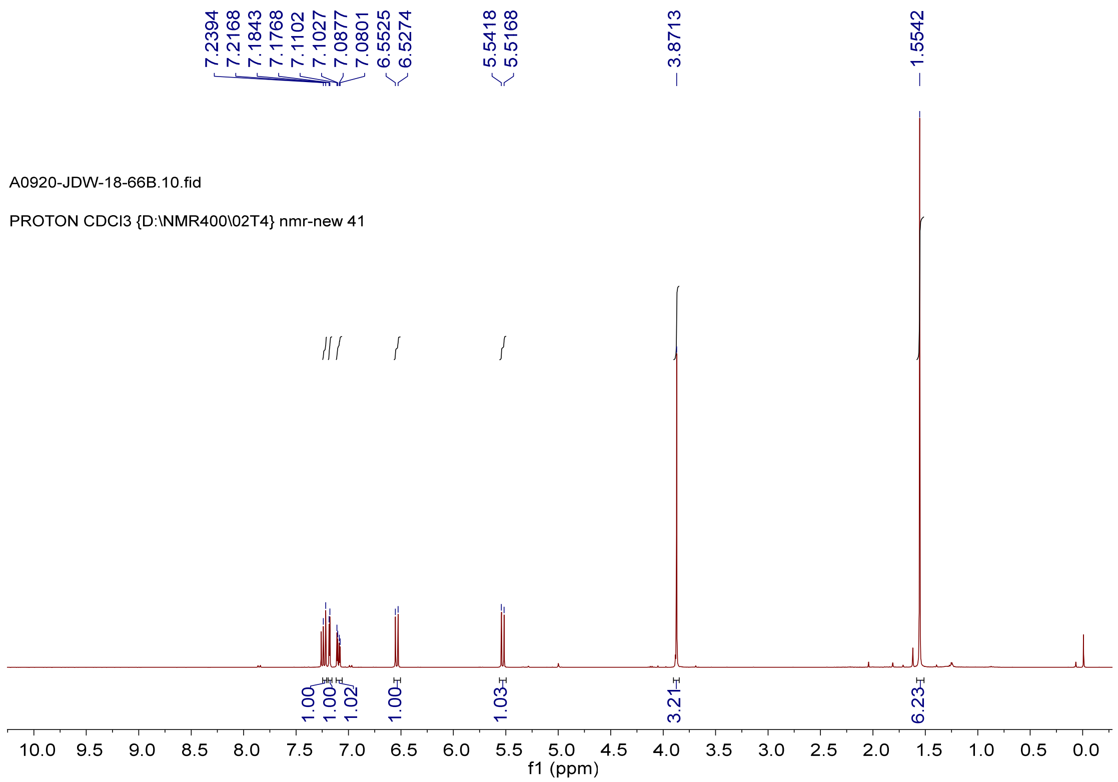

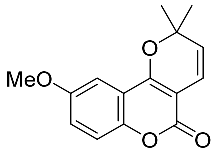

4l

¹H NMR (400 MHz, Chloroform-*d*)

¹³C NMR (100 MHz, Chloroform-*d*)

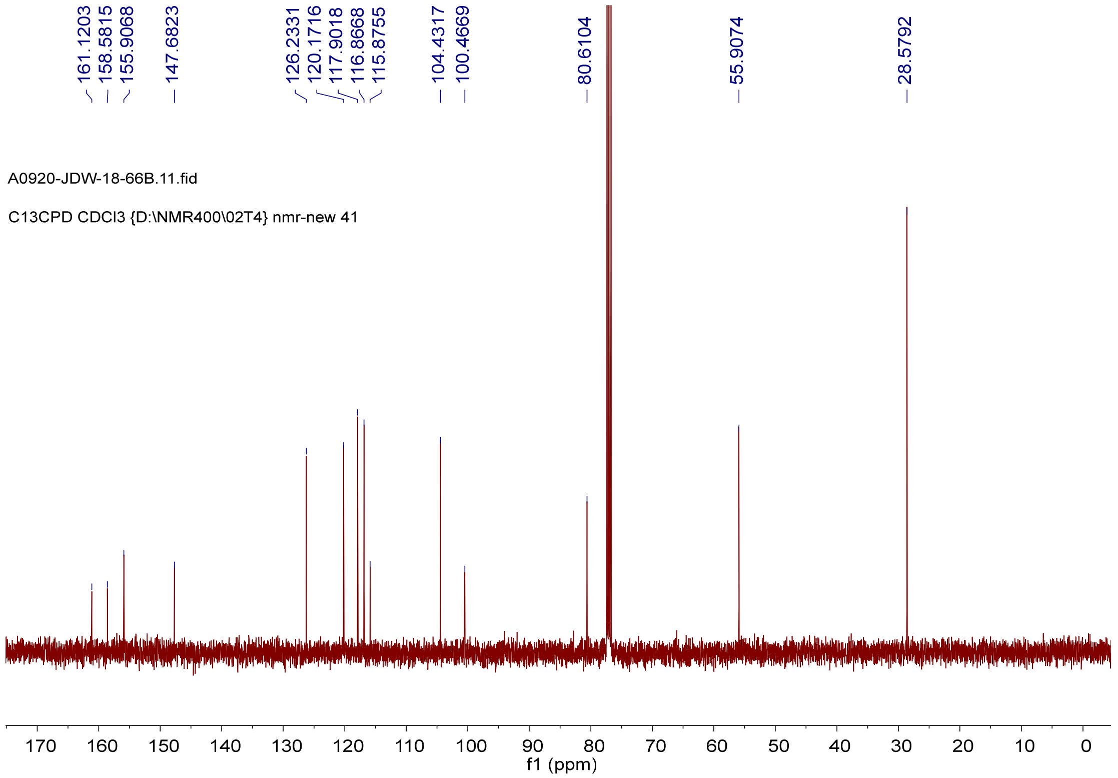

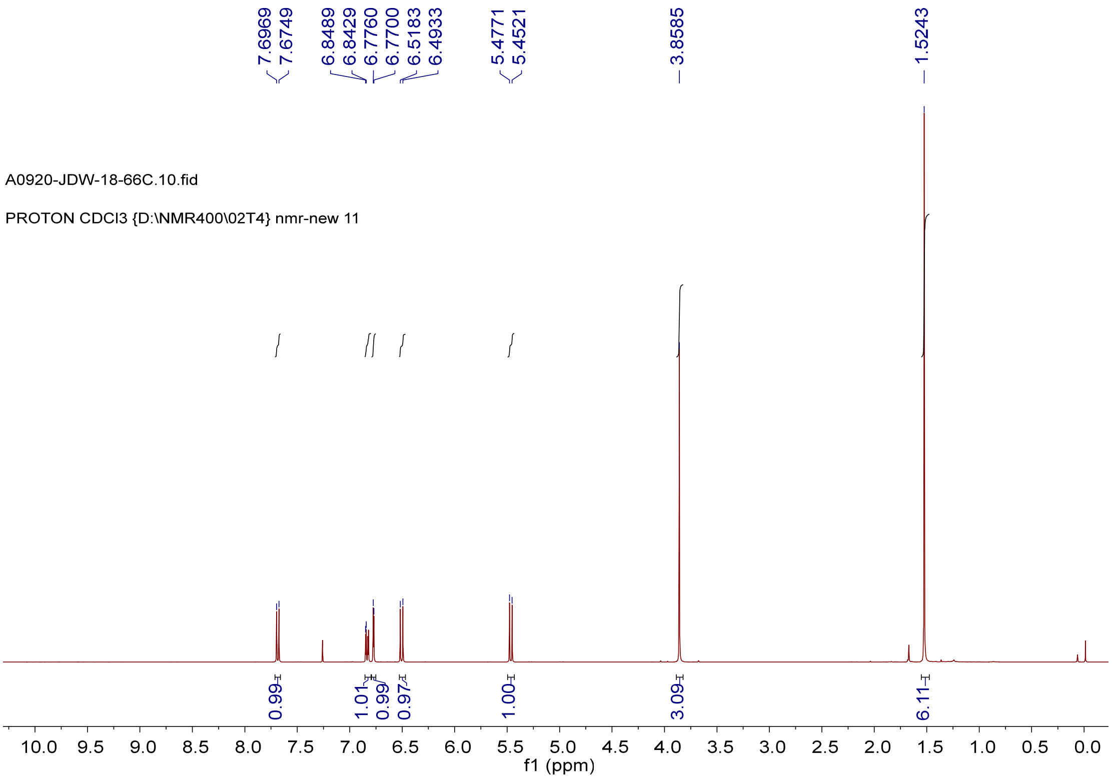

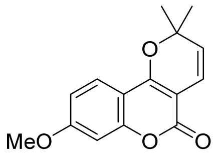

4m

^{1}H NMR (400 MHz, Chloroform-*d*)
^{13}C NMR (100 MHz, Chloroform-*d*)

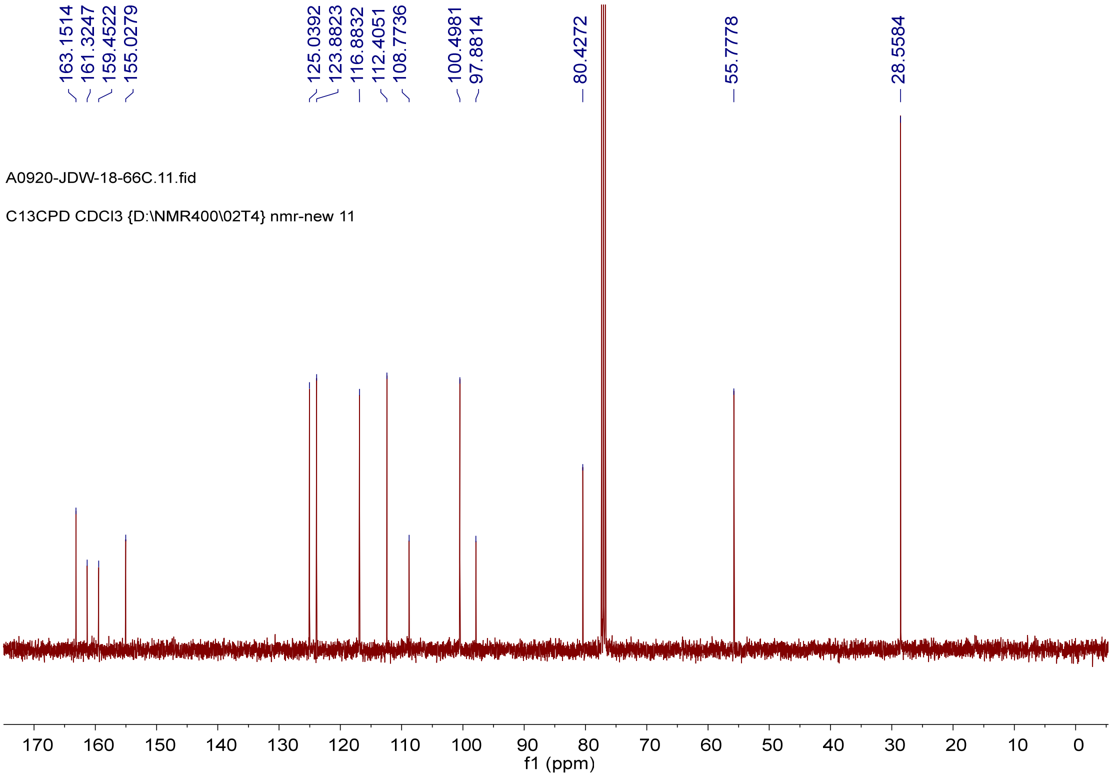

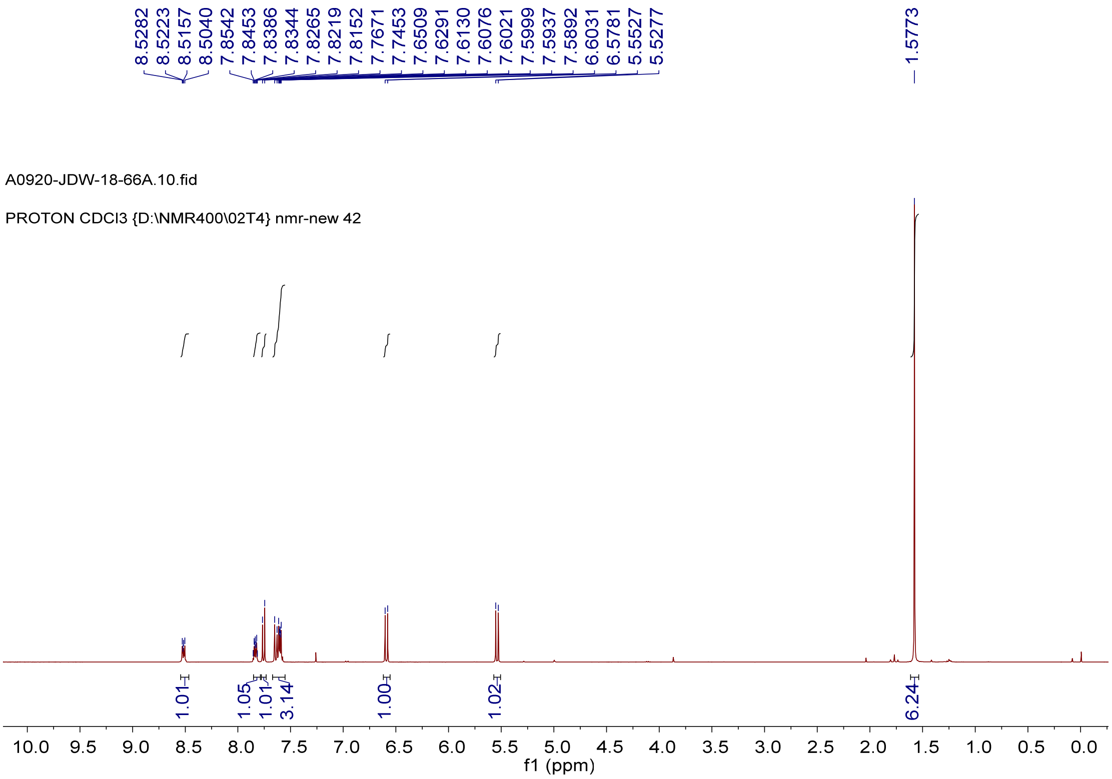

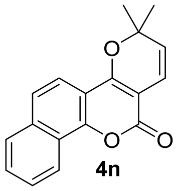

4n

^{1}H NMR (400 MHz, Chloroform-*d*)
^{13}C NMR (100 MHz, Chloroform-*d*)

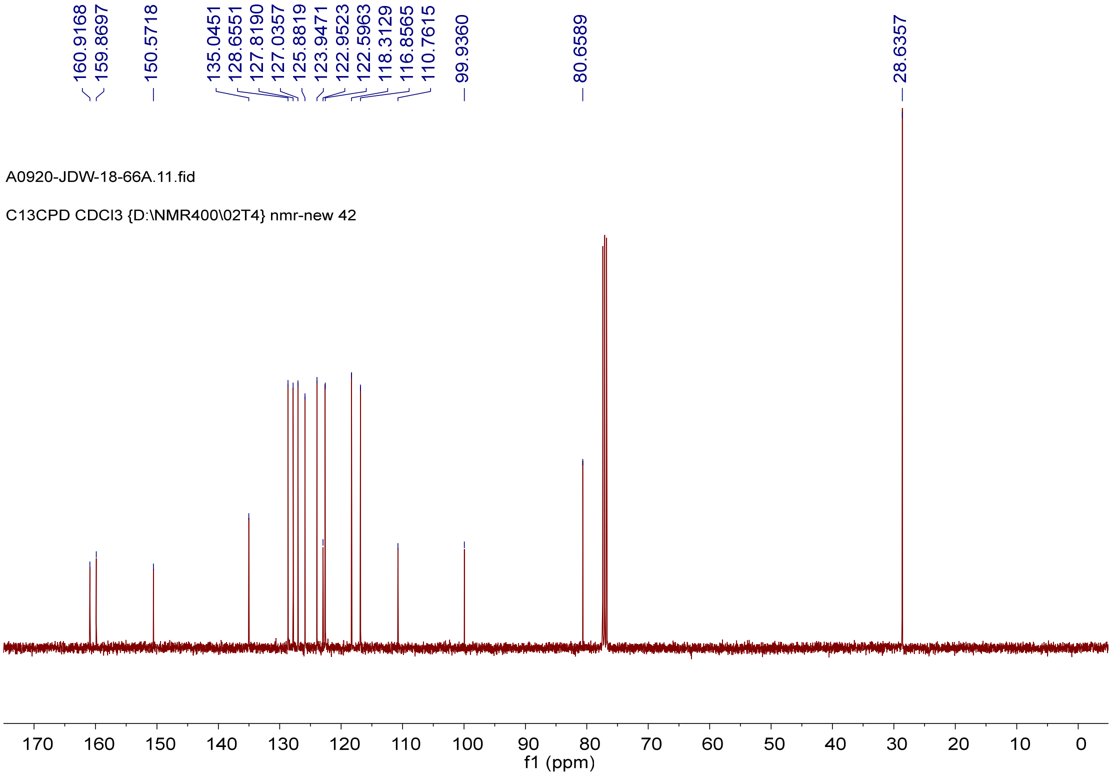

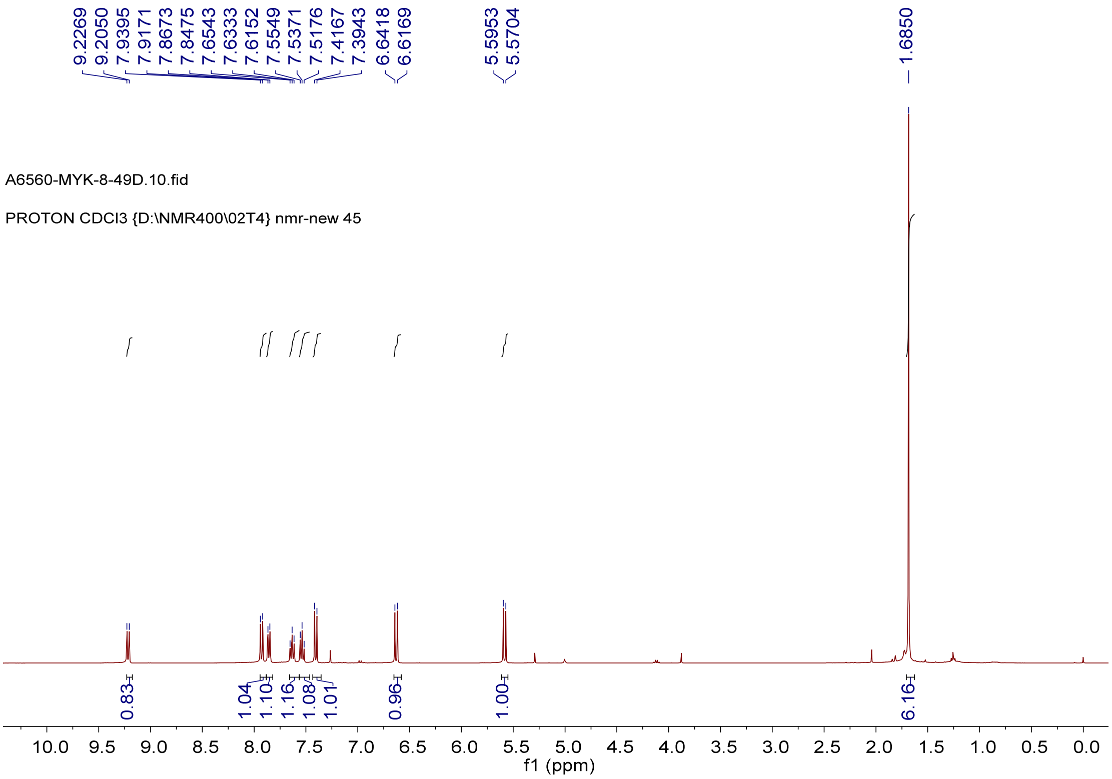

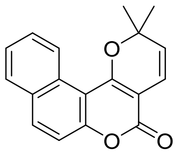

4o

^{1}H NMR (400 MHz, Chloroform-*d*)
^{13}C NMR (100 MHz, Chloroform-*d*)

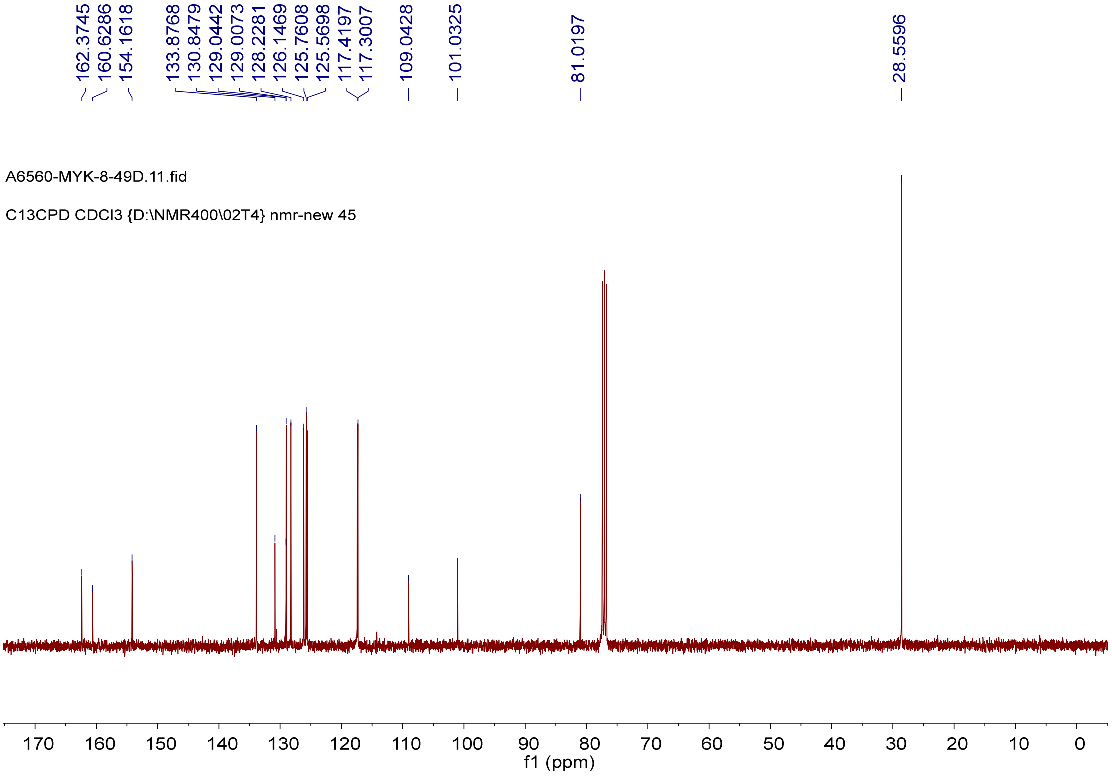

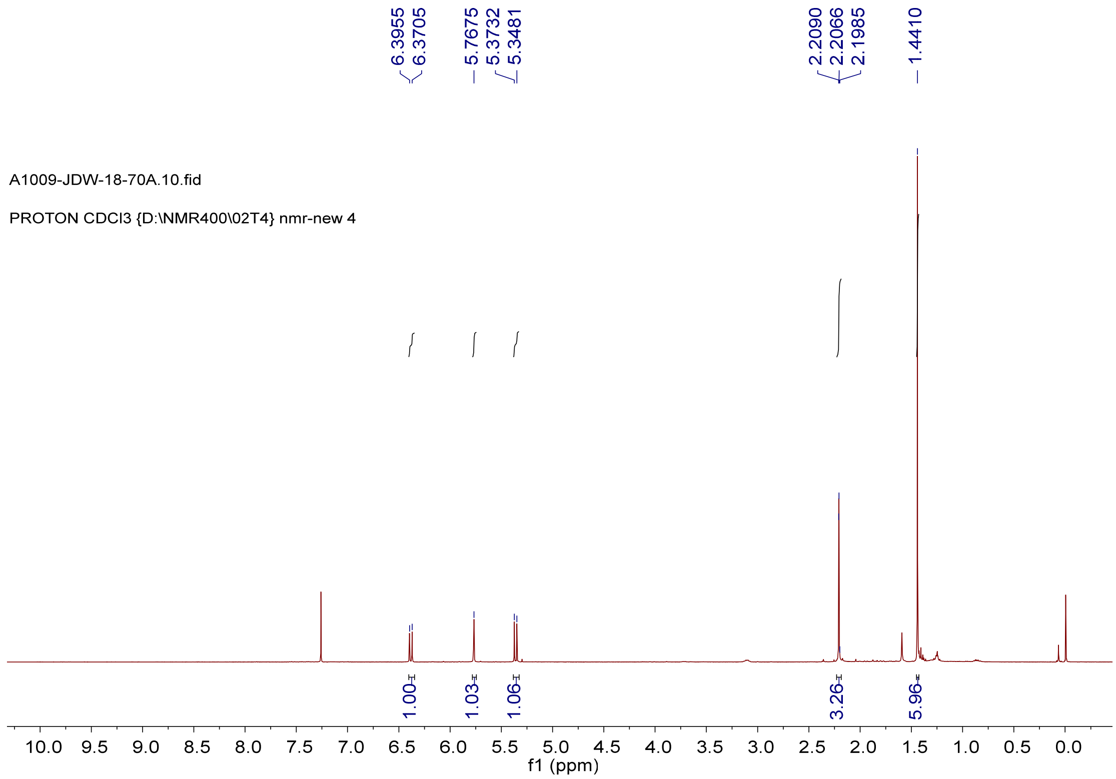

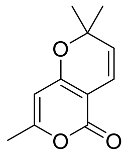

4p

^{1}H NMR (400 MHz, Chloroform-*d*)
^{13}C NMR (100 MHz, Chloroform-*d*)

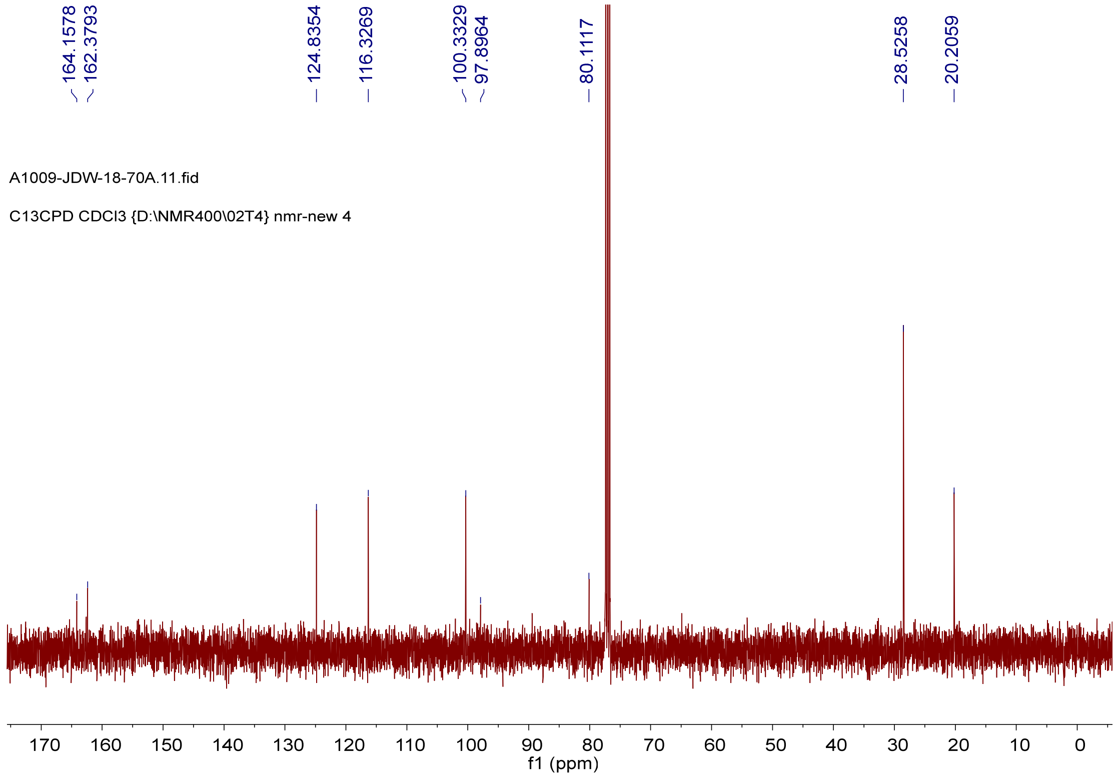

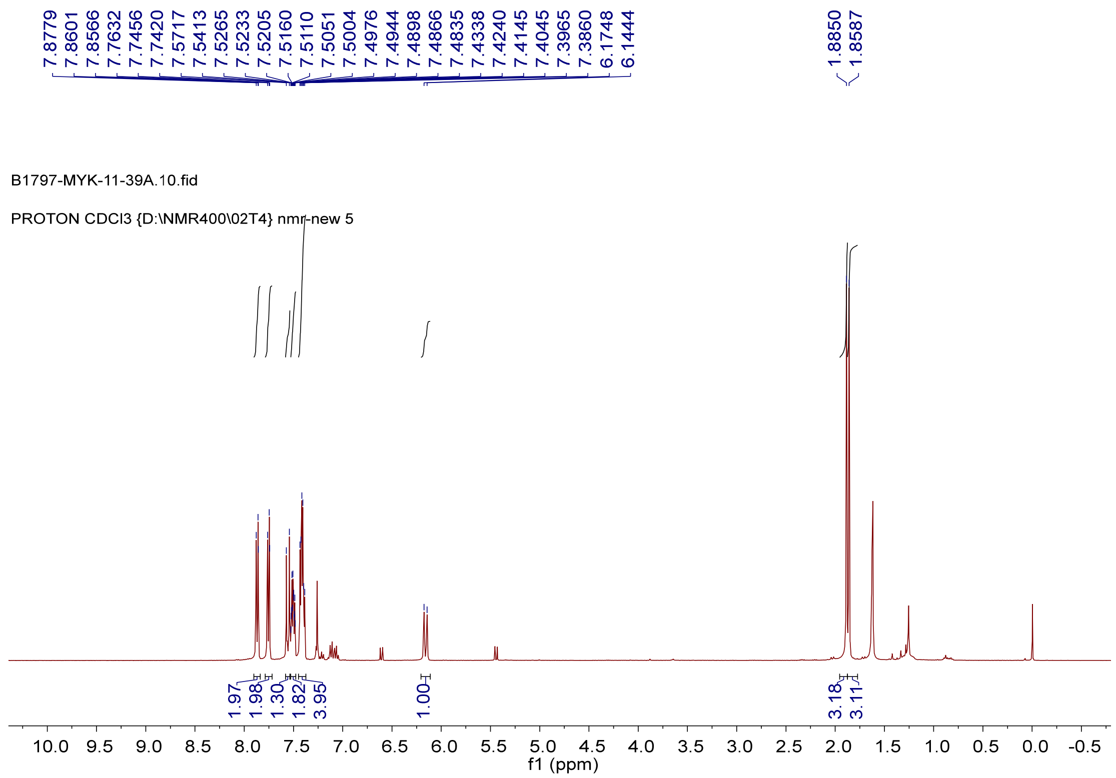

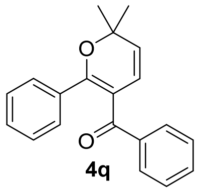

4q

^{1}H NMR (400 MHz, Chloroform-*d*)
^{13}C NMR (100 MHz, Chloroform-*d*)

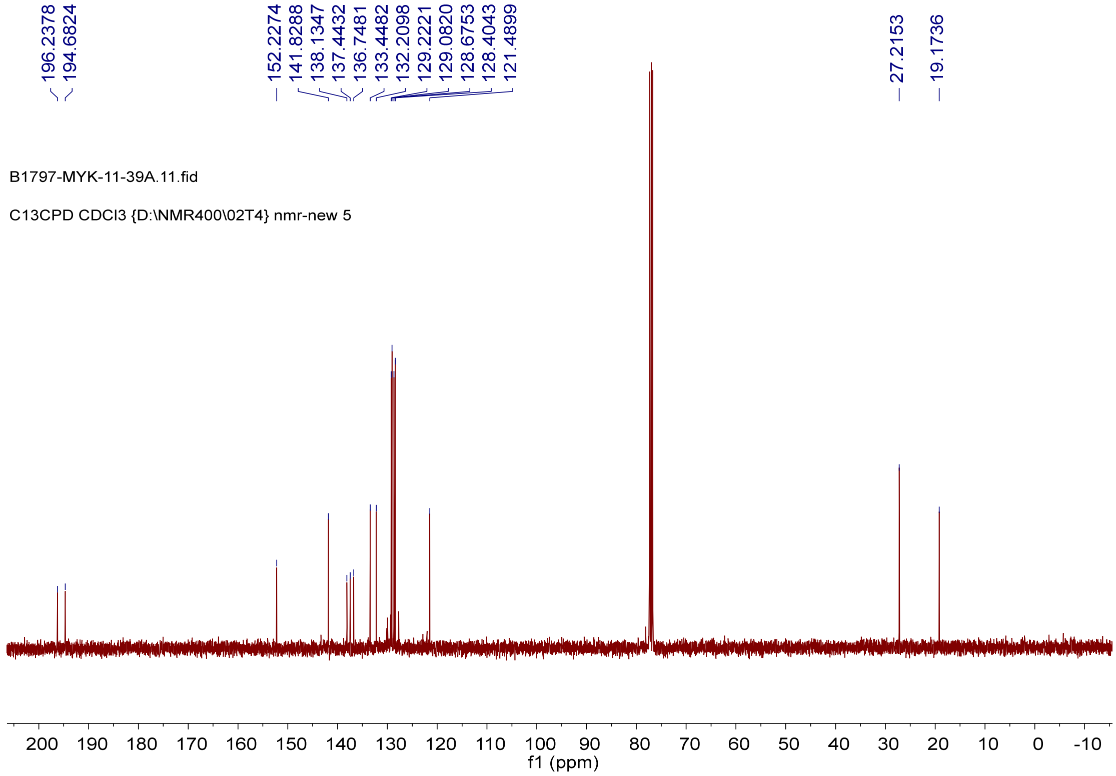

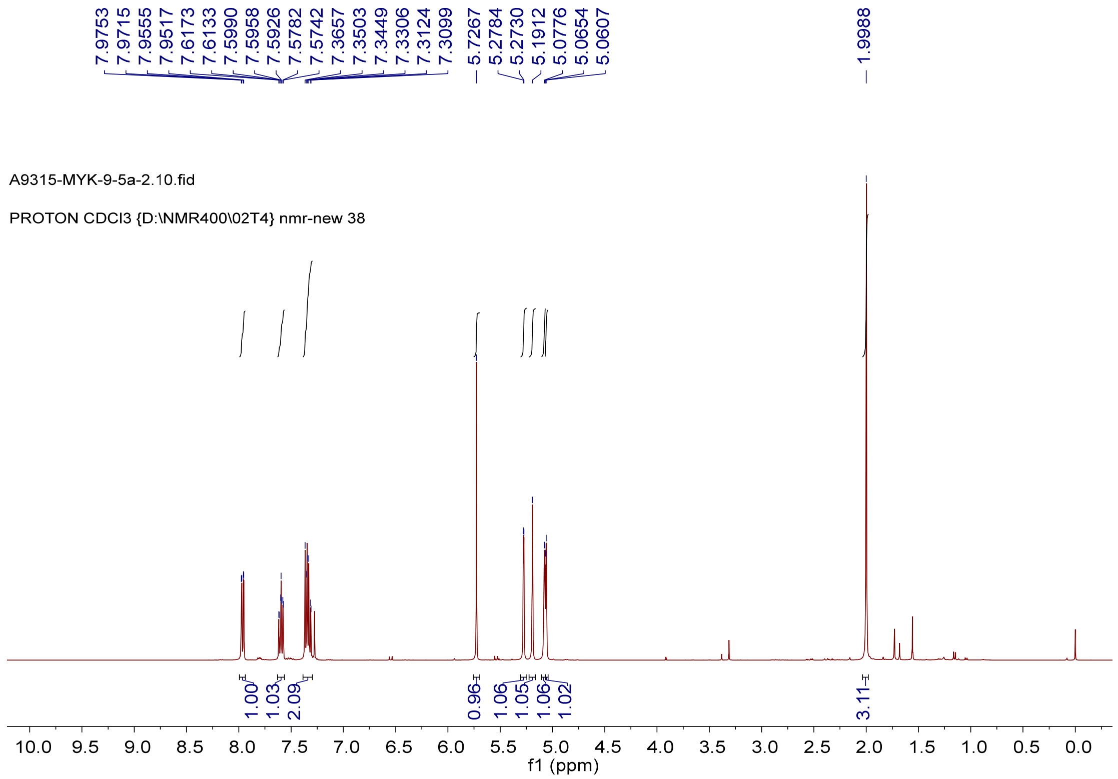

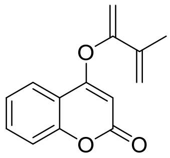

5a

^{1}H NMR (400 MHz, Chloroform-*d*)
^{13}C NMR (100 MHz, Chloroform-*d*)

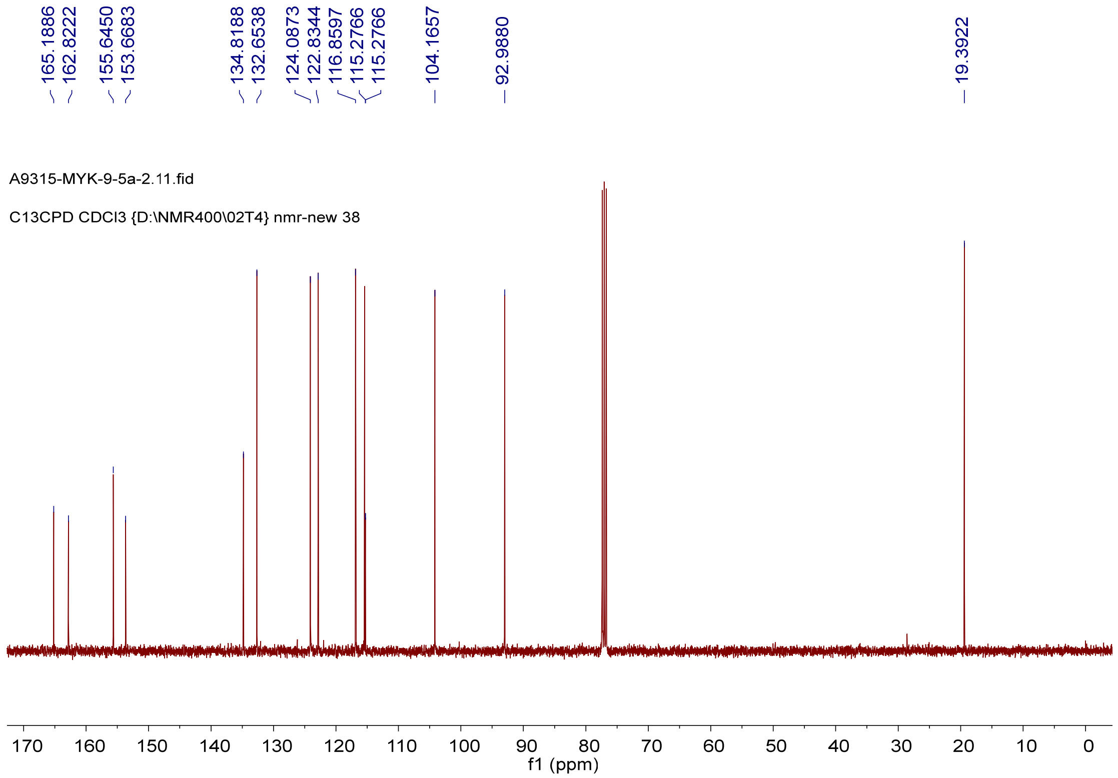

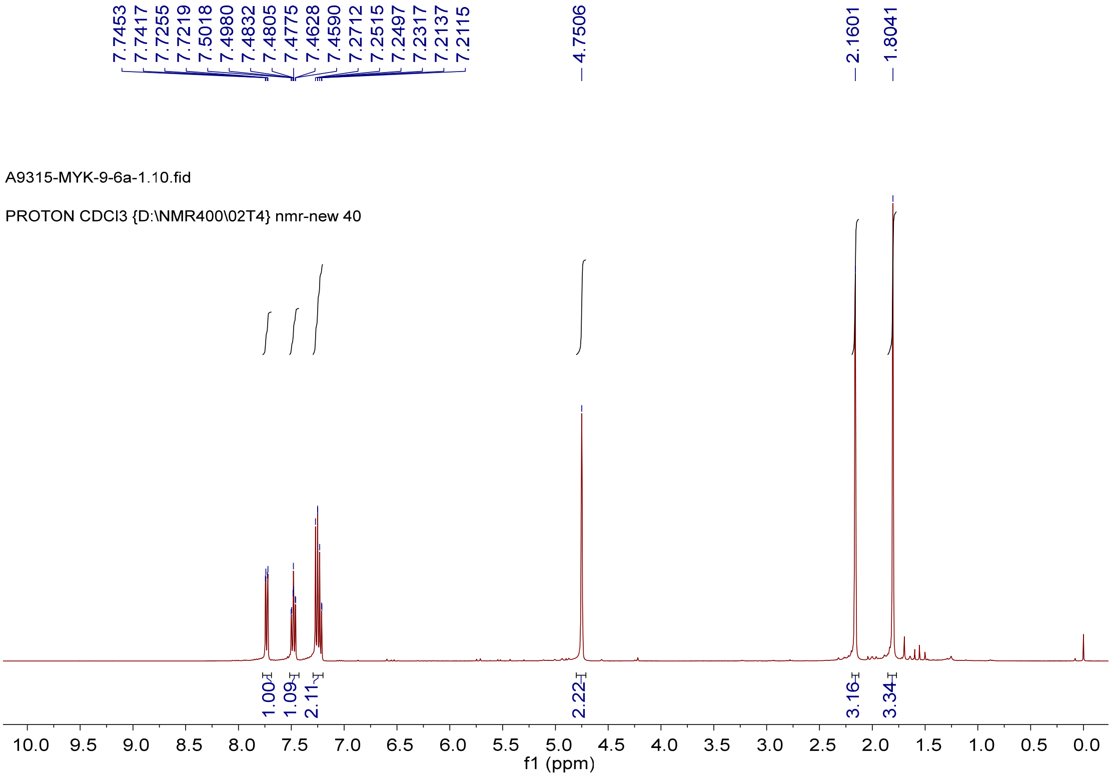

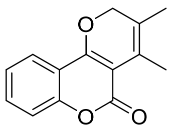

6a

^{1}H NMR (400 MHz, Chloroform-*d*)
^{13}C NMR (100 MHz, Chloroform-*d*)

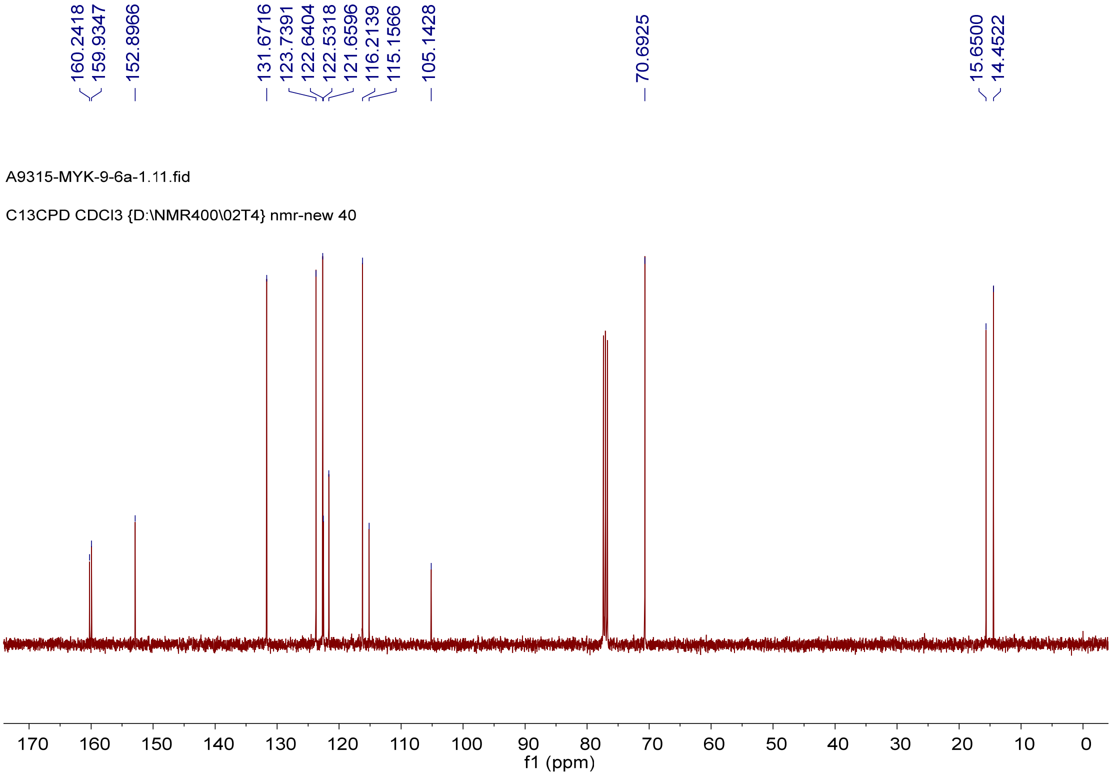

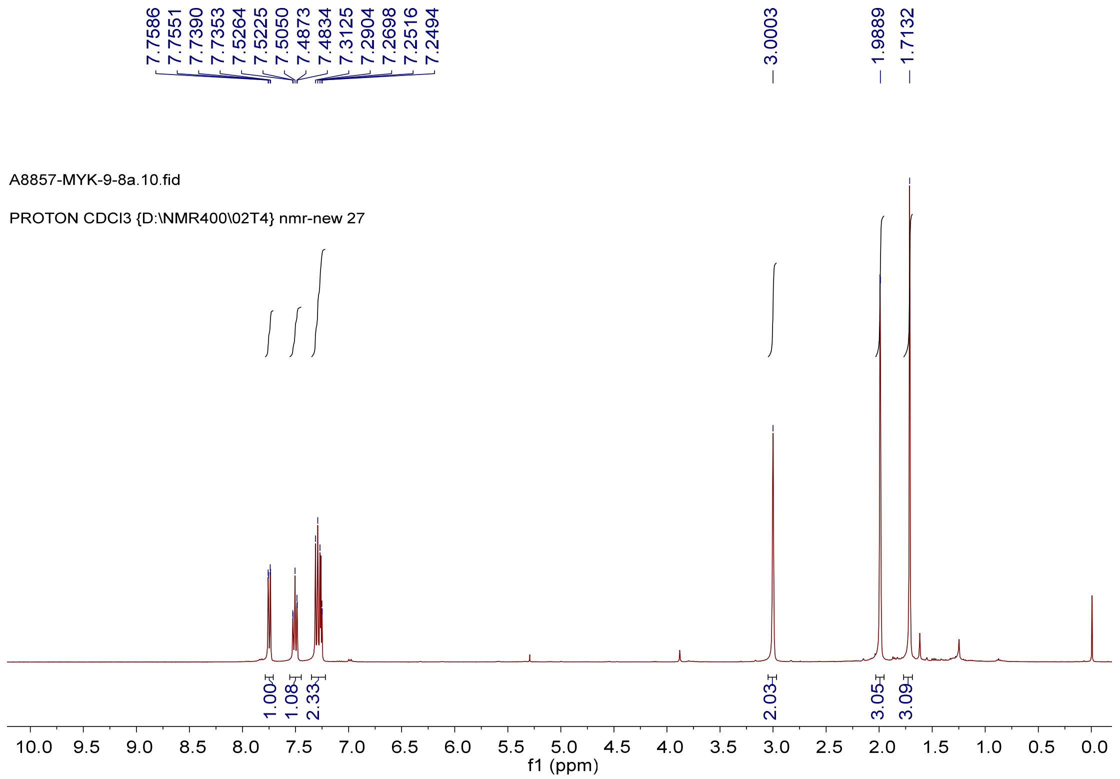

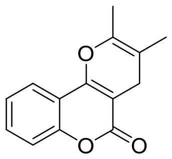

8a

^{1}H NMR (400 MHz, Chloroform-*d*)
^{13}C NMR (100 MHz, Chloroform-*d*)

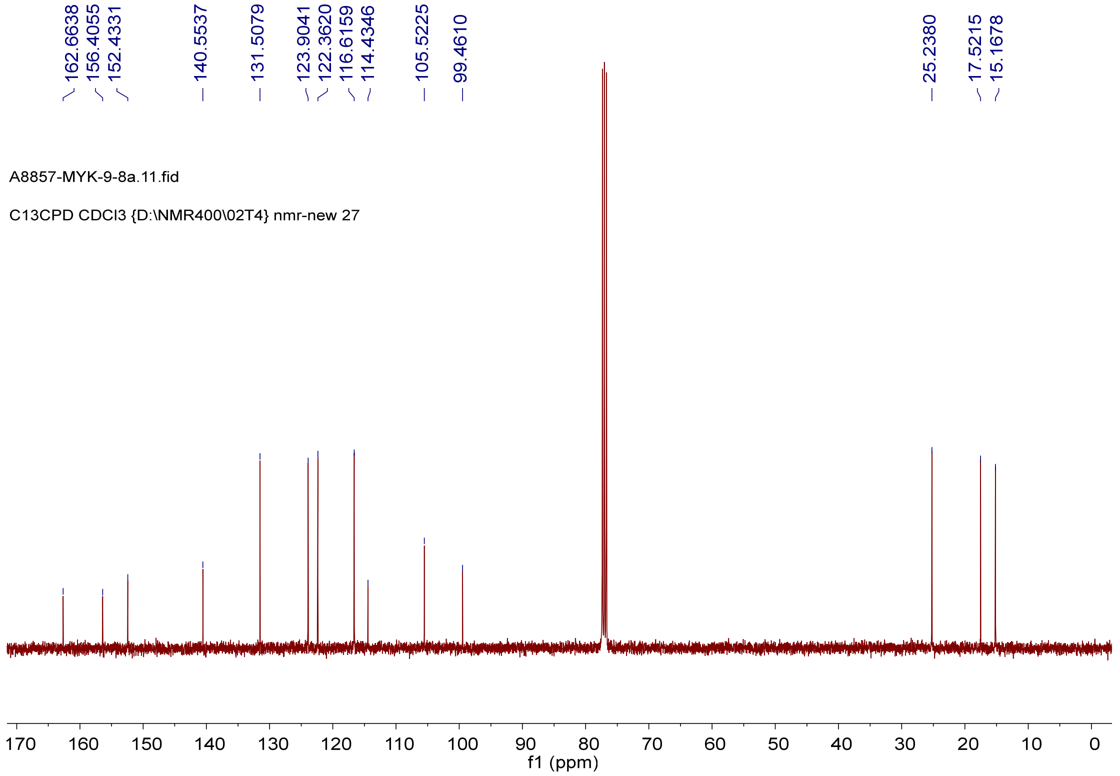

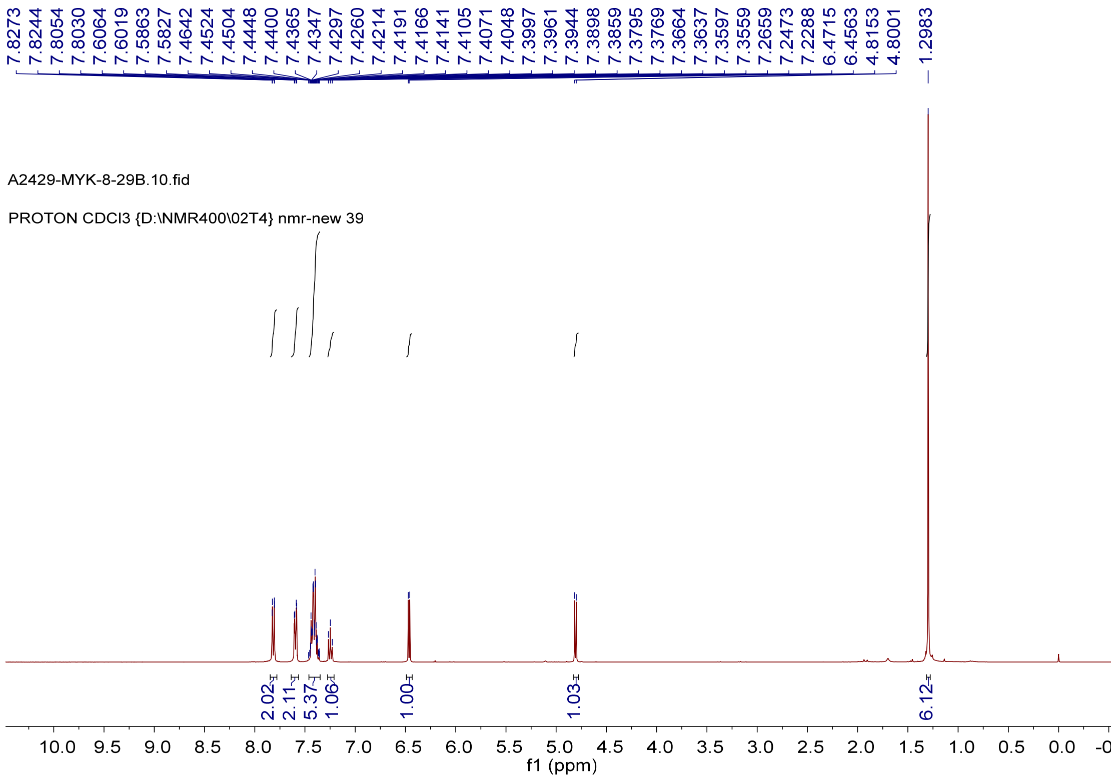

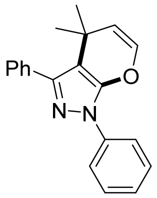

10a

^{1}H NMR (400 MHz, Chloroform-*d*)

^{13}C NMR (100 MHz, Chloroform-*d*)

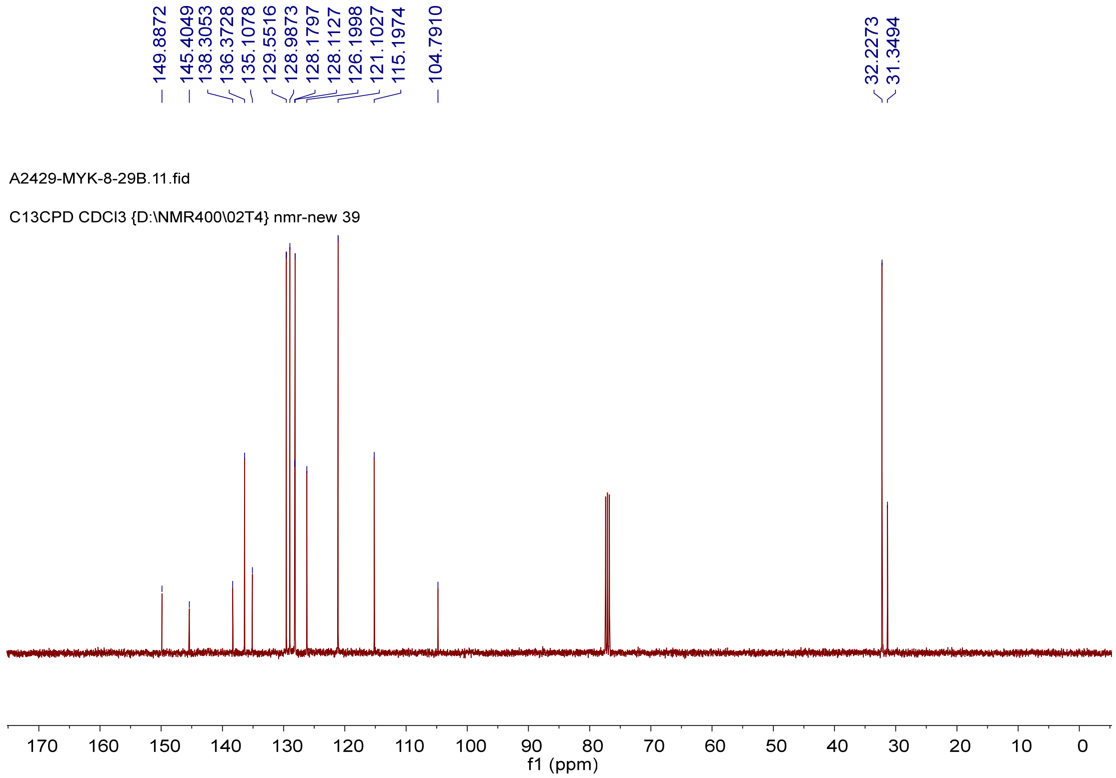

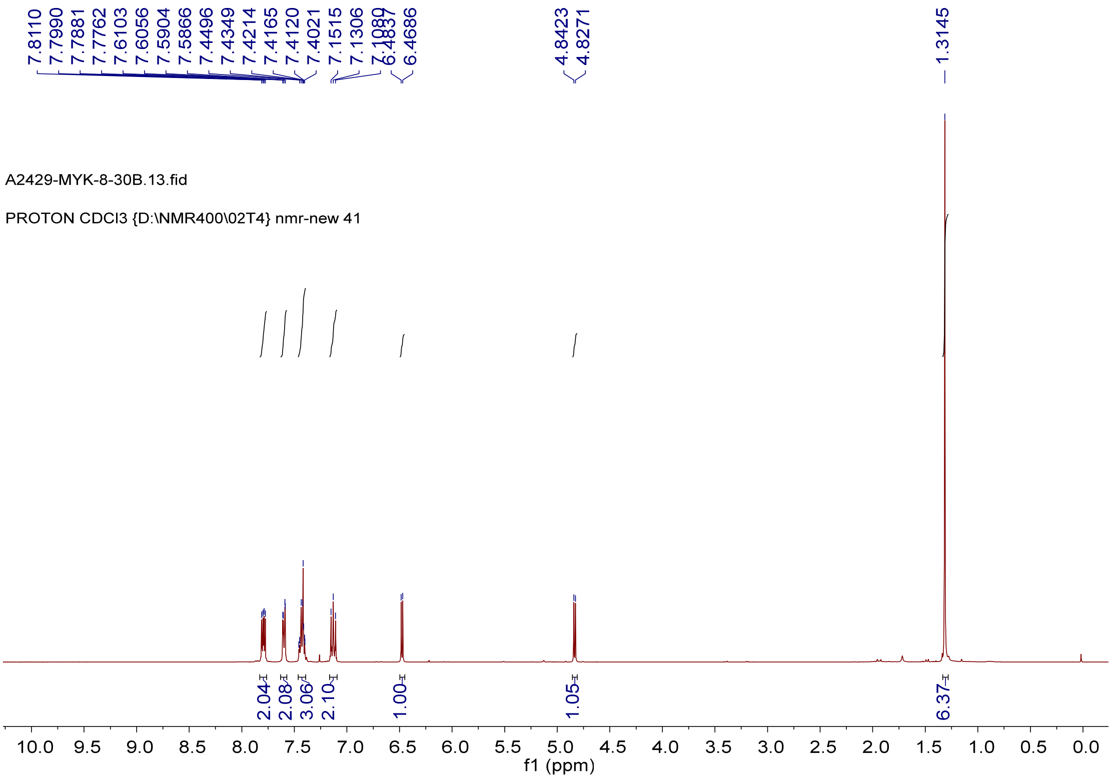

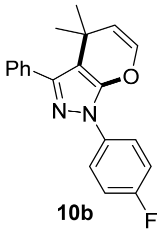

10b

^{1}H NMR (400 MHz, Chloroform-*d*)
^{13}C NMR (100 MHz, Chloroform-*d*)

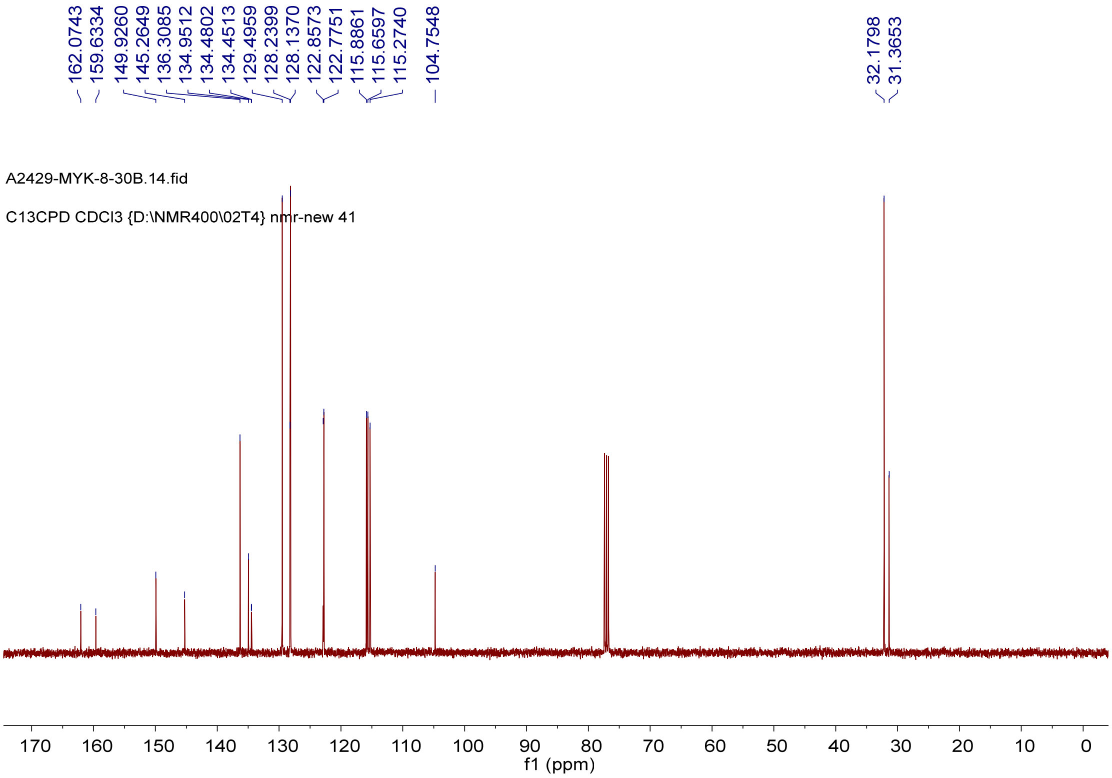

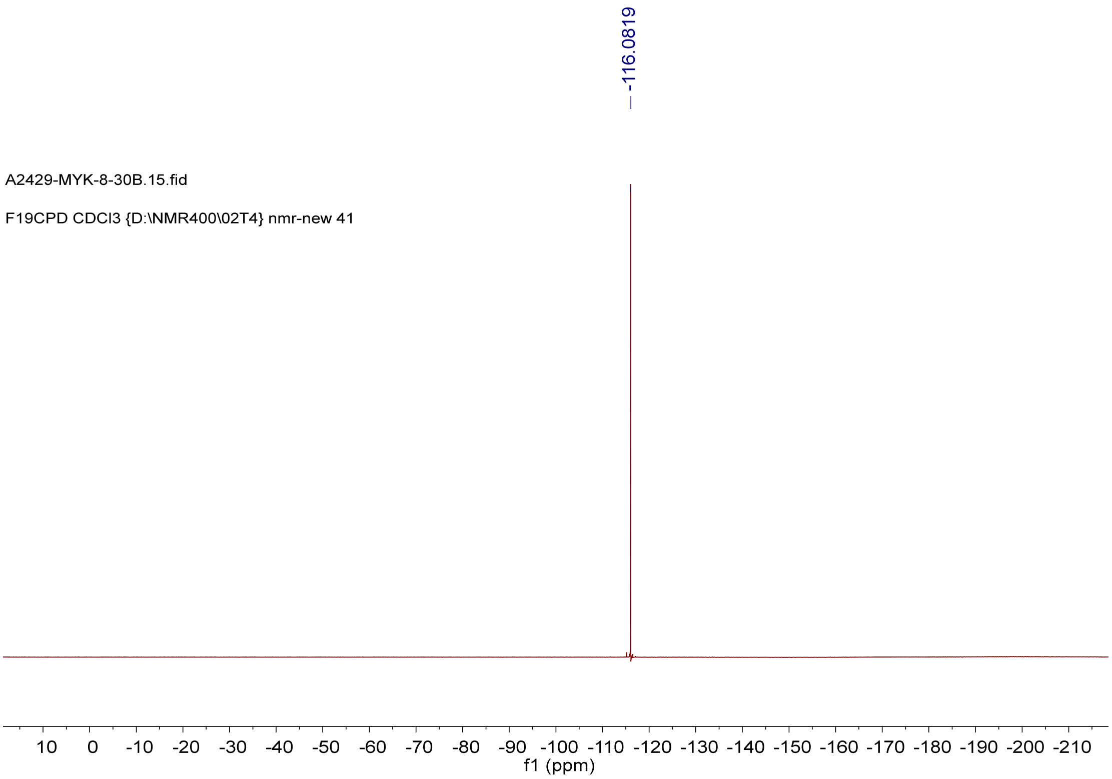

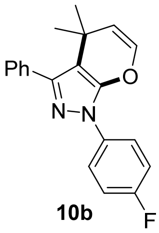

10b

^{19}F NMR (375 MHz, Chloroform-*d*)

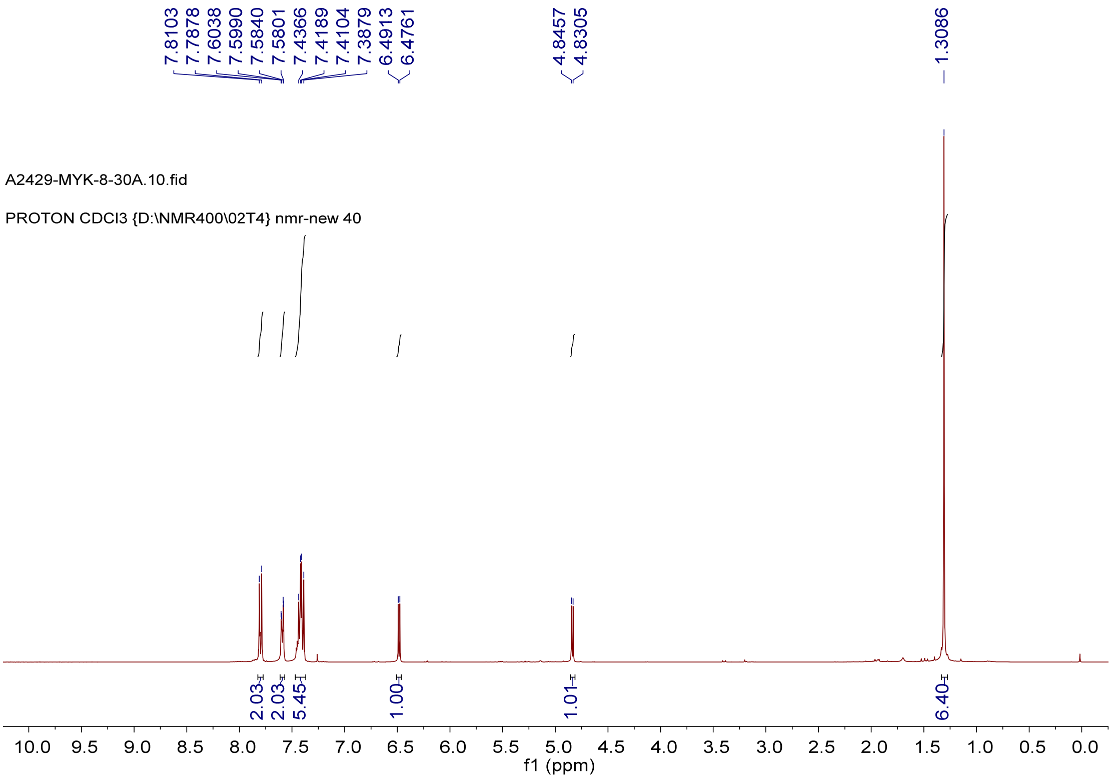

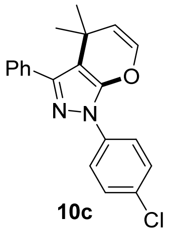

10c

^{1}H NMR (400 MHz, Chloroform-*d*)

^{13}C NMR (100 MHz, Chloroform-*d*)

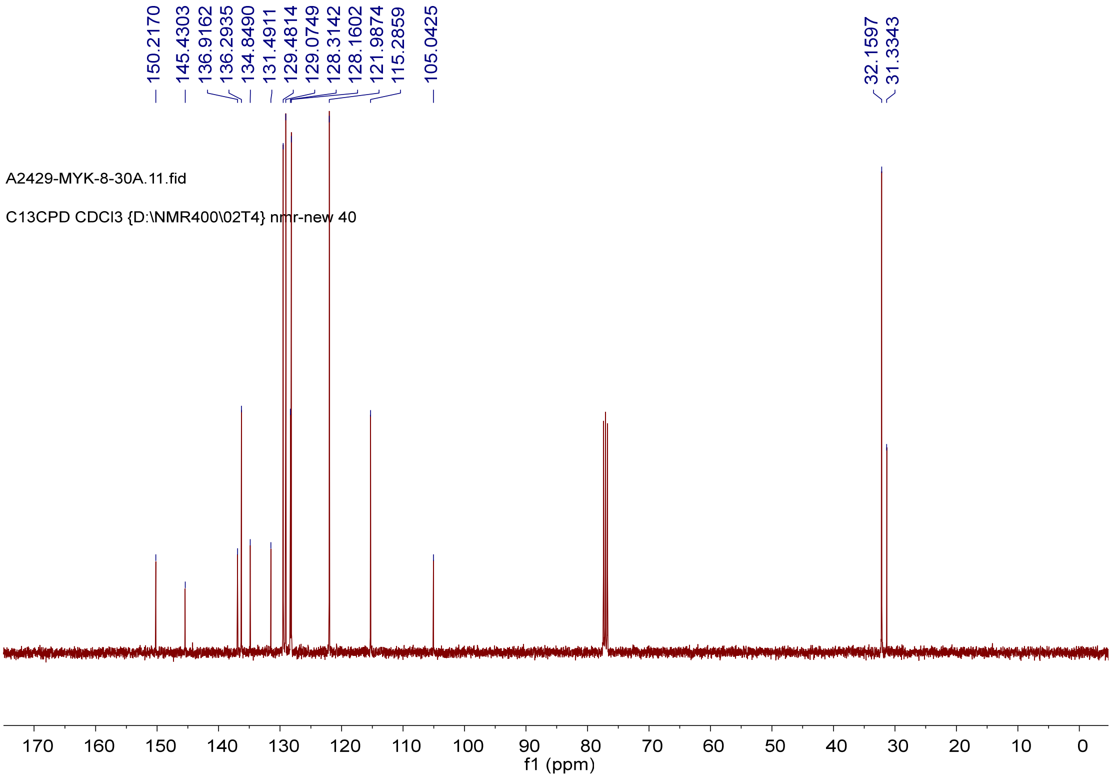

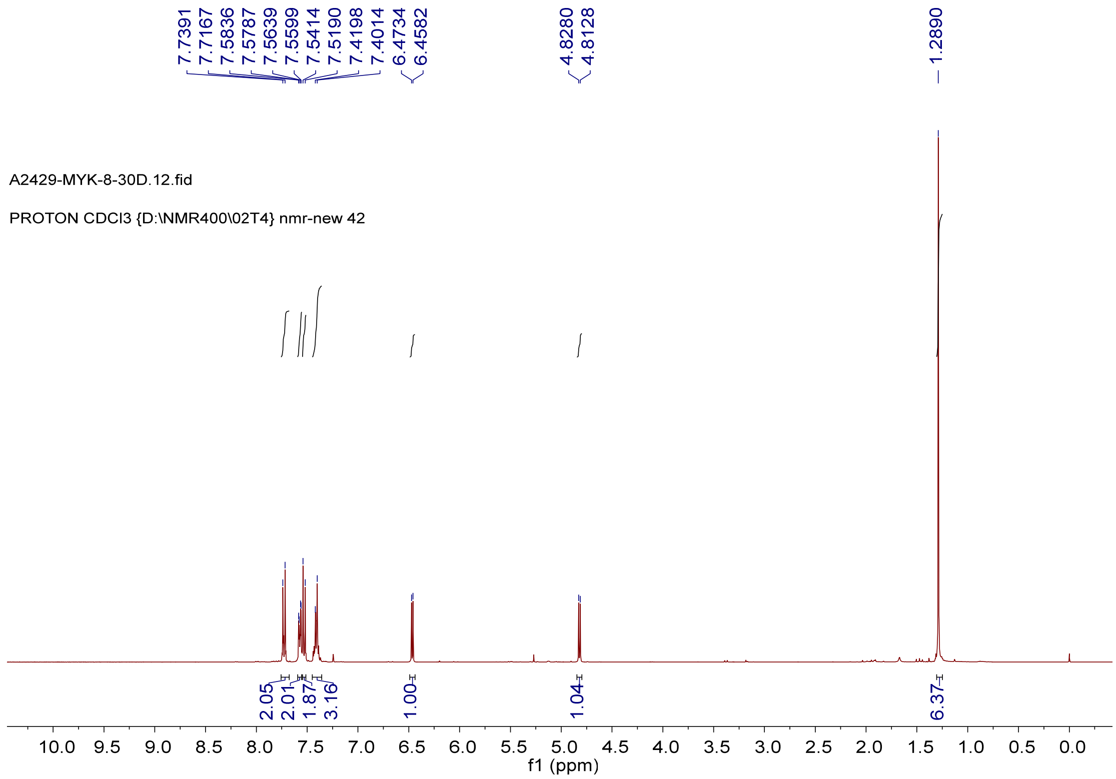

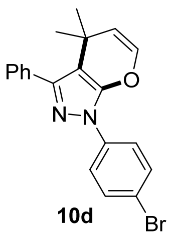

10d

^{1}H NMR (400 MHz, Chloroform-*d*)

^{13}C NMR (100 MHz, Chloroform-*d*)

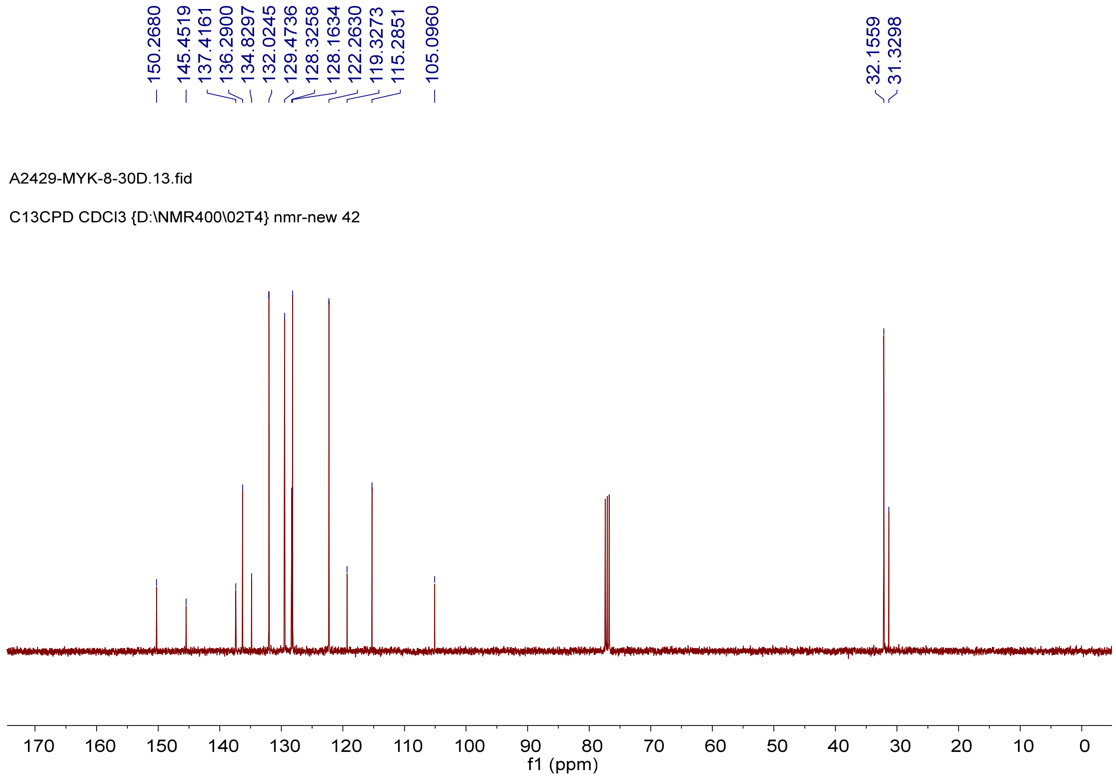

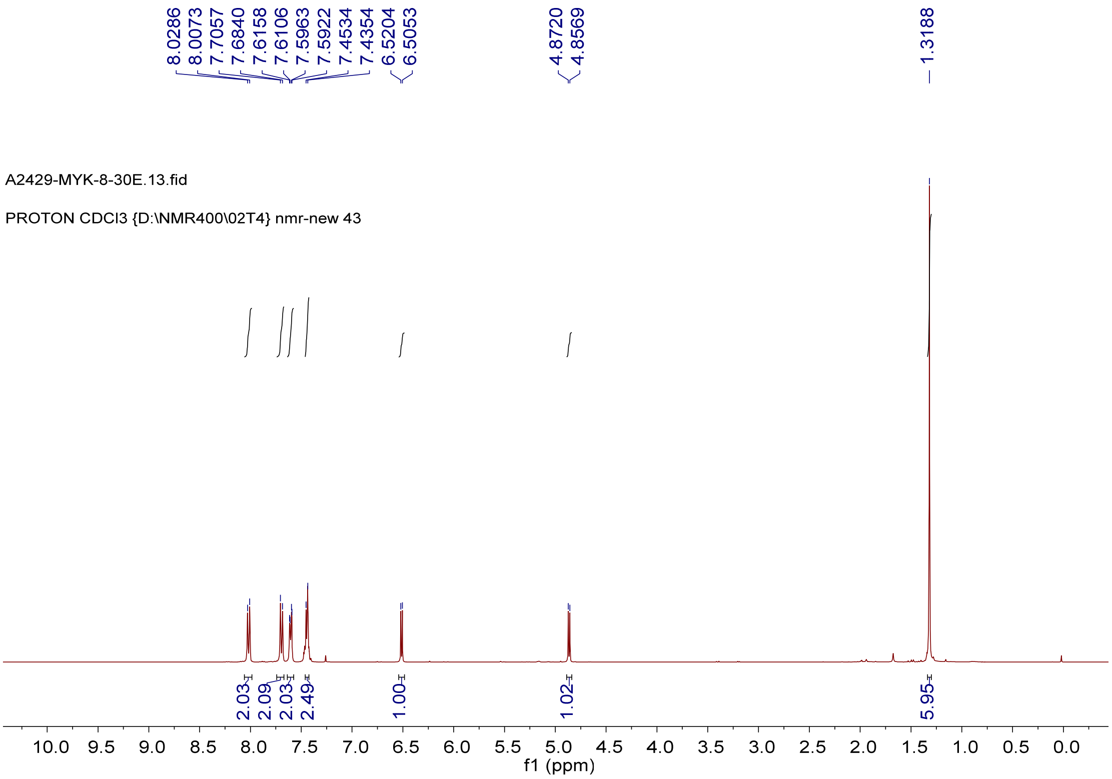

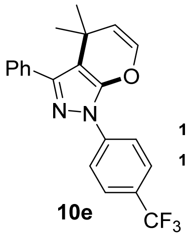

10e

^{1}H NMR (400 MHz, Chloroform-*d*)
^{13}C NMR (100 MHz, Chloroform-*d*)

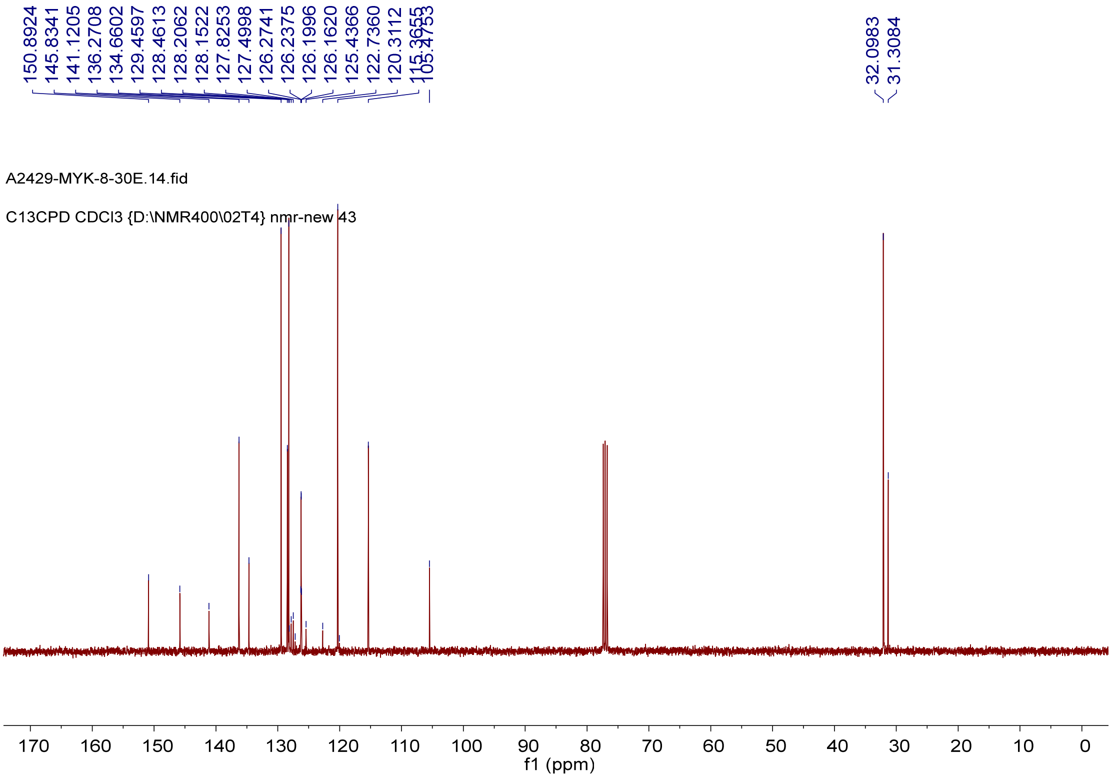

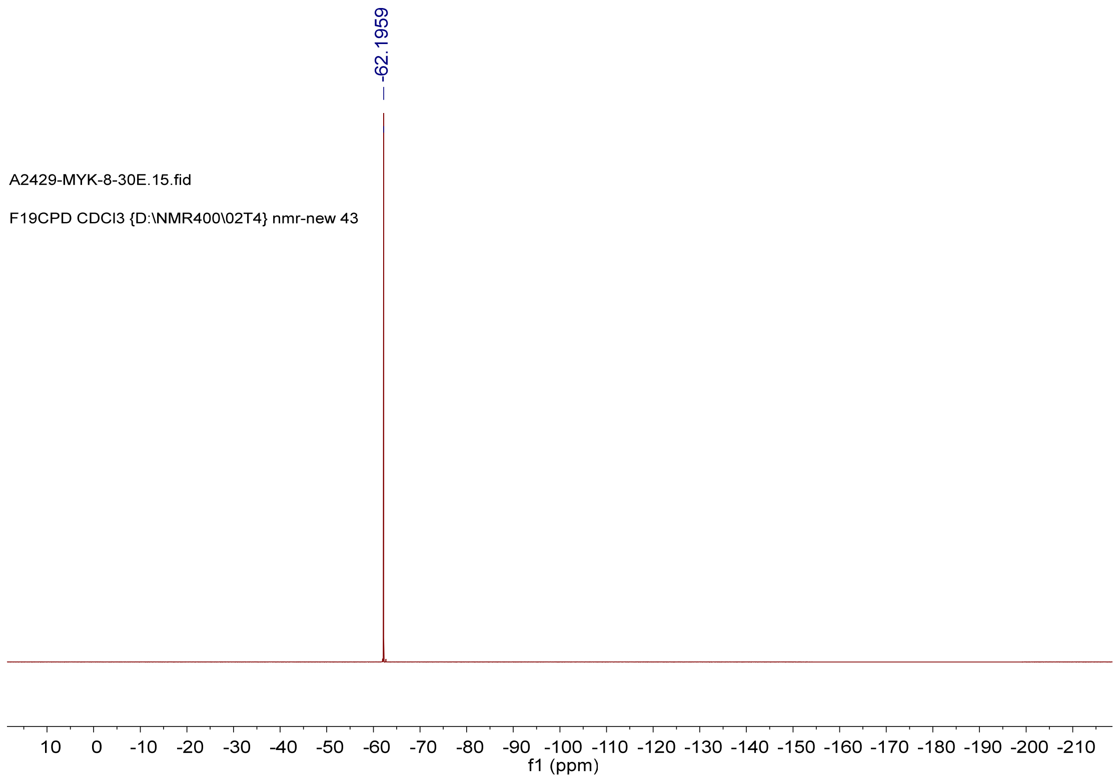

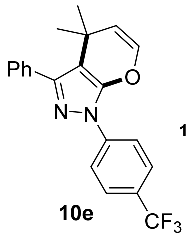

10e

^{19}F NMR (375 MHz, Chloroform-*d*)

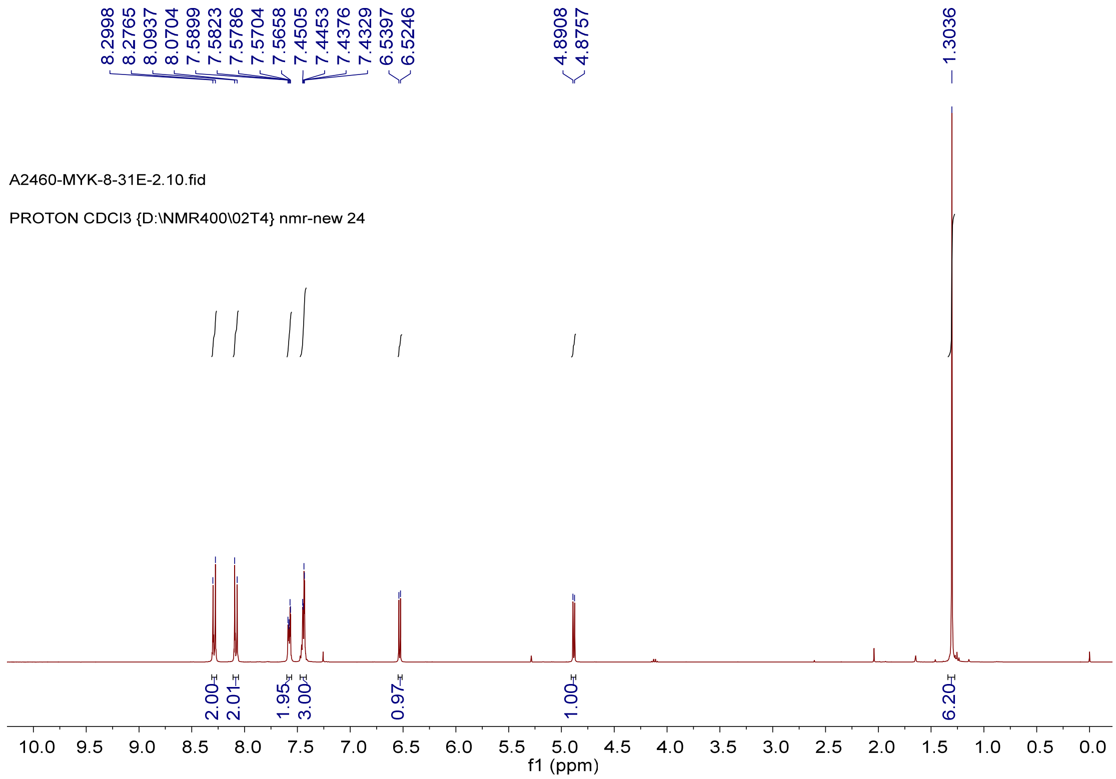

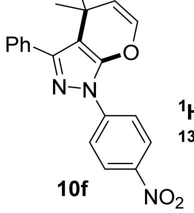

10f

^{1}H NMR (400 MHz, Chloroform-*d*)
^{13}C NMR (100 MHz, Chloroform-*d*)

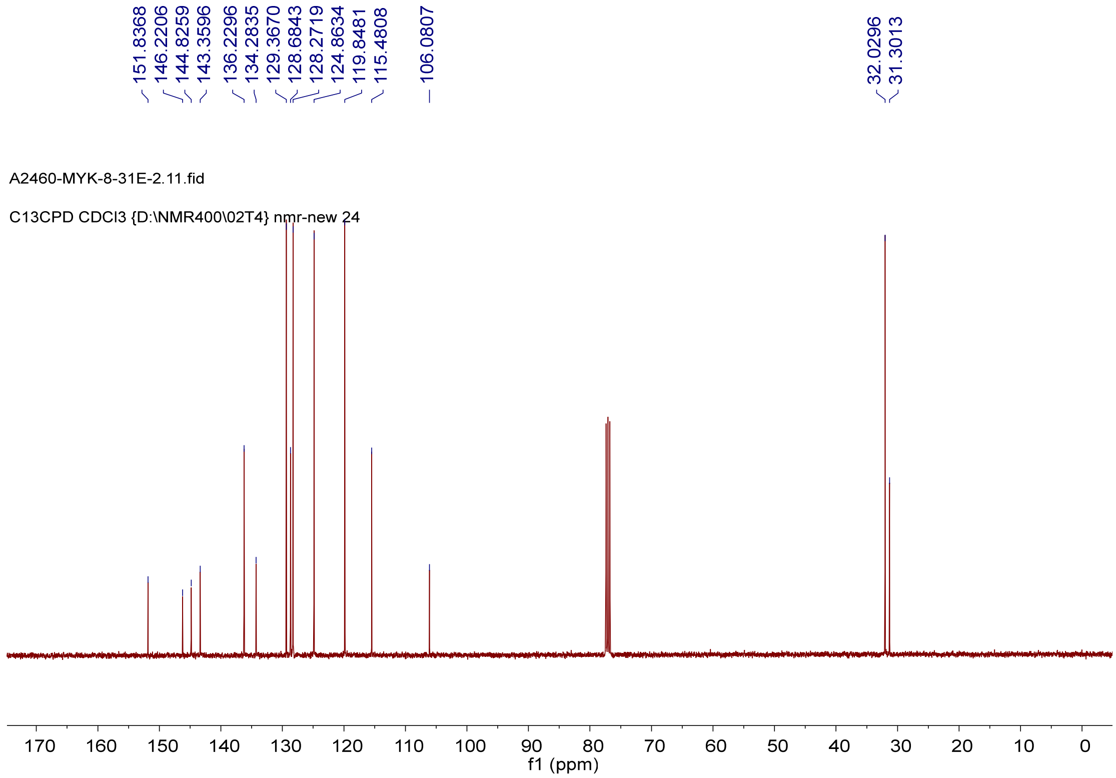

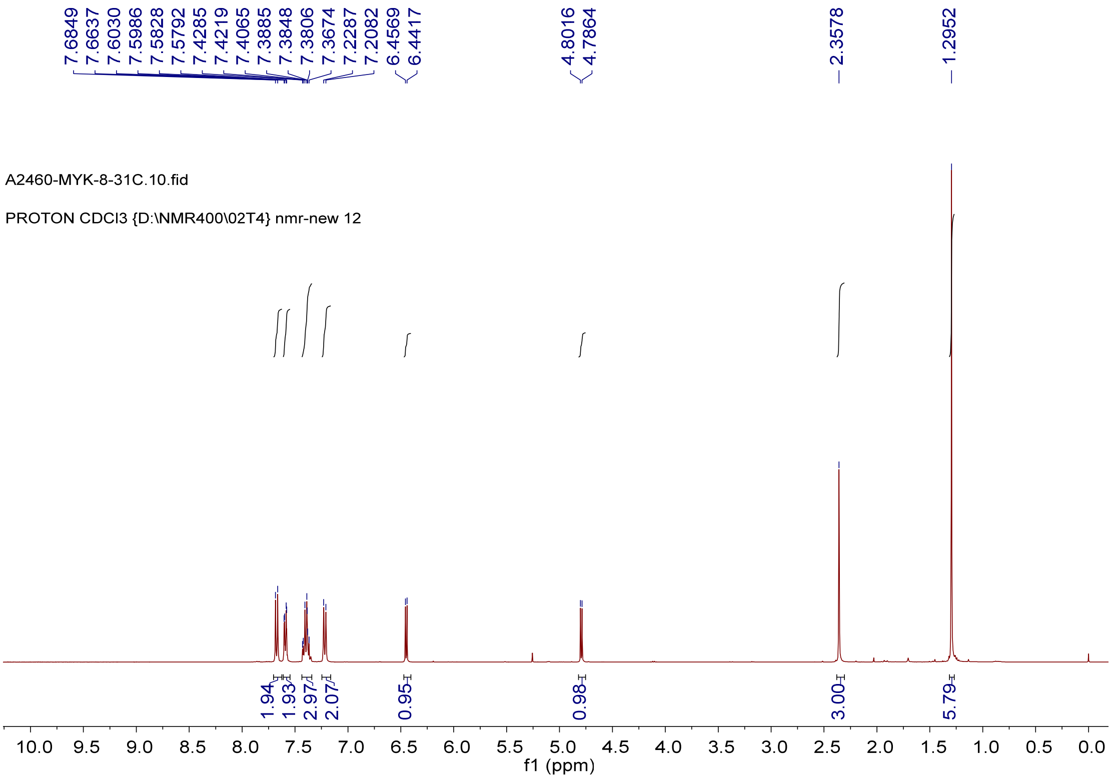

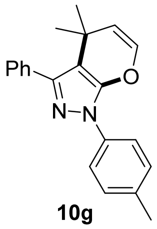

10g

^{1}H NMR (400 MHz, Chloroform-*d*)
^{13}C NMR (100 MHz, Chloroform-*d*)

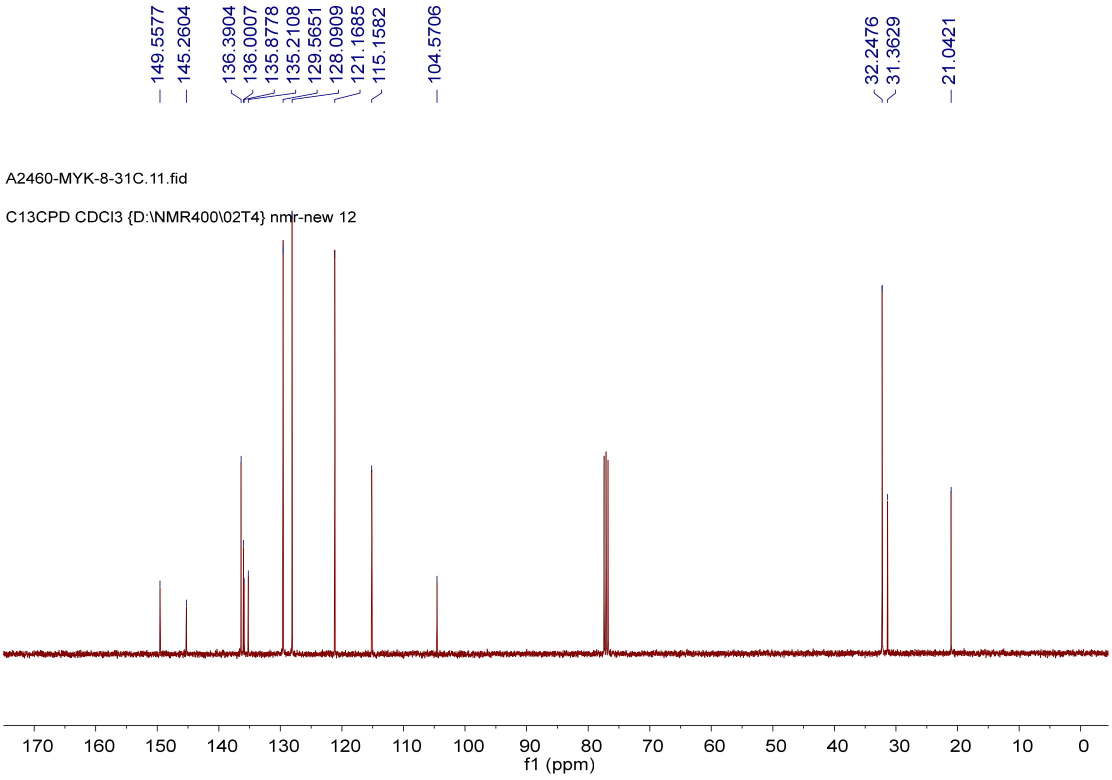

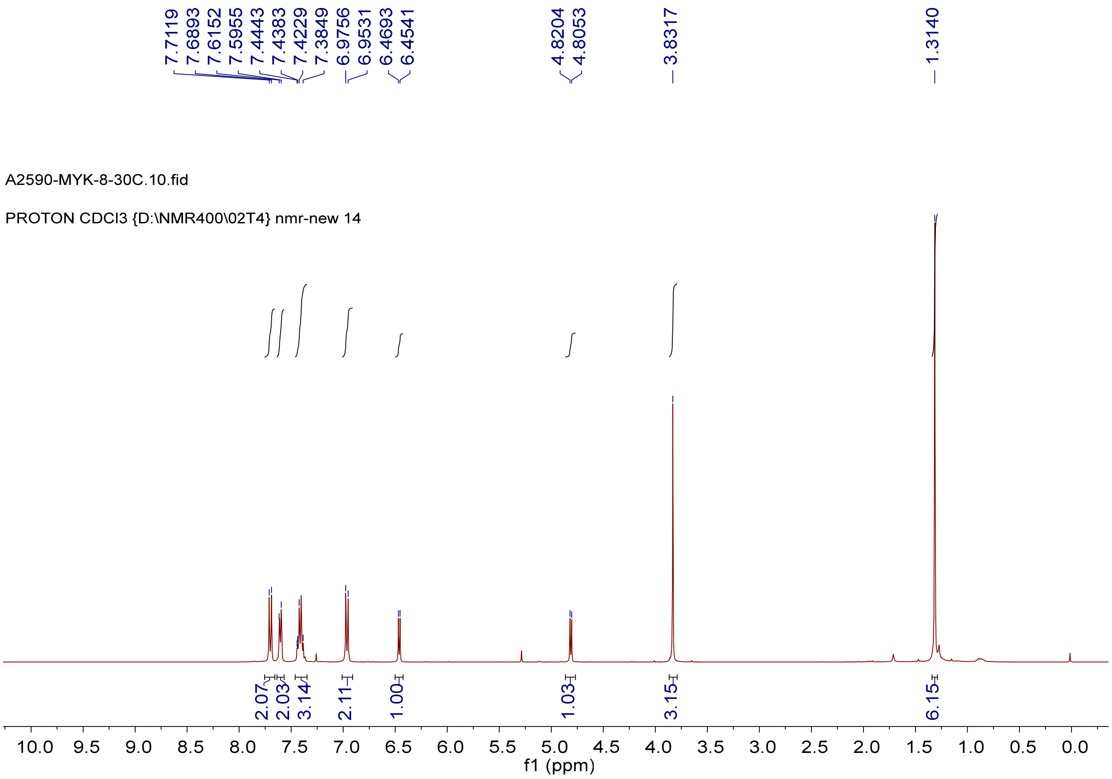

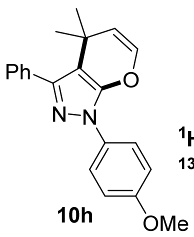

10h

^{1}H NMR (400 MHz, Chloroform-*d*)
^{13}C NMR (100 MHz, Chloroform-*d*)

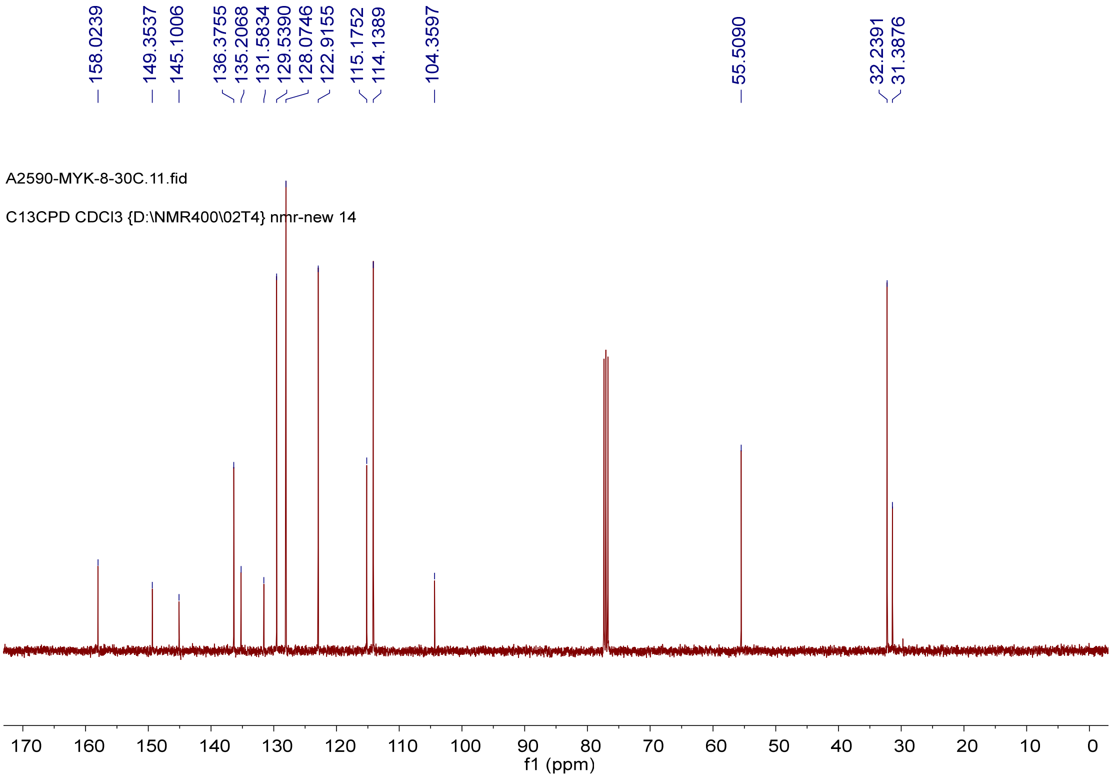

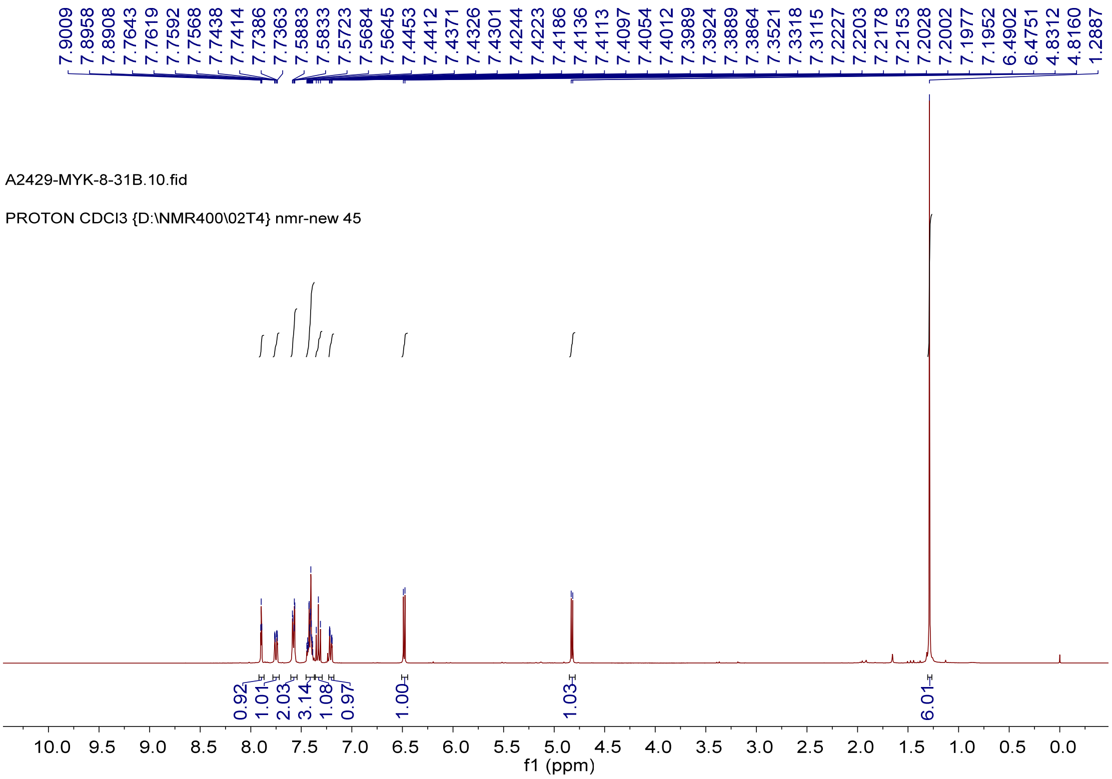

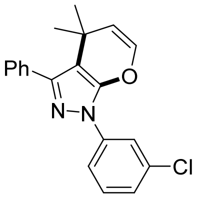

10i

^{1}H NMR (400 MHz, Chloroform-*d*)
^{13}C NMR (100 MHz, Chloroform-*d*)

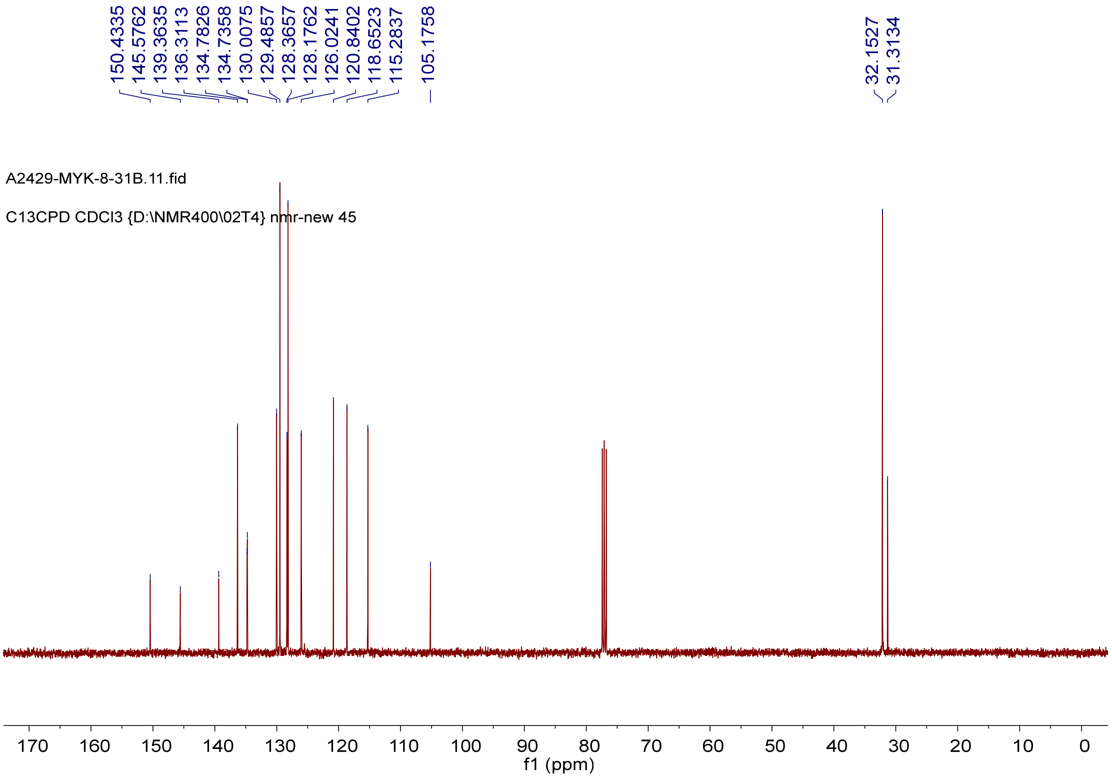

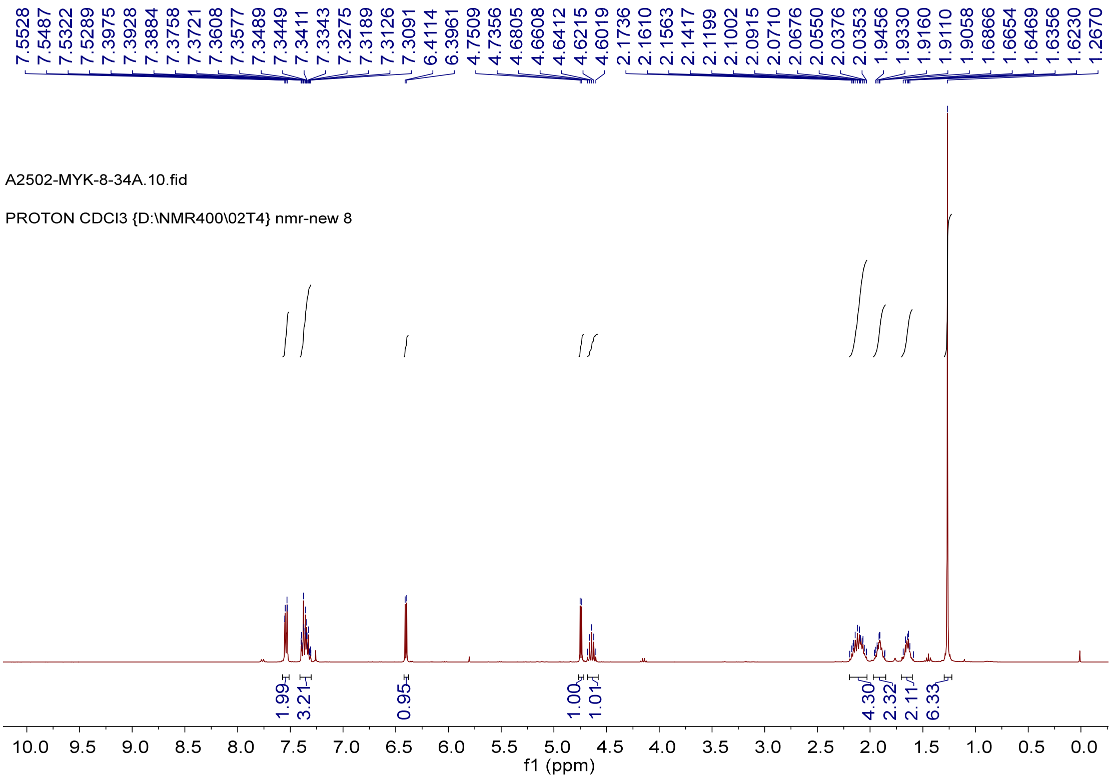

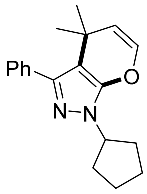

10j

^{1}H NMR (400 MHz, Chloroform-*d*)
^{13}C NMR (100 MHz, Chloroform-*d*)

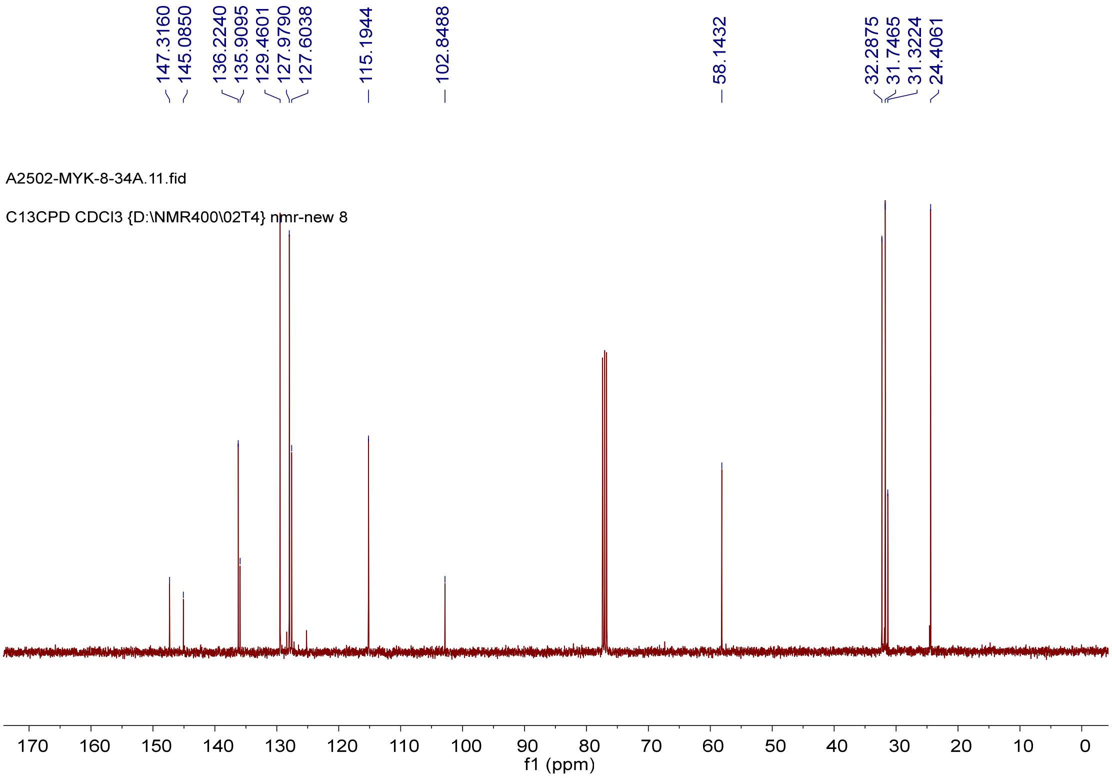

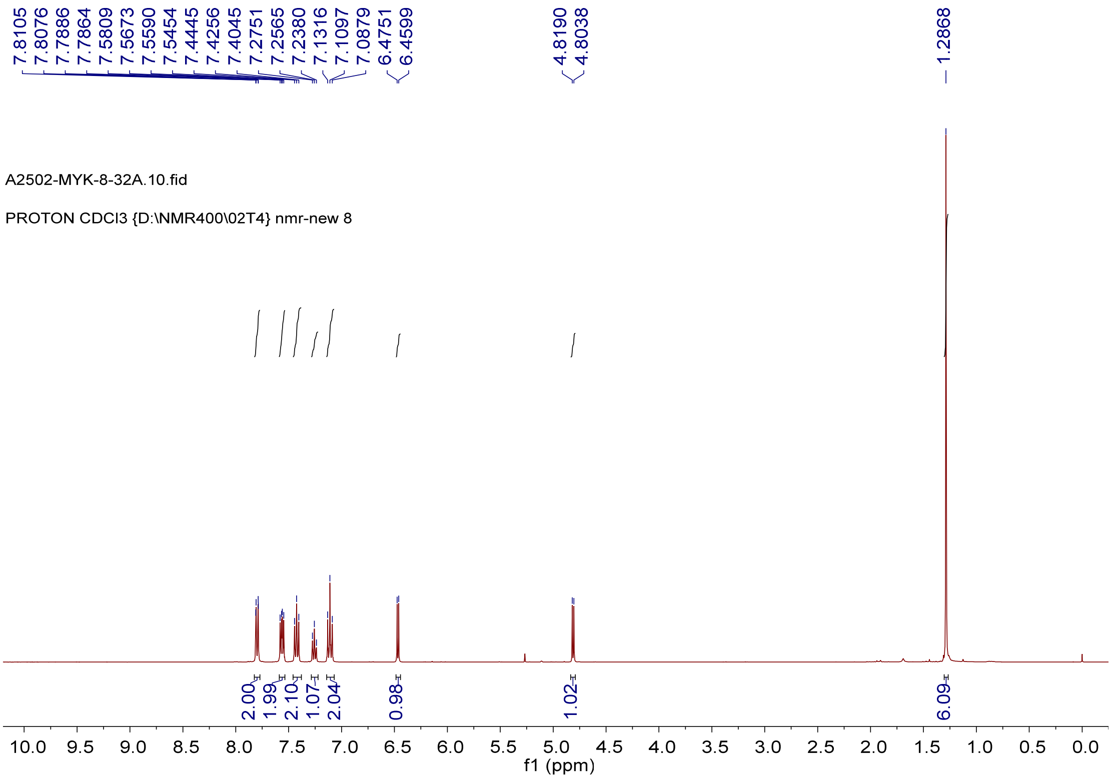

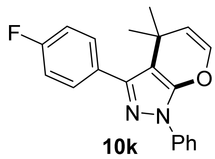

10k

^{1}H NMR (400 MHz, Chloroform-*d*)
^{13}C NMR (100 MHz, Chloroform-*d*)

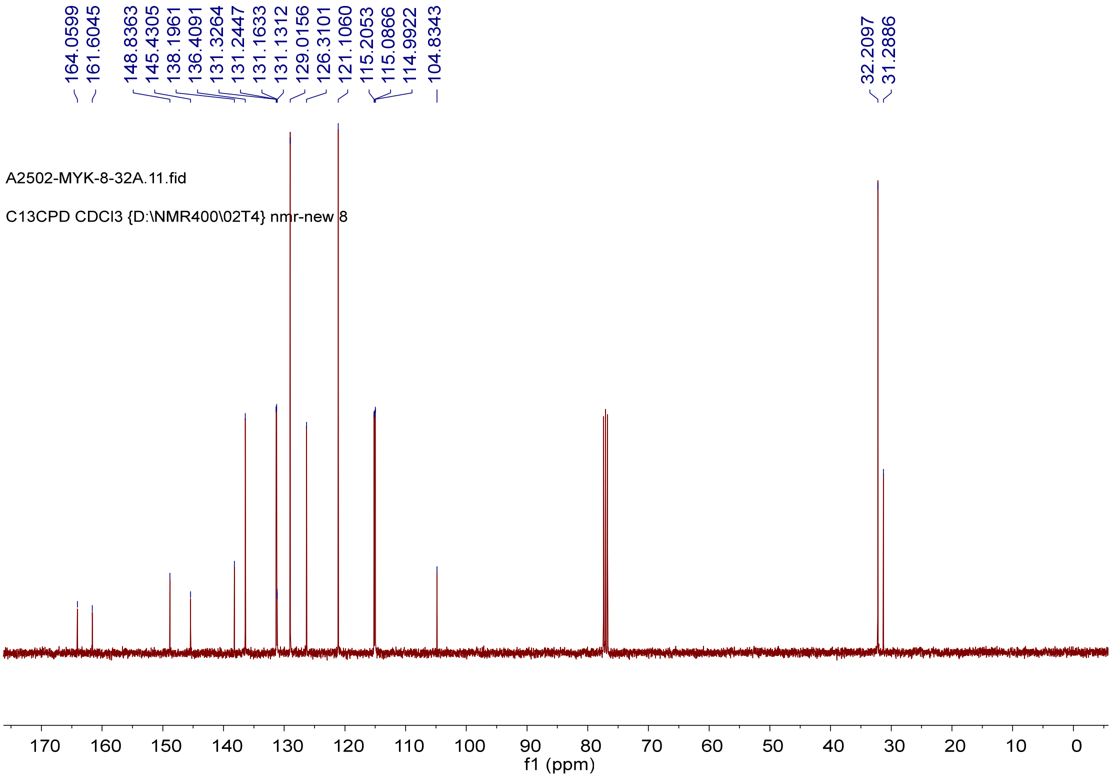

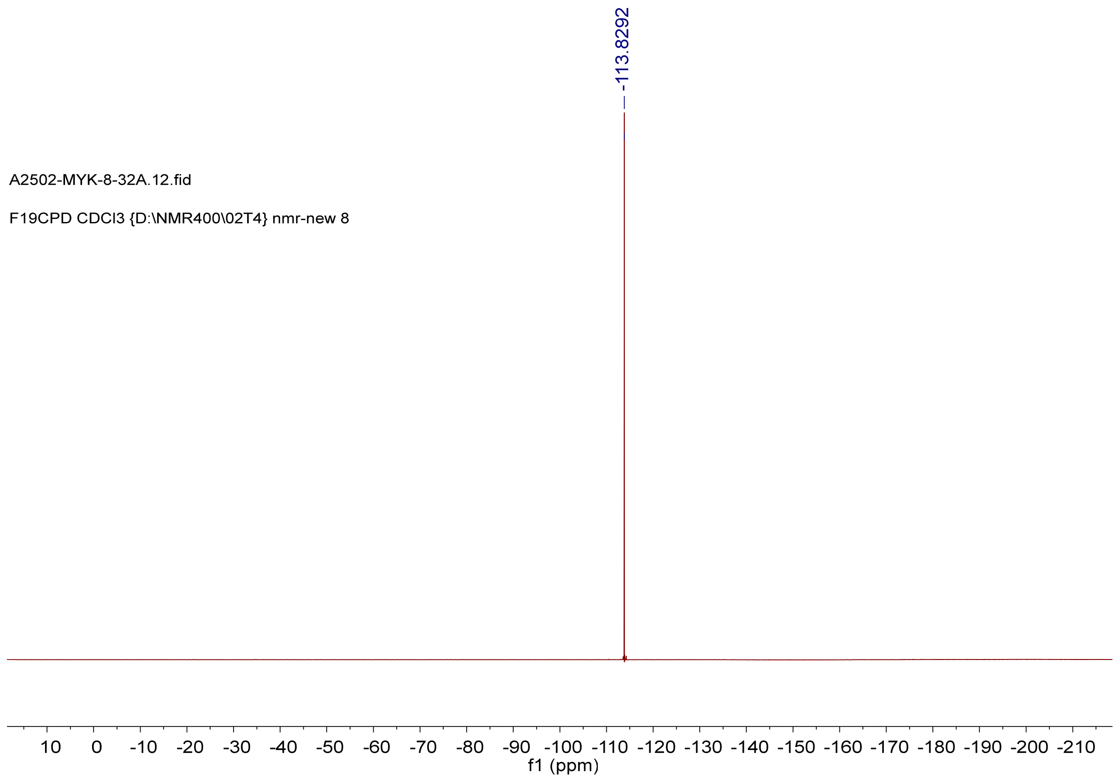

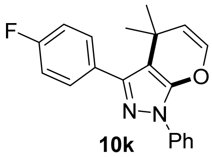

10k

^{19}F NMR (375 MHz, Chloroform-*d*)

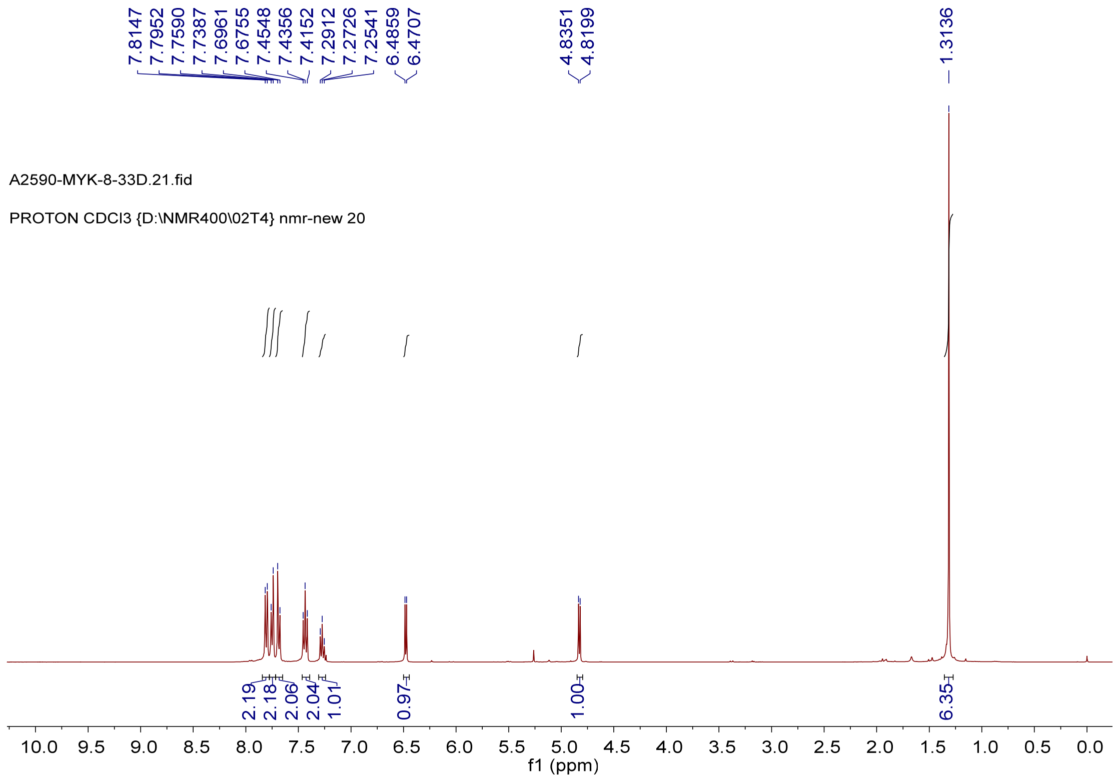

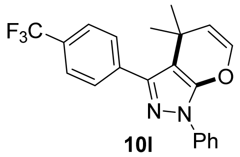

10l

1H NMR (400 MHz, Chloroform-*d*)
13C NMR (100 MHz, Chloroform-*d*)

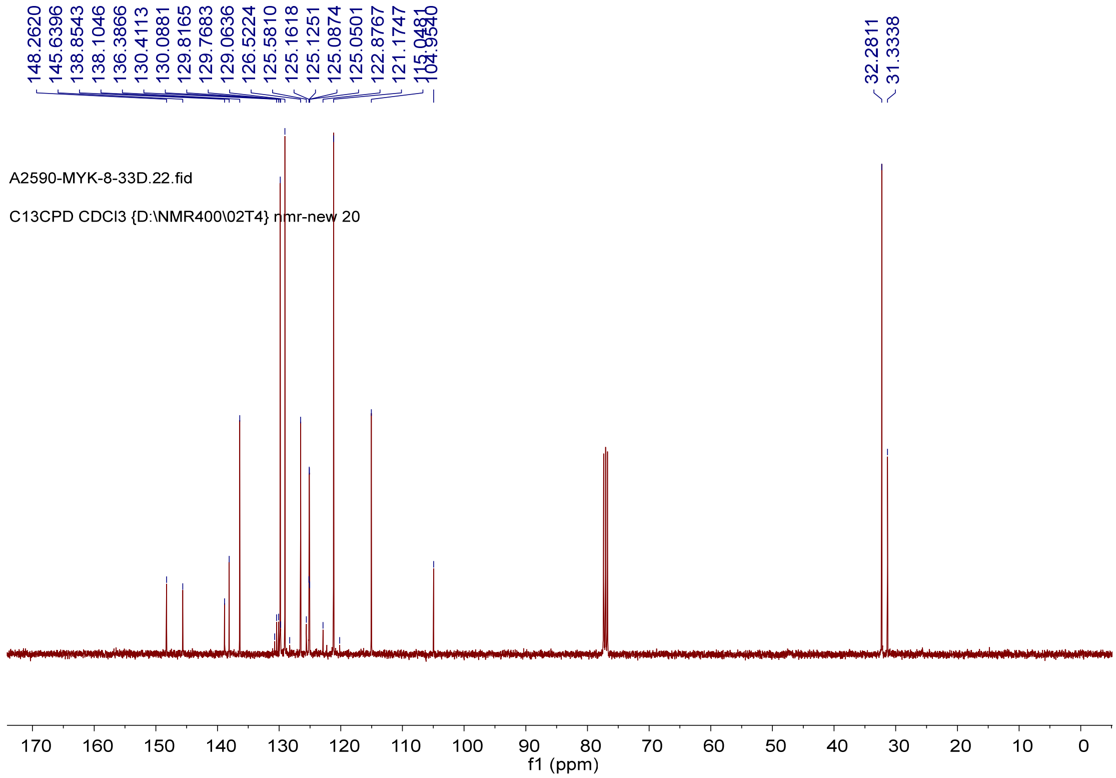

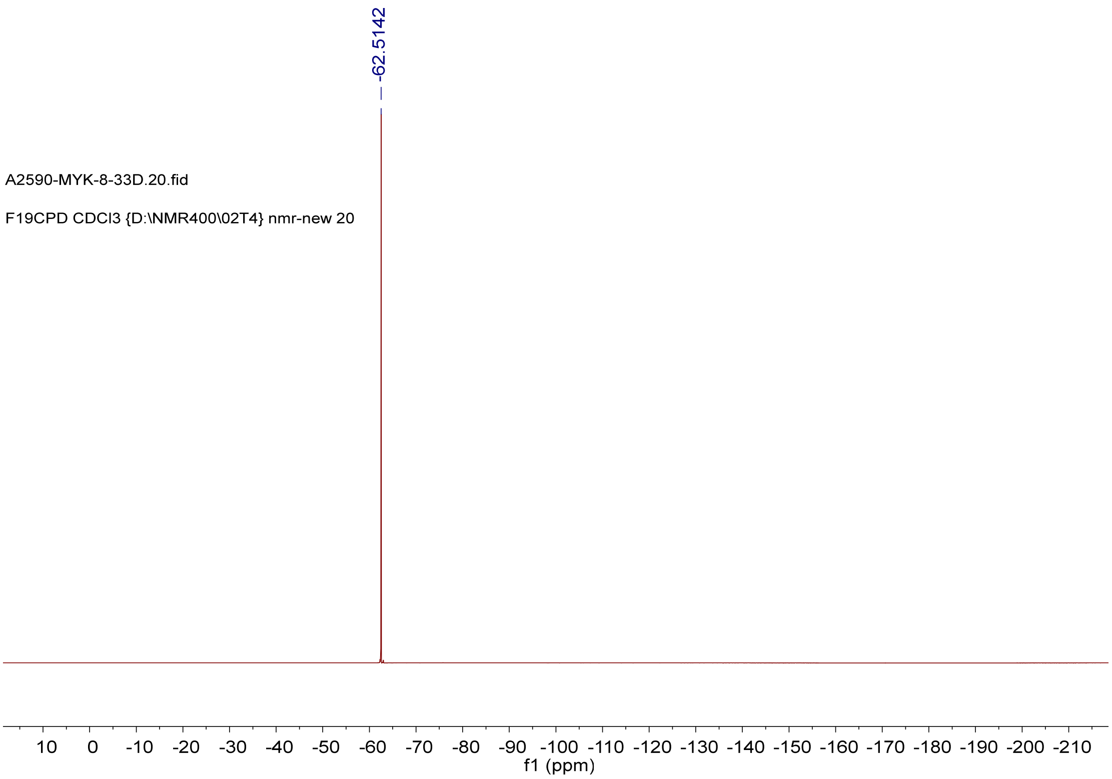

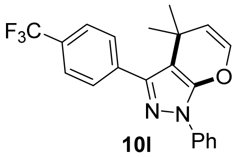

10l

^{19}**F NMR** (375 MHz, Chloroform-*d*)

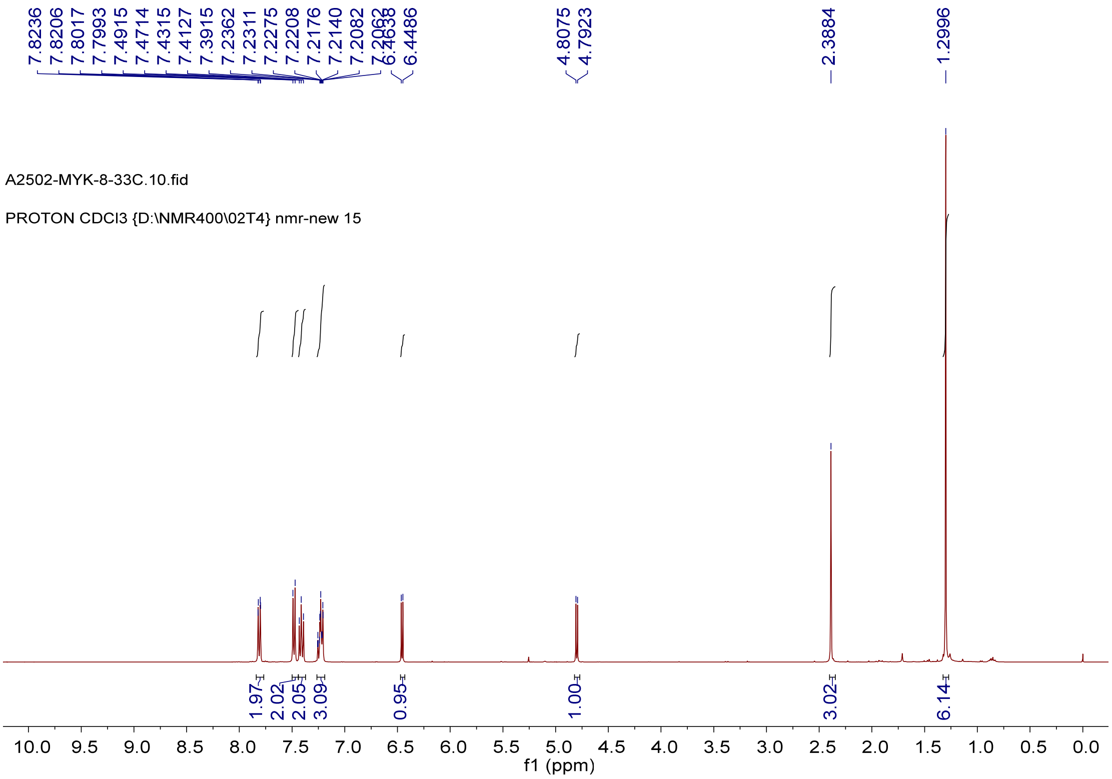

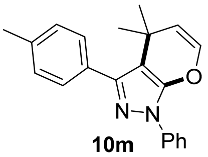

10m

^{1}H NMR (400 MHz, Chloroform-*d*)
^{13}C NMR (100 MHz, Chloroform-*d*)

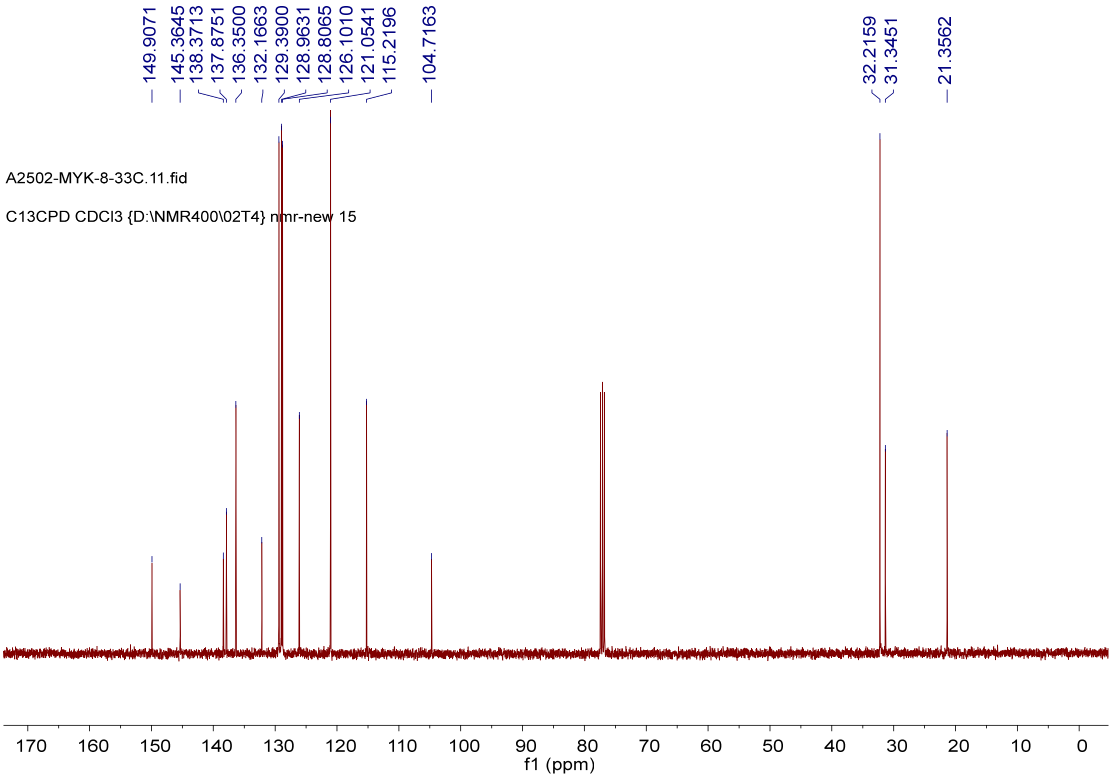

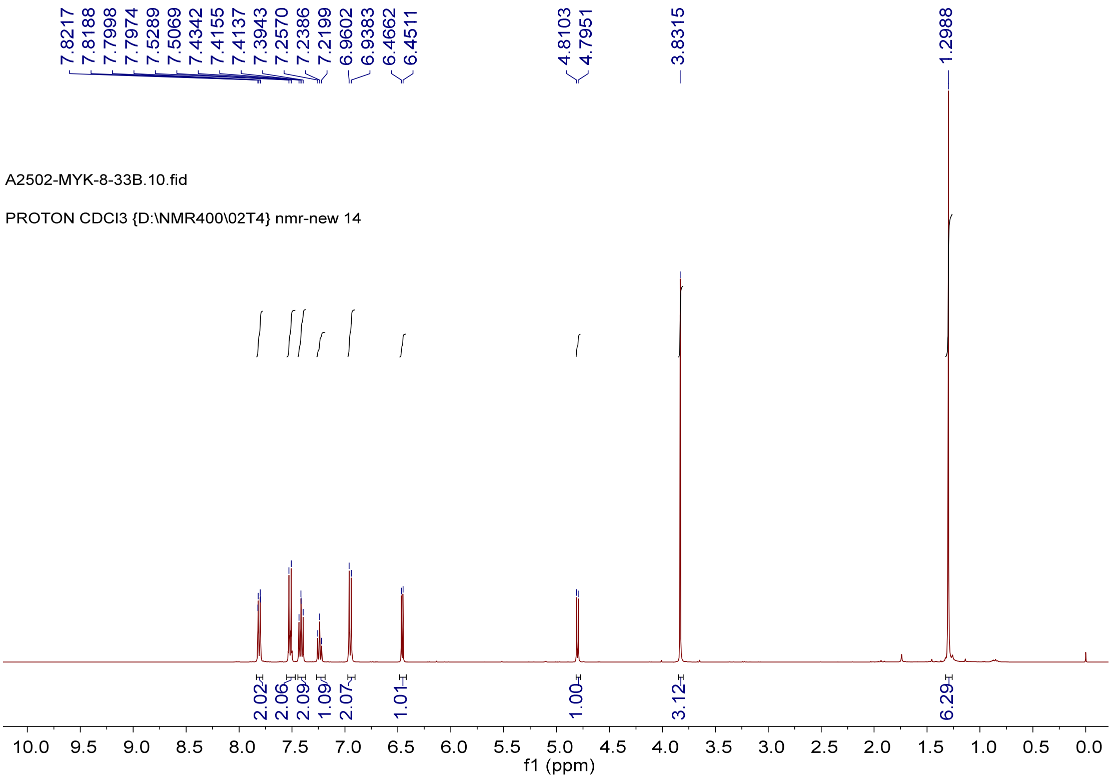

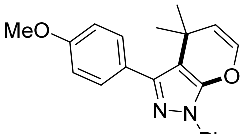

10n

^{1}H NMR (400 MHz, Chloroform-*d*)
^{13}C NMR (100 MHz, Chloroform-*d*)

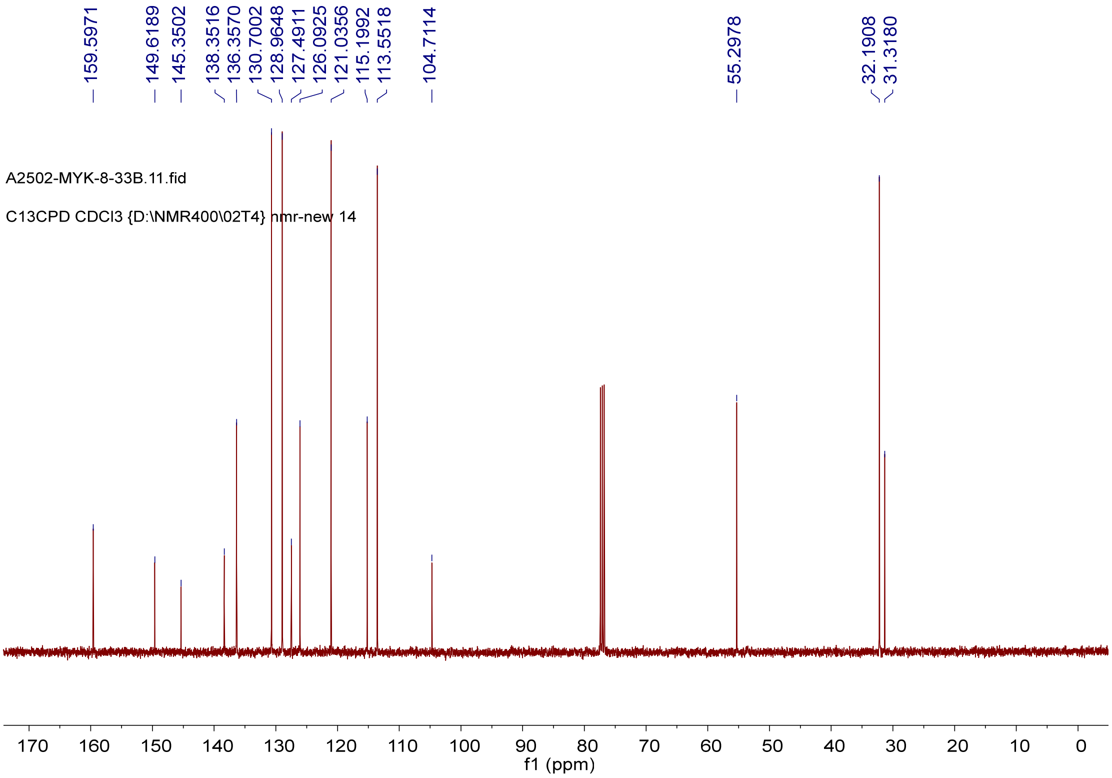

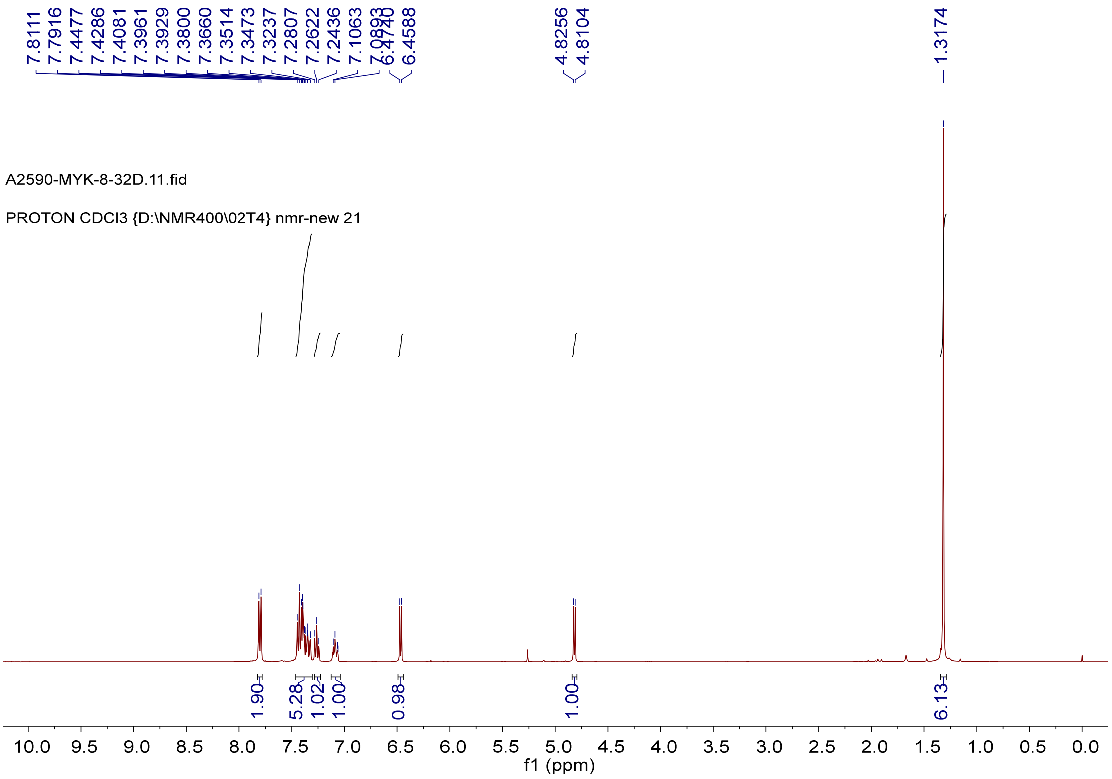

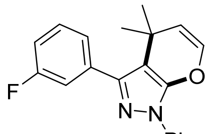

10o

^{1}H NMR (400 MHz, Chloroform-*d*)
^{13}C NMR (100 MHz, Chloroform-*d*)

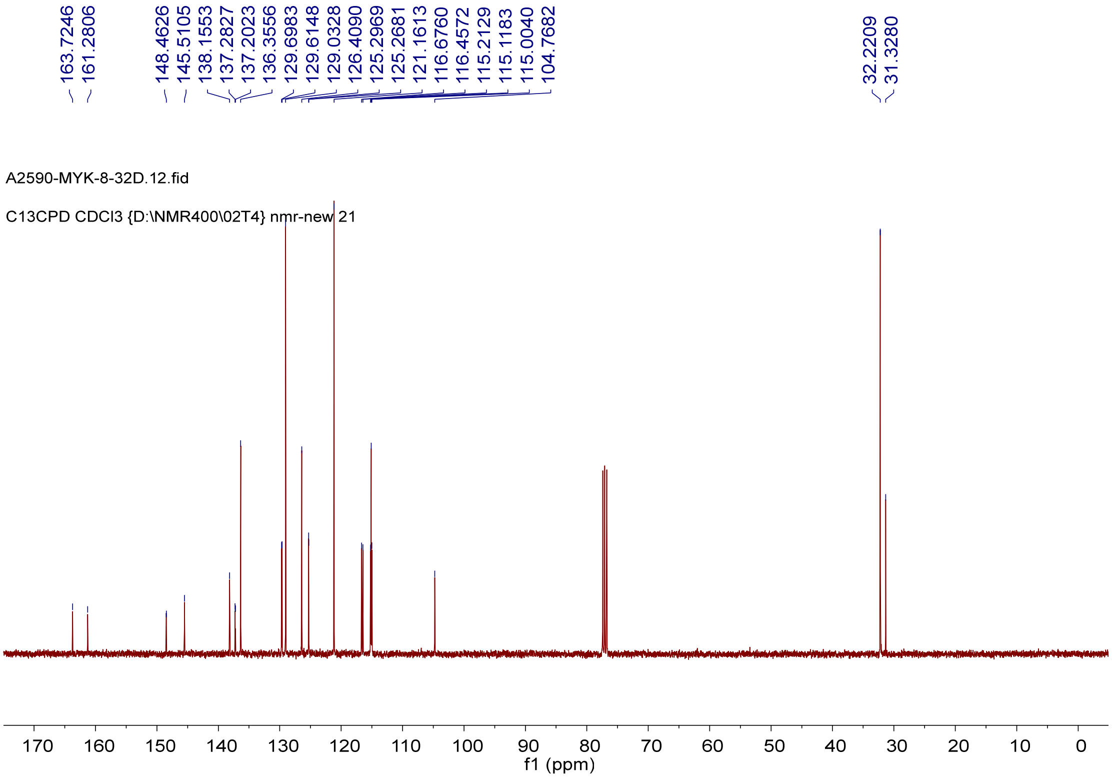

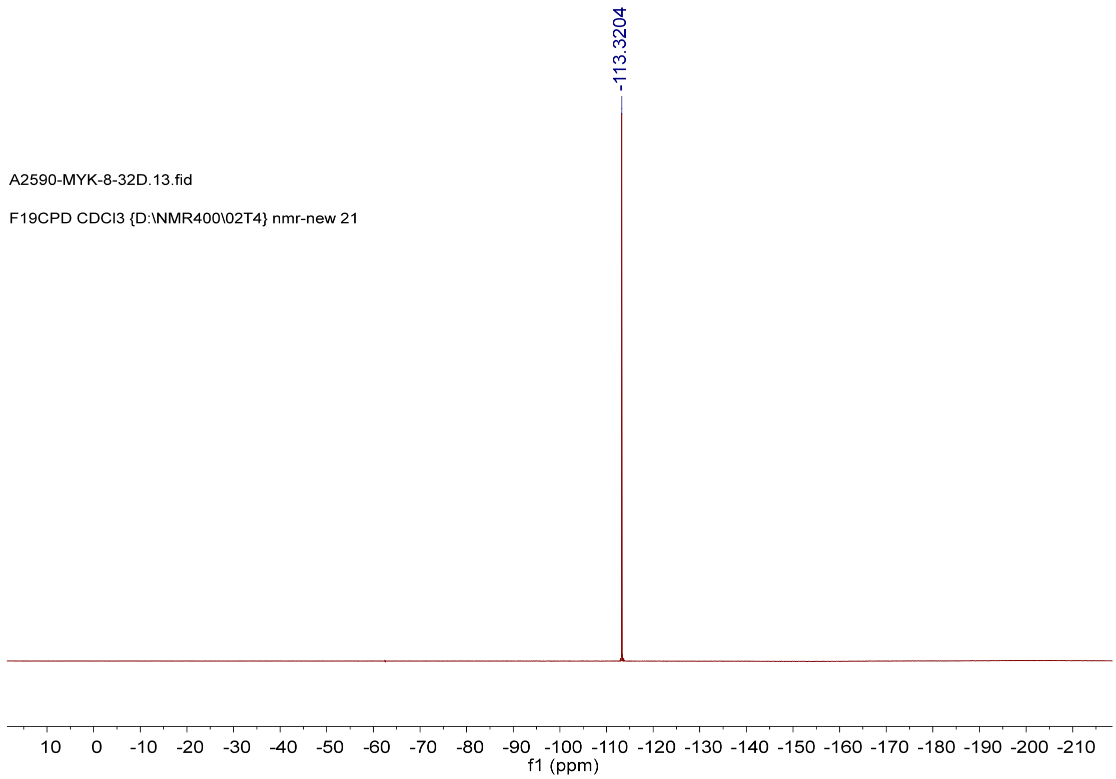

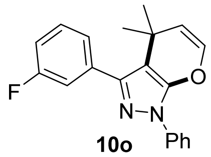

10o

^{19}F NMR (375 MHz, Chloroform-*d*)

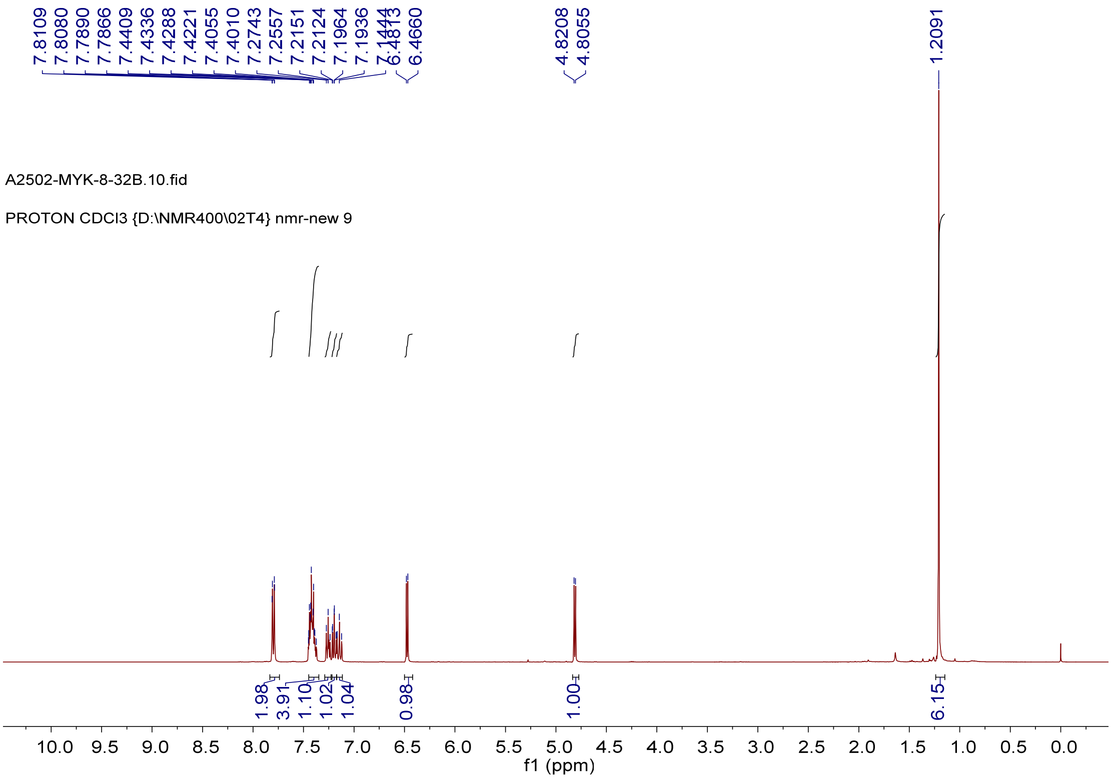

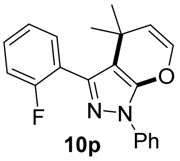

10p

^{1}H NMR (400 MHz, Chloroform-*d*)
^{13}C NMR (100 MHz, Chloroform-*d*)

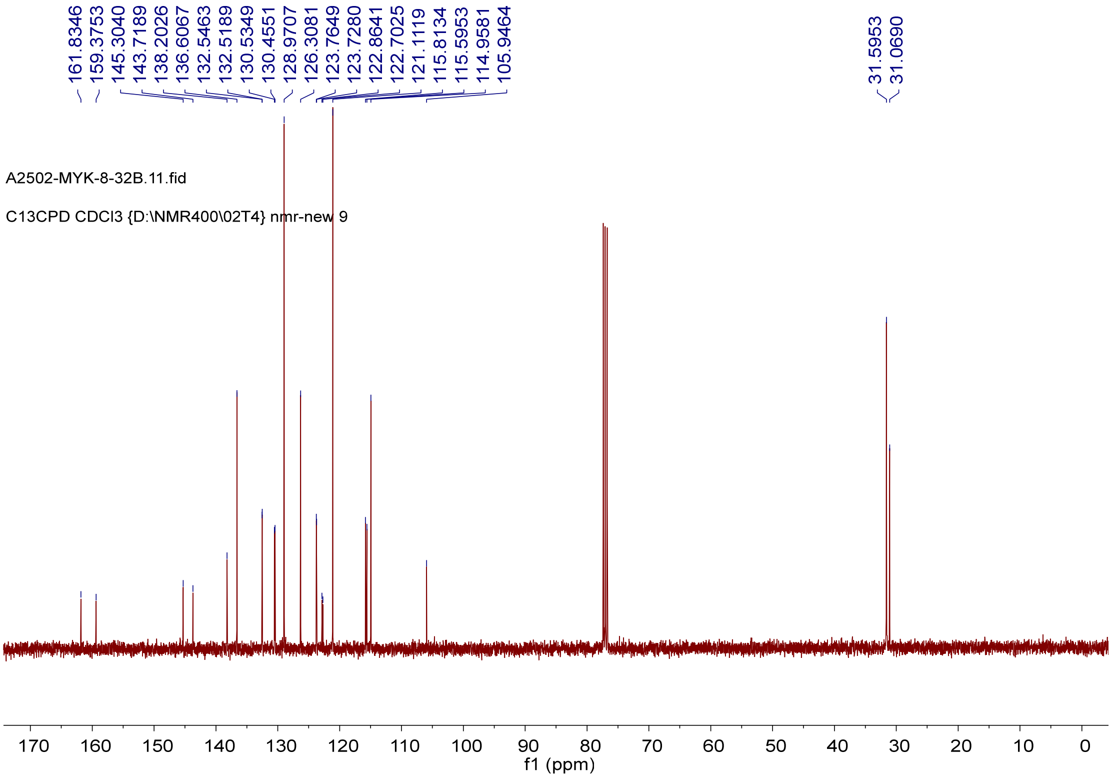

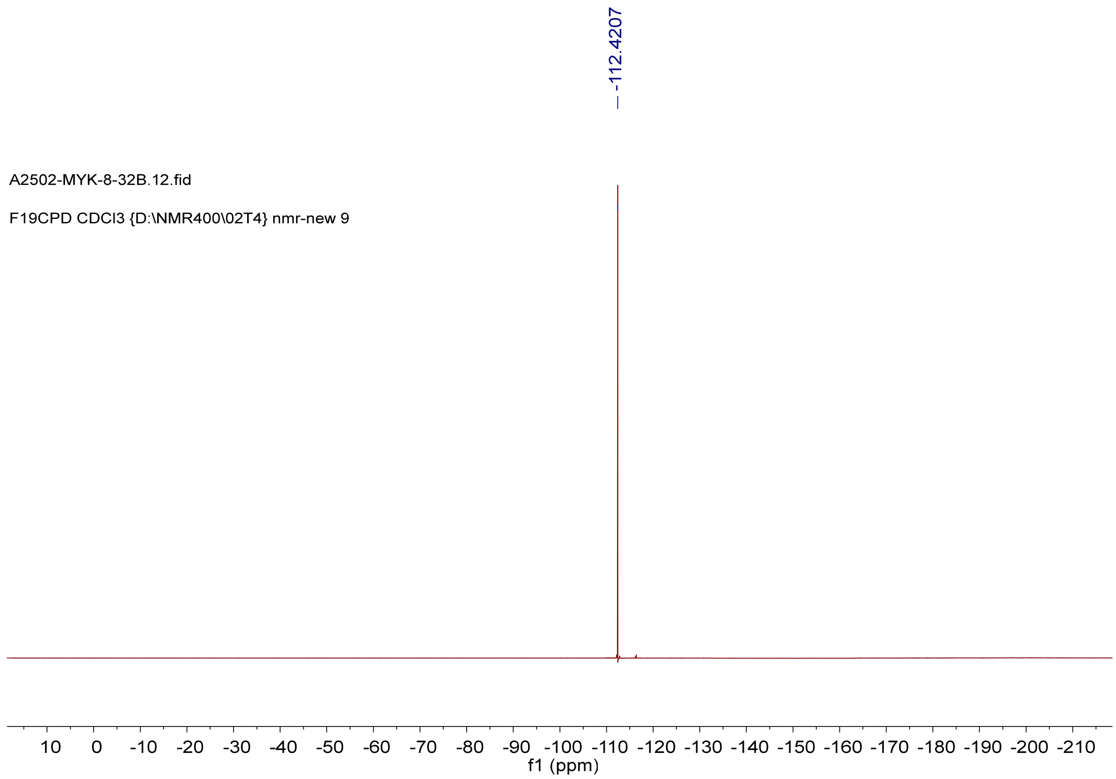

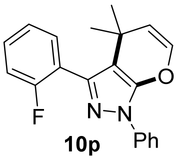

10p

^{19}F NMR (375 MHz, Chloroform-*d*)

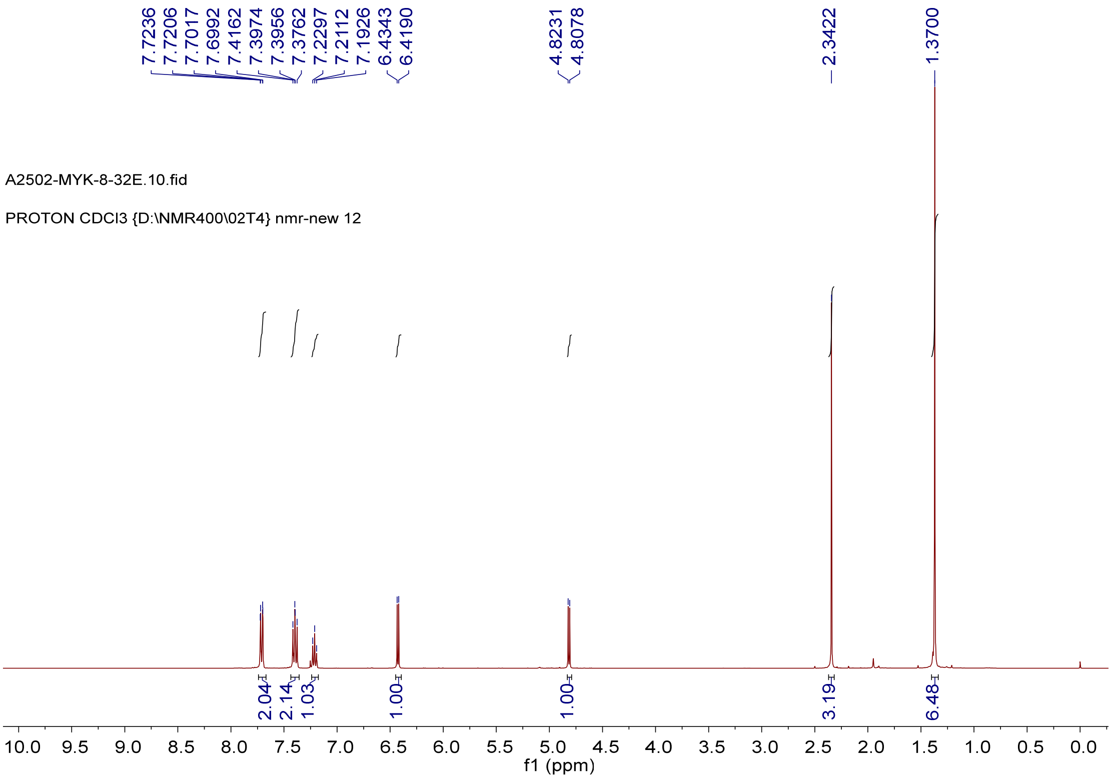

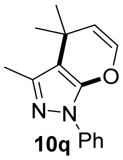

10q

^{1}H NMR (400 MHz, Chloroform-*d*)
^{13}C NMR (100 MHz, Chloroform-*d*)

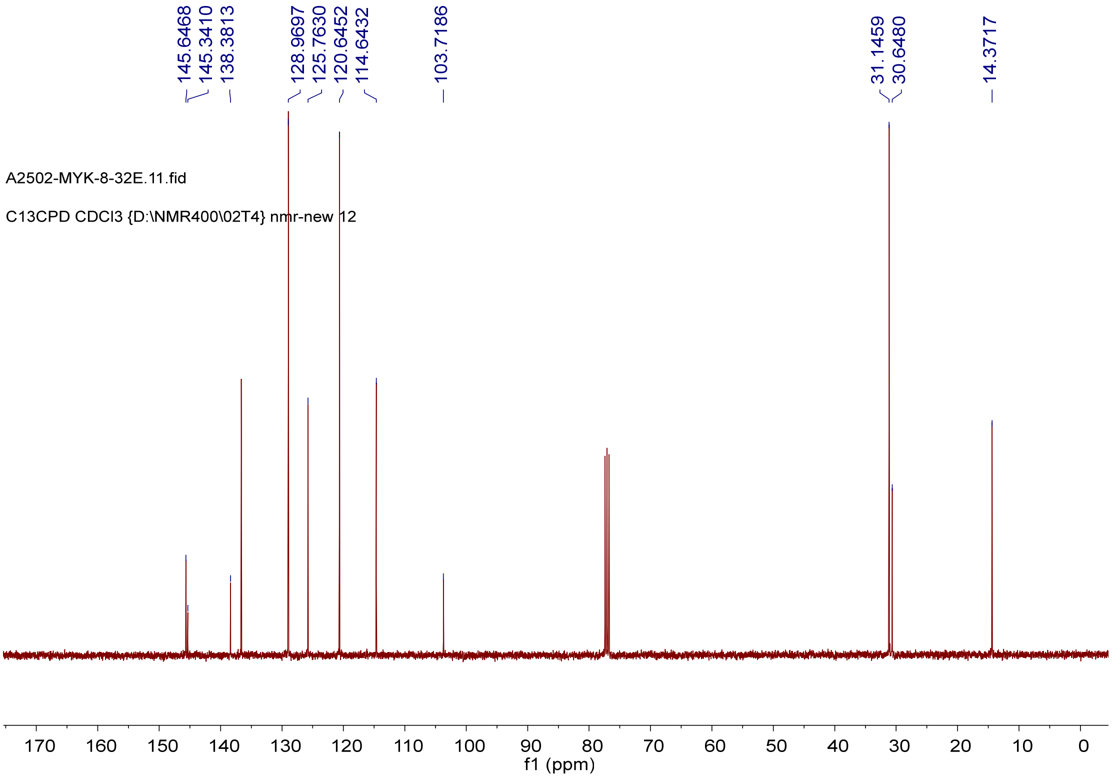

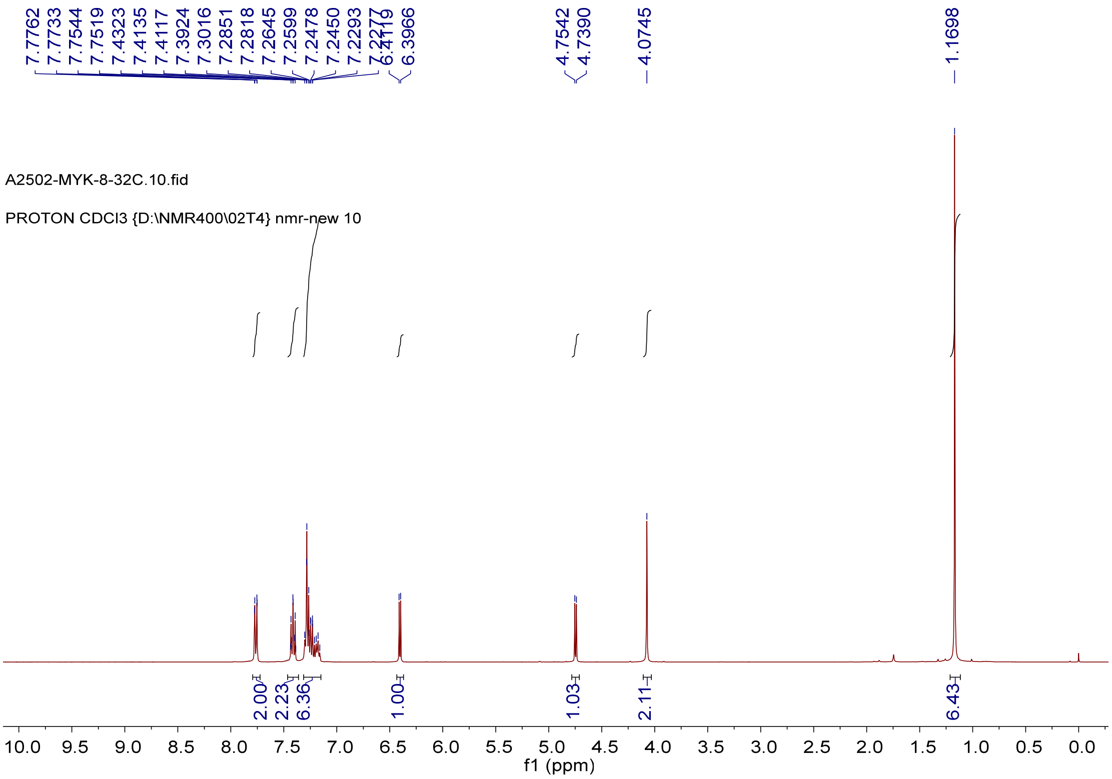

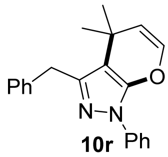

10r

^{1}H NMR (400 MHz, Chloroform-*d*)
^{13}C NMR (100 MHz, Chloroform-*d*)

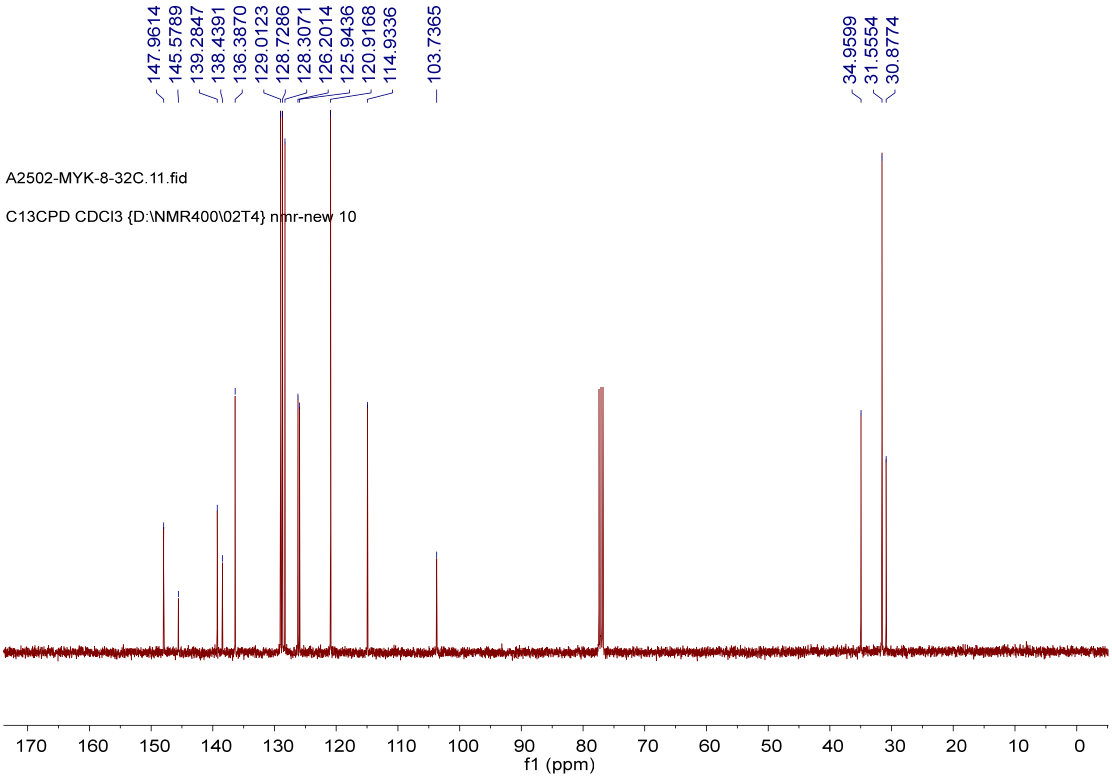

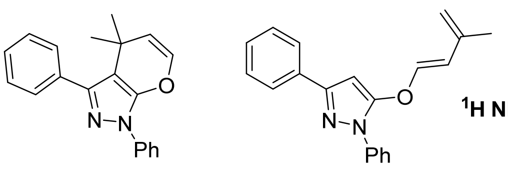

^{1}H NMR (400 MHz, Chloroform-*d*)

10a : 11a = 10.5 : 1

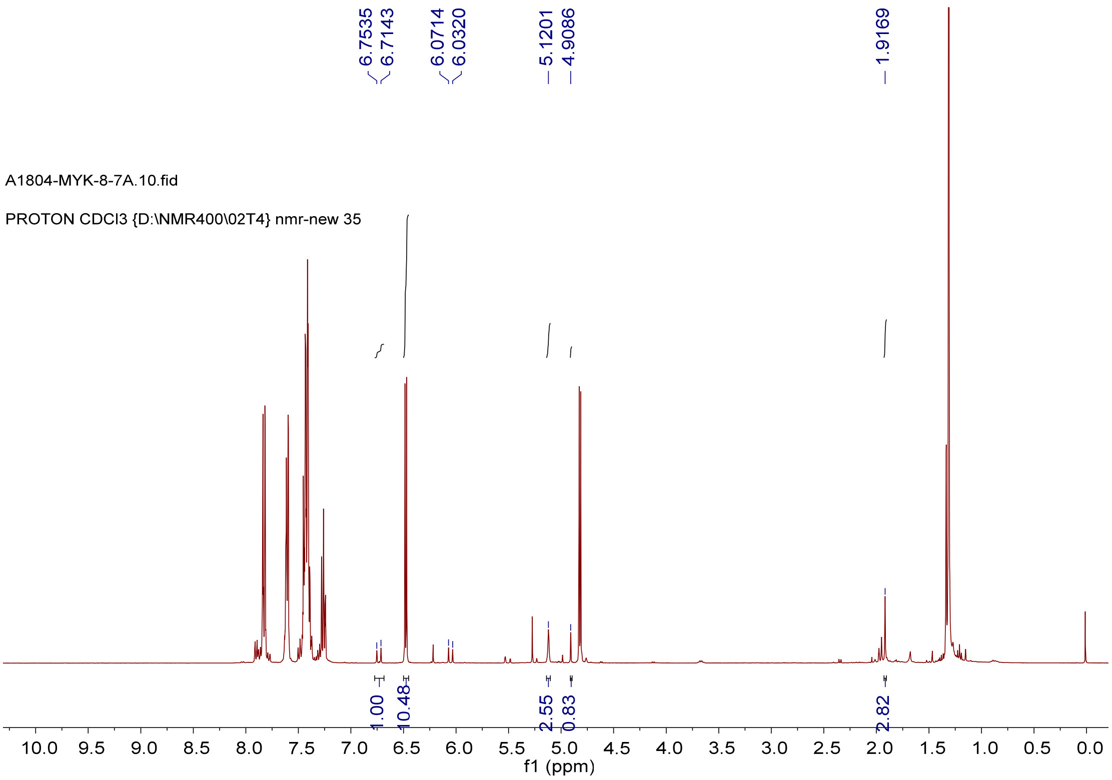

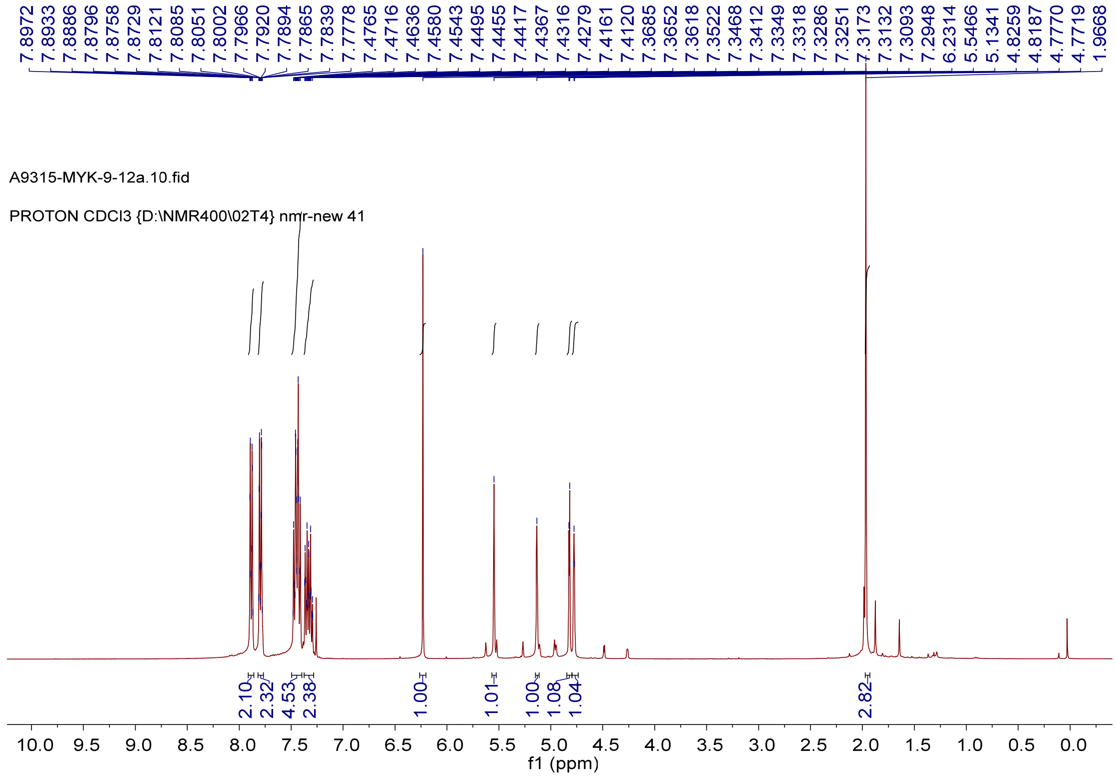

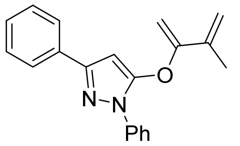

12a

^{1}H NMR (400 MHz, Chloroform-*d*)
^{13}C NMR (100 MHz, Chloroform-*d*)

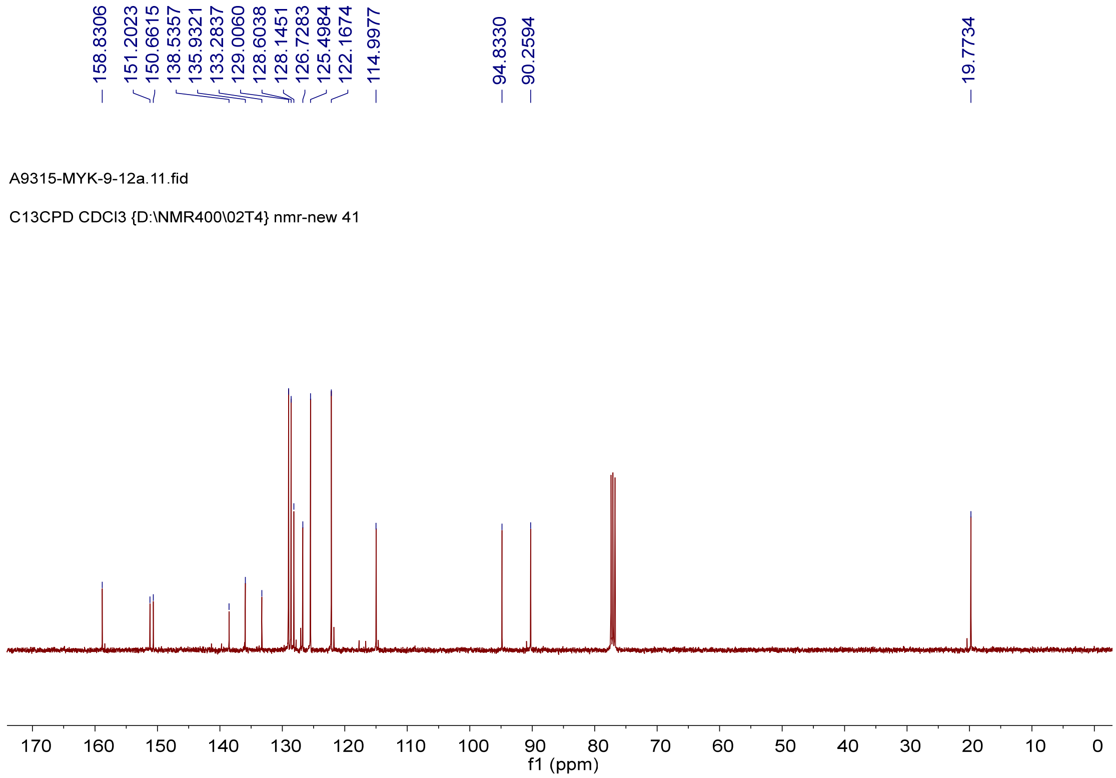

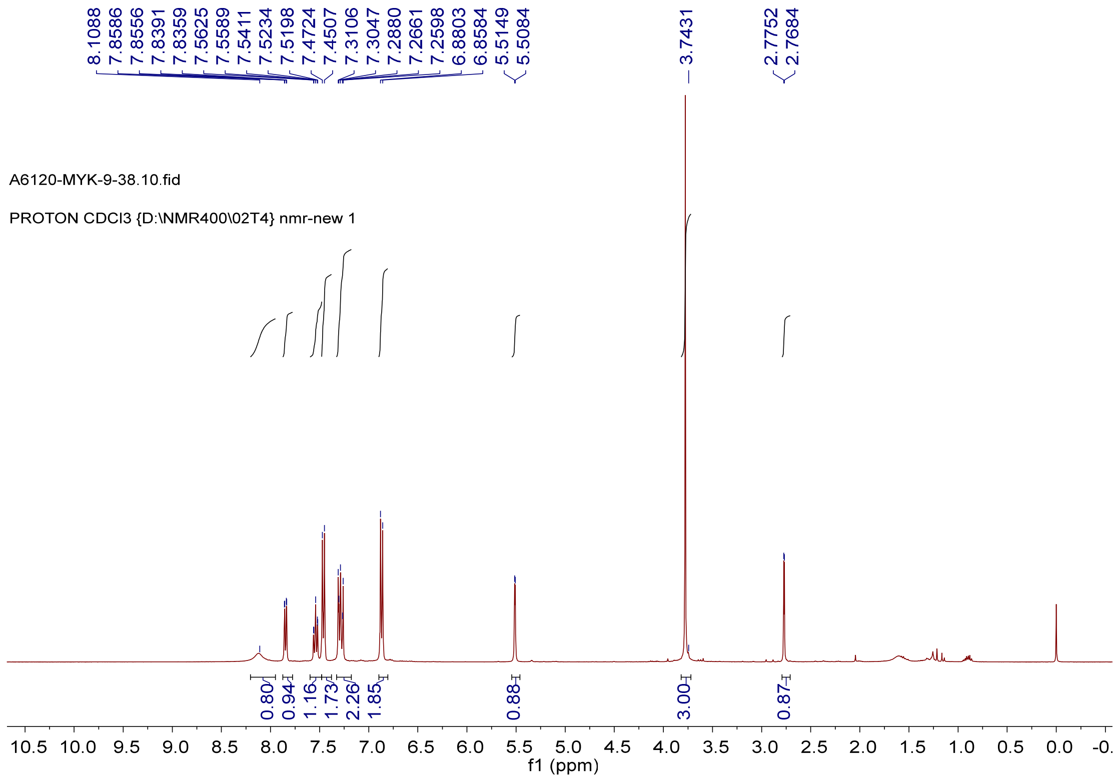

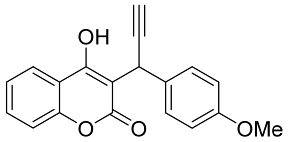

13

^{1}H NMR (400 MHz, Chloroform-*d*)
^{13}C NMR (100 MHz, Chloroform-*d*)

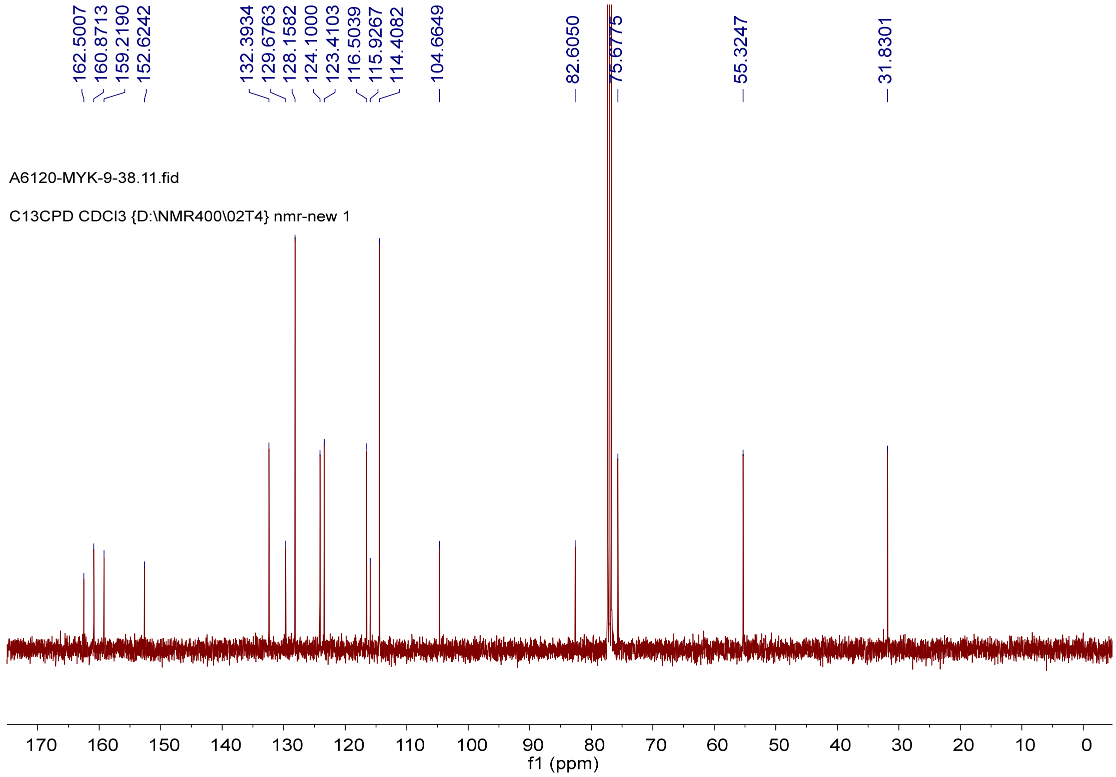

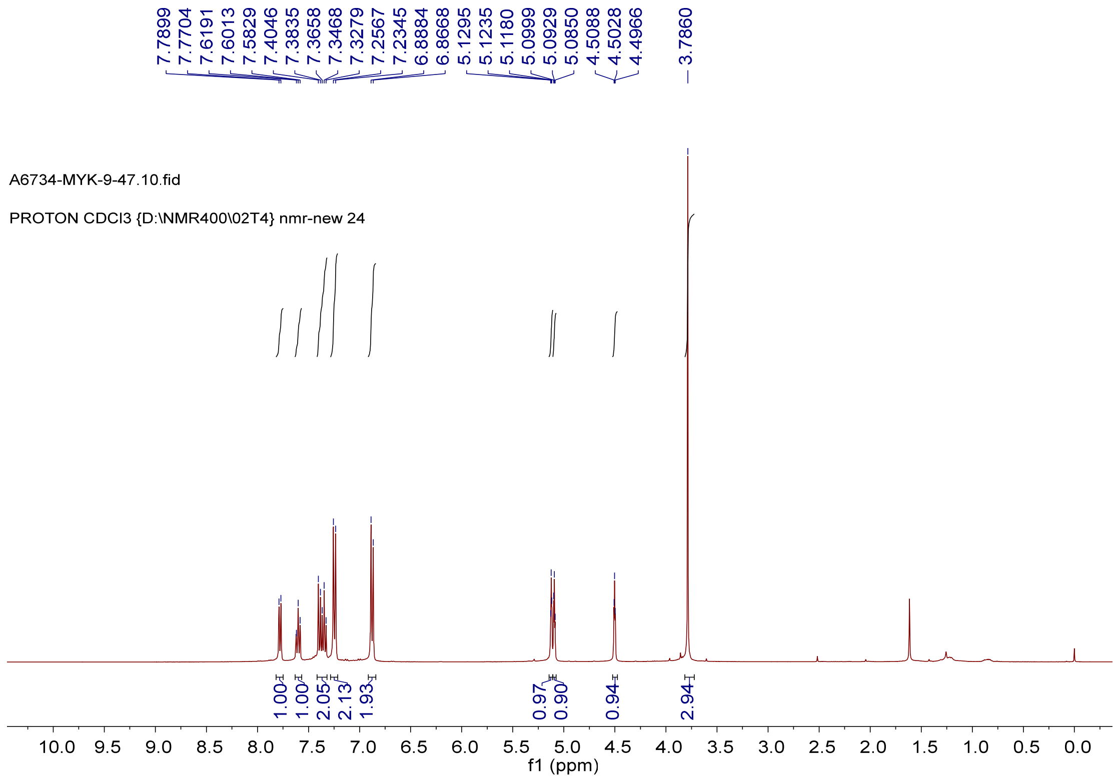

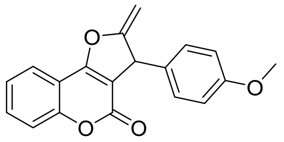

15

^{1}H NMR (400 MHz, Chloroform-*d*)
^{13}C NMR (100 MHz, Chloroform-*d*)

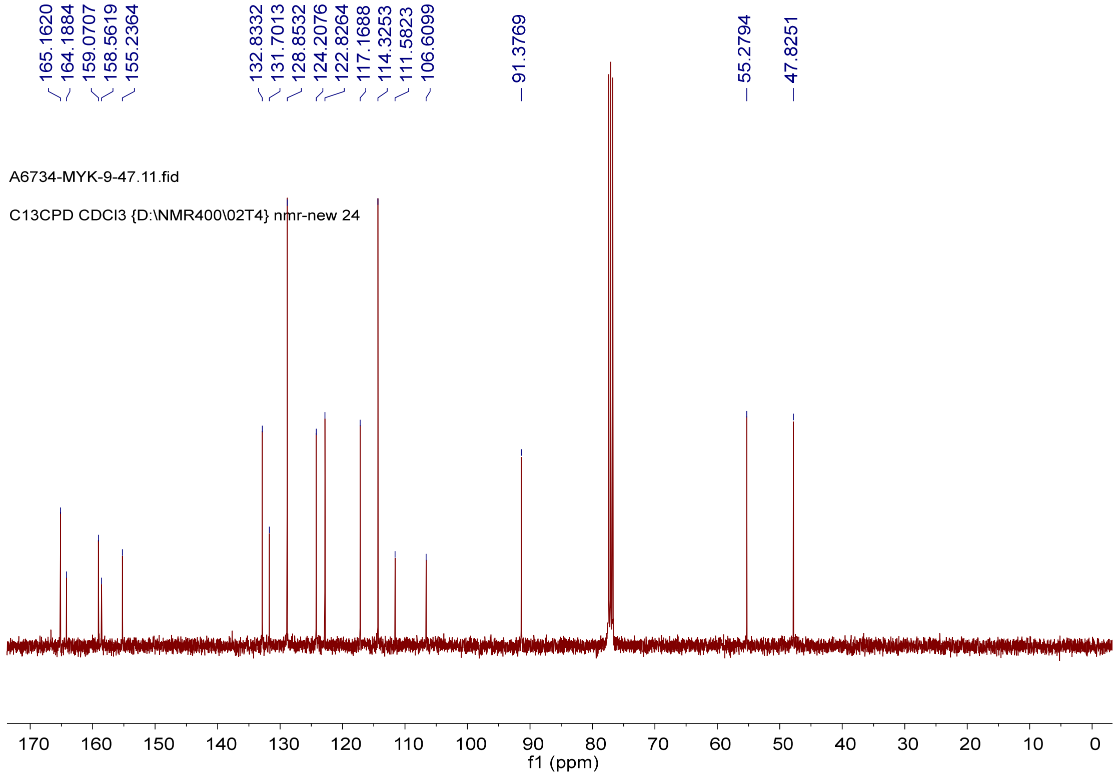

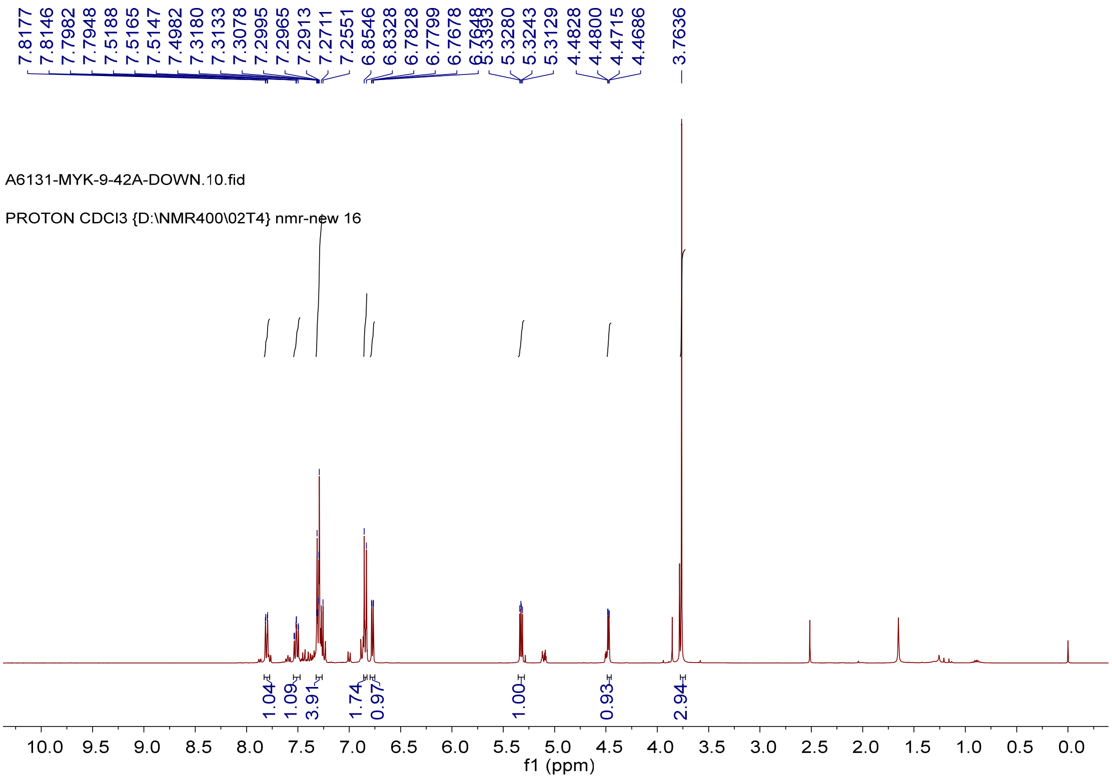

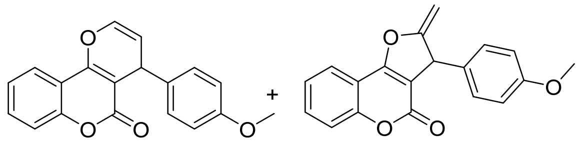

14 **15**

^{1}H NMR (400 MHz, Chloroform-*d*)
^{13}C NMR (100 MHz, Chloroform-*d*)

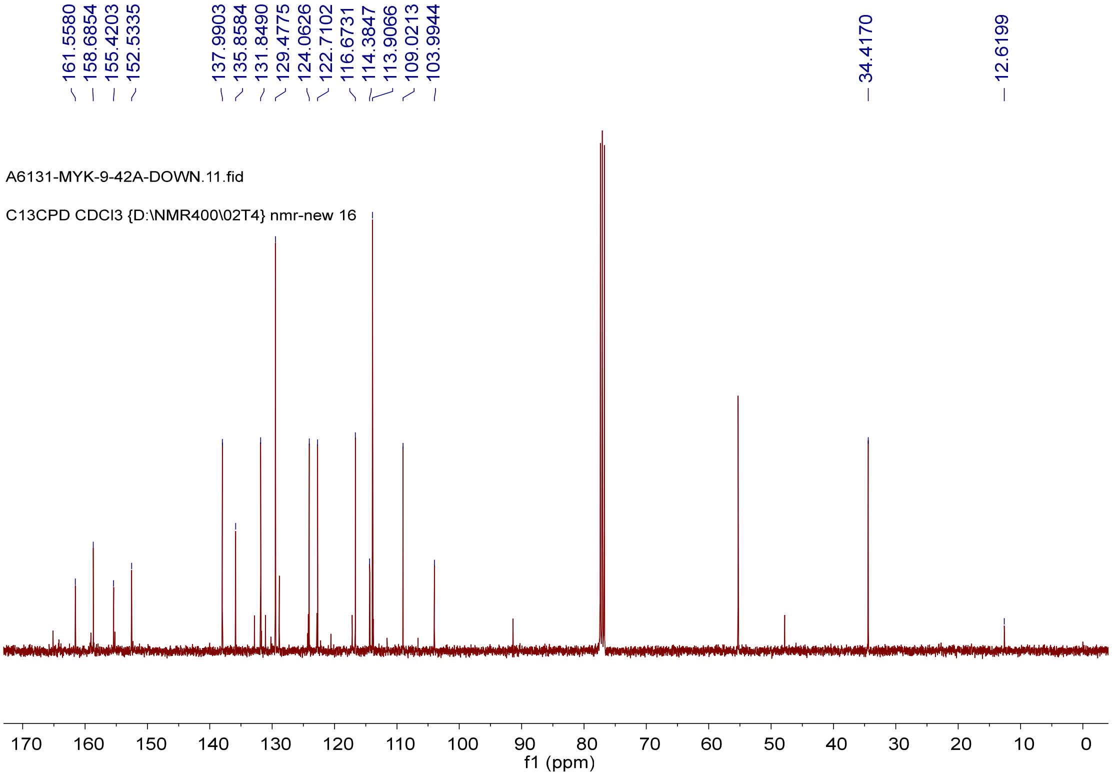

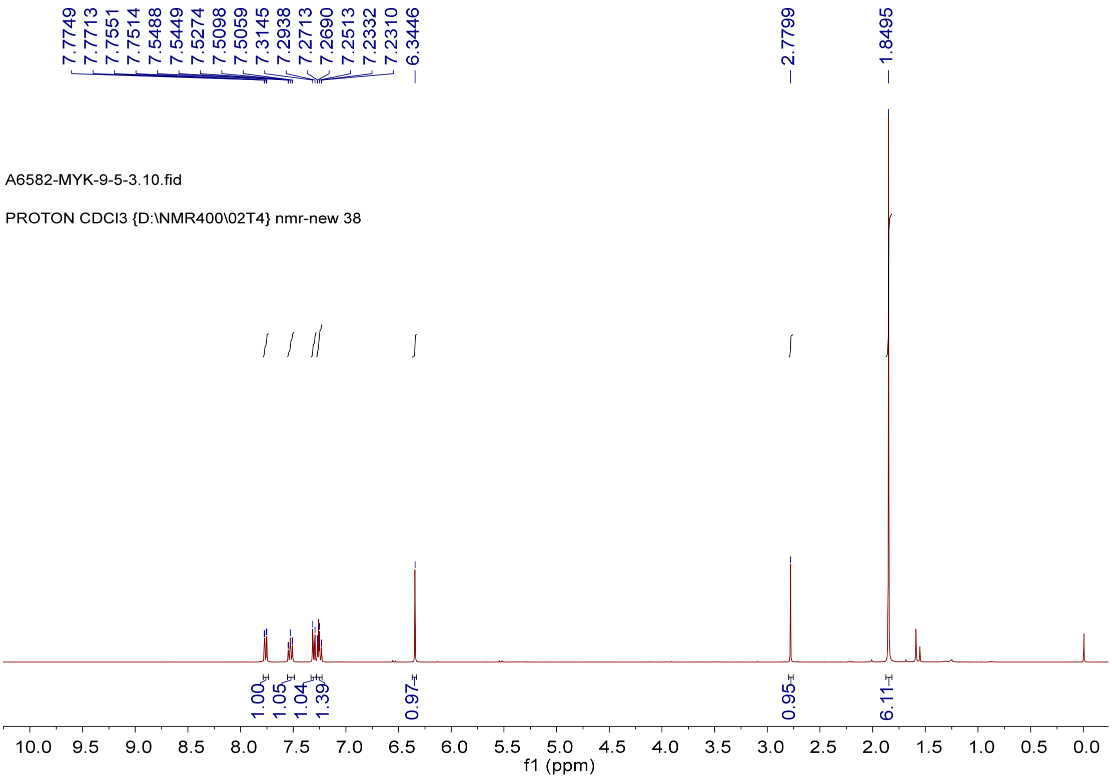

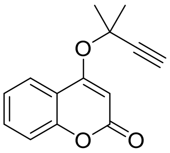

17

^{1}H NMR (400 MHz, Chloroform-*d*)
^{13}C NMR (100 MHz, Chloroform-*d*)

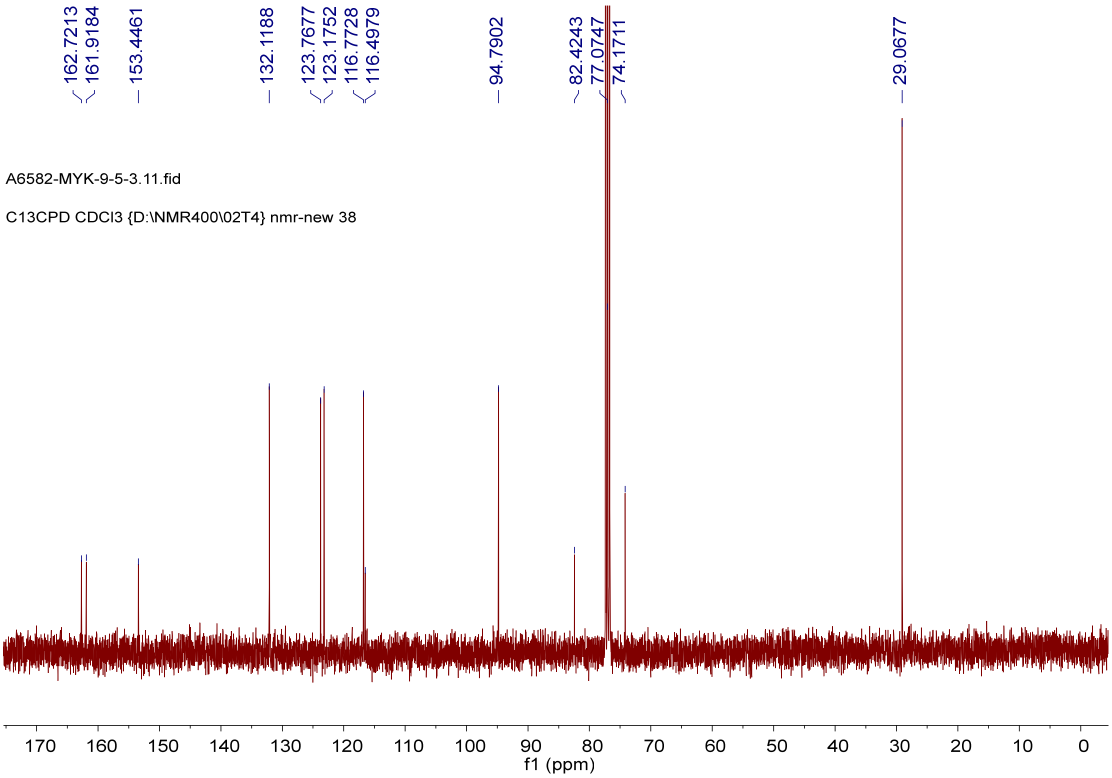

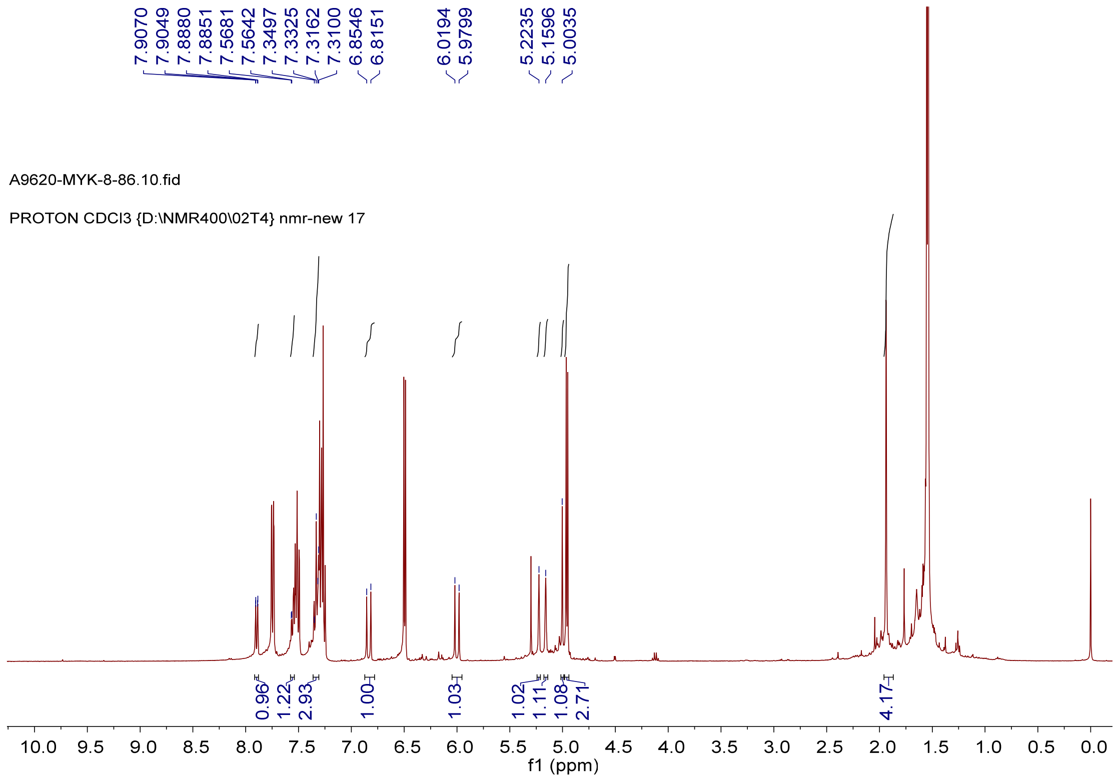

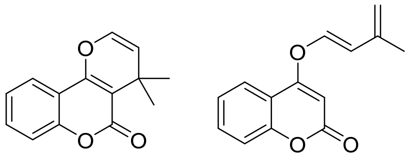

3a **7a**

^{1}H NMR (400 MHz, Chloroform-*d*)
^{13}C NMR (100 MHz, Chloroform-*d*)

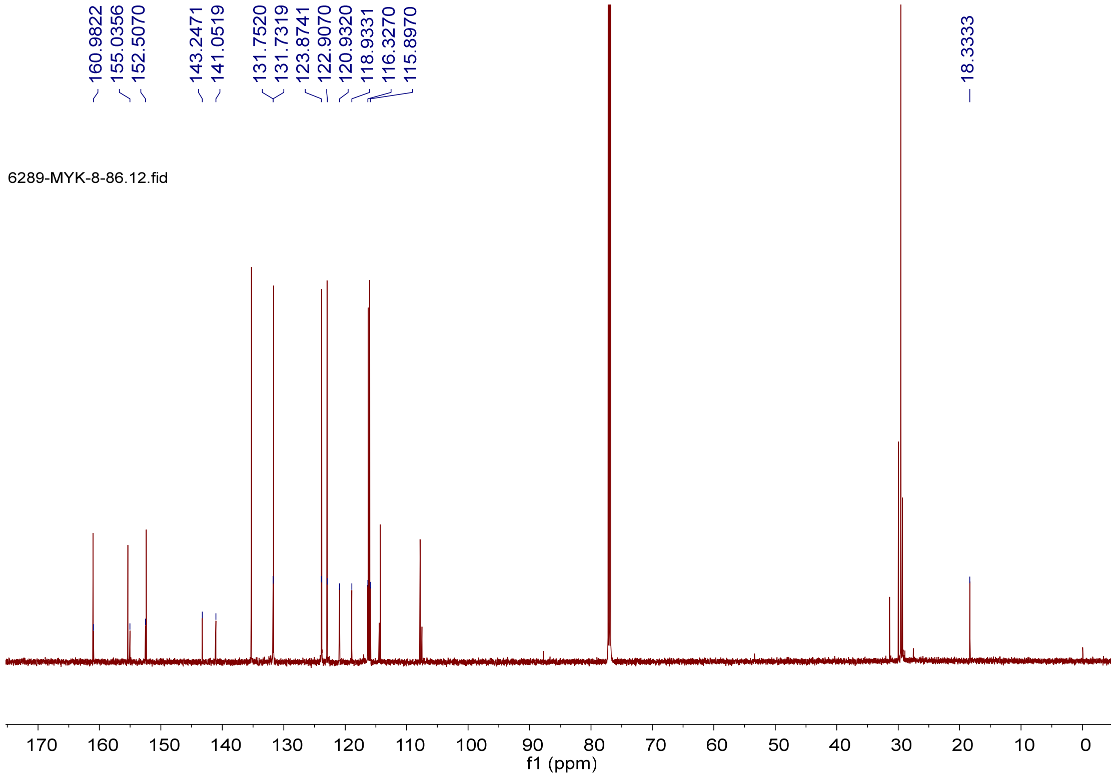

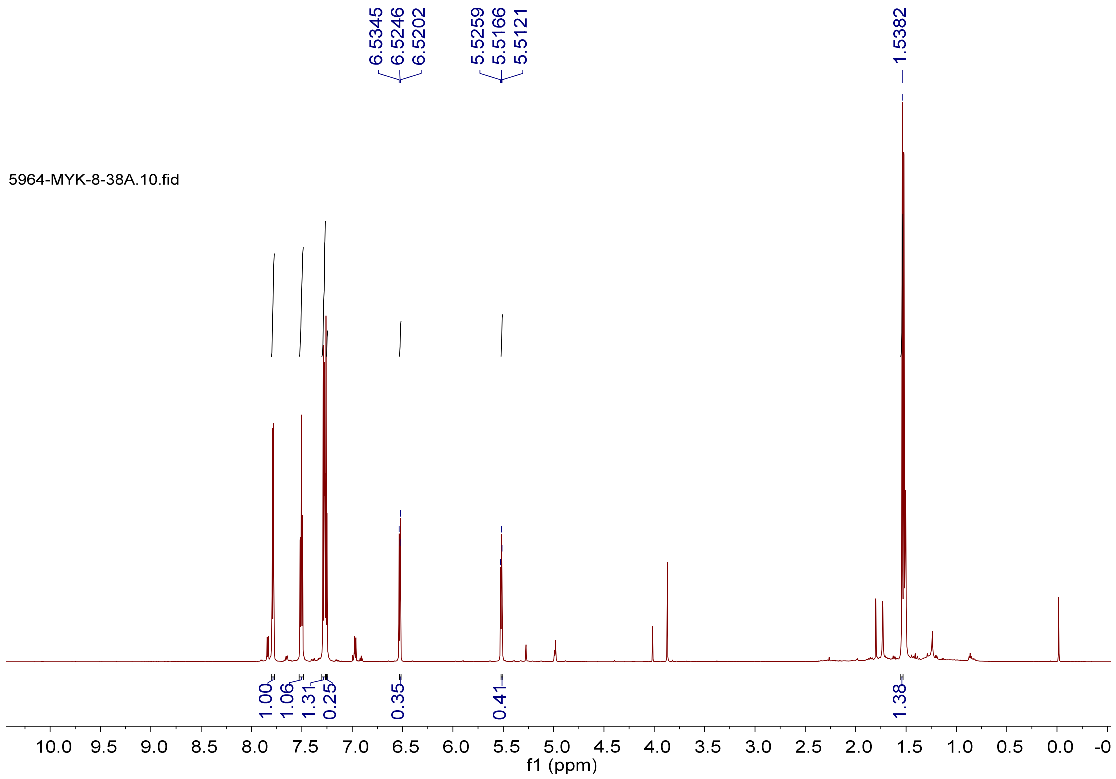

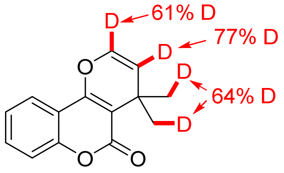

d-3a

¹H NMR (400 MHz, Chloroform-*d*)
²H NMR (107 MHz, Chloroform-*d*)

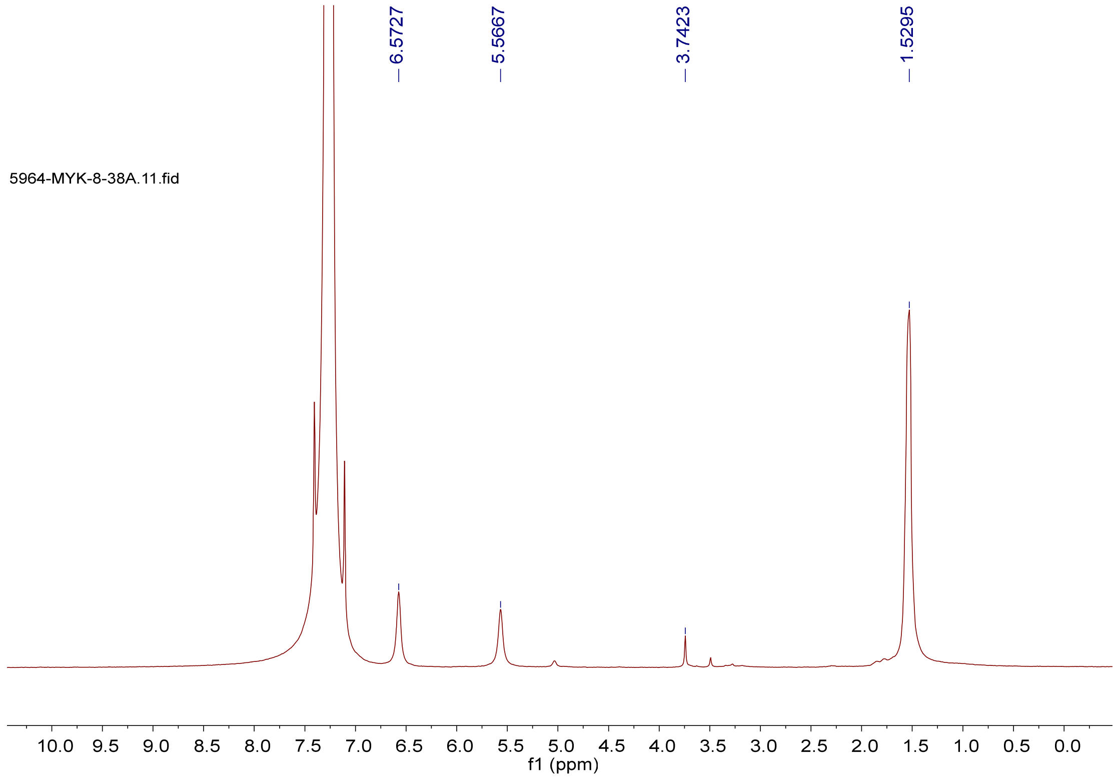

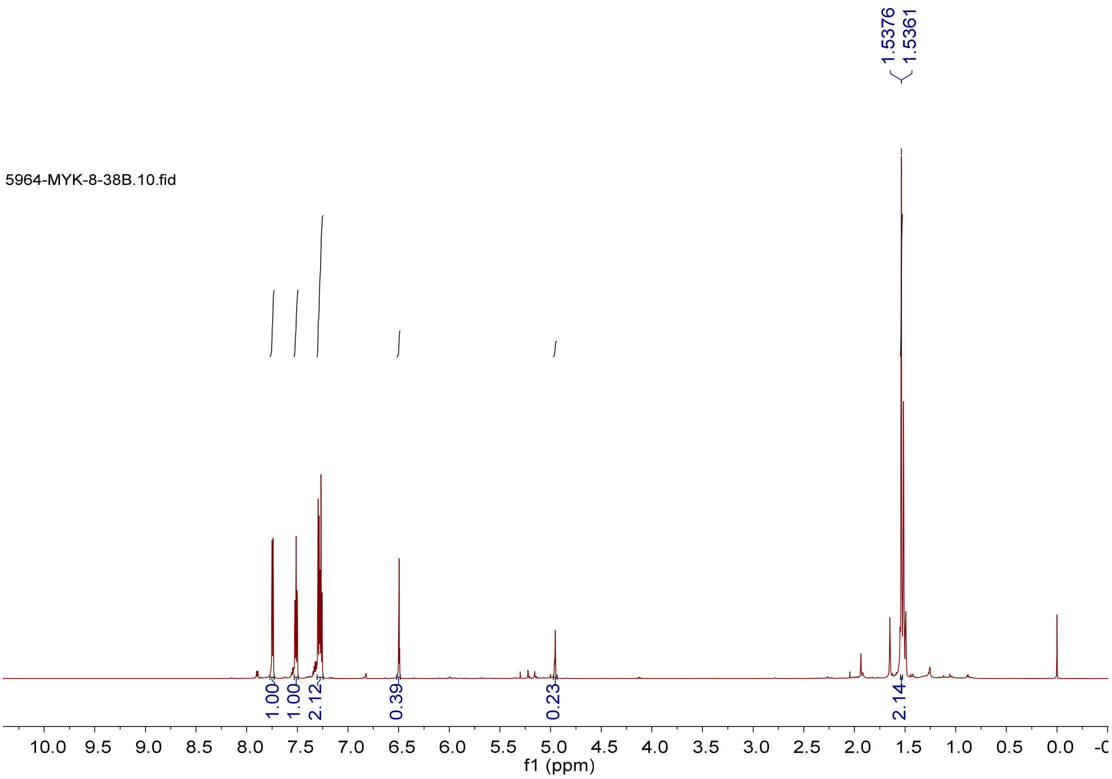

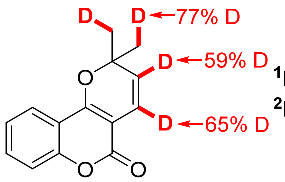

d-4a

^{1}H NMR (400 MHz, Chloroform-*d*)
^{2}H NMR (107 MHz, Chloroform-*d*)

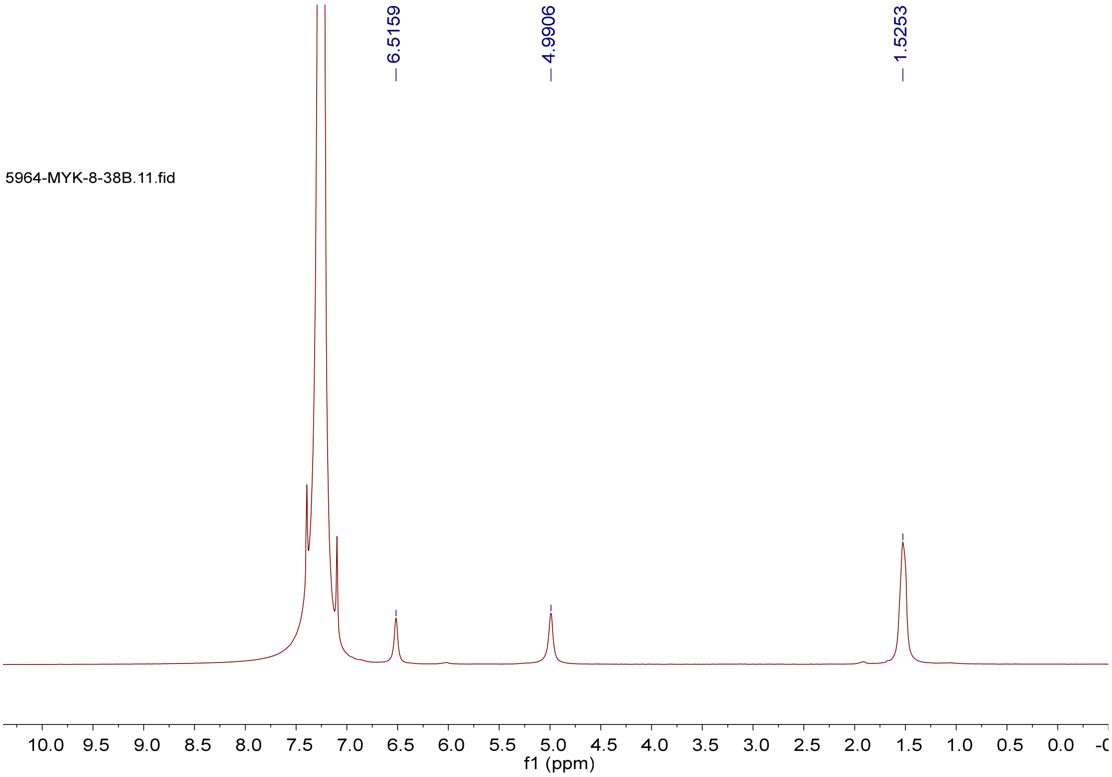

Supplement: Supplementary file 1 — Supporting Information [file ADVS-12-e11331-s001.pdf]
