# Supplementary material for: Dual‐Ligand Strategy in Rh‐Catalyzed Sequential Hydrofunctionalization of Valylene
Source: Adv Sci (Weinh). 2025 Jul 30;12(40):e11331. doi: 10.1002/advs.202511331 (PMC12561339; doi:10.1002/advs.202511331)

checkCIF/PLATON report

Structure factors have been supplied for datablock(s) 3d

THIS REPORT IS FOR GUIDANCE ONLY. IF USED AS PART OF A REVIEW PROCEDURE FOR PUBLICATION, IT SHOULD NOT REPLACE THE EXPERTISE OF AN EXPERIENCED CRYSTALLOGRAPHIC REFEREE.

No syntax errors found. CIF dictionary Interpreting this report

Datablock: 3d

Bond precision:	C-C = 0.0052 A		Wavelength=0.71073
Cell:	a=16.9170(8)	b=6.9625(4)	c=20.9672(11)
	alpha=90	beta=90	gamma=90
Temperature:	298 K		

	Calculated	Reported
Volume	2469.6(2)	2469.6(2)
Space group	P n m a	P n m a
Hall group	-P 2ac 2n	-P 2ac 2n
Moiety formula	C14 H11 Cl O3	2(C7 H5.5 Cl0.5 O1.5)
Sum formula	C14 H11 Cl O3	C14 H11 Cl O3
Mr	262.68	262.68
Dx,g cm-3	1.413	1.413
Z	8	8
Mu (mm-1)	0.306	0.306
F000	1088.0	1088.0
F000'	1089.71	
h,k,lmax	21,8,26	21,8,26
Nref	2768	2756
Tmin,Tmax	0.943,0.964	0.492,0.751
Tmin'	0.938	

Correction method= # Reported T Limits: Tmin=0.492 Tmax=0.751
AbsCorr = NONE

Data completeness= 0.996 Theta(max)= 26.453

R(reflections)= 0.0541(1681) wR2(reflections)= 0.1751(2756)

S = 1.051 Npar= 213

The following ALERTS were generated. Each ALERT has the format
test-name_ALERT_alert-type_alert-level.
Click on the hyperlinks for more details of the test.

Alert level C

```
PLAT042_ALERT_1_C Calc. and Reported MoietyFormula Strings  Differ     Please Check
              Calc: C14 H11 Cl O3
              Rep.: 2(C7 H5.5 Cl0.5 O1.5)
PLAT230_ALERT_2_C Hirshfeld Test Diff for    O15      --C20      .         5.2 s.u.
PLAT242_ALERT_2_C Low    'MainMol' Ueq as Compared to Neighbors of        C23 Check
PLAT340_ALERT_3_C Low Bond Precision on  C-C Bonds ...............    0.00518 Ang.
PLAT790_ALERT_4_C Centre of Gravity not Within Unit Cell: Resd.  #          1 Note
              C14 H11 Cl O3
PLAT911_ALERT_3_C Missing FCF Refl Between Thmin & STh/L=    0.600          4 Report
                0  2  0,   0  8  0,   2  0  0,  16  0  0,
```

Alert level G

```
PLAT790_ALERT_4_G Centre of Gravity not Within Unit Cell: Resd.  #          2 Note
              C14 H11 Cl O3
PLAT912_ALERT_4_G Missing # of FCF Reflections Above STh/L=  0.600          9 Note
PLAT913_ALERT_3_G Missing # of Very Strong Reflections in FCF ....          1 Note
                0  2  0,
PLAT969_ALERT_5_G The 'Henn et al.' R-Factor-gap value ...........      3.635 Note
              Predicted wR2: Based on SigI**2  4.82 or SHELX Weight 16.66
PLAT978_ALERT_2_G Number C-C Bonds with Positive Residual Density.          0 Info
PLAT992_ALERT_5_G Repd & Actual _reflns_number_gt Values Differ by          2 Check
```

0 **ALERT level A** = Most likely a serious problem - resolve or explain
0 **ALERT level B** = A potentially serious problem, consider carefully
6 **ALERT level C** = Check. Ensure it is not caused by an omission or oversight
6 **ALERT level G** = General information/check it is not something unexpected

1 ALERT type 1 CIF construction/syntax error, inconsistent or missing data
3 ALERT type 2 Indicator that the structure model may be wrong or deficient
3 ALERT type 3 Indicator that the structure quality may be low
3 ALERT type 4 Improvement, methodology, query or suggestion
2 ALERT type 5 Informative message, check

It is advisable to attempt to resolve as many as possible of the alerts in all categories. Often the minor alerts point to easily fixed oversights, errors and omissions in your CIF or refinement strategy, so attention to these fine details can be worthwhile. In order to resolve some of the more serious problems it may be necessary to carry out additional measurements or structure refinements. However, the purpose of your study may justify the reported deviations and the more serious of these should normally be commented upon in the discussion or experimental section of a paper or in the "special_details" fields of the CIF. checkCIF was carefully designed to identify outliers and unusual parameters, but every test has its limitations and alerts that are not important in a particular case may appear. Conversely, the absence of alerts does not guarantee there are no aspects of the results needing attention. It is up to the individual to critically assess their own results and, if necessary, seek expert advice.

Publication of your CIF in IUCr journals

A basic structural check has been run on your CIF. These basic checks will be run on all CIFs submitted for publication in IUCr journals (*Acta Crystallographica*, *Journal of Applied Crystallography*, *Journal of Synchrotron Radiation*); however, if you intend to submit to *Acta Crystallographica Section C* or *E* or *IUCrData*, you should make sure that full publication checks are run on the final version of your CIF prior to submission.

Publication of your CIF in other journals

Please refer to the *Notes for Authors* of the relevant journal for any special instructions relating to CIF submission.

PLATON version of 02/02/2025; check.def file version of 02/02/2025

Datablock 3d - ellipsoid plot

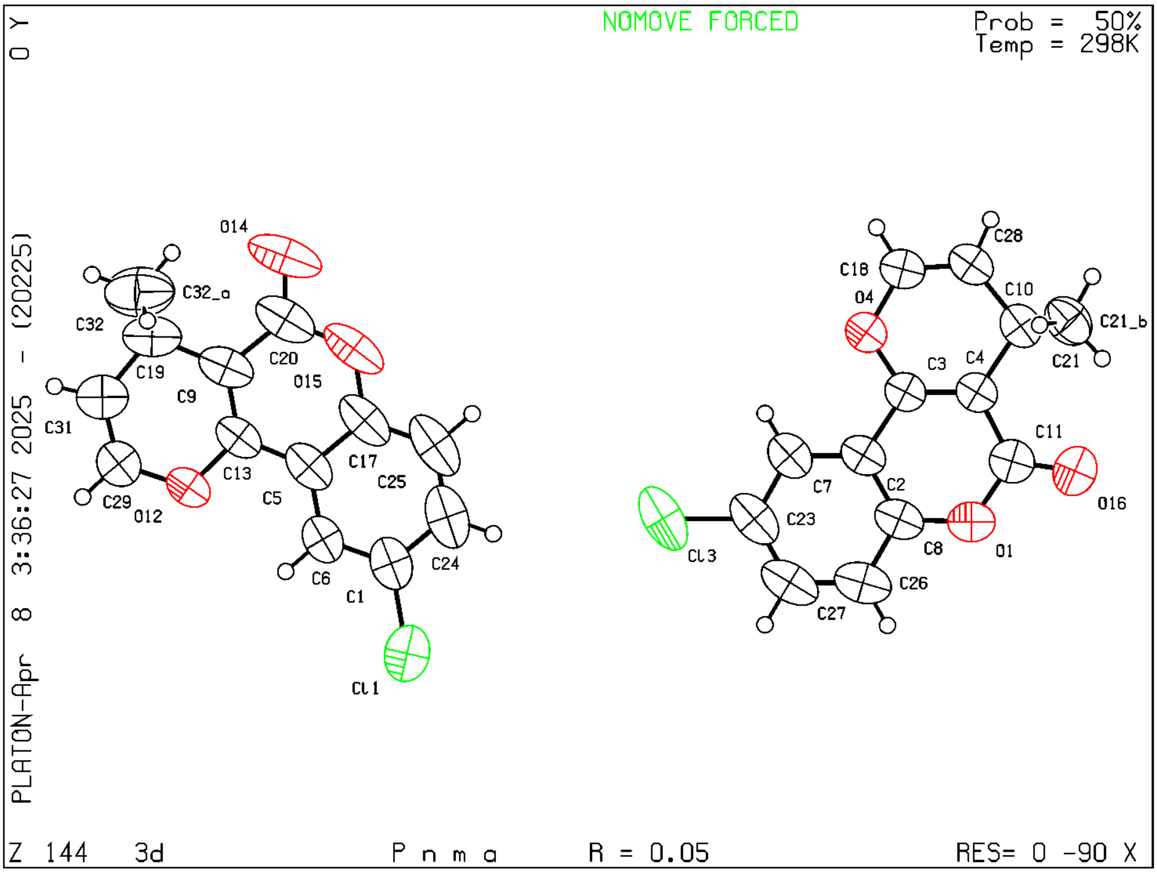

checkCIF/PLATON report

Structure factors have been supplied for datablock(s) 3k

THIS REPORT IS FOR GUIDANCE ONLY. IF USED AS PART OF A REVIEW PROCEDURE FOR PUBLICATION, IT SHOULD NOT REPLACE THE EXPERTISE OF AN EXPERIENCED CRYSTALLOGRAPHIC REFEREE.

No syntax errors found. CIF dictionary Interpreting this report

Datablock: 3k

Bond precision: C-C = 0.0055 A Wavelength=0.71073

Cell:	a=8.6358(13)	b=21.155(3)	c=6.8598(9)
	alpha=90	beta=90	gamma=90
Temperature:	295 K		

	Calculated	Reported
Volume	1253.2(3)	1253.2(3)
Space group	P b c m	P b c m
Hall group	-P 2c 2b	-P 2c 2b
Moiety formula	C14 H11 Br O3	C14 H11 Br O3
Sum formula	C14 H11 Br O3	C14 H11 Br O3
Mr	307.13	307.14
Dx,g cm-3	1.628	1.628
Z	4	4
Mu (mm-1)	3.277	3.276
F000	616.0	616.0
F000'	615.15	
h,k,lmax	10,25,8	10,25,8
Nref	1207	1186
Tmin,Tmax	0.606,0.697	0.404,0.735
Tmin'	0.567	

Correction method= # Reported T Limits: Tmin=0.404 Tmax=0.735
AbsCorr = NONE

Data completeness= 0.983 Theta(max)= 25.035

R(reflections)= 0.0511(1008) wR2(reflections)= 0.1383(1186)

S = 1.076 Npar= 107

The following ALERTS were generated. Each ALERT has the format
test-name_ALERT_alert-type_alert-level.
Click on the hyperlinks for more details of the test.

Alert level C

```
PLAT057_ALERT_3_C Correction for Absorption Required   RT(exp) ...       1.15 Do !
PLAT911_ALERT_3_C Missing FCF Refl Between Thmin & STh/L=    0.595         21 Report
                  3  0  0,   1  1  0,   2  1  0,   1  2  0,   1  3  0,   0  6  0,
                  4 11  0,   3 13  0,   1 16  0,   1  2  1,   1  4  1,   5  7  1,
                  6  7  1,   5  9  1,   0 14  1,   0  0  2,   3 11  2,   0 14  2,
                  0  0  4,   1  2  5,   0  0  6,
PLAT913_ALERT_3_C Missing # of Very Strong Reflections in FCF ....          5 Note
                  0  6  0,   1  2  1,   1  4  1,   0  0  2,   0  0  4,
```

Alert level G

```
PLAT720_ALERT_4_G Number of Unusual/Non-Standard Labels ..........          2 Note
              C0AA    H0AA
PLAT909_ALERT_3_G Percentage of I>2sig(I) Data at Theta(Max) Still        64% Note
PLAT969_ALERT_5_G The 'Henn et al.' R-Factor-gap value ..........      1.675 Note
              Predicted wR2: Based on SigI**2  8.26 or SHELX Weight 12.85
PLAT978_ALERT_2_G Number C-C Bonds with Positive Residual Density.          4 Info
```

0 **ALERT level A** = Most likely a serious problem - resolve or explain
0 **ALERT level B** = A potentially serious problem, consider carefully
3 **ALERT level C** = Check. Ensure it is not caused by an omission or oversight
4 **ALERT level G** = General information/check it is not something unexpected

0 ALERT type 1 CIF construction/syntax error, inconsistent or missing data
1 ALERT type 2 Indicator that the structure model may be wrong or deficient
4 ALERT type 3 Indicator that the structure quality may be low
1 ALERT type 4 Improvement, methodology, query or suggestion
1 ALERT type 5 Informative message, check

It is advisable to attempt to resolve as many as possible of the alerts in all categories. Often the minor alerts point to easily fixed oversights, errors and omissions in your CIF or refinement strategy, so attention to these fine details can be worthwhile. In order to resolve some of the more serious problems it may be necessary to carry out additional measurements or structure refinements. However, the purpose of your study may justify the reported deviations and the more serious of these should normally be commented upon in the discussion or experimental section of a paper or in the "special_details" fields of the CIF. checkCIF was carefully designed to identify outliers and unusual parameters, but every test has its limitations and alerts that are not important in a particular case may appear. Conversely, the absence of alerts does not guarantee there are no aspects of the results needing attention. It is up to the individual to critically assess their own results and, if necessary, seek expert advice.

Publication of your CIF in IUCr journals

A basic structural check has been run on your CIF. These basic checks will be run on all CIFs submitted for publication in IUCr journals (*Acta Crystallographica*, *Journal of Applied Crystallography*, *Journal of Synchrotron Radiation*); however, if you intend to submit to *Acta Crystallographica Section C* or *E* or *IUCrData*, you should make sure that full publication checks are run on the final version of your CIF prior to submission.

Publication of your CIF in other journals

Please refer to the *Notes for Authors* of the relevant journal for any special instructions relating to CIF submission.

PLATON version of 02/02/2025; check.def file version of 02/02/2025

Datablock 3k - ellipsoid plot

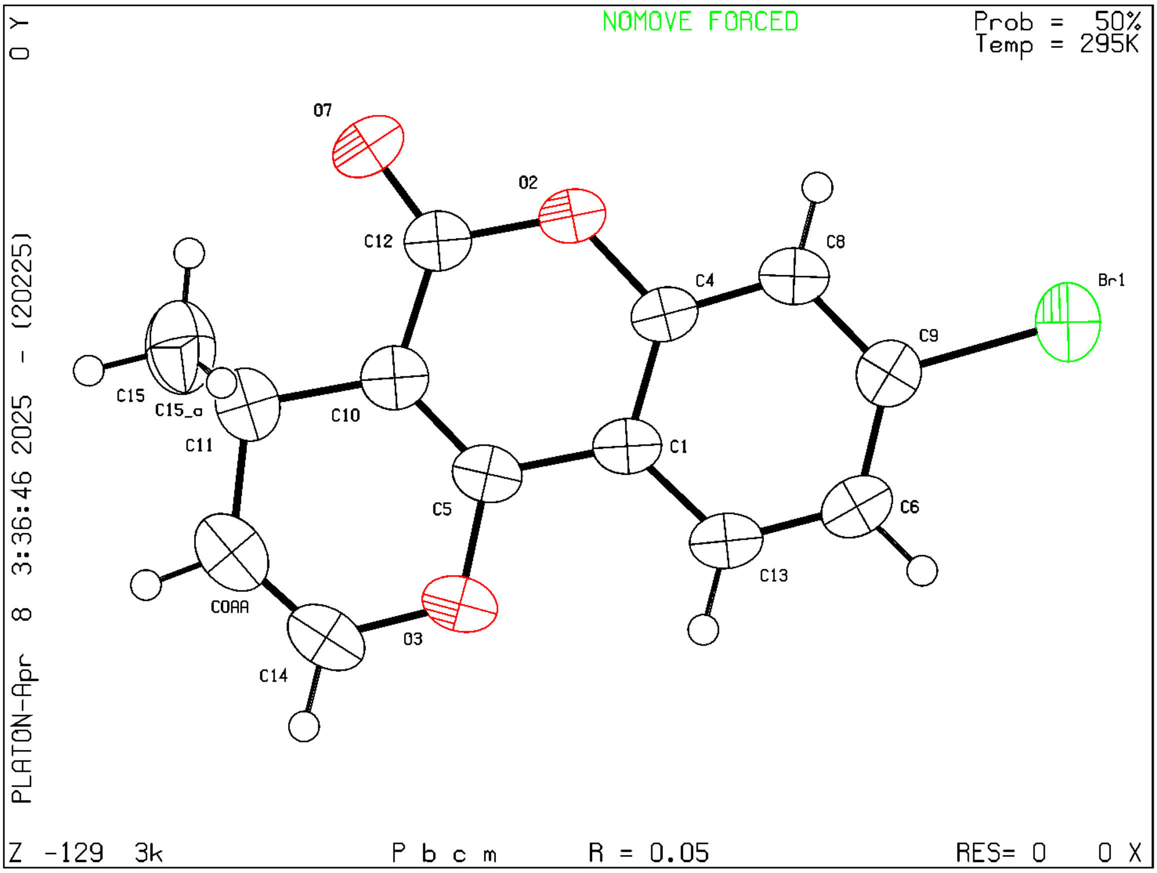

checkCIF/PLATON report

Structure factors have been supplied for datablock(s) 4d

THIS REPORT IS FOR GUIDANCE ONLY. IF USED AS PART OF A REVIEW PROCEDURE FOR PUBLICATION, IT SHOULD NOT REPLACE THE EXPERTISE OF AN EXPERIENCED CRYSTALLOGRAPHIC REFEREE.

No syntax errors found. CIF dictionary Interpreting this report

Datablock: 4d

```
Bond precision:   C-C = 0.0024 A           Wavelength=0.71073

Cell:            a=7.2396(5)        b=12.2973(10)      c=13.7364(11)
                 alpha=92.145(4)    beta=95.435(3)     gamma=97.982(4)
Temperature:     150 K

                  Calculated                 Reported
Volume            1204.04(16)                1204.04(16)
Space group       P -1                       P -1
Hall group        -P 1                       -P 1
Moiety formula    C14 H11 Cl O3              C14 H11 Cl O3
Sum formula       C14 H11 Cl O3              C14 H11 Cl O3
Mr                262.68                     262.68
Dx,g cm-3         1.449                      1.449
Z                 4                          4
Mu (mm-1)         0.313                      0.313
F000              544.0                      544.0
F000'             544.85
h,k,lmax          9,15,17                    9,15,17
Nref              5029                       4952
Tmin,Tmax         0.952,0.966                0.584,0.751
Tmin'             0.936

Correction method= # Reported T Limits: Tmin=0.584 Tmax=0.751
AbsCorr = NONE

Data completeness= 0.985           Theta(max)= 26.571

                                                wR2(reflections)=
R(reflections)= 0.0408( 3809)                   0.1149( 4952)

S = 1.068                  Npar= 329
```

The following ALERTS were generated. Each ALERT has the format
test-name_ALERT_alert-type_alert-level.
Click on the hyperlinks for more details of the test.

Alert level C

```
PLAT790_ALERT_4_C Centre of Gravity not Within Unit Cell: Resd.  #          1 Note
              C14 H11 Cl O3
PLAT906_ALERT_3_C Large K Value in the Analysis of Variance ......      2.146 Check
PLAT911_ALERT_3_C Missing FCF Refl Between Thmin & STh/L=    0.600         25 Report
                2  0  0,   2  1  0,   0 -1  1,   2 -1  1,   0  1  1,   1  1  1,
                2  1  1,   4  1  1,   7  3  1,   4  0  2,   2  1  2,  -1  2  3,
               -8  3  7,   3  8  8,   6 -1  9,  -7  4  9,   5 -3 10,   5 -2 10,
               -7  4 10,   4  4 10,  -7  5 10,   3  6 10,   4  2 11,  -6  6 11,
               -5  5 13,
```

Alert level G

```
PLAT720_ALERT_4_G Number of Unusual/Non-Standard Labels ..........         25 Note
              Cl0A    O3BA    C4BA    H4BA    O5BA    C6BA    H6BA    C7BA
              O8BA    C9BA    C1AA    C2AA    H2AA    O3AA    O4AA    C5AA
              H5AA    O6AA    C7AA    H7AA    C8AA    H8AA    C9AA    C0BA
              C2BA
PLAT790_ALERT_4_G Centre of Gravity not Within Unit Cell: Resd.  #          2 Note
              C14 H11 Cl O3
PLAT912_ALERT_4_G Missing # of FCF Reflections Above STh/L=  0.600         52 Note
PLAT913_ALERT_3_G Missing # of Very Strong Reflections in FCF ....          3 Note
                2  0  0,   2 -1  1,   2  1  1,
PLAT941_ALERT_3_G Average HKL Measurement Multiplicity ..........         3.3 Low
PLAT969_ALERT_5_G The 'Henn et al.' R-Factor-gap value ..........      1.724 Note
              Predicted wR2: Based on SigI**2  6.66 or SHELX Weight 10.75
PLAT978_ALERT_2_G Number C-C Bonds with Positive Residual Density.          5 Info
```

0 **ALERT level A** = Most likely a serious problem - resolve or explain
0 **ALERT level B** = A potentially serious problem, consider carefully
3 **ALERT level C** = Check. Ensure it is not caused by an omission or oversight
7 **ALERT level G** = General information/check it is not something unexpected

0 ALERT type 1 CIF construction/syntax error, inconsistent or missing data
1 ALERT type 2 Indicator that the structure model may be wrong or deficient
4 ALERT type 3 Indicator that the structure quality may be low
4 ALERT type 4 Improvement, methodology, query or suggestion
1 ALERT type 5 Informative message, check

It is advisable to attempt to resolve as many as possible of the alerts in all categories. Often the minor alerts point to easily fixed oversights, errors and omissions in your CIF or refinement strategy, so attention to these fine details can be worthwhile. In order to resolve some of the more serious problems it may be necessary to carry out additional measurements or structure refinements. However, the purpose of your study may justify the reported deviations and the more serious of these should normally be commented upon in the discussion or experimental section of a paper or in the "special_details" fields of the CIF. checkCIF was carefully designed to identify outliers and unusual parameters, but every test has its limitations and alerts that are not important in a particular case may appear. Conversely, the absence of alerts does not guarantee there are no aspects of the results needing attention. It is up to the individual to critically assess their own results and, if necessary, seek expert advice.

Publication of your CIF in IUCr journals

A basic structural check has been run on your CIF. These basic checks will be run on all CIFs submitted for publication in IUCr journals (*Acta Crystallographica*, *Journal of Applied Crystallography*, *Journal of Synchrotron Radiation*); however, if you intend to submit to *Acta Crystallographica Section C* or *E* or *IUCrData*, you should make sure that full publication checks are run on the final version of your CIF prior to submission.

Publication of your CIF in other journals

Please refer to the *Notes for Authors* of the relevant journal for any special instructions relating to CIF submission.

PLATON version of 02/02/2025; check.def file version of 02/02/2025

Datablock 4d - ellipsoid plot

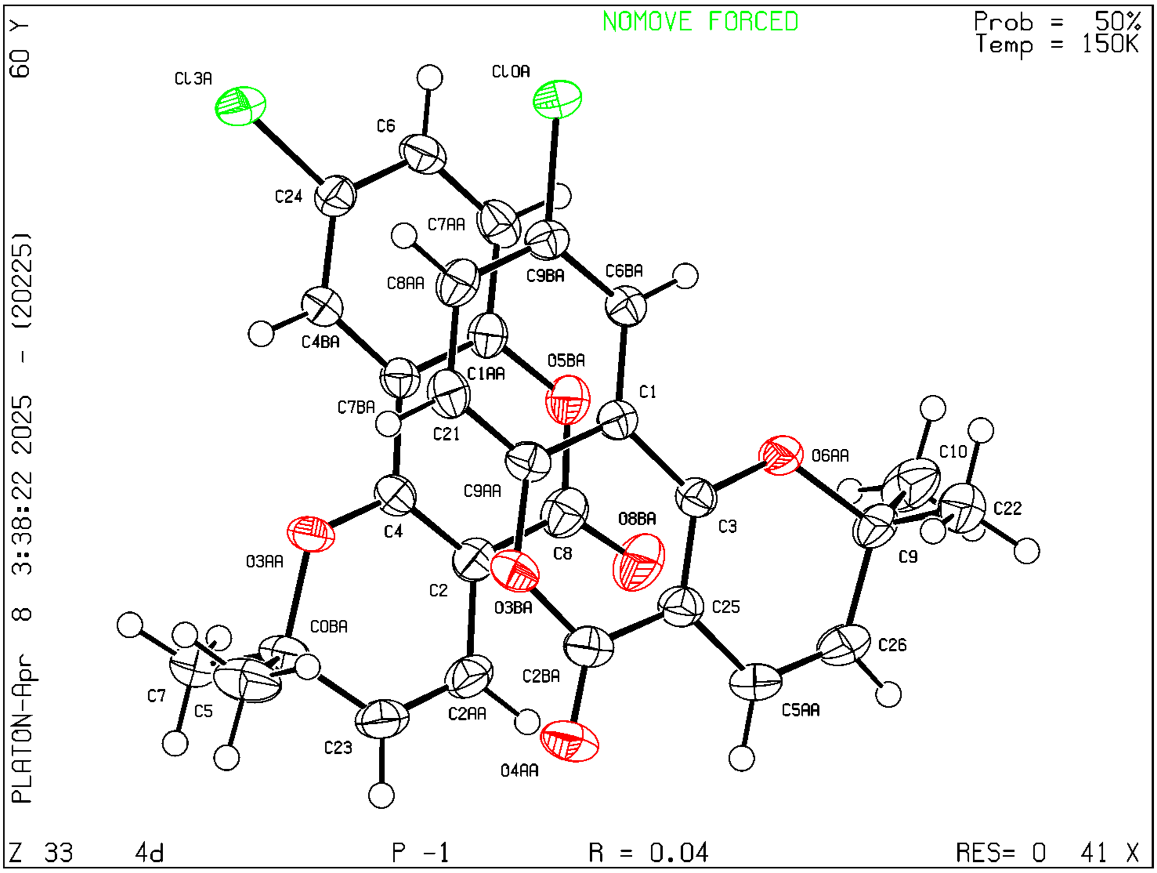

checkCIF/PLATON report

Structure factors have been supplied for datablock(s) 4e

THIS REPORT IS FOR GUIDANCE ONLY. IF USED AS PART OF A REVIEW PROCEDURE FOR PUBLICATION, IT SHOULD NOT REPLACE THE EXPERTISE OF AN EXPERIENCED CRYSTALLOGRAPHIC REFEREE.

No syntax errors found.   CIF dictionary   Interpreting this report

Datablock: 4e

```
Bond precision:   C-C = 0.0024 A           Wavelength=0.71073

Cell:             a=8.4159(6)      b=18.7306(16)      c=15.1115(13)
                  alpha=90         beta=99.176(4)     gamma=90
Temperature:      296 K

                 Calculated                   Reported
Volume           2351.6(3)                    2351.6(3)
Space group      C 2/c                        C 1 2/c 1
Hall group       -C 2yc                       -C 2yc
Moiety formula   C14 H11 F O3                 C14 H11 F O3
Sum formula      C14 H11 F O3                 C14 H11 F O3
Mr               246.23                       246.23
Dx,g cm-3        1.391                        1.391
Z                8                            8
Mu (mm-1)        0.108                        0.108
F000             1024.0                       1024.0
F000'            1024.65
h,k,lmax         10,22,18                     10,22,18
Nref             2319                         2261
Tmin,Tmax        0.977,0.985                  0.560,0.751
Tmin'            0.973

Correction method= # Reported T Limits: Tmin=0.560 Tmax=0.751
AbsCorr = NONE

Data completeness= 0.975            Theta(max)= 25.985

                                                     wR2(reflections)=
R(reflections)= 0.0456( 1562)                        0.1320( 2261)

S = 1.037                    Npar= 165
```

The following ALERTS were generated. Each ALERT has the format
test-name_ALERT_alert-type_alert-level.
Click on the hyperlinks for more details of the test.

Alert level C

```
PLAT906_ALERT_3_C Large K Value in the Analysis of Variance ......      4.111 Check
PLAT911_ALERT_3_C Missing FCF Refl Between Thmin & STh/L=    0.600        18 Report
                 1  3  0, -10  2  1,    5  3  1, -10  0  2,   -2  0  2,    2  0  2,
                 3 21  2, -10  0  4,   -2  0  4,    9  1  4,    9  1  5,    9  3  5,
                 7  1  8,    5 15  8,    7  1 10,    1 17 11,    5  1 14,    2  2 15,
```

Alert level G

```
PLAT910_ALERT_3_G Missing # of FCF Reflection(s) Below Theta(Min).          1 Note
                 0  2  0,
PLAT912_ALERT_4_G Missing # of FCF Reflections Above STh/L=  0.600         38 Note
PLAT913_ALERT_3_G Missing # of Very Strong Reflections in FCF ....          2 Note
                -2  0  2,  -2  0  4,
PLAT941_ALERT_3_G Average HKL Measurement Multiplicity ..........         3.8 Low
PLAT969_ALERT_5_G The 'Henn et al.' R-Factor-gap value ..........       3.661 Note
              Predicted wR2: Based on SigI**2  3.61 or SHELX Weight 12.72
PLAT978_ALERT_2_G Number C-C Bonds with Positive Residual Density.          0 Info
```

0 **ALERT level A** = Most likely a serious problem - resolve or explain
0 **ALERT level B** = A potentially serious problem, consider carefully
2 **ALERT level C** = Check. Ensure it is not caused by an omission or oversight
6 **ALERT level G** = General information/check it is not something unexpected

0 ALERT type 1 CIF construction/syntax error, inconsistent or missing data
1 ALERT type 2 Indicator that the structure model may be wrong or deficient
5 ALERT type 3 Indicator that the structure quality may be low
1 ALERT type 4 Improvement, methodology, query or suggestion
1 ALERT type 5 Informative message, check

It is advisable to attempt to resolve as many as possible of the alerts in all categories. Often the minor alerts point to easily fixed oversights, errors and omissions in your CIF or refinement strategy, so attention to these fine details can be worthwhile. In order to resolve some of the more serious problems it may be necessary to carry out additional measurements or structure refinements. However, the purpose of your study may justify the reported deviations and the more serious of these should normally be commented upon in the discussion or experimental section of a paper or in the "special_details" fields of the CIF. checkCIF was carefully designed to identify outliers and unusual parameters, but every test has its limitations and alerts that are not important in a particular case may appear. Conversely, the absence of alerts does not guarantee there are no aspects of the results needing attention. It is up to the individual to critically assess their own results and, if necessary, seek expert advice.

Publication of your CIF in IUCr journals

A basic structural check has been run on your CIF. These basic checks will be run on all CIFs submitted for publication in IUCr journals (*Acta Crystallographica*, *Journal of Applied Crystallography*, *Journal of Synchrotron Radiation*); however, if you intend to submit to *Acta Crystallographica Section C* or *E* or *IUCrData*, you should make sure that full publication checks are run on the final version of your CIF prior to submission.

Publication of your CIF in other journals

Please refer to the *Notes for Authors* of the relevant journal for any special instructions relating to CIF submission.

PLATON version of 02/02/2025; check.def file version of 02/02/2025

Datablock 4e - ellipsoid plot

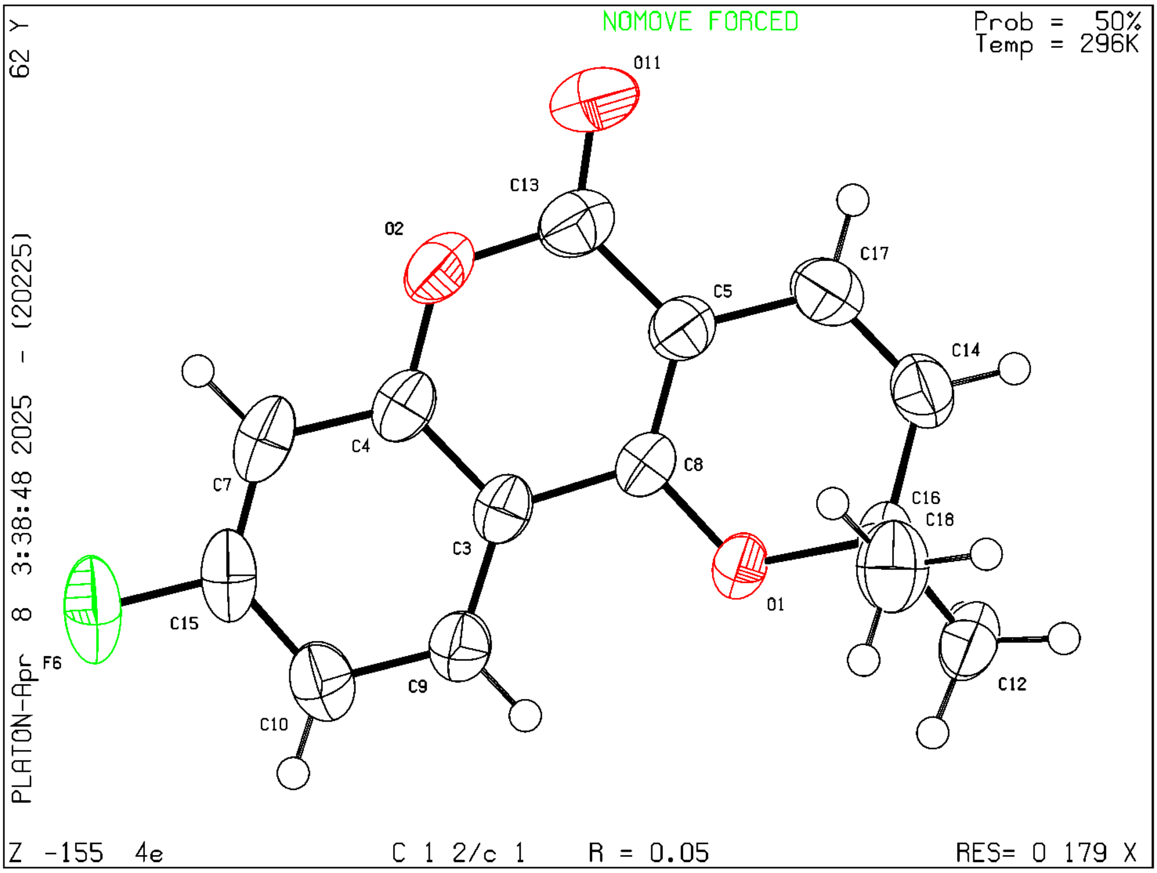

checkCIF/PLATON report

Structure factors have been supplied for datablock(s) 4g

THIS REPORT IS FOR GUIDANCE ONLY. IF USED AS PART OF A REVIEW PROCEDURE FOR PUBLICATION, IT SHOULD NOT REPLACE THE EXPERTISE OF AN EXPERIENCED CRYSTALLOGRAPHIC REFEREE.

No syntax errors found.   CIF dictionary   Interpreting this report

Datablock: 4g

Bond precision: C-C = 0.0198 A   Wavelength=0.71073

Cell:	a=7.1892(7)	b=12.3763(11)	c=14.3209(12)
	alpha=85.576(4)	beta=78.661(4)	gamma=81.573(4)
Temperature:	296 K		

	Calculated	Reported
Volume	1234.32(19)	1234.32(19)
Space group	P 1	P 1
Hall group	P 1	P 1
Moiety formula	C14 H11 Br O3	C14 H11 Br O3
Sum formula	C14 H11 Br O3	C14 H11 Br O3
Mr	307.13	307.14
Dx,g cm-3	1.653	1.653
Z	4	4
Mu (mm-1)	3.327	3.327
F000	616.0	616.0
F000'	615.15	
h,k,lmax	8,15,17	8,15,17
Nref	10046[5023]	9539
Tmin,Tmax	0.593,0.671	0.595,0.751
Tmin'	0.581	

Correction method= # Reported T Limits: Tmin=0.595 Tmax=0.751
AbsCorr = NONE

Data completeness= 1.90/0.95   Theta(max)= 26.365

R(reflections)= 0.0388(7382)   wR2(reflections)= 0.1051(9539)

S = 1.045   Npar= 657

The following ALERTS were generated. Each ALERT has the format
test-name_ALERT_alert-type_alert-level.
Click on the hyperlinks for more details of the test.

Alert level B

```
PLAT341_ALERT_3_B Low Bond Precision on  C-C Bonds ..............    0.01984 Ang.
```

Alert level C

```
STRVA01_ALERT_4_C           Flack test results are ambiguous.
           From the CIF: _refine_ls_abs_structure_Flack    0.410
           From the CIF: _refine_ls_abs_structure_Flack_su    0.020
PLAT057_ALERT_3_C Correction for Absorption Required   RT(exp) ...       1.13 Do !
PLAT090_ALERT_3_C Poor Data / Parameter Ratio (Zmax > 18) ........       7.58 Note
PLAT213_ALERT_2_C Atom C68             has ADP max/min Ratio .....        3.2 prolat
PLAT234_ALERT_4_C Large Hirshfeld Difference C14      --C54      .       0.16 Ang.
PLAT234_ALERT_4_C Large Hirshfeld Difference C26      --C32      .       0.16 Ang.
PLAT234_ALERT_4_C Large Hirshfeld Difference O8       --C28      .       0.17 Ang.
PLAT234_ALERT_4_C Large Hirshfeld Difference C28      --C51      .       0.18 Ang.
PLAT234_ALERT_4_C Large Hirshfeld Difference C57      --C63      .       0.20 Ang.
PLAT911_ALERT_3_C Missing FCF Refl Between Thmin & STh/L=    0.600         23 Report
                1  2  0,   0  4  0,  -1 -2  1,   0 -2  1,   2 -1  1,   2  0  1,
                2  1  1,   0  2  1,   1  3  1,  -1 -2  2,  -1 -1  2,   0 -1  2,
                1 -1  2,   2 -1  2,   0  1  2,   1  1  2,   2  1  2,   1 -2  3,
               -1 -1  3,   1  2  3,   0  0  4,   0 -3  5,   1-13  6,
PLAT913_ALERT_3_C Missing # of Very Strong Reflections in FCF ....          5 Note
                0  4  0,   2 -1  1,   2  0  1,   2 -1  2,   2  1  2,
PLAT987_ALERT_1_C The Flack x is >> 0 -  Do a BASF/TWIN Refinement     Please Check
```

Alert level G

```
PLAT033_ALERT_4_G Flack x Value Deviates > 3.0 * Sigma from Zero .      0.410 Note
PLAT111_ALERT_2_G ADDSYM Detects New (Pseudo) Centre of Symmetry .        100 %Fit
PLAT113_ALERT_2_G ADDSYM Suggests Possible Pseudo/New  Space Group        P-1 Check
              Check Model Parameter Symmetry for Reflection Data Support
PLAT154_ALERT_1_G The s.u.'s on the Cell Angles are Equal ..(Note)      0.004 Degree
PLAT910_ALERT_3_G Missing # of FCF Reflection(s) Below Theta(Min).          1 Note
                0  0  1,
PLAT912_ALERT_4_G Missing # of FCF Reflections Above STh/L=  0.600         19 Note
PLAT933_ALERT_2_G Number of HKL-OMIT Records in Embedded .res File          1 Note
                1  2  0,
PLAT941_ALERT_3_G Average HKL Measurement Multiplicity ..........         4.4 Low
PLAT969_ALERT_5_G The 'Henn et al.' R-Factor-gap value ..........       1.551 Note
              Predicted wR2: Based on SigI**2  6.78 or SHELX Weight 10.05
PLAT978_ALERT_2_G Number C-C Bonds with Positive Residual Density.          6 Info
```

---

0 **ALERT level A** = Most likely a serious problem - resolve or explain
1 **ALERT level B** = A potentially serious problem, consider carefully
12 **ALERT level C** = Check. Ensure it is not caused by an omission or oversight
10 **ALERT level G** = General information/check it is not something unexpected

2 ALERT type 1 CIF construction/syntax error, inconsistent or missing data

```
5 ALERT type 2 Indicator that the structure model may be wrong or deficient
7 ALERT type 3 Indicator that the structure quality may be low
8 ALERT type 4 Improvement, methodology, query or suggestion
1 ALERT type 5 Informative message, check
```

It is advisable to attempt to resolve as many as possible of the alerts in all categories. Often the minor alerts point to easily fixed oversights, errors and omissions in your CIF or refinement strategy, so attention to these fine details can be worthwhile. In order to resolve some of the more serious problems it may be necessary to carry out additional measurements or structure refinements. However, the purpose of your study may justify the reported deviations and the more serious of these should normally be commented upon in the discussion or experimental section of a paper or in the "special_details" fields of the CIF. checkCIF was carefully designed to identify outliers and unusual parameters, but every test has its limitations and alerts that are not important in a particular case may appear. Conversely, the absence of alerts does not guarantee there are no aspects of the results needing attention. It is up to the individual to critically assess their own results and, if necessary, seek expert advice.

Publication of your CIF in IUCr journals

A basic structural check has been run on your CIF. These basic checks will be run on all CIFs submitted for publication in IUCr journals (*Acta Crystallographica*, *Journal of Applied Crystallography*, *Journal of Synchrotron Radiation*); however, if you intend to submit to *Acta Crystallographica Section C* or *E* or *IUCrData*, you should make sure that full publication checks are run on the final version of your CIF prior to submission.

Publication of your CIF in other journals

Please refer to the *Notes for Authors* of the relevant journal for any special instructions relating to CIF submission.

PLATON version of 02/02/2025; check.def file version of 02/02/2025

Datablock 4g - ellipsoid plot

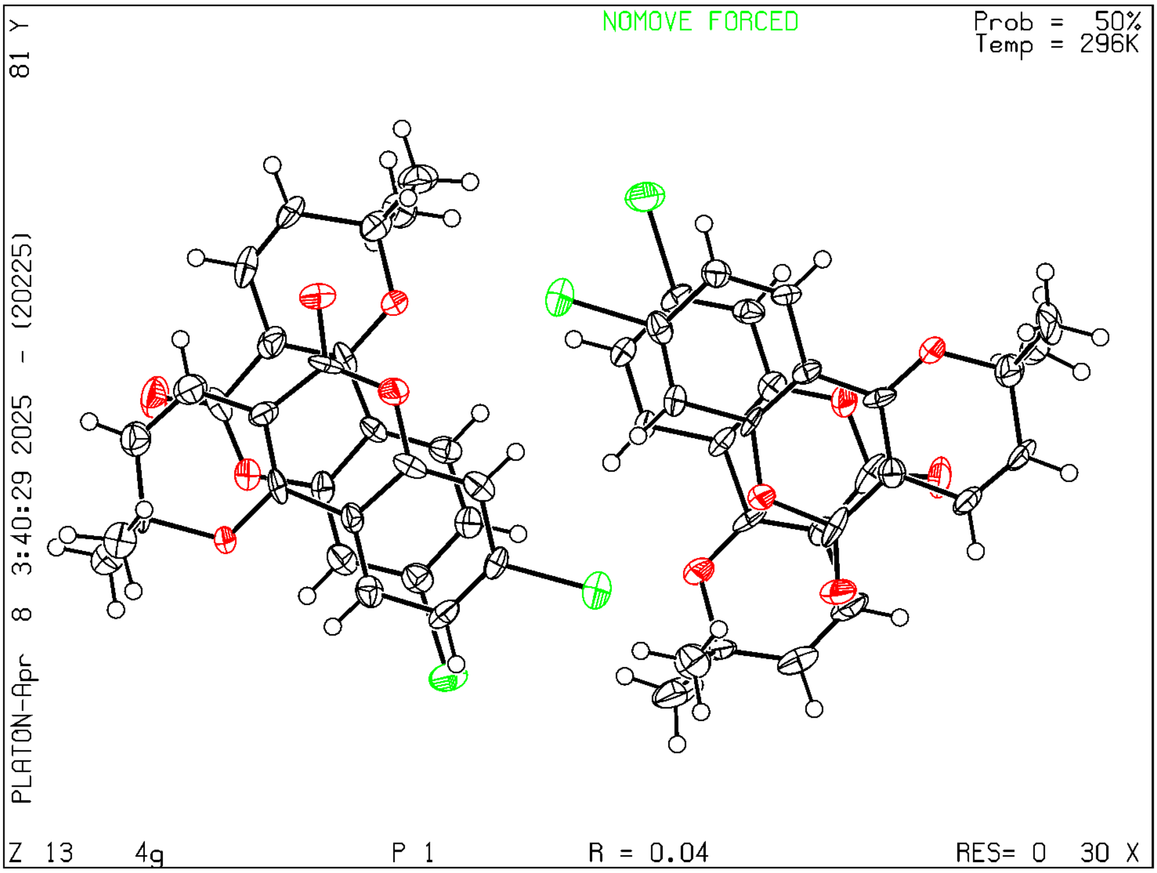

checkCIF/PLATON report

Structure factors have been supplied for datablock(s) 4k

THIS REPORT IS FOR GUIDANCE ONLY. IF USED AS PART OF A REVIEW PROCEDURE FOR PUBLICATION, IT SHOULD NOT REPLACE THE EXPERTISE OF AN EXPERIENCED CRYSTALLOGRAPHIC REFEREE.

No syntax errors found.   CIF dictionary   Interpreting this report

Datablock: 4k

```
Bond precision:   C-C = 0.0020 A           Wavelength=0.71073

Cell:              a=15.2572(4)      b=6.8430(2)      c=11.4579(3)
                   alpha=90          beta=90          gamma=90
Temperature:       296 K

                Calculated                  Reported
Volume          1196.26(6)                  1196.26(6)
Space group     P n m a                     P n m a
Hall group      -P 2ac 2n                   -P 2ac 2n
Moiety formula  C15 H14 O3                  C15 H14 O3
Sum formula     C15 H14 O3                  C15 H14 O3
Mr              242.26                      242.26
Dx,g cm-3       1.345                       1.345
Z               4                           4
Mu (mm-1)       0.093                       0.093
F000            512.0                       512.0
F000'           512.27
h,k,lmax        19,8,14                     19,8,14
Nref            1340                        1314
Tmin,Tmax       0.984,0.989                 0.594,0.751
Tmin'           0.981

Correction method= # Reported T Limits: Tmin=0.594 Tmax=0.751
AbsCorr = NONE

Data completeness= 0.981          Theta(max)= 26.432

                                                      wR2(reflections)=
R(reflections)= 0.0360( 1195)                         0.1002( 1314)

S = 1.053                    Npar= 108
```

The following ALERTS were generated. Each ALERT has the format
test-name_ALERT_alert-type_alert-level.
Click on the hyperlinks for more details of the test.

Alert level C

```
PLAT790_ALERT_4_C Centre of Gravity not Within Unit Cell: Resd.  #          1 Note
              C15 H14 O3
PLAT911_ALERT_3_C Missing FCF Refl Between Thmin & STh/L=    0.600         14 Report
                0  2  0,   0  4  0,   0  6  0,   4  2  0,   0  1  1,   1  1  1,
                1  2  1,   3  0  1,   0  0  2,   1  0  2,   2  0  2,   2  1  2,
                4  0  2,   0  0  4,
PLAT913_ALERT_3_C Missing # of Very Strong Reflections in FCF ....          7 Note
                0  2  0,   0  4  0,   0  6  0,   1  0  1,   1  1  1,   1  2  1,
                0  0  4,
```

Alert level G

```
PLAT299_ALERT_4_G Atom Site Occupancy Constrained at ............        0.5 Check
              H20A    H20B    H20C
PLAT367_ALERT_2_G Long?  C(sp?)-C(sp?) Bond  C1AA     - C20      .       1.50 Ang.
PLAT720_ALERT_4_G Number of Unusual/Non-Standard Labels .........         16 Note
              C1AA    C3AA    C0AA    H0AA    O5AA    O7AA    C2AA    C8AA
              H8AA    C4AA    C6AA    H6AA    C0BA    H0BA    C9AA    H9AA
PLAT789_ALERT_4_G Atoms with Negative _atom_site_disorder_group  #          3 Check
PLAT822_ALERT_4_G CIF-embedded .res Contains Negative PART Numbers          1 Check
PLAT910_ALERT_3_G Missing # of FCF Reflection(s) Below Theta(Min).          2 Note
                2  0  0,   1  0  1,
PLAT912_ALERT_4_G Missing # of FCF Reflections Above STh/L=  0.600         10 Note
PLAT933_ALERT_2_G Number of HKL-OMIT Records in Embedded .res File          1 Note
                1  0  1,
PLAT969_ALERT_5_G The 'Henn et al.' R-Factor-gap value ..........      3.896 Note
              Predicted wR2: Based on SigI**2  2.57 or SHELX Weight  9.51
PLAT978_ALERT_2_G Number C-C Bonds with Positive Residual Density.         13 Info
```

```
   0 ALERT level A = Most likely a serious problem - resolve or explain
   0 ALERT level B = A potentially serious problem, consider carefully
   3 ALERT level C = Check. Ensure it is not caused by an omission or oversight
  10 ALERT level G = General information/check it is not something unexpected

   0 ALERT type 1 CIF construction/syntax error, inconsistent or missing data
   3 ALERT type 2 Indicator that the structure model may be wrong or deficient
   3 ALERT type 3 Indicator that the structure quality may be low
   6 ALERT type 4 Improvement, methodology, query or suggestion
   1 ALERT type 5 Informative message, check
```

It is advisable to attempt to resolve as many as possible of the alerts in all categories. Often the minor alerts point to easily fixed oversights, errors and omissions in your CIF or refinement strategy, so attention to these fine details can be worthwhile. In order to resolve some of the more serious problems it may be necessary to carry out additional measurements or structure refinements. However, the purpose of your study may justify the reported deviations and the more serious of these should normally be commented upon in the discussion or experimental section of a paper or in the "special_details" fields of the CIF. checkCIF was carefully designed to identify outliers and unusual parameters, but every test has its limitations and alerts that are not important in a particular case may appear. Conversely, the absence of alerts does not guarantee there are no aspects of the results needing attention. It is up to the individual to critically assess their own results and, if necessary, seek expert advice.

Publication of your CIF in IUCr journals

A basic structural check has been run on your CIF. These basic checks will be run on all CIFs submitted for publication in IUCr journals (*Acta Crystallographica*, *Journal of Applied Crystallography*, *Journal of Synchrotron Radiation*); however, if you intend to submit to *Acta Crystallographica Section C* or *E* or *IUCrData*, you should make sure that full publication checks are run on the final version of your CIF prior to submission.

Publication of your CIF in other journals

Please refer to the *Notes for Authors* of the relevant journal for any special instructions relating to CIF submission.

PLATON version of 02/02/2025; check.def file version of 02/02/2025

Datablock 4k - ellipsoid plot

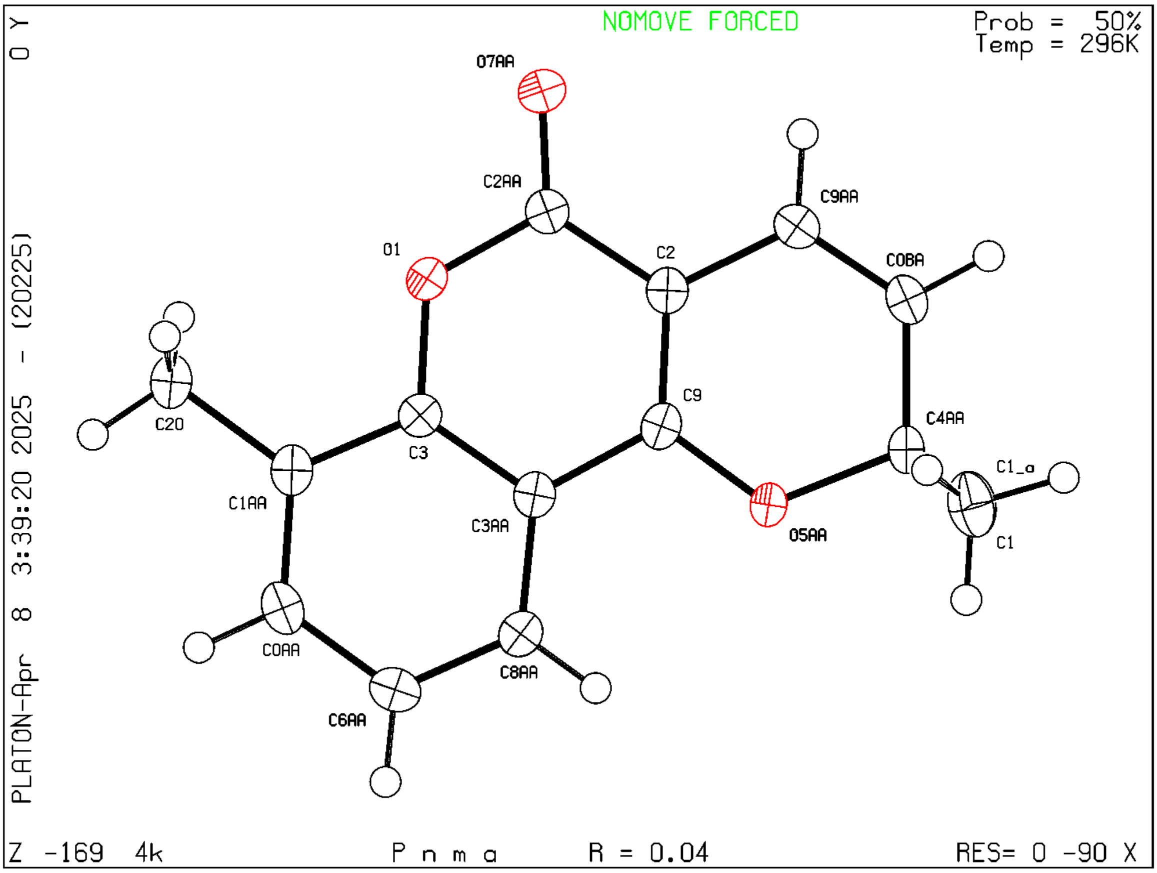

checkCIF/PLATON report

Structure factors have been supplied for datablock(s) 10d

THIS REPORT IS FOR GUIDANCE ONLY. IF USED AS PART OF A REVIEW PROCEDURE FOR PUBLICATION, IT SHOULD NOT REPLACE THE EXPERTISE OF AN EXPERIENCED CRYSTALLOGRAPHIC REFEREE.

No syntax errors found. CIF dictionary Interpreting this report

Datablock: 10d

Bond precision: C-C = 0.0054 A Wavelength=0.71073

Cell:	a=17.3667(12)	b=6.0766(4)	c=16.2575(16)
	alpha=90	beta=91.591(5)	gamma=90
Temperature:	298 K		

	Calculated	Reported
Volume	1715.0(2)	1715.0(2)
Space group	P 21/c	P 1 21/c 1
Hall group	-P 2ybc	-P 2ybc
Moiety formula	C20 H17 Br N2 O	C20 H17 Br N2 O
Sum formula	C20 H17 Br N2 O	C20 H17 Br N2 O
Mr	381.26	381.26
Dx,g cm-3	1.477	1.477
Z	4	4
Mu (mm-1)	2.405	2.405
F000	776.0	776.0
F000’	775.12	
h,k,lmax	21,7,20	21,7,20
Nref	3529	3507
Tmin,Tmax	0.655,0.749	0.357,0.751
Tmin’	0.556	

Correction method= # Reported T Limits: Tmin=0.357 Tmax=0.751
AbsCorr = NONE

Data completeness= 0.994 Theta(max)= 26.421

R(reflections)= 0.0662(2779) wR2(reflections)= 0.2136(3507)

S = 1.113 Npar= 219

The following ALERTS were generated. Each ALERT has the format
test-name_ALERT_alert-type_alert-level.
Click on the hyperlinks for more details of the test.

Alert level C

```
PLAT057_ALERT_3_C Correction for Absorption Required   RT(exp) ...       1.14 Do !
PLAT911_ALERT_3_C Missing FCF Refl Between Thmin & STh/L=    0.600         11 Report
                4  1  0,  -2  1  1,  -3  1  2,  -1  0  2,  -1  1  2,   0  1  2,
                1  0  2,  -4  0  4,  -3  0  4,  -2  0  4,  -6  0  8,
PLAT913_ALERT_3_C Missing # of Very Strong Reflections in FCF ....          7 Note
                4  1  0,  -2  1  1,  -3  1  2,   1  0  2,  -4  0  4,  -3  0  4,
               -2  0  4,
```

Alert level G

```
PLAT072_ALERT_2_G SHELXL First  Parameter in WGHT  Unusually Large       0.14 Report
PLAT910_ALERT_3_G Missing # of FCF Reflection(s) Below Theta(Min).          1 Note
                1  0  0,
PLAT912_ALERT_4_G Missing # of FCF Reflections Above STh/L=  0.600         11 Note
PLAT933_ALERT_2_G Number of HKL-OMIT Records in Embedded .res File          1 Note
                1  0  0,
PLAT941_ALERT_3_G Average HKL Measurement Multiplicity ..........         4.5 Low
PLAT969_ALERT_5_G The 'Henn et al.' R-Factor-gap value ..........       3.510 Note
              Predicted wR2: Based on SigI**2  6.09 or SHELX Weight 19.20
PLAT978_ALERT_2_G Number C-C Bonds with Positive Residual Density.          5 Info
```

0 **ALERT level A** = Most likely a serious problem - resolve or explain
0 **ALERT level B** = A potentially serious problem, consider carefully
3 **ALERT level C** = Check. Ensure it is not caused by an omission or oversight
7 **ALERT level G** = General information/check it is not something unexpected

0 ALERT type 1 CIF construction/syntax error, inconsistent or missing data
3 ALERT type 2 Indicator that the structure model may be wrong or deficient
5 ALERT type 3 Indicator that the structure quality may be low
1 ALERT type 4 Improvement, methodology, query or suggestion
1 ALERT type 5 Informative message, check

It is advisable to attempt to resolve as many as possible of the alerts in all categories. Often the minor alerts point to easily fixed oversights, errors and omissions in your CIF or refinement strategy, so attention to these fine details can be worthwhile. In order to resolve some of the more serious problems it may be necessary to carry out additional measurements or structure refinements. However, the purpose of your study may justify the reported deviations and the more serious of these should normally be commented upon in the discussion or experimental section of a paper or in the "special_details" fields of the CIF. checkCIF was carefully designed to identify outliers and unusual parameters, but every test has its limitations and alerts that are not important in a particular case may appear. Conversely, the absence of alerts does not guarantee there are no aspects of the results needing attention. It is up to the individual to critically assess their own results and, if necessary, seek expert advice.

Publication of your CIF in IUCr journals

A basic structural check has been run on your CIF. These basic checks will be run on all CIFs submitted for publication in IUCr journals (*Acta Crystallographica*, *Journal of Applied Crystallography*, *Journal of Synchrotron Radiation*); however, if you intend to submit to *Acta Crystallographica Section C* or *E* or *IUCrData*, you should make sure that full publication checks are run on the final version of your CIF prior to submission.

Publication of your CIF in other journals

Please refer to the *Notes for Authors* of the relevant journal for any special instructions relating to CIF submission.

PLATON version of 02/02/2025; check.def file version of 02/02/2025

Datablock 10d - ellipsoid plot

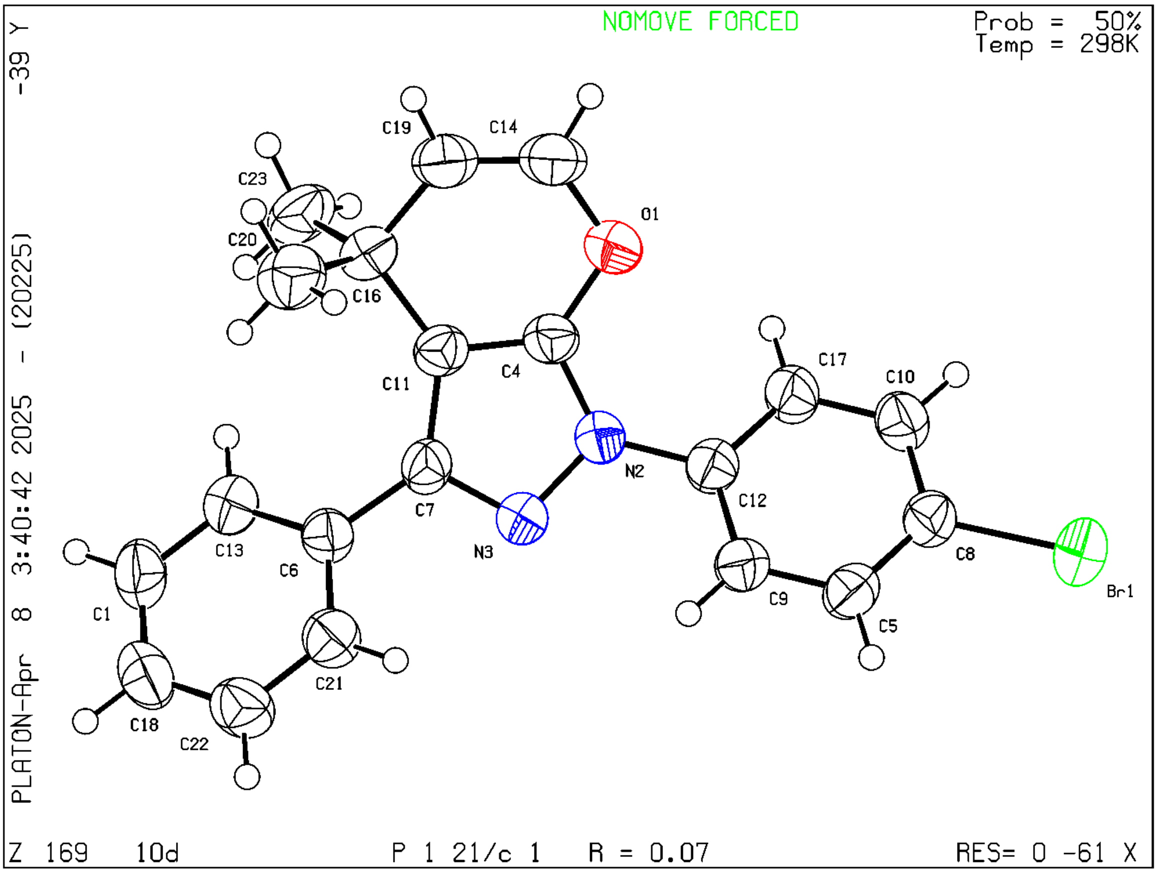

checkCIF/PLATON report

Structure factors have been supplied for datablock(s) 10k

THIS REPORT IS FOR GUIDANCE ONLY. IF USED AS PART OF A REVIEW PROCEDURE FOR PUBLICATION, IT SHOULD NOT REPLACE THE EXPERTISE OF AN EXPERIENCED CRYSTALLOGRAPHIC REFEREE.

No syntax errors found. CIF dictionary Interpreting this report

Datablock: 10k

Bond precision: C-C = 0.0021 A Wavelength=0.71073

Cell:	a=6.0254(4)	b=11.7227(8)	c=11.9718(9)
	alpha=77.622(3)	beta=80.706(3)	gamma=75.577(3)
Temperature:	296 K		

	Calculated	Reported
Volume	794.69(10)	794.69(10)
Space group	P -1	P -1
Hall group	-P 1	-P 1
Moiety formula	C20 H17 F N2 O	C20 H17 F N2 O
Sum formula	C20 H17 F N2 O	C20 H17 F N2 O
Mr	320.36	320.35
Dx,g cm-3	1.339	1.339
Z	2	2
Mu (mm-1)	0.092	0.092
F000	336.0	336.0
F000’	336.15	
h,k,lmax	7,14,15	7,14,14
Nref	3288	3199
Tmin,Tmax	0.983,0.989	0.631,0.751
Tmin’	0.983	

Correction method= # Reported T Limits: Tmin=0.631 Tmax=0.751
AbsCorr = NONE

Data completeness= 0.973 Theta(max)= 26.452

R(reflections)= 0.0440(2686) wR2(reflections)= 0.1258(3199)

S = 1.087 Npar= 219

The following ALERTS were generated. Each ALERT has the format
test-name_ALERT_alert-type_alert-level.
Click on the hyperlinks for more details of the test.

Alert level C

```
PLAT790_ALERT_4_C Centre of Gravity not Within Unit Cell: Resd.  #          1 Note
              C20 H17 F N2 O
PLAT911_ALERT_3_C Missing FCF Refl Between Thmin & STh/L=    0.600         54 Report
                1  0  0,   2  0  0,   1  1  0,   2  2  0,   0  3  0,   1  3  0,
                2  3  0,   1  4  0,  -1 -3  1,   0 -3  1,  -2 -2  1,  -1 -2  1,
                0 -2  1,  -1 -1  1,  -1  0  1,   1  0  1,  -1  1  1,   1  1  1,
                0  2  1,   0  3  1,   1  3  1,   1  4  1,   0 -3  2,  -1 -2  2,
                0 -2  2,  -2 -1  2,  -1 -1  2,   0 -1  2,  -1  0  2,   0  1  2,
                1  3  2,   0 12  2,  -1 -1  3,   0 -1  3,   1 -1  3,  -1  0  3,
                1  1  3,   0  2  3,   3 -8  4,  -1 -1  4,  -5 -7  5,  -5 -6  6,
               -5 -4  7,   0 11  7,   1 11  8,  -1 -4 10,   0 11 10,   3 -1 11,
                2  0 12,   3  4 12,   0 -3 13,   1 -2 13,   2 -2 13,   2 -1 13,
PLAT913_ALERT_3_C Missing # of Very Strong Reflections in FCF ....         13 Note
                1  0  0,   2  0  0,   0  3  0,   1  3  0,  -1 -3  1,  -1 -2  1,
                1  0  1,   0  1  1,   0  3  1,   1  3  1,  -1 -2  2,   0 -2  2,
               -1 -1  2,
PLAT934_ALERT_3_C Number of (Iobs-Icalc)/Sigma(W) > 10 Outliers ..          1 Check
                1  3  4,
```

Alert level G

```
PLAT154_ALERT_1_G The s.u.'s on the Cell Angles are Equal ..(Note)      0.003 Degree
PLAT910_ALERT_3_G Missing # of FCF Reflection(s) Below Theta(Min).          4 Note
                0  1  0,   0 -1  1,   0  0  1,   0  1  1,
PLAT912_ALERT_4_G Missing # of FCF Reflections Above STh/L=  0.600         29 Note
PLAT933_ALERT_2_G Number of HKL-OMIT Records in Embedded .res File          2 Note
                1  3  2,   0 -1  1,
PLAT941_ALERT_3_G Average HKL Measurement Multiplicity ..........         3.1 Low
PLAT969_ALERT_5_G The 'Henn et al.' R-Factor-gap value ..........       2.458 Note
              Predicted wR2: Based on SigI**2  5.12 or SHELX Weight 11.58
PLAT978_ALERT_2_G Number C-C Bonds with Positive Residual Density.          7 Info
```

0 **ALERT level A** = Most likely a serious problem - resolve or explain
0 **ALERT level B** = A potentially serious problem, consider carefully
4 **ALERT level C** = Check. Ensure it is not caused by an omission or oversight
7 **ALERT level G** = General information/check it is not something unexpected

1 ALERT type 1 CIF construction/syntax error, inconsistent or missing data
2 ALERT type 2 Indicator that the structure model may be wrong or deficient
5 ALERT type 3 Indicator that the structure quality may be low
2 ALERT type 4 Improvement, methodology, query or suggestion
1 ALERT type 5 Informative message, check

It is advisable to attempt to resolve as many as possible of the alerts in all categories. Often the minor alerts point to easily fixed oversights, errors and omissions in your CIF or refinement strategy, so attention to these fine details can be worthwhile. In order to resolve some of the more serious problems it may be necessary to carry out additional measurements or structure refinements. However, the purpose of your study may justify the reported deviations and the more serious of these should normally be commented upon in the discussion or experimental section of a paper or in the "special_details" fields of the CIF. checkCIF was carefully designed to identify outliers and unusual parameters, but every test has its limitations and alerts that are not important in a particular case may appear. Conversely, the absence of alerts does not guarantee there are no aspects of the results needing attention. It is up to the individual to critically assess their own results and, if necessary, seek expert advice.

Publication of your CIF in IUCr journals

A basic structural check has been run on your CIF. These basic checks will be run on all CIFs submitted for publication in IUCr journals (*Acta Crystallographica*, *Journal of Applied Crystallography*, *Journal of Synchrotron Radiation*); however, if you intend to submit to *Acta Crystallographica Section C* or *E* or *IUCrData*, you should make sure that full publication checks are run on the final version of your CIF prior to submission.

Publication of your CIF in other journals

Please refer to the *Notes for Authors* of the relevant journal for any special instructions relating to CIF submission.

PLATON version of 02/02/2025; check.def file version of 02/02/2025

Datablock 10k - ellipsoid plot

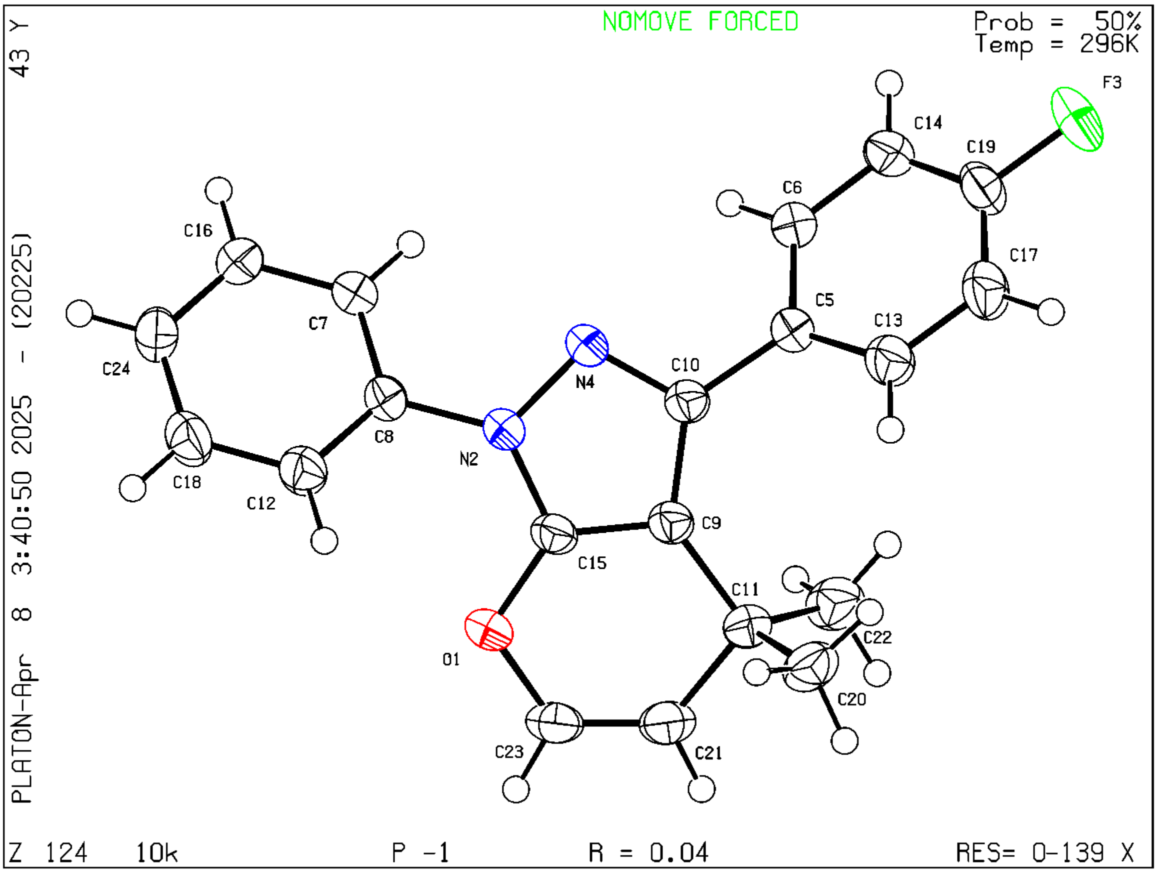

Supplement: Supplementary file 2 — Supplemental Data [file ADVS-12-e11331-s002.zip › checkcif.pdf]
